# SARS‐CoV‐2 infection and venous thromboembolism after surgery: an international prospective cohort study

**DOI:** 10.1111/anae.15563

**Published:** 2021-08-24

**Authors:** COVIDSurg Collaborative, Dmitri Nepogodiev, Joana FF Simoes, Elizabeth Li, Maria Picciochi, James C Glasbey, Glauco Baiocchi, Ruth Blanco‐Colino, Daoud Chaudhry, Ehab AlAmeer, Kariem El‐Boghdadly, Funmilola Wuraola, Dhruva Ghosh, Rohan R Gujjuri, Ewen M Harrison, Herman Lule, Haytham Kaafarani, Mohammad Khosravi, Irmgard Kronberger, Sezai Leventoğlu, Harvinder Mann, Kenneth A Mclean, Mengistu Gebreyohanes Mengesha, Maria Marta Modolo, Faustin Ntirenganya, Lisa Norman, Oumaima Outani, Riinu Pius, Peter Pockney, Ahmad Uzair Qureshi, April Camilla Roslani, Sohei Satoi, Catherine Shaw, Aneel Bhangu, Dmitri Nepogodiev, Omar M Omar, Waheed‐Ul‐Rahman Ahmed, Leah Argus, Alasdair Ball, Aneel Bhangu, Edward P Bywater, Ruth Blanco‐Colino, Amanpreet Brar, Daoud Chaudhry, Brett E Dawson, Irani Duran, Muhammed Elhadi, James C Glasbey, Rohan R Gujjuri, Conor S Jones, Ewen M Harrison, Sivesh K Kamarajah, James M Keatley, Samuel Lawday, Elizabeth Li, Harvinder Mann, Ella J Marson, Kenneth A Mclean, Dmitri Nepogodiev, Lisa Norman, Riinu Ots, Oumaima Outani, Maria Picciochi, Irène Santos, Catherine Shaw, Joana FF Simoes, Elliott H Taylor, Isobel M Trout, Chris Varghese, Mary L Venn, William Xu, Joana FF Simoes, Irida Dajti, Arben Gjata, Salah Eddine Oussama Kacimi, Luis Boccalatte, Maria Marta Modolo, Daniel Cox, Peter Pockney, Philip Townend, Felix Aigner, Irmgard Elisabeth Kronberger, Elgun Samadov, Amer Alderazi, Kamral Hossain, Greg Padmore, Gabrielle van Ramshorst, Ismaïl Lawani, Anis Cerovac, Samir Delibegovic, Glauco Baiocchi, Gustavo Mendonça Ataíde Gomes, Igor Lima Buarque, Muhammad Gohar, Mihail Slavchev, Chukwuemeka Nwegbu, Arnav Agarwal, Amanpreet Brar, Janet Martin, Joshua Ng‐Kamstra, Maria Marta Modolo, Maricarmen Olivos, Wenhui Lou, Dong‐Lin Ren, Jose Andres Calvache, Carlos J Perez Rivera, Ana Danic Hadzibegovic, Tomislav Kopjar, Jakov Mihanovic, Pablo Mijahil Avilés Jiménez, Nikolaos Gouvas, Jaroslav Klat, René Novysedlák, Nicolas Amisi, Peter Christensen, Alaa El‐Hussuna, Sylvia Batista, Eddy Lincango‐Naranjo, Sameh Emile, Danilo Alfonso Arévalo Sandoval, Hailu Dhufera, Samuel Hailu, Mengistu G Mengesha, Joonas H Kauppila, Alexis P Arnaud, Zaza Demetrashvili, Markus Albertsmeier, Hans Lederhuber, Markus W. Löffler, Daniel Kwesi Acquah, Bernard Ofori, Stephen Tabiri, Symeon Metallidis, Georgios Tsoulfas, Maria‐Lorena Aguilera‐Arevalo, Gustavo Recinos, Tamás Mersich, Dániel Wettstein, Dhruva Ghosh, Gabriele Kembuan, Peiman Brouki Milan, Mohammad Hossein Khosravi, Masoud Mozafari, Ahmed Hilmi, Helen Mohan, Oded Zmora, Gaetano Gallo, Francesco Pata, Gianluca Pellino, Yuki Fujimoto, Naoto Kuroda, Sohei Satoi, Mohamad K. Abou Chaar, Faris Ayasra, Ildar Fakhradiyev, Intisar Hisham Said Hamdun, Jang Jin‐Young, Mohammad Jamal, Lina Karout, Muhammed Elhadi, Aiste Gulla, Fanjandrainy Rasoaherinomenjanahary, Luc Hervé Samison, April Camilla Roslani, Iran Irani Durán Sánchez, Diana Samantha Gonzalez, Laura Martinez, María José Martínez, Alejandra Nayen, Antonio Ramos‐De la Medina, Jade Nunez, Oumaima Outani, Pueya Rashid Nashidengo, Rakesh Shah, Ashish Lal Shrestha, Pascal Jonker, Schelto Kruijff, Milou Noltes, Pieter Steinkamp, Chris Varghese, Deborah Wright, Lukman Abdur‐Rahman, Adesoji Ademuyiwa, Adewale Adisa, Babatunde Osinaike, Justina Seyi‐Olajide, Omolara Williams, Emmanuel Williams, Sofija Pejkova, Zainab Al Balushi, Ahmad Uzair Qureshi, Raza Sayyed, Mustafa Abo Mohsen, Sadi A. Abukhalaf, Moises Cukier, Hugo Gomez‐Fernandez, Sebastian Shu Yip, Ximena Paola Vasquez Ojeda, Marie Dione Sacdalan, Piotr Major, José Azevedo, Miguel F. Cunha, Irène Santos, Ahmad Zarour, Eduard‐Alexandru Bonci, Ionut Negoi, Sergey Efetov, Viktor Kochetkov, Andrey Litvin, Jc Allen Ingabire, Georges Bucyibaruta, Ntirenganya Faustin, Sosthene Habumuremyi, Alphonsine Imanishimwe, Haragirimana Jean de Dieu, Emmanuel Munyaneza, Isaie Ncogoza, Ehab Alameer, Abdourahmane Ndong, Dejan Radenkovic, Min Hoe Chew, Frederick Koh, James Ngu, Arpád Panyko, Uros Bele, Jurij Aleš Košir, Hassan Daoud, Ruth Blanco‐Colino, Ana Maria Minaya Bravo, Umesh Jayarajah, Dakshitha Wickramasinghe, Mohammed Elmujtba Adam Essa Adam, Martin Rutegård, Malin Sund, Michel Adamina, Eleftherios Gialamas, Karoline Horisberger, Muhammad Alshaar, Abel Huang, Varut Lohsiriwat, Shane Charles, Haithem Jlassi, Arda Isik, Sezai Leventoğlu, Hervé Monka Lekuya, Hervé Monka Lekuya, Herman Lule, Slava Kopetskyi, Hayder Alsaadi, Sattar Alshryda, Osaid Alser, Brittany Bankhead‐Kendall, Kerry Breen, Haytham Kaafarani, Hassan Mashbari, Fernando Bonilla Cal, Hamza Al‐Naggar, Mayaba Maimbo, Dennis Mazingi, Tom Abbott, Michel Adamina, Melika Akhbari, Ruth Benson, Shivam Bhanderi, Bruce Biccard, Edward Caruana, Sohini Chakrabortee, Reema Chapatwala, Ainhoa Costas‐Chavarri, Andreas K Demetriades, Anant Desai, Salomone Di Saverio, Thomas Drake, John Edwards, Jonathan Evans, Marco Fiore, Samuel Ford, Christina Fotopoulou, Alexander Fowler, Kaori Futaba, Ian Ganly, Harelimana Grace James, Ewen Griffiths, Alessandro Gronchi, Peter Hutchinson, Gabriella Yael Hyman, Joseph Incorvia, Ritu Jain, Michael Jenkinson, Tabassum Khan, Stephen Richard Knight, Angelos Kolias, Søren Kudsk‐Iversen, Tsun Yu Kwan, Elaine Leung, Julio Mayol, Siobhan McKay, John G. Meara, Emily Mills, Susan Moug, Akshay Patel, Roberto Perinotti, Henry E. Rice, Keith Roberts, Andrew Schache, Richard Shaw, Neil Smart, Matthew Stephens, Grant D. Stewart, Ella Teasdale, Peter Vaughan‐Shaw, Raghavan Vidya, Naomi Wright, Funmilola Wuraola, Natalie Zimmelman, Ervis Agastra, Dariel Thereska, Irida Dajti, Sergio Martin Lucchini, Veronica Laudani, Luis Boccalatte, Carina Chwat, Ivana Ines Pedraza Salazar, Diana Alejandra Pantoja Pachajoa, Sergio Martin Lucchini, Agustin Duro, José Alfredo Calderón Arancibia, Daniel Cox, Giuliana D’Aulerio, Nagendra Dudi‐Venkata, Natasha Egoroff, Shebani Farik, Natalie Lott, Jana‐Lee Moss, Sarah Rennie, Lorwai Tan, Chris Varghese, Uyen Giao Vo, David Watson, David Watters, Deborah Wright, Tim Bright, Paul Hollington, Xuanyu Zhou, Hidde M Kroon, Anthony Farfus, John Barker, Eleanor Watson, Sean Stevens, Haider Latif, Amanda Caroline Dawson, Alwin Chuan, Vijayaragavan Muralidharan, Enoch Wong, Travis Ackermann, Maurizio Pacilli, Russell Hodgson, Alexander Heriot, Peter Choong, Wendy Brown, Surjit Lidder, Justin Yeung, Luke Traeger, Guillermo Regalo, Ralph Gourlay, Peter Pockney, Peter Pockney, Sarit Badiani, Cherry Koh, Soni Putnis, Amanda Caroline Dawson, Fayza Haider, Ashrarur Rahman Mitul, Niels Komen, Bert Dhondt, Serge Cappeliez, Manon Pigeolet, Gabrielle van Ramshorst, Martijn Schoneveld, Jasper Stijns, Wouter Oosterlinck, Nicolas Flamey, Cyrille Kpangon, Mouhamed Agbadebo, S mèvo Romaric Tobome, Ismaïl Lawani, Anis Cerovac, Aldo Vieira Barros, Samuel Aguiar Júnior, Glauco Baiocchi, Heloisa Galvão do Amaral Campos, Jefferson Gross, Felipe José Fernandez Coimbra, Luiz Paulo Kowalski, Fabiana Makdissi, Suely Nakagawa, Joao Pedreira Duprat Neto, Jose Guilherme Vartanian, Guilherme Yazbek, Stenio C Zequi, Ronald Flumignan, Mihail Slavchev, Natalia Jaworska, Angela Dell, Harsha Shanthanna, Janet Martin, Abdollah Behzadi, Carolyn Nessim, Michelle Mozel, Pascal St‐germain, Crispin Russell, Gary Groot, Najib Safieddine, Duminda Wijeysundera, Antoine Eskander, Sami Chadi, Shawn MacKenzie, Alana Flexman, Fernando Heredia, Maria Marta Modolo, Julio Villanueva, Sofia Waissbluth, Roberto Macchiavello, Mario I Escudero, Tyare Fuentes, Ximena Mimica, Maricarmen Olivos, Dinimo Bolivar Saenz, Lina Caicedo, Juan Pablo Alzate, Joaquin Luna, Nestor Fabian Pedraza Alonso, Camilo Ortiz Silva, Carlos J Perez Rivera, Juliana Rodriguez, Liliana Silva‐Igua, Martha Luz Torres, Lina María Trujillo, Albaro José Nieto Calvache, Julián Balanta‐Melo, Rafael Figueroa‐Casanova, Oscar‐Julián García‐Montoya, Carlos Andres Marulanda Toro, Marcela Velez Botero, Maria Clara Mendoza Arango, Eneida Diaz Martinez, Valentina Gutiérrez Perdomo, Jose Andres Calvache, Jose Andres Calvache, Emileth Montenegro, Jakov Mihanovic, Pablo Mijahil Avilés Jiménez, Nikolaos Gouvas, René Novysedlák, Julia Rodriguez‐Abreu, Dolores Mejía, Eddy Lincango‐Naranjo, Galal Abouelnagah, Sameh Shehata, Ahmed Hossam Eldin Fouad Rida, Ramy A. Hassan, Mahmoud M. Saad, Mohamed Reda Loaloa, Badr Mostafa, Mohamed Qassem, Mohamed Fahmy, Hesham Abozied, Ahmed Y Azzam, Sherief Ghozy, Asser Sallam, Ahmed Shehta, Sameh Emile, Mohamed Abdelkhalek, Rehab Samaka, Amr Morsy, Ahmed Elshawadfy Sherif, Danilo Alfonso Arévalo Sandoval, Abraham Negussie, Tigist Fisseha, Kibruyisfaw Shumbash, Metasebia Abebe, Samuel Hailu, Seid Mohammed Yasin, Yemisirach Bizuneh Akililu, Abebe Megersa, Teshome Tefera, Melatework Assefa, Bahru Atnafu, Bereket Tsegaye, Yoseph Solomon Bezabih, Silamlak Sisay, Kebebe Bekele, Moa Jira, Mengistu G Mengesha, Habtamu Derilo, Eyueal Degefa, Anteneh Tadesse, Melkamu Nidaw, Elise Sarjanoja, Joonas H Kauppila, Sylvie Testelin, Sophie Boucher, Lionel Jouffret, Zaher Lakkis, Alban Zarzavadjian Le Bian, Luke Harper, Marc Danguy des Déserts, Benoît André, Karem Slim, Romain Verhaeghe, Andrea Police, Edouard Girard, Alexandre Chebaro, Armande Subayi Nkembi, Laurent Arnalsteen, Quentin Ballouhey, Diane Mege, Clement Jeandel, Emilie Duchalais, Pierre‐Alban Bouche, Gilles Manceau, Célia Crétolle, Erik Hervieux, Noémie Girard, Agathe Seguin‐Givelet, Sebastien Gaujoux, Belinda De Simone, Matthieu Boisson, Damien Bergeat, Alexis P Arnaud, Fabien Fredon, Francesco Nappi, Radwan Kassir, Aurélien Scalabre, Federico Migliorelli, Romain Verhaeghe, Anne‐Cecile Ezanno, Barbara Seeliger, Charlotte Vaysse, Helene Charbonneau, Vincent Misrai, Olivier Abbo, Martina Aida Angeles, Laurent Brunaud, Zaza Demetrashvili, Ali Modabber, Sebastian Wolf, Carsten Kamphues, Philipp Höhn, Tim R. Glowka, Alexander Christopher Rokohl, Ulrich Bork, Georg Fluegen, Raymund E. Horch, Andrea Schmedding, Andreas Schnitzbauer, Helge Eberbach, Daniel Schlager, Fritz Spelsberg, Lena Keppler, Andreas Hecker, Susanne Wolfer, Ulrich Ronellenfitsch, Christine Nitschke, Christian Peiper, Ibrahim Hakami, Stefan Welter, Karine Nikolaieva, Andreas Roth, Judith Lindert, Konstantinos Gousias, Anke Rissmann, Valerie Catherine Linz, Nuh Rahbari, Marie‐Claire Rassweiler‐Seyfried, Anna Eleonora Gut, Jens Gempt, Daniel Reim, Arthur Wagner, Markus Albertsmeier, Alexander M. Keppler, Mircea Gabriel Stoleriu, Tim Saier, Josef Stadler, Julia Christina Kaiser, Stefan M. Brunner, Karin Pfister, Jonas Herzberg, Kai Nowak, Tobias Reinhard, Gregor A. Stavrou, Alfred Königsrainer, Christian Konrads, Markus Quante, Simon Laban, Silke von Pusch, Markus Hirschburger, Johannes Doerner, Armin Wiegering, Ekaterini Christina Tampaki, Alitza Gutiérrez Ruiz, Alejandra Rodas, Ana Lucía Portilla, Gustavo Recinos, Maria‐Lorena Aguilera‐Arevalo, Jacqueline Carrera, Amalia Barrios Duarte, Megan Lowey, Sabrina Barillas, Atul Suroy, Dhaivat Vaishnav, Raghunandan Gorantlu Chowdappa, Irappa Madabhavi, Dhananjaya Bhat, Sunil Kumar Venkatappa, Sumit Thakar, Kavitha Jain, Aruna Kumar, Manoj Nagar, Tushar Mishra, Arunkumar Sekar, Anand Gupta, Lileswar Kaman, Madhivanan Karthigeyan, Manjul Tripathi, Ashwin Rammohan, Sudheer Othiyil Vayoth, Anupama Rajanbabu, Anbukkani Subbian, Rahul Gupta, Monish Raut, Nissi Evelyn, R Lavanya Kannaiyan, Anil Matai, Sanjeev Misra, Vishal Bhende, Sathish Muthu, Indranil Ghosh, Abhishek Sharma, Ankur Bajaj, Shiv Rajan, Gaurav Agarwal, Pranay Pawar, Philip Alexander, M Vijayakumar Vijayakumar, Zeeshan Hameed, L Badareesh, Navneet Kumar Chaudhry, Lipika Baliarsing, Satish Dharap, Amruta Kulkarni, Yuvaraja Thyavihally, Rahul Deo Sharma, C S Pramesh, Rajesh Soni, Surya Kumar Dube, Shilpa Sharma, Harvinder Singh, Lovenish Bains, Rahul Ghodke, Ashwani Kumar, Vivek Sodhai, Suvendu Maji, Somprakas Basu, Chandrashekhar Mahakalkar, Ravi Kannan, Asif Mehraj, N Ranganath, Ashish Phadnis, I Yadev, Alfie Kavalakat, Rohin Mittal, Karthik Chandra Vallam, Hamed Akhavizadegan, Esmaeil Rezghi Maleki, Naser Yousefzadeh Kandevani, Hilary Ikele, Catherine McNestry, Christina Fleming, Stephen O’Brien, Sami Abd Elwahab, Niall Davis, Mohsen Javadpour, Brendan McDonnell, Clare O Connor, Jarlath Bolger, Cillian Clancy, Stefanie M Croghan, Noel Donlon, Carolyn Cullinane, Ben Creavin, Muheilan Muheilan, Helen Earley, Syed Mohammad Umar Kabir, Muhammad Fahadullah, éanna Ryan, Tara Connelly, Oded Zmora, Daisuke Hashimoto, Majdi Ali Alqudah, Amer Alajalen, Rand Y. Omari, Faris Ayasra, Abdulrahman Qasem, Yazan Alawneh, Amer Ahmad, Omar Aladawi, Bourhan Alrayes, Hanan Haidar, Shatha Husain, Faisal Qassem, Adnan Sumadi, Ala’a Abu Salhiyeh, Balqees Mahmoud Al‐Manaseer, Zaid Alsunna, Hazim Ra’ed, Faten Reyad Bani Hamad, Amro Abuleil, Mohamad K. Abou Chaar, Elmi Ahmed Mohamed Jimaale, Marah Abu‐Mehsen, Noor Olaywah, Omar Wafi, Hazim Ababneh, Luai Abu‐Ismail, Almu’atasim Khamees, Ahmad Alkhatib, Raikhan Bolatbekova, Mukhtar Kulimbet, Talgat Nurgozhin, Timur Saliev, Baurzhan Zhussupov, Ydyrys Almabayev, Ildar Fakhradiyev, Timur Saliev, Dilyara Kaidarova, Khalil Tamoos, Ahmed Aqeelah, Alsnosy Abdullah Khalefa Mohammed, Faraj Al Maadany, Ghadah Alkadeeki, Milad Gahwagi, Wafa Aldressi, Mohamed Amnaina, Arowa Hassan Abdulrahman Alansari, Akram Alkaseek, Ghozlan Yagoub, Anass Ben Amer, Marwa Salem, Ayman Almugaddami, Dania Burgan, Mohammed Abdelkabir, Khayriyah Alshareef, Rayet al Islam Ben Jouira, Ayman Meelad, Ahmad Bouhuwaish, Sumayya Essayah Dwaga, Houda Khalifa, Bushray Almiqlash, Taha Suliaman, Mohammed Alawami, Fras Elhajdawe, Hajir Aboazamazem, Ibrahim Ellojli, Ahmed Msherghi, Ismail Ali Saleh, Mohammed Alayan, April Camilla Roslani, Marcel Didier Ndayishyigikiye, Akutu Munyika, Philipp Plarre, David W Borowski, Pueya Rashid Nashidengo, Milou Noltes, Pieter Steinkamp, Cameron Wells, Rebecca Teague, Brodie Elliott, David Kieser, Omar Mohyieldin, Chris Varghese, Nick McIntosh, Cheyaanthan Haran, Sarah Rennie, Jasmin King, Jeong Ha, Matthew James McGuinness, Opeoluwa Adesanya, Julius Olaogun, Akinola Akinmade, Kefas Bwala, Peter Agbonrofo, Akinwale Afolabi, Usang Usang, Sebastian Ekenze, Samson Olori, Taiwo Akeem Lawal, Justina Seyi‐Olajide, Abiodun Okunlola, Omolara Williams, Adewale Adisa, Lukman Abdur‐Rahman, Stephen Kache, Danjuma Sale, Lofty‐John Anyanwu, Chukwuma Okereke, Musliu Adetola Tolani, Venko Filipce, Lazar Todorovic, Sofija Pejkova, Sotir Stavridis, John George Massoud, Sareyah Alsibai, Rizwan Sultan, Humera Naz Altaf, Abu Bakar Hafeez Bhatti, Shahzad Hussain Waqar, Aliya Aziz, Asad Ali Kerawala, Lajpat Rai, Mariyah Anwer, Aiman Tariq, Bushra Ayub, Sami Ullah Niazi, Muhammad Yasir Naseem, Muhammad Zeeshan Sarwar, Muhammad Imran Khokhar, Imdad Ahmad Zahid, Haroon Javaid Majid, Nabila Talat, Muhammad Asif, Muhammad Hamid Chaudhary, Umer Farooq, Siddique Ahmad, Waleed Mabood, Syed Imran Bukhari, Muhammad Tariq, Eesha Yaqoob, Saad Javed, Saad Javed, Muhammad Usman Malik, Hassan Nawaz Yaqoob, Moises Cukier, Glenda Marina Falcon Pacheco, Robinson Mas Melendez, Arazzelly Del Pilar Paucar Urbina, Jose Rios Chiuyari, Carlos Eduardo Otiniano Alvarado, Lorena Fuentes Rivera Lau, Giuliano Borda‐Luque, Milagros Niquen‐Jimenez, Claudia Arias, Sergio Zegarra, Jenner Betalleluz Pallardel, Regina Amparo Ugarte Oscco, Gian Mendiola, Yahaira Tatiana Carpio Colmenares, Carlos Shiraishi Zapata, Maria Rosa Ortiz, Marie Dione Sacdalan, Piotr Major, Filipe Castro Borges, Octavio Viveiros, Pedro Serralheiro, Paulo Santos‐Costa, Filipa Mendes, Miguel Rocha Melo, Paulo Cardoso, Ana Soares, José Azevedo, Rita Gonçalves Pereira, Nelson Silva, André Caiado, Maria Luís Sacras, Pedro Azevedo, Rui Almeida‐Reis, Miguel F. Cunha, João Oliveira, Jorge Nogueiro, Mafalda Sampaio‐Alves, Luciana Cidade Costa, Catarina Baía, Ana Cláudia Deus, Rita Branquinho, André Marçal, André Tojal, Ahmad Zarour, Ahmad Zarour, Silviu Tiberiu Makkai‐Popa, Aurel Mironescu, Florin Grama, Elena Adelina Toma, Ionut Negoi, Daniela Filipescu, Nicolae Bacalbasa, Natalia Motas, Sebastian Ionescu, Octav Ginghina, Radu Costea, Narcis Octavian Zarnescu, Radu Drasovean, Eduard‐Alexandru Bonci, Mihail‐Gabriel Dimofte, Vlad Porumb, Mikhail Kirov, Yegor Molitvin, Andrey Litvin, Vadim Pykhteev, Marianna Raevskaya, Sergey Efetov, Aleksandr Butyrskii, Mushabab Alshahrani, Azah Althumairi, Nasser Alzerwi, Ahmed Al Ameer, Ahmed Al Ameer, Tariq Madkhali, Abddulrahman Saleh Almulhim, Salman Ghazwani, Abdu Ayoub, Othman Iskander, Mohammed Ghunaim, Mohammed Alharthi, Turki M Alzaidi, Azah Althumairi, Mohammad Alyami, Abdulrahman Al Amri, Azah Althumairi, Abdullah AlFakhri, Amal Alhefdhi, Sharfuddin Chowdhury, Thamer Nouh, Ameen Alshehri, Abdulrahman Alzahrani, Yousef Alalawi, Selmy Awad, Ibrahima Konate, Abdourahmane Ndong, Jacques Tendeng, Nan Zun Teo, Frederick Koh, Jurij Aleš Košir, Uros Bele, Sabra Aqil, Cristina Barrena López, Ana Sánchez Mozo, Antonio Rodriguez Infante, Patricia Caja Vivancos, Mikel Prieto, Igor Alberdi San Roman, Laura Gomez Fernandez, Josep Maria Muñoz Vives, Anna Carreras‐Castañer, Berta Díaz‐Feijoo, Ramon Sieira‐Gil, Victor Turrado‐Rodriguez, Anna Sánchez López, Santiago Sánchez‐Cabús, Marta Jimenez Toscano, MªPilar Canals Sin, Saura García Laura, Oriol Martin Sole, Pedro Palazon Bellver, Sonia Pérez‐Bertólez, Jordi Prat‐Ortells, Mireia Riba Martínez, Josep Rubio‐Palau, Xavier Tarrado, Jorge Nuñez, Veronica Alonso Mendoza, Coro Bescós, Eloy Espin‐Basany, Martin Espinosa‐Bravo, Daniel Gil‐Sala, Susana González‐Suárez, Nuria Montferrer Estruch, Jorge Nuñez, Lucia Porteiro Mariño, Ana Rodríguez‐Tesouro, Fabian Rojas Portilla, M Pilar Tormos Pérez, Inmaculada Vives, Unai Garcia De Cortazar, Kiara Tudela, Aitor Landaluce‐Olavarria, Mercedes Estaire Gómez, Jorge Almoguera, Bakarne Ugarte‐Sierra, Virginia Jimenez, Marta Bertrand, Laura Cárdenas Puiggrós, Olga Delisau‐Puig, Jorge Garcia‐Adam ez, David Julià Bergkvist, Eloy Maldonado‐Marcos, Lucia Diego García, Marta Roldón Golet, Iván Soto‐Darias, Aida Cristina Rahy‐Martín, Diego Enjuto, Adolfo Ramos‐Luengo, Juan Delgado Fernandez, Carolina Lugo Duarte, Cristina Ojeda Thies, Lucila Marquez, Diana Crego Vita, Jana Dziakova, Ana Maria Minaya Bravo, Jorge Caño Velasco, Olga Mateo‐Sierra, Begoña Quintana‐Villamandos, Cristina Rey Valcarcel, Javier Rio, Laura Román García de León, Marcello Di Martino, Jorge Prada, Javier Serrano González, Manuel Losada, Jose Tomas Castell Gomez, Ramon Corripio‐Sanchez, Alexander Forero‐Torres, José Manuel Morales‐Puebla, Hanna Perez‐Chrzanowska, Santiago Valderrabano Gonzalez, Alvaro Yebes, Ignacio Zapardiel, Manuel Diez Alonso, Nelson Morales Palacios, Alberto Cabañero Sánchez, Fátima Sánchez Fernández, Alfredo Abad Gurumeta, Ane Abad‐Motos, Fernando Corella, Javier Ripollés‐Melchor, Rosa Sanz‐Gonzalez, Marta Alcaraz Fuentes, Maria Teresa Fernández Martín, Pablo Calvo Espino, Milagros Carrasco Prats, Antonio‐José Fernández‐López, Damián García Escudero, Vanesa Garcia Soria, Jesús Aarón Martínez Alonso, Miguel Ruiz‐Marín, Beatriz Gómez Pérez, Joaquin Moya‐Angeler, Daniel Fernández Martínez, Heura Llaquet Bayo, Enrique Colás‐Ruiz, Susana Bella Romera, M. Teresa Gavaldà Pellicé, Misericòrdia Jordà Solé, Enrique Jose Ruiz Velasquez, Bernardo Núñez, Raul Jimenez, Jon Zabaleta, Maria Jose González‐Gimeno, Irene Ortega Vázquez, Antonio Perez Ferrer, Rubén Martín‐Láez, Marcelo Moreno Suarez, Miguel Angel Freiria Eiras, Irene Ramallo‐Solís, Juan‐Carlos Gomez‐Rosado, Jose Ramon Oliver Guillen, Mar Achalandabaso Boira, Juan Carlos Catalá Bauset, Julio Domenech, Rafael Badenes, Juan Carlos Bernal‐Sprekelsen, Jorge Sancho‐Muriel, Beatriz De Andrés‐Asenjo, Francisco J Tejero‐Pintor, Marc Vallve‐Bernal, Alba Vazquez Melero, Laura Sánchez Blasco, Jorge Escartin, Victoria Duque Mallén, Selvaratnam Srishankar, Umesh Jayarajah, Charitha Sooriyabandara, Oshan Basnayake, Nalaka Gunawansa, Dakshitha Wickramasinghe, Prabuth Dulanjan Weeraddana, Thanusan Vimalakanthan, Shanthamoorthy Gishanthan, Pramodh Chandrasinghe, Eleftherios Gialamas, Marc‐Olivier Sauvain, Ahmad Ghazal, Yusra Al‐Sabbagh, Turki Alhassoun, Sara Maa Albared, Antoine Naem, Hareth Alnahr, Ghassan Jisry, Ali Hammed, Arda Isik, Okedi Francis Xaviour, Gaston Turinawe, Isaac Mubezi, Franck K. Sikakulya, Andrew Kakeeto, Wilberforce M. Kabweru, Hervé Monka Lekuya, Ronald Kiweewa, Herman Lule, Paul Matovu, Otolia Isaac, Mohamed Mashhour, Amin El Helw, Sattar Alshryda, Safeena Kherani, Awadelkarim Mohamed, Ferial Mohamed Ali Abbas, Diary Mohammed, Ehab Aldlyami, Rakesh Kundra, Mohamed Mashhour, Mohamed Mashhour, Antony Louis Rex Michael, Hayder Alsaadi, Kareem S. Khalil, Rachel Dbeis, Shafaque Shaikh, Jenny Ferry, Aiman Jamal, Haleema Siddique, Rishi Das, Nikhil Ponugoti, Sivesh K Kamarajah, Pornjittra Rattanasirivilai, Asma Sultana, Frances Mosley, Matthew Chan, Antony Bateman, Gareth Davies‐Jones, Fanourios Georgiades, Grant D. Stewart, Navid Ahmadi, Aman Coonar, Mariam Baig, Chetan Khatri, Arthika Surendran, Julian Sonksen, Robert Sinnerton, Caitlin Brennan, Gemma Faulkner, Michael Greenhalgh, Hannah Emerson, Kiran Singisetti, Joshua Totty, Michael Wilson, Terence Lo, Harriet Corbett, Ijeoma Okonkwo, Gill Arbane, Kariem El‐Boghdadly, Cyrus Kerawala, Chetan Parmar, Tom Abbott, Michael Bath, Funlayo Odejinmi, Jayesh Sagar, Rishi Talwar, Samuel Newman, John Hammond, John Moir, Natalie Duric, Tamas Szakmany, Ahmar Iftikhar Talib, Mina Youssef, Christopher Lewis‐Lloyd, Christopher Lewis‐Lloyd, Mariam Lami, Khurram Ayub, Benjamin Dean, Supriya Balasubramanya, Sathya Lakpriya, Luke Rogers, Paul Turner, Mark Maher, Kohila Sigamoney, John Edwards, Jihène El Kafsi, John Hardie, David Johnson, Christin Henein, Marianne Hollyman, Ketan Agarwal, Simon Powell, Govind Singh Chauhan, Rakesh Patel, Joel Gagnier, Heather Carmichael, Aurora, Kristofor A. Olson, Eric Etchill, Joseph Incorvia, Sameer Hirji, Matthew Naunheim, Frederick Drake, Haytham Kaafarani, Caroline Reinke, Anna Alecci, Dennis Vaysburg, Jennifer Rodriquez, Emily Shih, Vin Shen Ban, Julia Coleman, Henry E. Rice, Krista Kaups‐Fresno, Emmanouil Giorgakis, Maggie DiNome, Neal Bhutiani, Omar Alnachoukati, Brittany Bankhead‐Kendall, Taylor Aiken, Thomas Diehl, Ankush Gosain, Rishi Rattan, Muhammad Owais Abdul Ghani, Nensi Melissa Ruzgar, Anna Liveris, Nina Glass, Charu Paranjape, Theresa Chin, Antonio Meola, Kristina Nicholson, John Squiers, Stephanie Lueckel, Janani Reisenauer, Rachael Callcut, Ahmed Mansour, Allison Berndtson, Lucy Kornblith, Sara Seegert, Paulo Martins, Hamza Al‐Naggar, Mohammed Al‐Shehari, Ibrahim Al‐Raimi, Allan Ngulube, Maphios Siamuchembu, Willard Mushiwokufa, Busisiwe Mlambo, Simbarashe Chinyowa, Ervis Agastra, Enton Bollano, Kostandin Gjyli, Dariel Thereska, Irida Dajti, Jola Kerpaci, Enxhi Vrapi, Lorena Zijaj, Bahraoui Djahida, Belabbes Fatima Zohra, Kouidri Khadidja, Oussama Bali, Nassim Benallel, Ismahene Lalmi, Meriem Abdoun, Nesrine Aouabed, Souad Bouaoud, Kamel Bouchenak, Assia Haif, Zineddine Soualili, Joaquin Bastet, Agustín Bianco, Daniel Capitaine, Jorge Centeno Lozada, Carlos Esquivel, Rodrigo Figueroa, Manuel Garcia, Pablo Martín García, José Ignacio Gerchunoff, Fernando Martinez Iascano, Jose Mondino, María Emilia Muriel, Carmignani Pablo, Martin Passadore, Alejandra Tornini, Rogelio Traverso, Lara Vargas, Gerardo Zanoni, Norberto Berber, Estefanía Cotta, Marcela Di Vincenzo, Esteban Sebastian Gallino, Cecilia Gigena, Bianca Grassano, Veronica Laudani, Ignacio Lugones, Constanza Madrid, Maximiliano Maricic, Antonio Alberto Martinez, Andres Rosso, Pablo Scher, Sofia Tachella, Damaris Idara Anabel Zezular, Carla Abuawad, Agustin Albani Forneris, Carola Allemand, Laura Gisela Alvarez Calzaretta, Luis Boccalatte, Jorge G. Boretto, Rocio Boudou, Rodrigo Brandariz, Martin Buljubasich, Arturo Burchakchi, Martin Buttaro, Juan Pablo Campana, Virginia Cano Busnelli, Tomas Carminatti, Agustina Florencia Castro Lalín, Julian Cereghini, María Sol Crespi Amor, Roberto Sebastián Croattini, Maria Sol Fernandez, Marcelo Figari, Uriel Fraidenraij, Diego Gallegos, Agustín Maria García‐Mansilla, Marcos Gonzalez, Matias Ignacio Gonzalez, Esteban Gonzalez Salazar, Jeremias Goransky, Guillermo Hernandez Gauna, Felipe Higuera, Fernando Holc, Esteban Gabriel Jauregui, Juan Larrañaga, Juan Liyo, Lionel Llano, Pablo Lobos, Emilia Luzzi, Gustavo Mastroianni, Santiago Miguel Mata‐Suarez, Horacio F. Mayer, Santiago Mc Loughlin, Efrain Mendoza, Ricardo Esteban Mentz, Pedro Mercado, Pablo Moyano, Florencia Noll, Diego Odetto, Agustina Rene Oliva, Rafael Perez Vidal, Catalina Poggi, Eduardo Jorge Premoli, María Lourdes Ramos, Patricio Rosas, Luciano Rossi, Gustavo Rossi, Jose Saadi, Jordán Scherñuk, Pablo Slullitel, María Verona Stang, María Victoria Taboada, Sebastian Tirapegui, Constanza Uffelmann, Carlos Vaccaro, Roberto Vagni, Ana Clara Valerio, Celeste Soledad Zarratea, Carina Chwat, Silvina Montal, Brian Morris, Pedro Valdez, Fernando Diaz‐Couselo, Ivana Ines Pedraza Salazar, Luciana Sabatini, Fernando Andres Alvarez, Micaela Avila, Nicole Benitez Benitez, Nicolás Bruera, Marcelo Doniquian, Manuel Gielis, Julian Liaño, Florencia Llahi, Facundo Mandojana, Walter Páez, Diana Alejandra Pantoja Pachajoa, Matias Parodi, Héctor Picon Molina, Agustin Pinsak, German Viscido, Roberto Badra, Diego Belisle, Hernan Borla, Georgina Eberle, Agustin Esteban, Carlos Ignacio Ferrero, Micaela Furlan, José Sebastian García, Lucas Granero, Mariel Henzenn, Rodrigo Juaneda, Sergio Martin Lucchini, Esteban Politi Vidal, Guillermo Romero Reyna, José Gabriel Yaryura Montero, Maria Mercedes Caubet, José Luis D’Addino, Agustin Duro, José Alfredo Calderón Arancibia, Deepu Daryanani, Martijn Gosselink, Alex Ponson, Mohamed Afzal, Mathew Amprayil, Mark Brooke‐Smith, John Chen, James Grantham, Benjamin Gricks, Amanda Hii, Nikhil Kundu, David Liu, Charles Livingston, Matthew Marshall‐Webb, Christina McVeay, Hamish Moore, Gavin Nair, Bee Shan Ong, Dominic Parker, Victoria Rudolph‐Stringer, Malgorzata Szpytma, Ravi Vissapragada, Melissa Yun Wee, Geoffrey Yuet Mun Wong, Xuanyu Zhou, Dylan Richard Barnett, Melissa Bochner, John Bolt, Karel Buddingh, Brendon Coventry, Sean Davis, Joseph Dawson, Stuart Denham, Christopher Dobbins, Nagendra Dudi‐Venkata, Robert Fitridge, Siang Wei Gan, Izhar‐Ul Haque, Matheesha Herath, Shivangi Jog, Harsh Kanhere, Hidde M Kroon, Beatrice Kuang, Yick Ho Lam, Virginia Lambert, Alicia Lim, Grace Maina, Cea‐Cea Moller, Eu Nice Neo, Eu Ling Neo, Alain Nguyen, Shalvin Prasad, Jennifer Roy, Christine Russell, Tarik Sammour, Nicholas Smith, Richard Smith, Conrad Stranz, Saam Tourani, Steven Tran, Lucinda Van de Ven, Leigh Warren, Robert Whitfield, Jamie Wormald, Yijie Yin, Christopher Bierton, Benjamin Cribb, Anthony Farfus, Katarina Foley, Daniel Ong, Matthew Watson, John Barker, Jacob Hampton, James Karam, Anya Rugendyke, Tyson Zhang, Carolyn Vasey, Eleanor Watson, Eduardo Apellaniz, Adam Frankel, Lara Gahan, Glen R Guerra, Amanda Liesegang, Nicholas Lutton, Andrew Maurice, Scott Miron, Aveechal Prasad, Ashleigh Sercombe, Thanusan Sivapalan, Syed Danial Syed Ahmad, Chin Li Tee, Lachlan Yaksich, Martin Batstone, Omar Breik, Thomas Young, Michael Field, Benjamin Scott, Sean Stevens, Gabriella Charlton, Si Chen, Henry Yan Chi Cheung, Neha Gauri, Ross Hayhurst, Sophie James, Seyoung Jang, Fangzhi Jia, Helen Zhang, Randeep Aujla, Michael Finsterwald, Digby Percy, Alvin Tanaya, Anand Trivedi, Uyen Giao Vo, Andrew Aylett, Richard Grills, Angela Holmes, Matthew Ming Kei Kwok, Anton Lambers, Leo Lambers, Haider Latif, Evania Lok, Sonal Nagra, Richard Page, Nicholas Paltoglou, Vishwakar Panuganti, Sophie Riddell, Gareth Rudock, Hannah Tan, Kirk Underwood, David Watters, Daniel Youssef, Ricci Amoils, Nazli Bahtigur, Savitha Bengeri, Lily Builth‐Snoad, Hock Ping Cheah, Paul Chen, Kenneth Chew, Alyssa Chong, Sara Clark, Rhea Darbari Kaul, Amanda Caroline Dawson, Eliya Devan, Christina Ewington, Aleeza Fatima, Sophia Fitt, John Gaul, Paloma Ghosal, Amy Gojnich, Indu Gunawardena, Peter Hamer, Samuel Holmes, Zhen Hou, Danielle Jolly, James Dimitri Kane, Micayla Kaufman, Tanishq Khandelwal, Sukhwant Khanijaun, Kelvin Kwok, Edward Latif, Sharon Laura, Vivian Lee, Catherine Leung, Ina Liang, Peter Lin, Elizabeth Weng Yan Lun, Haili Luo, Victor Ly, Jolande Ma, Brooke Macnab, Richard McGee, Stephanie Miles, William Munro, Harry Narroway, Tin Yau Ngan, Anthony Noor, Upuli Pahalawatta, Melissa Park, Cameron Parkin, Rita Poon, Shanmugam S Somasundaram, Claudia Saab, Renwick Simpson, Dana Steinel, Emily Taylor, Adrian Tchen, Shu Thong, Kelvin Tran, Kevin Tree, Lucille Vance, Ken Wong, Victor Yu, Michael Zhang, Aram Cox, David Chi Hau Tan, Nova Thani, Shefali Das, Taigh Macdonald, Paul Salama, Gagandeep Sandhu, Rachel Shadbolt, Du Phan, David Townend, Alwin Chuan, Eunmaro Ju, Sang Mi Lee, Stephen Barnett, Daniel Cox, Hajar Hasan Kheslat, Andrew Higgins, Chloe Jamieson‐Grigg, Victoria Jenkins, Brett Larner, Vijayaragavan Muralidharan, Marcos Perini, David Proud, Kirby Qin, Georgina Riddiough, Sivendran Seevanayagam, Damien M. Wu, Chris Zhao, Sonali Aggarwal, Vinna An, Melanie Battershell, Elliot Chan, King Tung Cheung, Anna Drake, Janindu Goonawardena, Benjamin Hunt, Christopher Ip, Vikram Iyer, Anshini Jain, Joshua Kealey, Chen Lew, Christopher Seng Hong Lim, Tara Luck, Sean Mackay, Natalie Maher, Georgia Maroske, Nicholas Roubos, Shomik Sengupta, Christopher Steen, Zac Tsigaras, Zachary Tuttle, Salena Ward, Marli Williams, Enoch Wong, Travis Ackermann, Evie Yeap, Jessie Zhou, Amy Coates, Nora Mutalima, Ton Tran, Ramesh Nataraja, Maurizio Pacilli, Claire Sharpin, David Bird, Kay Tai Choy, Sarah Condron, Jurstine Daruwalla, Isabela dos Anjos, Hanna El‐Khoury, Robert Fabian, Andrew Gillard, Rebecca Greenop, Russell Hodgson, Michael Issa, Sharon Lee, Krinal Mori, Nikki Petrakis, Maryum Qureshi, Danielle Sabella, Prassannah Satasivam, Niranjan Sathianathen, Sean Ezekiel Seow, Amanda Shen, Margaret Shi, Meher Tabassum, Rodrigo Teixeira, Josephine Vivian‐Taylor, Jennifer Wheatley, Alexander Heriot, Sally Shepherd, Mikael Soucisse, Sebastian King, Brendan O’Connor, Warwick Teague, Simon Banting, Lynn Chong, Peter Choong, Sharnel Clatworthy, Angela Cochrane, Adrian Fox, Michael Wei Hii, Anna Isaacs, Mary Ann Johnson, Brett Knowles, Andrew Newcomb, Veronique Price, Jaishankar Raman, Matthew Read, Alistair Rowcroft, Lillian Taylor, Salena Ward, Gavin Wright, Wendy Brown, Prem Chana, Kalai Shaw, Jacob Bock, Jordan Cory, Katharine Drummond, Jack Lahy, Surjit Lidder, Thomas McIntire, Benjamin Price, Claire Stark, Alex Besson, Richard Gartrell, Brianne Lauritz, Ha My Ngoc Nguyen, Chui Foong Ong, Meron Pitcher, Lorna Scullion, Howard Tang, Danielle Taylor, Brigid Wolf, Justin Yeung, Luke Traeger, Matthew Watson, Matthias Wichmann, Amelia Davis, Amelie Maurel, Kyle Raubenheimer, Cassidy Campbell, Ashley Colaco, Maria Julia Corbetta Machado, Abbie Heffernan, Costa Karihaloo, Isabella Ludbrook, Sean SW Park, Bibi Nabeeha Peerally, Guillermo Regalo, Camila Singhai, Heidi Stevens, Sergey Vavilov, Anne‐Marie Aubin, Ralph Gourlay, Margaret Harris, Zhi Ying Lim, Rebecca Spring, Melissa Stieler, Nicholas Adamson Barnes, Ahmad Seraj Alam, Ali Alsoudani, Francesco Amico, Rebecca Anning, Zsolt J. Balogh, Alison Blatt, Scott Cairns, Jesse Carroll, Andrew Caterson, Adam Christie, Daron Cope, Tyson Dale, Grace Dennis, Anchal Duggal, Brendan Ennis, Mark Fenton, Yi Xin Joanna Fu, Ana Galevska‐Dimitrovska, Jonathan Gani, Scott Gelzinnis, Madelyn Gramlick, Claudia Hadlow, Ruth Hardstaff, Munish Heer, Merran Holmes, Bridget Hone, Sophie Hu, Linna Huang, Ramiz Iqbal, Niall Jefferson, Dimithi Kasthurirathne, Stephen Kuo, Wayne Yan Lau, Hsin‐Ping Liang, Daniel Lim, Rosalina Lin, Jack McDonogh, Elysse Mcilwain, Yan Joyce Ming, Christine O’Neill, Ryan James Ocsan, Benjamin Oosthuizen, Nicole Organ, James Otieno, Felicity Park, Amanda Paterson, Marisol Perez Cerdeira, Luke Peters, Josefin Petersson, Peter Pockney, Sonia Rubbo, Venesa Siribaddana, Kabilan Thurairajah, Caleb Ting, Antonia Watson, Teagan Way, Elvina Wiadji, Ellisha Willoughby, Ashley Bailey, Samuel Broadbent, Viswanathan Narayanan, Samuel Stephens, Anna Wilkes, Jie Zhao, Mohammed Ballal, Carl D’Souza, Clara Forbes, Matthew Goss, Monique Haddleton, Jacinda Harty, Dickon Hayne, Nicole Hew, Wee Ling Koh, Shawn Lee, Shahbaz S Malik, Naseer Mohammed Abdul, Jana‐Lee Moss, Toby Richards, Yi Th Ng Seow, Anand Trivedi, Uyen Giao Vo, James Wong, Leigh Archer, Nur Sabrina Binti Babe Azaman, Tina Dilevska, Jonathan Foo, Tasvinder Hans, Shirley Jansen, Mohit Kumar, Christopher Leeson, Terence Pham, Fernando Picazo Pineda, Saravanan Rajakumar, Supisara Suk‐Udom, Jonathan Tan, Benjamin Thurston, Bichen Zhao, Shivangi Gupta, Lachlan Hou, Nivedan Jeyamanoharan, James Leigh, Wei Shearn Poh, Michael Sala, Rebecca Crothers, Madhulika Dravid, Ben Harrison, Sumayya Islam, Conor McCartney, Henco Nel, Natasha Pearson, Christos Apostolou, Sarit Badiani, Christophe Berney, Richard Chou, Sam Hanna, Andrew Lam, Vanessa Ma, Salonee Shubhen Phanse, Michael Elliott, Daniel Phung, Jessica Barry, Eleanore Clark‐Mackay, Jennifer Cope, Richard Halliwell, Ji Chen, Marie Shella De Robles, Alyssa Llorando, Humaira Haider Mahin, Soni Putnis, Dominic Rao, Faisal Syed, Daniel Abulafia, Benjamin Buckland, Timothy Cordingley, Ashe DeBiasio, Andrew Drane, Patrick Ireland, Danielle Jolly, Benji Julien, Lan‐Hoa Le, Austin Yeon Suk Lee, Eu Jhin Loh, Andrew Middleton, Brienna Mortimer, Samuel Sebastian, Brooke Short, Peter Stewart, Stephanie van Ruyven, Daniel Wong, Charry Zhang, Bonnie Zhu, William Ziaziaris, Aran Leitner, Lukas Tolzman, Matthias Zitt, Gergely Rakos, György Székely, Gabriel Djedovic, Ingmar Königsrainer, Felix Aigner, Caterina Allmer, Barbara Herritsch, Martin Mitteregger, Christian Schauer, Gerald Seitinger, Carmen Siebenhofer, Stefan Uranitsch, David Duller, Erwin Mathew, Christoph Skias, Alexandros Andrianakis, Armin Belarmino, Petra Brinskelle, Christoph Castellani, Tina Cohnert, Melanie Fediuk, Andrea Fink, Clemens Holzmeister, Judith Kahn, Josip Kresic, Andreas Leithner, Joerg Lindenmann, David Lumenta, Birgit Michelitsch, Saulius Mikalauskas, Sebastian P. Nischwitz, Paul Puchwein, Andrej Roj, Peter Schemmer, Georg Singer, Freyja‐Maria Smolle‐Juettner, Holger Till, Axel Wolf, Tibor Oliver Andraschofsky, Rafael Angerer, Daniel Arco, Claudia Kaufmann, Nura Kilic, Peter Widschwendter, Reinhard Angermann, Marlies Bauer, Nicole Bergmann, Christian Freyschlag, Martin Gisinger, Can Gollmann‐Tepeköylü, Michael Graber, Alexander Haim, Carina Harasser, Bettina Härter, Jakob Hirsch, Markus Hofer, Johannes Holfeld, Anna Lena Huber, Martina Kralinger, Dietmar Krappinger, Irmgard Elisabeth Kronberger, Marlene Kuen, Michael Liebensteiner, Franka Messner, Alex Messner, Felix Naegele, Marijana Ninkovic, Yvonne Nowosielski, Antonia Osl, Cornelia Ower, Leo Pölzl, Teresa Rauchegger, Daniel Reimer, Johannes Riecke, Anton H. Schwabegger, Filipp Sokolovski, Anna Strimmer, Markus Süss, Martin Thaler, Julian Umlauft, Claus Zehetner, Alain G. Zeimet, Jakob Allerstorfer, David Haslhofer, Daniel Hofer, Matthias Luger, Lorenz Pisecky, Nikolaus Poier, Nina Rubicz, Christoph Schmolmüller, Paul Zwittag, Tobias Rossmann, Francisco Ruiz‐Navarro, Harald Stefanits, Hend Elsayed, Peter Habertheuer, Tereza Hajkova, Wolfgang Loidl, Ferdinand Luger, Amadeus Windischbauer, Ines Fischer, Reinhold Függer, Patrick Kirchweger, Thomas Saini, Julian Berger, Wolfgang Fraz, Arastoo Nia, Mihaly Kenez, Margit Nichita, Astrid Magele, Thomas Mayr, Chiara Noe, Reinhard Bittner, Kurosch Borhanian, Isabella Dornauer, Klaus Emmanuel, Ana Gabersek, Antonia Gantschnigg, Michael Grechenig, Ricarda Gruber, Jörg Hutter, Tarkan Jäger, Oliver Koch, Michael Lechner, Lisa Manzenreiter, Franz Mayer, Iris Muehlbacher, Jaroslav Presl, Daniel Rezaie, Philipp Schredl, Martin Varga, Michael Weitzendorfer, Angela Wimmer, Eberhard Brunner, Michael de Cillia, Michaela Gruber, Martin Grünbart, Elmar Heinrich, Hannes Hoi, Vanessa Kemmetinger, Christof Mittermair, Victoria Mosshammer, Christian Obrist, Peter Paal, Judith Roesch, Elisabeth Russe, Tobias Schaetz, Jan Schirnhofer, Karl Schwaiger, Gottfried Wechselberger, Helmut Weiss, Maximilian Lanner, Alf‐Dorian Binder, Thomas Gürtler, Marcus Fink, Daniel Reichhold, Radoslava Stoyanova, Klara Beitl, Martin H. Bernardi, Philip Datler, Christopher Dawoud, Alex Farr, Philipp Foessleitner, Christoph Grimm, Felix Harpain, Mir Alireza Hoda, Simone Holawe, Marlene Kranawetter, Johannes Ott, Alexandra Perricos, Maximilian Pesta, Robert Pillerstorff, Christian Reiterer, Stefan Riss, Klara Rosta, Georg Scheriau, Edda Tschernko, Rene Wenzl, Dominik Wiedemann, Peter Wohlrab, Bernhard Zapletal, Matthias Zimmermann, Daniel Zimpfer, Oliver Findl, Andreea Fisus, Manuel Ruiss, Melanie Komaz, Nikolaus Meindl, Florian Primavesi, Karl Heinz Stadlbauer, Stefan Stättner, Florian Steiner, Ondrej Cerny, Eva Falkensammer, Hans Knotzer, Paul Köglberger, Bernhard Poidinger, Clemens Georg Wiesinger, Johannes Burtscher, Sebastian Rath, Felipe Trivik‐Barrientos, Gunay Aliyeva, Vuqar Behbudov, Gurbankhan Muslumov, Natiq Zeynalov, Mahmood Alam, Fatema Alfayez, Fayza Haider, Batool Hasan, Maryam Mahdi, Husain Mulla, Kawthar Qader, Amr Saeed, Ahmed Shirazi, Aysha Albastaki, Nuha Birido, Hiba Hameed Chagla, Abdulla Dawaishan, Baheya Dawaishan, Abeer Farhan, Isam Juma, Asher Khan, Aqsa Mohammad Eqbal Patel, Madhu Srinivasan, Fatema Waheed Akbar Mohamad Akbar Nawab Deen, Seemal AbdulQadir, Abdulla Jabr, Fauzia Maqsood, Noora Almoosa, Mai Naseer, Mohammed Shadrul Alam, AKM Khairul Basher, Shahnoor Islam, S.M. Nazmul Islam, Sabbir Karim, Muntasir Faisel, Sadia Khan, Iftekhar Ibne Mannan, Nawshin Nazia, Malissa Bentham, Sasha Corbin, Alex Doyle, Asha Eastmond, Amelia Haynes, Margaret O’Shea, Greg Padmore, Emil Phillips, Keisha Walkes, Evgeniy L. Artyushkov, Mark L. Kaplan, Vladislav Straltsov, Yauheniya Litvina, Alexei Lyzikov, Victor E. Tihmanovich, Mafalda Borges, Tim Brits, Nicolas De Hous, Stefan De Wachter, Niels Komen, Tomas Menovsky, Xavier Mortiers, Dorien Vermeulen, Nils Vleminckx, Dirk Ysebaert, Julie Bontinck, Danny Bulthé, Bert Dhondt, Ricky Rasschaert, Anne‐Sophie Van Haver, Koen Van Belle, Laurence Verstraeten, Manon Vounckx, Serge Cappeliez, Sébastien D’ulisse, Yasmine De Bruyne, Badih El Nakadi, Tessely Heloise, Matteo Luisetto, Sotirios Marinakis, Maxime Maton, Manon Pigeolet, Claire Viste, Elke Van Daele, Gabrielle van Ramshorst, Mathieu Vandeputte, Marc Duinslaeger, Daniel Jacobs‐Tulleneers‐Thevissen, Yanina Jansen, Ward Janssens, Rastislav Kunda, Nouredin Messaoudi, Michael Ruyssers, Martijn Schoneveld, Jasper Stijns, Ellen Van Eetvelde, Marian Vanhoeij, Wouter Oosterlinck, Jef Van den Eynde, Raf Van den Eynde, Ahmed M. Chaoui, Nicolas Flamey, Marcellin Akpla, Cyrille Kpangon, Souliath Lawani, Hermann Agossou, Hulrich Aouagbe Behanzin, Covalic Bokossa, Mouhamed Agbadebo, Hubert Dewanon, Hugues Yome, Alassan Boukari, Oswald Gbehade, Sèmèvo Romaric Tobome, Labissi Francois Amossou, Francis Dossou, Ismaïl Lawani, Tatjana Barišić, Miran Boras, Zdrinko Brekalo, Ivana Čuljak Blagojević, Ana Damjanović, Vedran Dragisic, Ana Dugandžić šimić, Filip Gunaric, Martin Kajic, Darko Knežević, Tanja Krešić, Valentina Lasić, Ludvig Letica, Vlatka Martinovic, Iva Mikulic, Josip Miskovic, Hrvoje Pehar, Pejana Rastović, Irena Sesar, Violeta šetka‐Čuljak, Martina Soljic, Dejan Tiric, Vajdana Tomić, Anja Vasilj, Anis Cerovac, Igor Hudic, Renato Aguera Oliver, Felipe Azenha Lamonica, Jorge De Medeiros, Henrique Donizetti Bianchi Florindo, Rodolfo Jose Favaretto Filho, Fernanda Costa Pereira, Rodrigo Rodrigues, Antonio Antunes Rodrigues Junior, Fernanda Ruiz de Andrade, Maisa Salvetti, Débora Schalge Campioto, Mayra Tuboi Lamonica, Gustavo Urbano, Ricardo Villela Prado, Nivaldo Alonso, Carlos Ferreira dos Santos, Cristiano Tonello, Robinson Esteves Pires, Ricardo Fernandes Rezende, Igor Reis, Fernando Augusto Lima Marson, Erick Pires Ferreira, Camila Vantini Capasso Palamim, Marcelo Brandao, Tiago Henrique de Souza, Rebecca Maunsell, Alfeu Accorsi Neto, Guilherme Accorsi, Murilo Francisco Fernandes, Camila Machareth, Barbara Viegas Moura, Vanessa Dias, Luciano Guarienti, Lia Regina de Sampaio, Cristiano Vendrame, Tatiane Amorim Coelho, Karin Becker, Andre Dias, Camila Girardi Fachin, Monica Maria Gomes‐da‐Silva, Isabela Moraes, Amanda Pinto, Adriano Seikiti Stychnicki, Nathalia Siqueira Julio, José Mauro dos Santos, Humberto Fenner Lyra Junior, João Carlos Costa de Oliveira, Tiago Rafael Onzi, Marlus Tavares Gerber, Filipe Osni Coelho, Janaína Carla da Silva, Carolina Panis, Daniel Rech, Melissa Avelino, Lais Botacin, Mateus Capuzzo Gonçalves, Gustavo Mendonça Ataíde Gomes, Igor Lima Buarque, Amanda Lira dos Santos Leite, Laercio Pol‐Fachin, Tainá Santos Bezerra, Aldo Vieira Barros, Alcimar Lavareda dos Santos Junior Alcimar, Robson Amorim, Maria Eduarda Bellotti Leão, João José Corrêa Bergamasco, Cintia Cardoso Pinheiro, Rubem Alves Da Silva Neto, Elaine Francisca De Araújo, Lilian Guimaraes, Tatiana Liborio‐Kimura, Isabelle Melo da Camara, Victor Ripardo Siqueira, Juan Rodriguez, Jeancarllo Silva, Rubem Alves Silva Junior, Cláudio Cardoso, Andre Guimaraes, Wislene Sarajane Moreira Alves, Agna Soares Da Silva Menezes, Marcelo Araujo, Kattiucy Brito, Josie Marcelle Lira Albuquerque, Jairo Alberto Dussan‐Sarria, Andressa Souza, Andre Ricardo Stüker, Tiago Bresciani, Luciana Cadore Stefani, Leandro Totti Cavazzola, Tainá Costa, Matheus Dasqueve, Gustavo De Bacco Marangon, Otávio Ritter Silveira Martins, Josy Rodrigues, Guilherme Roloff Cardoso, Brasil Silva Neto, Tilaê Soares, Aline Zanella, Lucas Torelly Filippi, Enilde Eloena Guerra, Ricardo Pedrini Cruz, Antonio Nocchi Kalil, Gustavo Laporte, Moacyr Salem, Murilo de Lima Brazan, Caio Antonio de Campos Prado, Elaine Christine Dantas Moises, Hilda Satie Suto, Alice Gadotti Yasuda, Ana Carolina Tagliatti Zani, Roberto Cardoso Cardoso dos Santos, Geraldo Duarte, Edwaldo Edner Joviliano, Carolina Lourenço Gomes dos Santos, Silvana Maria Quintana, Edwin Tamashiro, Fabiana Cardoso Pereira Valera, Marília Veccechi Bijos Zaccaro, Isis Andreotti, Leonardo Lima, Wilson Salgado, Alonço da Cunha Viana Júnior, Lana Moutinho, Cora Pichler de Oliveira, Isabela Trindade Martins, Isabela Vieira Toledo, Marco Antonio Correa Guimaraes‐Filho, Rodrigo Arrivabeno, Claudio Bovolenta Murta, Danniel Frade Said, Jose Pontes Junior, Felipe Guimarães Pugliesi, Paulo Gregorio, Alessandro Mariani, Fabio Minamoto, Juliana Lourenço da Silva, Bruno Muller, Caio Zanon, Vladimir C. Carvalho, Ana Lucia Munhoz Lima, Priscila R Oliveira, Jorge dos Santos Silva, Mariana Faccini Teixeira, Luiz Paulo Kowalski, Marco Kulcsar, Leandro Matos, Kamilla Schmitz Nunes, Joel Abdala Junior, Emne Abdallah, Samuel Aguiar Júnior, Glauco Baiocchi, Heloisa Galvão do Amaral Campos, Genival Barbosa Carvalho, Igor Correia de Farias, Cassia da Silva, Fabio Fernando Eloi Pinto, Luciana Facure, André Godoy, Jefferson Gross, Felipe José Fernandez Coimbra, Luiz Paulo Kowalski, Bruna Kupper, Fernanda Leite, Matheus de Melo Lôbo, Fabiana Makdissi, Henrique Mantoan, Narimã Marques, Tomas Marques, João Paulo Medici, Silvio Melo Torres, Katheryne Merlos Garcia, Suely Nakagawa, Joao Pedreira Duprat Neto, Rafael Ribeiro Meduna, Ana Carolina Scintini Herbst, Silvana Soares Dos Santos, Bruna Tirapelli Gonçalves, Jose Guilherme Vartanian, Stenio C Zequi, Marcelo Antonini, Carolina Brienze, José Francisco Farah, Rodrigo Gonçalves, Vinicius Machado, Luis Roberto Nadal, Danilo Nadal Rodrigues, Adrieli Pansani, Maria Luiza Rocha, Marcelo Simonsen, Daniela Tsuchiya, Rafaela Vasques, Jorge Amorim, Ana Alyra Carvalho, Rebeca Correia, Brena Costa dos Santos, Ronald Flumignan, Henrique Jorge Guedes Neto, Luis Nakano, Mariana Pereda, Vladimir Vasconcelos, Jamile Barakat Awada, Guilherme Gava, Tiago Riuji Ijichi, Nam Jin Kim, Rafael Nunes, Fabio Pinto, Thiago Henrique Sigoli Pereira, Gustavo Jardim Volpe, Arthur Gatti, Caroline Nardi, Ramon Oliva, Layze Braz de Oliveira, Herica Emilia Félix de Carvalho, Ivonizete Pires Ribeiro, Gabriel Renan Soares Rodrigues, álvaro Francisco Lopes de Sousa, Michel Chebel, Paulo Henrique de Sousa Fernandes, Marcelo Augusto Faria Freitas, Juliano Rodrigues da Cunha, Michel Chebel, Paulo Henrique de Sousa Fernandes, Juliano Rodrigues da Cunha, Zeenia Ather, Dobromir Dimitrov, Emil Filipov, Muhammad Gohar, Elitsa Gyokova, Ivelina Ilieva, Tsvetomir Ivanov, Martin Karamanliev, Vasil Neykov, Boyko Atanasov, Nikolay Belev, Mihail Slavchev, Paolina Kamenova, Kaloyan Tonev, Tsanko Yotsov, Evguenia Hristova, Kolyo Spassov, Tsanko Tsankov, Dragomir Dardanov, Petar Gribnev, Manol Sokolov, James Brown, Chukwuemeka Nwegbu, John Tanyi, Heather Hurdle, Natalia Jaworska, Anthony MacLean, Joshua Ng‐Kamstra, Michael Sander, Salim Al Riyami, Krittika Bali, David Bigam, Khaled Dajani, Angela Dell, Mehran Anvari, Rafik Bolis, David Choi, Susan Ellis, Michael Gupta, Wael Hanna, Dennis Hong, Cynthia Horner, Lea Luketic, Peter Moisiuk, Harsha Shanthanna, Yaron Shargall, Hilda Alfaro, Nawar Alkhamesi, Laura Allen, Muriel Brackstone, Eunice Chan, Davy Cheng, Jason Chui, Nelson Gonzalez, Brent Lanting, S. Danielle MacNeil, Janet Martin, Robert Mayer, Jacob McGee, Mahesh Nagappa, Nicholas Power, Agya Prempeh, Mehdi Qiabi, Hassan Razvi, Emil Schemitsch, Herman Sehmbi, Ushma Shah, Yamini Subramani, Edward Vasarhelyi, Kelly Vogt, Homer Yang, Abdollah Behzadi, Amanpreet Brar, Ali Ghorbani Abdehgah, Saba Balvardi, Liane Feldman, Julio Flavio Fiore, Melissa Hanson, Brent Hopkins, Pepa Kaneva, Lawrence Lee, Julia Leonard, Jad Abou‐Khalil, Andre Martel, Carolyn Nessim, James Stevenson, Susan Lee, Richard Merchant, Michelle Mozel, Samuel Avoine, Laurence Belanger, Mathieu Belanger, Etienne Belzile, Anne‐Sophie Blais, Sofia Boucher‐Kovalik, Cindy Boulanger‐Gobeil, Etienne Cardinal, Pierre‐Olivier Champagne, Jonathan Cloutier, Simon Corriveau‐Durand, Mathieu Cote, Maxime Cote, Mathilde Côté, Valérie Courval, Suzanne Demers, Christine Desbiens, Mehdi El Ouazzani, Karine Girard, ève‐Marie Girard, Karo Gosselin, Helene Khuong, Nathalie Labrecque, Eve‐Lyne Langlais, Audrey Larouche, Andréane Lavallée, Madeleine Lemyre, Sarah Maheux‐Lacroix, Melissa Marien, Patrick Marin, Julie Mauger, Genevieve Milot, Sylvie Nadeau, Sebastien Nguyen, Mélinda Paris, Stéphane Pelet, Marie Plante, Carole Plante, Frederic Pouliot, Marie Claude Renaud, Mathilde Sarlabous, Isabelle Schmit, Pascal St‐germain, Raphaël St‐germain, Brian Johnston, Crispin Russell, Gary Groot, Nicole Labine, Amit Persad, Hong Pham, Melissa Wood, Riordan Azam, Najib Safieddine, Amit Atrey, Andrew Beckett, Daniel Cohen, Sunit Das, Julian Daza, Cheryl Dunkerton, Yosef Ellenbogen, Ciara Hanley, Sorcha Kellett, Amir Khoshbin, Karim Ladha, Amanda McFarlan, Spencer Montgomery, Janneth Pazmino‐Canizares, Hrishikesh Suresh, Duminda Wijeysundera, Antoine Eskander, Ravleen Vasdev, Maira Ahmed, Sami Chadi, Fred Gentili, Aristotelis Kalyvas, Matthias Millesi, Can Sarica, Taariq Shaikh, Leslie St, Jacques, Raha Tabasinejad, Gelareh Zadeh, Marvin Hsiao, Nicole Jedrzejko, Shawn MacKenzie, Peter Black, Claire Broe, Charlotte Dandurand, Rebecca Grey, Philemon Leung, Andrew Lindberg, Juan Mata Gutierrez, Kelly Mayson, Adam Meneghetti, Drew Phillips, John Street, Fernando Heredia, Carolina Carvajal Calderón, Daniel Alejandro Donoso Pizarro, Esteban Fernández, Maria Marta Modolo, ángela Molero, Nastassja Mutarello, Jorge Núñez Lucic, Katherine Ochoa Gaete, Catalina Ruiz Lopez, Rina Sepúlveda, Camila Ulloa, Mauricio Barred a, Matias Dallaserra, Matías Günther Wood, Mauricio Sandoval Tobar, Julio Villanueva, Francisco Garcia‐Huidobro, Sofia Waissbluth, Jimena Dona, Roberto Macchiavello, Carolina Soto Diez, Mario I Escudero, Daniel Igor, Javier Mena, José Tomás Reyes, Catalina Arredondo Soto, Jose Campos, Tyare Fuentes, Javier Gonzalez, Barbara Rivera, Jaime Altamirano‐Villarroel, David Cohn, Luis Marin de Amesti, Ximena Mimica, Pedro Recabal, Camilo Sandoval, Bruno Catoia Fonseca, Monica Contador, Andres Hodali, Camila Pincheira, Andrea Ramos Mantilla, Claudio Salas Garrido, Janina Torres, Marco Valenzuela, Zehua Chen, Jiankun Hu, Jin Tao, Kun Yang, Yuexin Zhang, Yuan Chen, Wang Cunchuan, Wah Yang, Xueli Bai, Tingbo Liang, Tao Ma, Wenhui Lou, Ning Pu, Hanlin Yin, Dinimo Bolivar Saenz, Camilo Caicedo, Diego Felipe Tellez Beltran, Alejandro Escobar, Carolina Perez Granados, Lisbeth Alexandra Urueña Pinzon, Carlos Bonilla, Joaquin Luna, Diana Santana, Carlos Bonilla, Diana Santana, Oscar Serrano, Andrea Garcia, Fernando Giron Luque, Nasly G. Patino‐Jaramillo, Nestor Fabian Pedraza Alonso, Laura Giselle Contreras Baquero, Nestor Augusto Muñoz Botero, Jhoana Andrea Murillo Castellanos, Camilo Ortiz Silva, Andrea Juliana Vega Calvera, Lina M. Acosta Buitrago, Maria Paz Bohórquez‐Tarazona, Paulo Andrés Cabrera Rivera, J Felipe Casas, Valeria Cormane Alfaro, Julian Corso, Bayron Guerra, Albert Franz Guerrero‐Becerra, Akram Kadamani Abiyomaa, Felix Ramon Montes, Manuel Santiago Mosquera Paz, Carlos J Perez Rivera, Camilo Andrés Polanía Sandoval, Natalia Quintana, Carlos Fernando Roman Ortega, Carlos Santacruz, Manuela Téllez, Camilo Alejandro Velandia Sánchez, Fernando Arias‐Amézquita, Luis Felipe Cabrera Vargas, Natalia Cortes Murgueitio, Elkin Escorcia, Kemel Ahmed Ghotme Ghotme, Jorge Luis Gomez‐Mayorga, Alvaro Felipe Guerrero Vergel, Gabriel Herrera‐Almario, Eduardo Londono‐Schimmer, Juan A. Mejia, Guillermo Monsalve, Juliana Rodriguez, Camilo Rodriguez, Juan Nicolas Rodriguez Niño, Luis Martín Rodríguez Ortegón, Daniel Sanabria, Andres Alvarez, Arnulfo Andrade, Javier Ardila‐Montealegre, David Baquero, Edgar Mauricio Barrios Vidales, María Alejandra Caicedo Giraldo, María Camila Carvajal, Maria Castillo, Maria Carolina Castillo Florez, María Angelica Cendales, Danny Conde Monroy, Henry Cortes, Mario Daniel, Jairo De la Peña, Elena Leonor Delgado‐Nieto, Hernando Espitia, Carlos Figueroa Avendaño, Edgar Oswaldo Hernandez Burgos, Andres Isaza‐Restrepo, German Londono, Margarita Maria Maldonado, Andrea Montenegro, Juan Carlos Navarro, Jorge Navarro‐Alean, Katherine Parra Abaunza, Eliana Pulido, Erika M. Ramírez Amaya, Carlos Eduardo Rey Chaves, Natalia Andrea Rivera Rincón, William Mauricio Riveros Castillo, Lizeth Rodriguez Sanchez, Wilson Rubiano, Maria Russi, Juan Carlos Sabogal Olarte, Javier Mauricio Salgado Tovar, Maria Juliana Sanchez, Liliana Silva‐Igua, David Mauricio Solano Varela, Martha Luz Torres, Paula Torres Gomez, Marcial Trillos, Ana María Vargas Patiño, Felipe Vargas‐Barato, Juan Pablo Villate Leon, María Alejandra Wagner Useche, Jesus Acosta, Adriana Almeciga, Luis Becerra Mendez, Rafael Jose Beltran, Ana Bonilla, Miguel Buitrago, Marino Cabrera, Lina Caicedo, Pedro Hernando Calderon Quiroz, Diego Camacho‐Nieto, Carlos Andrés Carvajal Fierro, Sergio Cervera Bonilla, Sandra Diaz, Helena Facundo, Jorge Forero, Mauricio Garcia Mora, Karena Victoria Garcia Tirado, German Fabian Godoy Perez, Felipe Gonzalez, Oscar Guevara, Jairo Alonso Hernandez, David Ricardo Herrera Mora, Juan David Lalinde, Carlos Lehmann, Eduardo Leon Llanos, Byron Eduardo Lopez De Mesa Lopez, Ivan Mariño, Monica Medina, Raúl Eduardo Pinilla Morales, Angela Puerto, Ma, Andrea Quintero‐Ortíz, Juliana Rendón Hernández, Juliana Rodriguez, Elio Fabio Sánchez Cortés, Alvaro Eduardo Sánchez Hernández, Diana Santana, Raul Suarez, Oscar Suescun, Lina María Trujillo, Marco Vanegas, Rodolfo Varela, Jorge Luis Velez Bernal, David Viveros‐Carreño, Daniela Camargo Gómez, Angelica Fletcher, Abel Merchan, Euler Javier Burbano Luna, Indira Cujiño, Albaro José Nieto Calvache, Mauricio Velasquez Galvis, Lina Maria Vergara Galliadi, Orlando Abonia Gonzalez Abonia Gonzalez, Maria Fernanda Acuna Saravia, Roberto Arroyave, Jose Maria Barreto Angulo, Francisco Javier Bonilla‐Escobar, Uriel Cardona, Ivan Castañeda Giacometto, José Luis Castillo, Diego Fernando Castillo‐Cobaleda, Diego Jose Caycedo Garcia, Brenda Marcela Coll Tello, Juan Carlos Dueñas‐Ramirez, Oscar Andres Escobar Vidarte, Luis Figueroa, Herney Garcia‐Perdomo, Alden Gomez, Laura Gomez, Alejandro Gomez, Ana María Grande‐Gil, David Guarin, Natalia Guzman, Enrique Herrera Castañeda, Marisol Hinaoui, Anuar Armando Idrobo Escobar, Christian Kammerer Kammerer, Sergio Leon, Carlos Antonio Llanos Lucero, Angie López, Alexander Maximiliano Martinez‐Blanco, Antonio José Montoya Casella, Ricardo Andres Niño Corredor, Sebastian Ordoñez, Diego Alfredo Palta Uribe, Andrea Carolina Perea Serna, July Ríos, Gabriel Ríos‐Samper, Juan David Rivera Garcia, Juan Camilo Salcedo Moreno, John Sandoval, Guillermo Alberto Sarmiento Ramirez, Paola Andrea Tabares Romero, Ricardo Urzola, Iliana Maria Valdes‐Duque, Miguel Velasquez, Lina Maria Villegas, James Zapata‐Copete, José Omar Zorrilla Lara, Mauricio Zuluaga Zuluaga, Alejandra Echeverri Moreno, Juan Sebastian Figueroa, Rafael Figueroa‐Casanova, Adolfo Enrique Gómez Ortiz, Maria Fernanda Gonzalez Mosos, Alejandro González‐Orozco, Juan Jose Jaramillo Roncancio, María Camila Leyva Martínez, Monica Mosos, Juan Sebastian Ramirez, Henry Andrés Rodríguez, Juan David Saavedra Henao, María Alejandra Torrado Varón, Oscar‐Julián García‐Montoya, Leidy Natalia Idarraga Ramírez, Lorena Ocampo, Monica Cardona Marin, Ana Maria Marin Gonzalez, Carlos Andres Marulanda Toro, David Alejandro Mejia, Maria Clara Mendoza Arango, Luis Emiro Vanegas, Yicel Alvarez Martinez, Sandra Aruachan Vesga, Dayana Vanessa Baron Fuentes, Alejandra Caballero Salas, Eneida Diaz Martinez, Gustavo Antonio Martinez Estrada, Kelly Viloria Campo, Juan Antonio Corralez Alvarez, Cesar David Galindo Regino, Angela Maria Giraldo Velasquez, Valentina Gutiérrez Perdomo, Carlos Enrique Melo Moreno, Jorman H. Tejada, Jesus Hernan Tovar, German Alirio Tovar, Edison Alexander Benavides Hernández, Jose Andres Calvache, Claudia Milena Orozco‐Chamorro, Gustavo Adolfo Angel, Christian Ali Buesaquillo, Jose Andres Calvache, Victor David Olave Montaño, Andrés Sánchez‐Gómez, Daniela Patricia Escalante Ureche, Jairo Martinez Garrido, Emileth Montenegro, Eben‐ezer Genda, Nicolas Amisi, Fabrice Eboma, Kristina Bitunjac, Karlo Grulović, Marijana VuČković, Goran šantak, Emanuel Borovic, Ana Bosak Versic, Damir Hasandić, Natasa Poldan Grabar, Suzana Srsen Medancic, Lucija Brkic, Sara Cokarić, Petra Pavic Palac, Zeljka Samac, Josipa Tomić, Marija Vrdoljak, Ivan Bacic, Bernarda Bakmaz, Nikolina Bratošević VuČiČić, Domagoj Brzic, Samir Canovic, Emilio Dijan, Maja Grgec Dragicevic, Robert Karlo, Suzana Konjevoda, Petra KovaČević, Ivan KovaČić, Frane Markulić, Luka Matak, Jakov Mihanovic, Domagoj Morović, Gordan Perišić, Andrea Simic, Neven Skitarelić, Matea Veršić, Vanja žufić, Matea Zuzul, Srdan Ante Anzic, Iva Carevic, Dubravka Heli Litvic, Duska Markov‐Glavas, Tatjana Savic Jovanovic, Goran Augustin, Jerko Biloš, Bojan BioČina, Vedrana Biosic, Dino Bobovec, Iva Botica, Boris Bumber, Petra Čerina, Ana Danic Hadzibegovic, Katarina Duric Vukovic, Hrvoje Gasparovic, Lucija Gatin, Kresimir Grsic, Ika Gugić Radojković, Ivan JelČić, Zeljko Kastelan, Juraj Kolak, Tomislav Kopjar, Tomislav Kulis, Kristian Kunjko, Marjan Maric, Marcel Marjanović Kavanagh, Borna Milicic, Trpimir Morić, Miram Pasini, Luka Penezić, Drago Prgomet, Ratko Prstacic, Andreja Prtorić, Rudolf Radojković, Ivan Romić, Tomislav SeČan, Dora škrljak šoša, Juraj Slipac, Mislav Tomic, Jurica Zedelj, Toni Zekulić, Zoran Zimak, Mia Lorencin, Ivica Luksic, Matija Mamic, Pablo Mijahil Avilés Jiménez, Norberto Miranda Espinsa, Ailén Sánchez Cruz, Stavros A. Antoniou, Michael Kakas, Yiannis Panayiotou, Heyam Almezghwi, Kalbim Arslan, Hasan Besim, Ali özant, Necdet özçay, Nikolaos Gouvas, Georgios Kokkinos, Panayiotis Papatheodorou, Ioanna Pozotou, Olga Stavrinidou, Anneza Yiallourou, Lukáš Burda, Michal Dosoudil, Lubomir Martinek, Wladyslaw B. Gawel, Milan Lerch, Matúš Peteja, Jan žatecký, Toman Daniel, Martin Formánek, Veronika Javurkova, Markéta KepiČová, Jaroslav Klat, Nicole MaceČková, Petr Matousek, Ondrej Simetka, Petr Vavra, Karol Zelenik, Jan Černý, Sami Khan, Robert Lischke, René Novysedlák, Martin Přibyl, Temoore Younus, Yenuksha Amarasena, Karel Klíma, Setareh Pirmorad, Zuzana Hotová, Aleš Mladěnka, Nulvin Bozo, Peter Christensen, Julie Lykke Harbjerg, Louise Hviid, Helle ø Kristensen, Mira Mekhael, Mette Fugleberg Nielsen, Laerke Paulsen, Saija Sinimäki, Louise Zinck Mogensen, Peter Bonde, Anders Lyng Ebbehøj, Anne‐Sophie Fenger, Aleksander Fjeld Haugstvedt, Christine Hangaard Hansen, Maria Lovisa Jönsson, Lars N Jorgensen, Peter‐Martin Krarup, Anne‐Louise Lihn, Christian Meyhoff, Helena Otte, Anas Ould Si Amar, Henrik Palm, Nis Schlesinger, Henry Smith, Ida Tryggedsson, Anne Reiss Axelsen, Jens Kristian Bælum, Mark Bremholm Ellebaek, Signe Bremholm Ellebæk, Anders Hogh, Maria Acosta, Yancy Acosta, Pedro Baez, Sylvia Batista, Luis Alfredo Betances, Luis Rodolfo Bonilla, Daniel Cabreja, Wilton Cabrera Cruz, María Mabel Collado Expósito, Aldo Crespo, Octavio Cruz‐Pineda, Claudio Samuel D’óleo García, Pedro Díaz, Remberto Escoto, Jatnna Figueroa, Luis Garcia, U Garcia‐Dubus Rodriguez, Humberto Gomez, Adrian Grullon, Fatherin Guerrero, Lillian Guzman, Thelma Rocío Jiménez Mosquea, Engels Lazala, Ariela Lopez, Leeany Maletta Francisco, Jhomayri Mercado, Herisardy Munoz, Jeffrey Paulino, Maricely Ambar Perez Fernandez, Rudeily Reyes, Lilia Rosa Reyes Guilamo, Ruben Rivas, Claudia Alejandra Rivas Torres, Ada Rodríguez, Julia Rodriguez‐Abreu, Madelin Tamar Rosario Villa, Eleazar Santana, Joel Soto, Nassin Tactuk, Cristina Kristel Tonos Sardiñas, Raul Ubinas, Jose Victoria, Aaron Villegas, Ricardo Acra‐Tolari, Larissa Beltran, Luis Fernand Betances, Alejandro Blaubach, Denisse Idalia Campos Mejía, Nathalia Capellan, María Fernanda Cedeño Bruzual, Saray Cordero Spencer, Gabriella Cuevas Lantigua, Luis De Jesus, Gabriela Díaz, Pedro Pablo Díaz Vásquez, Karla Disla, Jesús Antonio Echavarría Uceta, Paola Irina Eusebio Jimenez, Damaris Fernandez, Marlin Fernandez Camilo, Jiomar Manuel Figueroa Germosen, Maruel Fortunato, Leyla Gabriel Fernández, Alicia German Dihmes, Héctor Herrera, Elizabeth Leon Cuevas, Jose Alejandro Mata, Mirian Mateo De La Cruz, Dolores Mejia De la Cruz, Marcos Mirambeaux, Fabio Ortiz De La Cruz, Henry Paulino, Gabriela Pelletier, Merycarla Pichardo, Rodriguez Pumarol Próspero Enrique, Julio Rivas, Ann Stephany Sanchez Marmolejos, Roman Santana Santana, Irina Suero Almanzar, María Magdalena Vásquez Sánchez, Nicolás Campuzano, Eddy Lincango‐Naranjo, José Ricardo Negrete Ocampo, Maria Armas, Alvaro Santiago LeMarie Guerra, Gustavo León Vizcaya, Omar Ahmed Abdelwahab, Ahmed O. Elmehrath, Mohammed Ezzat Mostafa, Gamal Amira, Ibrahim Sallam, Mohamed Sherief, Ahmed Abdelmajeed, Mostafa Abdou, Ahmed Abo Shanab, Aya Abodeeb, Roger Aboelkhel, Nour Eldin Abosamak, Amna Abou Bakr, Samar Aboubakr, Galal Abouelnagah, Yossof Abouelnagah, Omar AbouMadawy, Dina Adel, Nermeen Afifi, Sara Ahmed, Abedelrahman Ahmed, Mohamed AL Sayed, Abdelrahman Alberkamy, Mohamed Aldahma, Ahmed Ali, Mostafa Ali, Mohamed Amin Bakr, Alaa Anter, Doaa Asal, Fouad Ashoush, Olfat Ashraf, Abdel Rahman Ashraf, Rewan Atta, Salma Badr, Mohamed Bahaaeldin, Mostafa Bastawesy, Sara Darwish, Rasha El Kharashy, Ziad Elassar, Seifeldin Elbadawy, Dina Elkhity, Alromisaa Elsaka, Manal Elsayed, Mostafa Elsayed Elsayed Hewalla, Mohamed Elshafey, Youssof Eshac, Fares Eshac, Amr Essameldin, Moataz Ewedah, Yara Ezz, Mohamed Farag, Inas Gadelkarim, Ziyad Gadelrab, Dina Gamal, Mira Ghaly, Nathalie Girgis, Omar Gouda, Nancy H. El Goweini, Nour Hafez, Youssef Hafez, Abdelkader Hamed Abdin, Rodina Hanno, Aliaa Hassanin, Mohamed Hassanin, Hanan M. Hemead, Abdelrahman Hemida, Maher Hosain, Ahmed Hossam, Hamza Hussein Aly Salama Aly, Mohamed Ibrahim, Abdelrahman Ibrahim, Nourhan Ibrahim, Noha Mohamed Salah Ibrahim Moussa Hamouda, Monica Iskander, Islam Khalifa, Mostafa Kotb, Nada Mahmoud, Alaa Mahmoud Abo Shabana, Nora Mamdouh, Maram Metwalli, Khaled Metwally, Mahmoud Moghazy, Moustafa Mohamed, Mohamed Mourad, Esraa Moustafa, Kamilia Mubarek, Nour Eldin Nader, Ahmed Nasser, Samaa Omar, Mostafa Shehata Qatora, Mohamed Ragab, Marawan Ragal, Dina Ramadan, Rana Ramadan, Sara Ramadan, John Romany, Ahmed Sabry, Yasmeen Said, Sara Salamah, Ahmed Mostafa Saleh, Mona Saleh Mesbah Mohamed Elkaffas, Alaa Salem, Hashem Salim, Ahmed Samih, Ahmed Samir Abdelaal, Ahmed Shaheen, Yassmeen Sharafeldin Mohammed, Sameh Shehata, Abdelrahman Shehata, Mohamed Shemeis, Karim Shenit, Mennatallah Sheta, Asmaa Soffar, Yousef Tanas, Ahmed Tarek, Nermin Yehia, Omar Khaled Mohamed Eid, Ammar Yasser Abdulfattah, Ahmed ElSaghir, Mohamed Fouad Elganainy, Ahmed Hossam Eldin Fouad Rida, Omar Ibrahim Elsayed, Omar Elmandouh, Omar Hamam, Mostafa Ahmed Shehata, Areej A. Abdelaziz, Ahmed M. Abbas, Wael Abd El‐Ghani, Hossam Aldein S. Abd Elazeem, Mustafa Abd Elsayed, Ahmed Yassien Abd‐Elkariem, Shimaa Abdalla, Mahmoud Abdel‐Aleem, Khaled Abdelazeem, Mahmoud Abdelfattah, Mohammed Abdelhafez, Mohamed M. Abdelkarem, Ali Abdelraouf, Lamess Abdullaha, Hossam Abubeih, Moaiad Eldin Ahmed, Nagm Eldin Abu Elnga Ahmed, Ahmed Ahmed, Sarah Ahmed Saad, Sherif Alaa, Ali Alhussaini, Abdelrahman Ahmed Abdelrahman Ali, Ibrahim Ali, Wagdi Ali, Mohamed Ali Mohamed, Mohamed Ashraf, Muhammad Bassiouni, Mohamed G. El‐adawy, Mohammad El‐Sharkawi, Hussein Elkhayat, Khaled Elmaghraby, Shady Elsdfy, Mohamed Elsharkawy, Almoutaz Eltayeb, Mohamed Esmat Mohamed, Esraa Essam, Mahmoud Fahd, Osama Farouk, Rabea Gadelkareem, Ahmed Ghoneim, Mohammed Hamada Takrouney, Ahmed Hamdan, Abd El‐Rahman Hamed, Ahmad Hasan, Ramy A. Hassan, Mohamed Abdelghafor Hassanin, Islam Hawal, Kerollos Henes, Mohamed Omar Herdan, Helal F. Hetta, Omar Ibrahem, Mostafa Ibrahim, Islam H. Ibrahim, Mahmoud Kamel, Mohamed Khallaf, Shrouk M. Elghazaly, Abobakr Mahfouz, Osman Mahmoud, Wesam Mahran, Doha Mahrous, Abadeer Marsis, Ayman Mohamed, Aliae Mohamed Hussein, Amr Mohamed Sayed, Mahmoud M Mohammed, Ahmed Mokhtar, Fatma A. Monib, Ahmed Nageeb, Mariam Albatoul Nageh, Mohammed Nageh, Nehal Gamal Omar, Abdelrahman Ragab, Abdelrahman Ramadan, Abdallah Rashad Temerik, Mahmoud M. Saad, Aya Sabry, Hadeer Safwat, Omar Salah, Ahmed Elhussiny Salah Mahmoud, Ahmed Saleh, Mahmoud Sallam, Ahmed Samir, Reem Sayad, Esraa Sayed, Ahmed S. Sedik, Mohammed Shahine, Abdelrahman Shehata, Antonios Soliman, Wael Soliman, Mohamed Gamal Taher, Randa Wanees, Ebrahim Ahmed Yousof, Ahmed Youssef, Omar Zein Elabedeen, Ahmed Emad Sayed Hassan, Shrouk Abdel Fattah, Ahmed Abostate, Samar Ali, Saad Ali Saad Salama, Mohamed Allam, Ahmed ALsadek, Ebrahim Arafa, Ahmed Barakat, Mohammed El Sherpiny, Gehad Elbehairy, Mahmoud Eleisawy, Abdelrahman Elgendy, Rewan Elhawary, Sherif Eltregy, Mahmoud Hamdy, Gehad Hassan, Aya Khalifa, Ahmed Mahmoud, Ahmed Mohamed Altukhy, Kareem Noah, Yasser Noureldin, Mostafa Nowar, Mohamed Reda Loaloa, Moustafa Saad, Ahmed Saad Elsaeidy, Khaled Saad Elsaeidy, Hesham Sabry, Mostafa Sameh, Ahmed Tarek Said‐elnaby, Mohamed Zahed, Mohamed Zahran, Abdelrahman Zaid, Ahmed Zaki Zoghary, Mohamed Atef, Ahmed Mohamed, Mohamed Youssef, Mahmoud Hossameldin Saad Abdelhamid, Emad Alazab, Ahmed K. Awad, Amr Darwesh, Lobna El Fiky, Ibrahim ElGarhy, Badr Mostafa, Mohamed Adel Nassef, Mohamed Qassem, Mahmoud Shaban, Mahmoud Mohamed Mohamed Shalaby, Abdelrahman Wahba, Omar Youssif Omar Fouad, Menan Elsadek, Mohamed Fahmy, Dalia Gad, Ahmed Abdelsamed, Hesham Abozied, AbdulHakeem Bayomy, Mohamed Elsalhy, Ahmed Fahim, Ahmed Seleim, Eman Afifi, Fatma Alzahraa Gamal, Mennatullah Gamal, Omnia Eldesouky, Mohamed Hatem Elmetwalli Eldwini Eldwini, Mostafa Medhat Fahmy Fahmy, Ahmed Hussein, Abdallah Ouf, Omar Sami, Salem Shaat, Abdullah Wael Mostafa Khalil Bahi, Abdurhman Atea, Ibrahim Gamal, Mohamed El Kassas, Wael Omar, Ahmed Tawheed, Saeid Al‐oribi, Nuran Khaled Aly, Mahmoud ElFiky, Alaa Eldine Elmaghraby, Abdulrahman Elrahmany, Motaz Elsherbeeny, Youssef Helmy, Ahmed Nabil, Ahmed Samir Farahat, Mostafa Soliman, Amr Wassef, Abdelrahman Abdelrahman, Seif ElSaban, Galal Ghaly, Rana Hamdy, Hamada Mondy, Lubna Abdallah, Abdelrhman KZ Darwish, Mohamed Rabea, Mohammed A Azab, Ahmed Y Azzam, Alzhraa Salah Abbas, Sherief Ghozy, Marwa El‐Deeb, Mohamed Fawzy, Galal Ghaly, Maher Ibraheem, Abdelrahman Bakry, Sarah Elnems, Randa Elsheikh, Mohamed Ibrahim Gbreel, Mahmoud Hafez, Mohamed Jammal, Khaled M Hamam, Abdelrahman M Makram, Omar Mohamed Makram, Salma Rabie, Sherine Yousery Askalany, Mokhtar Mohamed Ibrahim Abushanab, Abdulrahman Eid, Mohammed Refaat Ibrahiem Amin El Ghalid, Ahmed Adel Abdelaty, Elsayed A. Fayad, Asmaa Radwan, Asser Sallam, Moataz Sallam, Ahmad Shokry, Ahmed Mohamed Farouk, Ahmed Shehta, Ahmed Elfallal, Hossam Elfeki, Mohamed Elsaeed, Mahmoud Elsaid, Sameh Emile, Amany Makroum, Dina Mohamed Elsaid, Mohamed Mostafa, Mohamed W Omar, Mohamed Rezk, Ahmed Sakr, Aly Sanad, Mostafa Shalaby, Mohammed Shawqy, Mohamed Shetiwy, Ashraf Shoma, Noura Tawakl, Asmaa Yunes, Mohamed Abdelkhalek, Amr Abouzid, Khalid Atallah, Rami Elmorsi, Ahmed Elsherbini, Khaled Gaballa, Omar Hamdy, Mohamed Hamdy, Islam Hany Metwally, Basel Refky, Mosab Shetiwy, Mohammed Zuhdy, Mohammed A Zahran, Fatima Abdellah, Ahmed Abdelmawla, Ahmed Abdrabou, Asmaa Abubakr, Rawda Al Gohary, Mohammed Hamdy Al‐Shazly, Mohamed Ali, Abdallah R Allam, Aya Aposaeeda, Mohammed Asfour, Ammar Ayman, Abdelrahman Azzam Omran, Ibrahim Tawfiq Daghash, Mahmoud Ahmed Ebada, Taher Eid, Ahmed El Kelany, Esraa El Shemy, Mohammed El‐Hag‐Aly, Omar ELgamal, Mahmoud Elghoury, Ahmed Farag ElKased, Takwa Hamed Ellakwa, Hamed Ellakwa, Amr Elmeanawy, Salma Elnoamany, Alaa Elsabagh, Abdelrahman Elsawey, Enas Elshabrawy, Seliman ELShakhs, Naira Elsoudy, Esraa Ezzat, Ahmed Fawzy, Abrar Gamal, Ahmed Gameel, Khaled Gharbia, Mohamed Ghonaim, Alaa Ghonaim, Ahmed Hafez, Abdelrahman Hafez, Zainab Ismail, Mohamed Khaled, Mohammed Meselhy, Pola Mikhail, Mervat Mohamed, Mohamed Mougahed, Abdulla Mustafa, Ahmed Nada, Janna Omran, Mohamed S. Ebiad, Ibrahiem Saleh, Nourhan Salem, Osama Salem, Rehab Samaka, Ahmed Samir, Salma Selim, Ghada Shalaby, Hoda Sherif, Hatem Soltan, Mahmoud Wahbah, Eman Zakaria Abdelbary, Ahmad Helmy Zayan, Abdelrahman Afify, Hossam Ali Hadiya, Mohamed Jamal Elshref, Mostafa Mohamed Ahmed, Nourhan Nasser, Hesham Abdeldayem, Ibrahim Abdelkader Salama, Khaled Ammar, Islam Ayoub, Amr Aziz, Mohammad Taha Badawy, Mohamed Balabel, Yahya Fayed, Emad Hamdy Gad, Maher Gomaha, Essam Hammad, Osama Hegazy, Elsayed Hegazy, Tarek Ibrahim, Mostafa Kallaf, Mahmoud Macshut, Ammar Magdy, Ahmed Oteem, Ahmed Sallam, Samy Samaan, Mohamed Sharshar, Ahmed Elshawadfy Sherif, Hany Shoreem, Elsayed Soliman, Hossam Eldeen Soliman, Taha Yassein, Hazem Zakaria, Abdullah Eldaly, Sarah Mashaly, Sherief Abd‐Elsalam, Wafaa Abdel‐Elsalam, Ahmed Abdullah Shaalah, Mohamed Elbahnasawy, Ismael Elhalaby, Mohamed Hamada, Sarah Hamdy Soliman, Ahmed Hawila, Mohamed Sherif Morsy, Mohmed Naieem, Mohammed Nasreddin, Samar Salman, Sameh Sarsik, Ahmed Shabana, Engy Tolba, Mohamed Zagho, Danilo Alfonso Arévalo Sandoval, Liisa Kams, Tõnu Rätsep, Karolin Riips, Oumer Abdurehman, Azarias Admasu, Ataklitie Berhea, Yegeremu Eado, Nebiyou Hailu, Meklit Kidane, Abdurezak Mohammed, Khalid Mohammed, Abraham Negussie, Admasu Tibelt, Mersha Abebe Woldemariam, Lydya Yonael, Megersa Alemu, Tigist Fisseha, Abenezer Tirsit Aklilu, Frehun Asele, Solomon Assefa, Dawit Azmach, Philimon Bekele, Mickyas Mamo, Nahom Maru, Haile Mekuria, Abel Menkir, Filagot Mikru, Kibruyisfaw Shumbash, Eyerusalem Siraw, Hilkiah Suga, Natnael Sumoro, Aklilu Teka, Efeson Thomas, Dereje Woldemariam, Estifanos Wubishet, Metasebia Abebe, Engida Abebe, Kirubel Abebe, Fitsum Asfaw, Eyerusalem Bergene, Mahder Eshete, Fitsum Gebreegziabher Gebrehiwot, Yetsedaw Gedefaw, Mulualem Wondafrash Mengesha, Netsanet Mengiste, Abeje Menjeta, Mahteme Bekele Muleta, Sena Sefera, Yonatan Tedla, Daniel Teklu, Henok Teshome, Leake Tirfe, Sahle Tsegabrhan, Milkias Tsehaye, Bereket Worku, Million Molla, Melka Supha, Tewodros Taye, Nebyou Abebe, Berhanu Alemu, Abera Chanie, Hailegebriel Degefu, Dawit Desalegn, Hiwot Gebre, Samuel Hailu, Husnia Hussen, Dawit Kassa, Tsegazeab Laeke, Tihitena Negussie Mammo, Samuel Negash, Abat Sahlu, Abraham Genetu Tiruneh, Amanuel Wolde, Hanna Getachew Woldeselassie, Mnewer Y. Ahmed, Betelhem Zewdneh, Jibril Fentaw, Shemsedin Salia, Ketema Tabore, Seid Mohammed Yasin, Yemisirach Bizuneh Akililu, Addisalem Gurara, Tassew Abreha, Teklebirhan Abrha, Gaym Beyene, Niguse Hailu, Mulu Atsbaha Weldu, Desalegn Abdissa, Abebe Megersa, Teshome Tefera, Mequannet Tesfaw, Melatework Wolle, Dawit Asmamaw, Biniam Zemedu Assefa, Bahru Atnafu, Dereje Bedane, Ephrem Bekele, Aderaw Getie, Solomon Melkamu, Workineh Mengesha, Getachew Shumye, Gashaye Tagele, Abrham Amare Tesfa, Wubshet Workneh, Sileshi Genetu, Eneyew Mebratu, Lidya Chanyalew, Tilahun Deresse, Adissu Girma, Marta Seid, Dagim Shimelash, Abrham Shitaw, Bereket Tsegaye, Tewabe Ayalew, Yoseph Solomon Bezabih, Tesfaye Diress, Berhanu Kassahun, Bersabeh Kassaye, Esubalew Mulugeta, Getasew Tesfaw, Beminet Yimenu, Semir Benecha, Silamlak Sisay, Mudesir Aman, Kebebe Bekele, Adem Ibrahim, Alem Mekete, Abdi Tesemma, Moa Jira, Adnan Abdulkadir Mohammed, Yasir Younis Abdullahi, Ephrem Adem, Mahlet Ahmedin, Yared Assefa, Fitsum Ayde, Alazar Berhe, Zersenay Gebremeskel, Mengistu G Mengesha, Eneyew Getachew Siyoum, Sintayehu Teresa, Zinaye Wude, Fasika Yemer, Dagnachew Yohannes, Ewunetu Zeleke, Gulilat Zerihun, Derje Worku Degefe, Habtamu Derilo, Hankore Tamirat Derilo, Nebiyu Eliyas, Firew Bayissa, Eyueal Degefa, Yadani Deressa, Lidya Gemechu, Tegenu Gurmu, Ashenafi Kasaye, Lemi Melese, Lemesa Muleta, Gersam Mulugeta, Seifu Taye, Abraham Teshome, Birhanu Tesso, Gebreagziabher Gebrekirstos, Mhreteab Haile, Haftamu Kassa, Mohammed Saddik, Anteneh Tadesse, Alemneh Mengist, Melkamu Nidaw, Elise Sarjanoja, Juuso Heikkinen, Olli Helminen, Heikki Huhta, Joonas H Kauppila, Tommi Kotkavaara, Matti‐Aleksi Mosorin, Joel Pitkänen, Cheng Qian, Jaakko Sinikumpu, Henri Sova, Mikko Tastula, Ville P Virta, Jérémie Bettoni, Stéphanie Dakpé, Bernard Devauchelle, Nolwenn Lavagen, Sylvie Testelin, François Bastard, Kim Bin, Sophie Boucher, Renaud Breheret, Olivier Fouquet, Alexandre Gueutier, Nicolas Henric, Alexis Kahn, Jean‐Daniel Kün‐Darbois, Didier Moukoko, Anna Pineau, Guillaume Podevin, Françoise Schmitt, Fadi Alshawared, Carlos Daniel Beyrne, Lionel Jouffret, Laurene Lugans, Lysa Marie‐Macron, Alexandre Doussot, Zaher Lakkis, Omar Ahmed, Louy Alnajjar, Tommaso Cipolat Mis, Souha Fliss, Audrey Giocanti‐Auregan, Thomas Gregory, Patrice Guiraudet, Adrien Le Fouler, Emmanuel Martinod, Ilaria Onorati, Marine Peretti, Julien Quilichini, Dana Radu, Tresallet Trésallet, Alban Zarzavadjian Le Bian, Luke Harper, Christophe Andro, Marc Danguy des Déserts, Alexis Maffert, Benoît André, Tracy Chapman, Maxime Halden, Julie Fayon, Catherine Mattevi, Karem Slim, Herjean Marion, Romain Verhaeghe, Lynda Bendjemar, Charre Lionel, Elie Mikhael, Rosa Montero Macías, Andrea Police, Vincent Villefranque, Enrico Volpin, Edouard Girard, Bertrand Trilling, Emmanuel Boleslawski, Alexandre Chebaro, Houlzé‐Laroye Constance, Vincent Drubay, Mehdi El Amrani, Clarisse Eveno, Katia Lecolle, Louis Martin, Barbara Noiret, Guillaume Piessen, Stephanie Truant, Philippe Zerbib, Estelle Aubry, Armande Subayi Nkembi, Laurent Arnalsteen, Franck Denimal, Antoine Lamblin, Quentin Ballouhey, Benjamin Barrat, François Caire, Niki Christou, Laurent Fourcade, Jerome Laloze, Margaux Mekann Bouv‐Hez, Henri Salle, Abdelkader Taibi, Jeremy Tricard, Julie Usseglio, Sophie Chopinet, Emilie Gregoire, Diane Mege, Marion Delpont, Clement Jeandel, Claire Blanchard, Vincent Crenn, Stéphane de Vergie, Waast Denis, Emilie Duchalais, Henri Fragnaud, Nicolas Regenet, Jerome Rigaud, Yoann Varenne, Philippe Anract, Maxime Barat, David Biau, Pierre‐Alban Bouche, Raphaël Dautry, Anthony Dohan, Louis Idier, Elena Lang, Camille Thouny, Stylianos Tzedakis, François Audenet, Antoine Cazelles, Alexandre Chamouni, Mehdi Karoui, Gilles Manceau, Arnaud Mejean, Célia Crétolle, Pauline Clermidi, Erik Hervieux, Tristan Langlais, Lorenzo Leonelli, Emeline Maisonneuve, Beaud Nicolas, Ophelie Perrot, Doriane Prost, Anne Thomin, Thouement Thouement, Noémie Girard, Yoann Athiel, Richard Berry, Guillaume Boddaert, Stéphane Bonnet, Nathalie Cathala, Christel Conso, Christine Denet, Anaïs Laforest, Yael Levy‐Zauberman, Petr Macek, Annick Mombet, Didier Ollat, Adriana Scamporlino, Agathe Seguin‐Givelet, Frederic Zadegan, Chamakhi Ahmed Amine, Baratte Baratte, Emmanuel Chartier‐Kastler, Nathalie Chereau, Pierre‐Antoine Colas, Igor Duquesne, Sebastien Gaujoux, Laurent Genser, Gaëlle Godiris Petit, Claire Goumard, Ariola Hasani, Chetana Lim, Sophie Martellotto, Charlotte Melot, Fabrice Menegaux, Ugo Pinar, Marc Pocard, Morgan Roupret, Olivier Scatton, Thomas Seisen, Noullet Séverine, Célia Turco, Elie Chouillard, Belinda De Simone, Paul Beganton, Matthieu Boisson, Denis Frasca, Thomas Kerforne, Damien Bergeat, Lisa Corbiere, Anis Gasmi, Marwane Ghemame, Sonia Guérin, Zine‐Eddine Khene, Marie Livin, Fedy Mahmoud, Betty Maillot, Aude Merdrignac, Frederic Mouriaux, Fabien Robin, Laurent Sulpice, Charles Vazeux, Alexis P Arnaud, Nicolas Bertheuil, Soline Bonneau, Clément Thierry Cazemajou, Ianis Cousin, Coralie Defert, Elisa Fustec, Melodie Juricic, Vincent Lavoue, Gwenaël Mevel, Krystel Nyangoh Timoh, Annaëlle Renault, Philippe Violas, Lilian Schwarz, Jean Jacques Tuech, Tristan Morichau‐Beauchant, Francesco Nappi, Radwan Kassir, Frederique Sauvat, Elie Haddad, Aurélien Scalabre, Sophie Vermersch, Federico Migliorelli, Andrea Patrizi, Romain Verhaeghe, Fabien Fredon, Alexia Roux, Adeline Aimé, Anne‐Cecile Ezanno, Brice Malgras, Zineb Cherkaoui, Antonio D’urso, Emanuele Felli, Cristians Alejandro Gonzalez, Mihaela Ignat, Didier Mutter, Patrick Pessaux, Barbara Seeliger, Michel Vix, Hugo Gornes, Charlotte Vaysse, Kélig Vergriete, Pierre Berthoumieu, Ludivine Genre, Hélène Le Gall, Vincent Misrai, Trocard Pierre, Olivier Abbo, Marc Chalhoub, Martina Aida Angeles, Mathilde Del, Alejandra Martinez, Laurent Brunaud, Thomas Fuchs‐Buder, Antoine Vancon, Camara Abraham Faya, Elvam Asaph, Elisee Baruwa, Gwen Hofman, Solomon Machemedze, Michael Mayombo Idiata, Roger Muhemi, Olivier Ndizeye, Elysé Nkunzimana, Jennifer O’Connor, Zachary O’Connor, Simplice Tchoba, Natacha Boumas, Zaza Demetrashvili, Grigol Devidze, Givi Pisarevi, Sabine Baumgarten, Linda Grüßer, Frank Hölzle, Pascal Kowark, Ana Kowark, Ali Modabber, Rolf Rossaint, Benedikt Schäfer, Julia Wallqvist, Philipp Winnand, Sebastian Ziemann, Matthias Anthuber, Tobias Broecheler, Florian Edlinger, Yvonne Goßlau, Alexander Hyhlik‐Duerr, Florian Maksymiw, Ehab Shiban, Florian Sommer, Björn Sommer, Sebastian Wolf, Sebastian Zerwes, Katharina Beyer, Carsten Kamphues, Johannes Christian Lauscher, Lucas D. Lee, Florian N Loch, Christian Schineis, Ilgar Aghalarov, Orlin Belyaev, Chris Braumann, Annika Enste, Tim Fahlbusch, Torsten Herzog, Philipp Höhn, Julian Horn, Julia Jedanowski, Julia Knipschild, Andreas Minh Luu, Prem Vignesh Mohan, Leonie Siemen, Illya Slobodkin, Johanna Josefine Strotmann, Waldemar Uhl, Katerina Wolf, Mark Coburn, Eva Egger, Klaus Eichhorn, Jana Enderes, Frederick Far, Alina Franzen, Tim R. Glowka, Erdem Güresir, Alexis Hadjiathanasiou, Jörg C. Kalff, Zaki Kohistani, Steffen Manekeller, Alexander Mustea, Chris Probst, Thomas Randau, Florian Recker, Patrick Schuss, Nadine Straßberger‐Nerschbach, Sebastian Strieth, Hendrik Treede, Cornelius J. van Beekum, Hartmut Vatter, Markus Velten, Tim O. Vilz, Dieter Wirtz, Maria Wittmann, Michael Behr, Dirk Rolf Bulian, Benedikt Marche, Tobias Moczko, Robin Otchwemah, Sissy‐Amelie Schulz, Panagiotis Thomaidis, Christiane Bruns, Claus Cursiefen, Christian Domröse, Hans Fuchs, Ludwig Maximilian Heindl, Michael R. Mallmann, Christoph Mallmann, Dominik Alexander Ratiu, Alexander Christopher Rokohl, Ulrich Bork, Ulrich Canzler, Marius Distler, Sandra Korn, Cornelia Meisel, Marcus Meusel, Andrea Petzold, Christian Praetorius, Janusz von Renesse, Juergen Weitz, Pauline Wimberger, Nour Alkhanji, Georg Fluegen, Stephen Fung, Kefah Jaber, Wolfram Trudo Knoefel, Christian Vay, Octavian Clonda, Tatiana Cottin, Evelina Juodiene, Domantas Juodis, Biljana Petrovic, Oleksandra Solodarenko, Johannes Binder, Robert Grützmann, Danilo Hackner, Raymund E. Horch, Stefanie Junker, Mani Arsalan, Severine Banek, Felix Chun, Sara Fatima Faqar‐Uz‐Zaman, Daniel Keese, Luis Kluth, Udo Rolle, Andrea Schmedding, Andreas Schnitzbauer, Arnaud Van Linden, Thomas Walther, Jörg Bayer, Jürgen Beck, Helge Eberbach, Christian Fung, Luisa Mona Kraus, Christian Leiber, Nicolas Neidert, Richard Sandkamp, Daniel Schlager, Oliver Schnell, Antonia Schulte, Jakob Strähle, Stefanie Jarmusch, Helmut Franz Georg Novotny, Fritz Spelsberg, Johannes Becker, Christian Fulghum, Bernhard Gonschor, Marit Herbolzheimer, Lena Keppler, Sina Nicolaiciuc, Alexander Trulson, Holger Vogelsang, Benno Zimmermann, Amir Ali Akbari, Andreas Boening, Fabian Edinger, Andreas Hecker, Matthias Hecker, Michael Knitschke, Christian Koch, Martin Reichert, Michael Sander, Götz Schmidt, Emmanuel Schneck, Eberhard Uhl, Silvia Flachs Nóbrega, Philipp Kauffmann, Clemens Miller, Marcus Nemeth, Denise Sievers, Susanne Wolfer, Ulrich Kisser, Jorg Kleeff, Johannes Klose, Kerstin Lorenz, Nancy Papendick, Stefan Plontke, Ulrich Ronellenfitsch, Ingmar Seiwerth, Susanne Steer, Christoph Thomssen, Jörg Ukkat, Christian W. Dumpies, Isabel Fischer, Michael Gessner, John Hanke, Friederike Klauke, Sebastian Leuschner, Thomas Mendel, Katharina Müller, Birte Schmidt, Valentin Schreiter, Peter Stosberg, Franziska Vinz, Beate Herbig, Johannes Sander, Thilo Maria Schulte, Christian Stephan Betz, Julian Bewarder, Johannes Bier, Arne Böttcher, Simon Burg, Chia‐Jung Busch, Lara Bußmann, Martin Gosau, Annika Heuer, Jakob Izbicki, Till Orla Klatte, Daniela König, Leon‐Gordian Köpke, Nikolaus Moeckelmann, Christine Nitschke, Mark Praetorius, Matthias Priemel, Rupert Stadlhofer, Martin Stangenberg, Faik G. Uzunoglu, L ukas Wittig, Henrike Zech, Nina Zeller, Christian Peiper, Frederic Roux, Tsiona Spaeth, Roland Fricker, Thomas Müller, Lars Schröder, Mohammed Alasmari, Clara Boeker, Ibrahim Hakami, Ibrahim Abdullah Hakami, Julian W Mall, Ioannis Kyritsis, Stefan Welter, Christian Graeb, Kristin Huber‐Strößner, Karine Nikolaieva, Daniela Branzan, Markus Doss, Ines Gockel, Carolin Jödicke, Georg Osterhoff, Christina Pempe, Andreas Roth, Robert Sucher, Tina Adler, Kathrin Kelly, Judith Lindert, Janica Merkle, Julia Siebert, Christoph Hirche, Ulrich Kneser, Christian Tapking, Rachit Agrawal, Konstantinos Gousias, Homeira Qureischie, Roland Croner, Lisa Koslowski, Hardy Krause, Frank Meyer, Anke Rissmann, Salmai Turial, Bilal Al‐Nawas, Marco Johannes Battista, Jan Goedeke, Annette Hasenburg, Julia Heider, Valerie Catherine Linz, Lena Katharina Mueller, Simon Zeller, Sina Louisa Patrizia Jentschura, Karl‐Friedrich Kowalewski, Maximilian Kriegmair, David Männle, Nuh Rahbari, Marie‐Claire Rassweiler‐Seyfried, Christoph Reissfelder, Nicole Rotter, Claudia Scherl, Steffen Seyfried, Andreas Kirschniak, Jens Rolinger, Peter Wilhelm, Franz G. Bader, Anna Eleonora Gut, Stephanie Ottl, Alexandra Viktoria Behr, Alexandros Diamantis, Andreas Fichter, Jens Gempt, Florian Grill, Matthias Heck, Daniel Jira, Michael Kallmayer, Florestan Koll, Stefan Luhne, Bernhard Meyer, Robert Patachia, Ilaria Pergolini, Daniel Reim, Seyer Safi, Christoph Schäffer, Moritz Schirren, Arthur Wagner, Helmut Wegmann, Markus Wirth, Zhaojun Zhu, Ughur Aghamaliyev, Markus Albertsmeier, Mahmoud Almaghrabi, Wolfgang Böcker, Jan Bruder, Konstantin Frank, Viktoria Herterich, Verena Huber, Matthias Ilmer, Christian Kammerlander, Alexander M. Keppler, Carl Neuerburg, Viktor H. von Ehrlich‐Treuenstätt, Jens Werner, Nikolaus Börner, Florian Fegg, Daniela Hartmann, Roland Ladurner, Paris Liokatis, Kathrin Patzer, Justin Gabriel Schlager, Wenko Smolka, Petra Zimmermann, Rudolf Hatz, Diana Steinhart, Mircea Gabriel Stoleriu, Claudio Glowalla, Tim Saier, Dorien Schneidmueller, Karl Wilhelm Henkel, Josef Stadler, Martin Steiner, Katharina Hölz, Julia Christina Kaiser, Christian Knorr, Stefan M. Brunner, Britta Kuehlmann, Kyriakos Oikonomou, Karin Pfister, Lukas Prantl, Roland Flurschütz, Jonas Herzberg, Human Honarpisheh, Marie Kröger, Dominic Lepiorz, Charlotte Luths, Andreas Niemeier, Yara Sras, Tim Strate, Thore Winter, Kai Nowak, Tobias Reinhard, Thomas Freiman, Florian Gessler, Sae‐Yeon Won, Gregor A. Stavrou, Rizky Widyaningsih, Sebastian Hoffmann, Ruth Christine Schäfer, Johannes Tobias Thiel, Elisa Bertolani, Alfred Königsrainer, Christian Konrads, Markus W. Löffler, Markus Quante, Christoph Steidle, Lisa überrück, Can Yurttas, Patrick Ziegler, Alexander Zimmermann, Christian Bolenz, Davut Dayan, Jens Greve, Thomas K. Hoffmann, Wolfgang Janni, Simon Laban, Niklas Löbig, Fabienne Schochter, Julius Malte Vahl, Felix Wezel, Veronika Greif, Silke von Pusch, Stefan Schmidbauer, Markus Hirschburger, Imke Marsch, Rolf Schneider, Lars Boenicke, Stephan Degener, Johannes Doerner, Nici Markus Dreger, Franz Christian Horstmeier, Jakob Kruschwitz, Adrian Rombach, Nele Schmidt, Rose Seiberth, Jaswinder Singh, Marieke Smit, Friedrich‐Carl von Rundstedt, Hubert Zirngibl, Joachim Diessner, Sabine Friedrich, Christoph‐Thomas Germer, Philipp Helmer, Johannes Herrmann, Peter Kranke, Hubert Kübler, Johan Lock, Christopher Lotz, Rainer Meffert, Patrick Meybohm, Sophie Müller, Quirin Notz, Maria Popp, Nicolas Schlegel, Tobias Schlesinger, Benedikt Schmid, Magdalena Sitter, Andreas Steinisch, Agnes Treutlein, Anne van den Berg, Armin Wiegering, Thomas Erik Wurmb, Nana Kwaku Agyeman‐Duah, Enoch Appiah, Ralph Armah, Christopher Asare, Lawrence Awere‐Kyere, Dennis Daary, Delali Gakpetor, Stephen Minlah Allah, Ambe Obbeng, Dorcas Osei‐Poku, Diana Puozaa, Enoch Tackie, Nii Armah Adu‐Aryee, Nelson Agboadoh, Offei Asare, Antoinette Bediako Bowan, George Darko Brown, Joe‐Nat Clegg‐Lamptey, Florence Dedey, Cedric Dery, Benjamin Sena Fenu, Marian Abedua Harrison, Philemon Kumassah, Ekins Kuuzie, Josephine Nsaful, David Olatayo Olayiwola, Cecilia Smith, Charles Banka, Romeo Hussey, Diallo Abdoul Azize, Luke Adagrah Aniakwo, Yvonne Adofo‐Asamoah, Evans Kofi Agbeno, Meshach Manu Agyapong, Thomas Agyen, Kwasi Agyen Mensah, Baba Alhaji Bin Alhassan, Mabel Amoako‐Boateng, Peter Appiah‐Thompson, Nita Asamoa‐Manu Gyimah, Moses Asante‐Bremang, Alvin Asante‐Asamani, Henry Atawurah, Anthony Baffour Appiah, Richard Ogirma Baidoo, Ebikela Ivie Baidoo, Benedict Boakye, Abigail Boateng, Dora Dadoe, Makafui Seth Caleb‐Joshua Kwasi Dayie, Samuel Debrah, Kingsley Doku, Enti Enti, Sebastian Ken‐Amoah, Patience Koggoh, Richard Kpangkpari, Patrick Maison, Samuel Mensah, Philip Mensah, Teresa Aba Mensah, Martin Tangnaa Morna, Jilac Nimako‐Mensah, John Nkrumah, Michael Nortey, Emmaunel Owusu Ofori, Isabella Naa Morkor Opandoh, Ethel Osei‐Tutu, Jefferson Owusu Adae, Kofi Quansah, Elizabeth Quartson, Ganiyu Adebisi Rahman, David Walawah, Makafui Yigah, Safia Yussif, Nuna Jiagge, Emmanuel Nachelleh, Jane Acquaye, Kwabena Agbedinu, Fareeda Agyei, Akosua Agyemang‐Prempeh, Kwabena Amo‐Antwi, Michael Amoah, George Amoah, Yaw A Amoako, Daniel Gyawu Aning, Frank Ankobea‐Kokroe, Dominic Annor Mintah, David Anyitey‐Kokor, Adu Appiah‐kubi, Joshua Arthur, Vincent Ativor, Isaac Barnor, Yasmine Braimah, Regina Darko‐Asante, Anthony Davor, Mohammed Duah Issahalq, Mawutor Dzogbefia, Tano Emile, Papa Fiifi‐Yankson, Valerie Gaveh, Senyo Gudugbe, Solomon Gyabaah, Frank Enoch Gyamfi, Adam Gyedu, Derrick Gyimah, Bernard Hammond, Boakye‐Yiadom Jonathan, Yorke Joseph, Thomas Okpoti Konney, Anna Konney, Kwasi Kusi, Ishmael Kyei, Agbenya Lovi, Nuhu Naabo, Boateng Nimako, Beauty Nyadu, Obed Ofori Nyarko, Ben Blay Ofosu‐Barko, Philip Peprah Oppong, Anita Osabutey, Martha Poku, Robert Sagoe, Abiboye Yifieyeh, George Ansong, Adam Abass, Alhassan Abdul‐Mumin, Theophilus Adjeso, John Abanga Alatiiga, Munira Amadu, Nathaniel Annan, German Azahares Leal, Mohammed Bukari, Alexis Buunaaim, Ernest Cheyuo, Latif Daboo Salifu, Michael Damah, Malcolm Dery, Edem Kojo Dzantor, Odoniel Guerra Garcia, Yabasin Iddrisu Baba, Adamu Issaka, Abdul‐Jalilu Mohammed Muntaka, James Murphy, Yaa Nyarko Agyeman, Wisdom Opoku Amankwaa, Imoro Osman, Samuel Pie, Anwar Sadat Seidu, Mohammed Sheriff, Ana Maria Simono Charadan, Stephen Tabiri, Abraham Titigah, Mundashiru Yahaya, Musah Yakubu, Edwin Mwintiereh Ta‐ang Yenli, Ioannis Grypiotis, Nikolaos Kiriakopoulos, Georgios Koliopoulos, Vasilis Kyvelos, Georgia Micha, Triada Papadopoulou, Dimitrios Balalis, Evangelos Fradelos, Dimitrios Korkolis, Nicholas Alexakis, Kyveli Angelou, Dimitrios Haidopoulos, Anastasia Prodromidou, Alexandros Rodolakis, Nikolaos Thomakos, Dimitris Psychogios, Pantelis Antonakis, Konstantinos Bramis, Leonidas Chardalias, Ioannis Contis, Nikos Dafnios, Dionysios Dellaportas, Papalouka Dimitra, Georgios Fragkoulidis, Georgios Gkiokas, Antonios Gklavas, Theodoros Hadjizacharias, Dimitra Karageorgou, Manousos Konstadoulakis, Christina Kontopoulou, Dimitrios Massaras, Nikolaos Memos, Ioannis Papaconstantinou, Dimitrios Politis, Andreas Polydorou, Konstantinos Stamatis, Theodosios Theodosopoulos, Antonios Vezakis, Konstantinos Avgerinos, Jevgeni Katunin, Aristotelis Kechagias, Dionysia Kelgiorgi, Neoklis Kritikos, Pasi Pengermä, Theodosios Bisdas, Argyrios Ioannidis, Michael Konstantinidis, Sofia Konstantinidou, Nikolaos Patelis, Maria Ioanna Antonopoulou, Eirini Deskou, Vasileios Kalles, Dimitrios K. Manatakis, Nikolaos Stamos, Nikolaos Tasis, Nikolaos Arkadopoulos, Nikolaos Danias, Panagiota Economopoulou, Maximos Frountzas, Panagiotis Kokoropoulos, Nikolaos Michalopoulos, Jonida Selmani, Theodoros Sidiropoulos, Panteleimon Vassiliu, Kosmas I. Paraskevas, Dimitrios Bartziotas, Konstantinos Bouchagier, Ilias Galanis, Theodosis Kalamatianos, Stylianos Kapiris, Angeliki Kolinioti, Eleni Mavrodimitraki, Panagiotis Metaxas, Alexandrina Nikova, Michail Psarologos, Maria Sotiropoulou, George Stranjalis, Konstantinos Albanopoulos, Panagiotis Kondilis, Gavriella Zoi Vrakopoulou, Aristeidis Chrysovergis, Georgios Chrysovitsiotis, Evangelos Giotakis, Vasiliki Kanellopoulou, Efthymios Kyrodimos, Andreas Larentzakis, Pavlos Pantos, Vasileios Papanikolaou, Spyridon Potamianos, Charalampos Theodoropoulos, Alexandra Triantafyllou, Tania Triantafyllou, Kleoniki Georgousi, Peter Panagiotou, Ekaterini Christina Tampaki, Emmanouil Avramidis, George Babis, Evangelos Zafeiris, Afroditi Antoniou, Nikolaos Bessias, Dimitris Maras, Theofanis Papas, Konstantinos Roditis, Ioannis Tsagkos, Paraskevi Tsiantoula, Andreas Alexandrou, Efstratia Baili, Alexandros Charalabopoulos, Dimitrios Dimitroulis, Panagiotis Dorovinis, Zoe Garoufalia, Prodromos Kanavidis, Ioannis Karavokyros, Lysandros Karydakis, Stylianos Kykalos, Eleandros Kyros, Nikolaos Machairas, Aikaterini Mastoraki, Nikolaos Nikiteas, Alexandros Papalampros, Dimitrios Schizas, Antonia Skotsimara, Paraskevas Stamopoulos, Athanasios Syllaios, Alexis Terras, Nefeli Tomara, Gerasimos Tsourouflis, Ilias Vagios, Constantinos Zografos, Konstantinos Apostolou, Nikolaos Georgopoulos, Theofani Antoniou, Christina Antzaka, Areti Falara, Socrates Fragoulis, Panagiotis Ftikos, Fedra Matsouka, Konstantinos Perreas, Panagiota Rellia, Evangelia Samara, Anna Smirli, Androniki Tasouli, Apostolos Thanopoulos, Petros Loukas Chalkias, Georgia Dedemadi, Panagiotis Mourmouris, Andreas Skolarikos, Nikoletta Theochari, Lazaros Tzelves, Stylianos Gaitanakis, Theodoros Milas, Emmanuel Theodorakis, Paraskevi Karona, Pagona Kastanaki, Angelos Tzouganakis, Christos Agalianos, Ioannis Tsouknidas, Andreas Xenakis, Emmanuel Chrysos, Konstantinos Lasithiotakis, Taxiarchis Nikolouzakis, Sofia Xenaki, Evangelos Xynos, Eftychios Lostoridis, Eleni‐Aikaterini Nagorni, Antonio Pujante, Paraskevi Tourountzi, Aggeliki Al, Kyriaki Baxevanidou, Konstantinos Bouliaris, Matheos Efthimiou, Christos Kalfountzos, Georgios Koukoulis, Vasileios Lachanas, Konstantinos Petropoulos, Ioannis Tsitiridis, Fragkiskos Angelis, Eleni Arnaoutoglou, Ioannis Baloyiannis, Metaxia Bareka, Anna Bouronikou, Gregory Christodoulidis, Alexandros Daponte, Maria Fergadi, Nick Gkolias, Eleni Gkrinia, Jiannis Hajiioannou, Nikos Kalogritsas, Eleni Karoni, Christos Korais, Giorgos Krestinidis, Antigoni Ktisti, Dimitrios Magouliotis, Charikleia Maiou, Konstantinos Malizos, Maria Minasidou, Maria Ntalouka, Anna Maria Ntziovara, Effrosyni Palla, Konstantinos Perivoliotis, Fani Saini, Athina Samara, Athanasios Saratziotis, Charalampos Skoulakis, Efthymios Solomi, Konstantinos Stamoulis, Christos Dimitrios Terzoudis, Evangelia Tsironi, George Tzovaras, Kyriaki Vallianou, Dimitris Zacharoulis, Anna Ziogkou, Κωνσταντί__ς Δακής, Liolis Elias, Ioannis Maroulis, Francesk Mulita, Kerasia‐Maria Plachouri, Michail Vailas, Charalampos Doitsidis, Eva Filo, Ioanna Gkalonaki, Eleni Kogia, Konstantina Kontopoulou, Magdalini Mitroudi, Christina Panteli, Ioannis Patoulias, Olga Ioulia Semkoglou, Dimitrios Sfoungaris, Ioannis Valioulis, Γι ωργος __υτσ_υ μης, Ioannis Astreidis, Panagiotis Christidis, Orestis Ioannidis, Lydia Loutzidou, Antonis Mantevas, Konstantinos Paraskevopoulos, Dimitris Tatsis, Apostolos Athanasiadis, Themistoklis Dagklis, Ioannis Kalogiannidis, Georgios Kapetanios, Apostolos Mamopoulos, Chrysoula Margioula‐Siarkou, Stamatios Petousis, Ioannis Tsakiridis, Christos Anthoulakis, Chrysanthos Christou, Antonios Fantakis, Eirini Iordanidou, Christos Kaselas, Sousana Panagiotidou, Vasileios Papadopoulos, Kyriakos Papavasiliou, Athanasios Piachas, Ioannis Siasios, Ioannis Spyridakis, Theodoros Theodoridis, Andreas Tooulias, Eleftherios Tsiridis, Maria Tsopozidi, Georgios Tsoulfas, María Alemán, Estuardo Brolo, Alitza Gutiérrez Ruiz, Ana Lucia Lemus, Jennifer Greenberg, Krisna Mishel Morales Chew, José Rodrigo Oliva, Alejandra Rodas, María Alejandra De León Lima, Ismar Lopez Muralles, Ana Lucía Portilla, Gustavo Recinos, Felipe Solares, Maria‐Lorena Aguilera‐Arevalo, Gaby Ajcip, Claudia Anton, Jacqueline Carrera, Jose Cojulun, Mario‐Andrés Flores, Noriega José, Carlos Adolfo Marroquín Paiz, Steffanía Morales, Ramiro Najera, Eduardo Quiñónez Lorenzana, Pablo Rivera, Lesly Rodas, Victor Santos, Dianne Sosa, Natalia Ybarra, Amalia Barrios Duarte, José David Pérez Cajti, Carlos Régil, Walter A Osorio, Rember Rosales Arriola, Luis‐Fernando Talé‐Rosales, Danilo Herrera, Servio Tulio Torres Rodríguez, Sergio Alejandro Villeda, Joshua Anicetti, Megan Lowey, Andrea Michelle Lowey Medina, Javier Ardebol, Kathia Barillas, Sabrina Barillas, Francis De Leon, Salvador Recinos, Miguel‐Angel Marroquin‐Alpirez, Yessica Yax, Sophie Hon, Yuk Ho Liu, Alex Qinyang Liu, Shirley Liu, Hei Tung Natalie Chiu, Chi Man Tom Chow, Victor Hau, Ho Wai Ip, Brian Mak, Chung Ying Mok, Dennis Ng, Yin Yu Eva Siu, Kiu Fung Wong, Jingya Jane Pu, Yu‐xiong Su, Kit Ying Au‐Yeung, David Yuen Chung Chan, Albert Chan, Shannon Melissa Chan, Tsz Ching Chang, Tor Wo Chiu, Wang Kei Chiu, Kaori Futaba, Zhexi He, Man Fung Ho, Kevin Ki Wai Ho, Jacky Yan Kit Ho, Janet Wui Cheung Kung, Cheuk Ho Lam, Rainbow W.H. Lau, Samuel Ka Kin Ling, Hon Ting Lok, Tony Wing Chung Mak, Chi‐Fai Ng, Simon Ng, Kelvin Kwok‐Chai Ng, Calvin S.H. Ng, Michael Tim Yun Ong, Teresa Tan, Sui Fan Tang, Jeremy Yuen‐Chun Teoh, Anthony Teoh, Bess Siu Yan Tsui, George Kwok Chu Wong, Randolph Wong, Kwok Chuen Wong, Chi Hang Yee, Zsuzsanna Antal, Attila Kalman, Peter Voros, Kiarash Bahrehmand, Timea Echim, Tamás Mersich, Zoltan Novak, Tamás Sztipits, Dániel Wettstein, Laszló ádám Bihari, László Hidi, Laszlo Piros, Balazs Rózsa, Peter Sotonyi, Lilla Szatai, György Herczeg, Bálint Pordány, Fanni Tornyi, Kristof Dede, Tamás Egyed, György Saftics, Judit Kulcsicka‐Gut, Zsófia Sipos, Dezso Toth, Bhavin Patel, Dhaivat Vaishnav, Raghunandan Gorantlu Chowdappa, Hardil Majmudar, Saptak Mankad, Sohilkhan Pathan, L Sharathkumarkl, Irappa Madabhavi, Lokesh Sasatti, Dhananjaya Bhat, Subramanyam Mahankali, Santhosh Kumar Sampengere Annayappa, Tulika Agrawal, Premkumar Anandan, Aditya Baindur, Manjunath Bd, C Savitha, G S Anitha, Sandeep Harigond, K K Tejeswini, Venkatesh Kesarla, Sunil Kumar Venkatappa, Mallikarjuna Manangi, P B Hareesh, Jyothi K R Ranjan, Athish Shetty, Santhosh Shivashankar Chikkanayakanahalli, Tanvi Sunil, Shreya Syamala, Kavya Tharanath, Aditya Atal, Niranjana Rajagopal, Sumit Thakar, B S Srinath, Kavitha Jain, Vinod Nk, P L Thirumanikandan, Aruna Kumar, Nitu Mishra, Sushruta Shrivastava, Rekha Wadhwani, Reyaz Ahmad, Zainab Ahmad Haq, Prateek Behera, Ritika Dhurwe, Rehan Haq, Vaibhav Jain, Anuj Jain, Sunaina Karna, Manoj Nagar, Kameshwarachari Pushpalatha, Sumit Raj, John Ashutosh Santoshi, Pooja Singh, Virendra Verma, Vaishali Waindeskar, Moorat Singh Yadav, Zaheda Aziz, Koyel Chakraborty, Preetam Chappity, Gurudip Das, Debajyoti Datta, Saubhagya Kumar Jena, Madhabananda Kar, Susanta Khuntia, Pankaj Kumar, Ravi Kumar, Aswathi Kv, Abhijeet Mishra, Tushar Mishra, Swastik Mishra, Yash Mittal, Dillip Muduly, Ritesh Panda, Sibasish Panigrahi, Sucheta Parija, Saroj Patra, Bikram Rout, Rabi Sahu, Saurav Sarkar, Arunkumar Sekar, Sweta Singh, Sanjibani Sudha, Mahesh Sultania, Sujit Tripathy, Paulson Varghese, Anand Gupta, Rajeev Kansay, Robin Kaushik, Simrandeep Singh, Sunil Kumar Gupta, Lileswar Kaman, Madhivanan Karthigeyan, Siddhant Khare, Vishal Kumar, Sandeep Mohindra, Ninad Patil, Pravin Salunke, Ajay Savlania, Kavindra Singh, Manjul Tripathi, RV Pradeep Krishna, Gomathy Narasimhan, Ashwin Rammohan, Mohamed Rela, Akhila Appukuttan, Swati Goudar, Shweta Mallick, Sudheer Othiyil Vayoth, Anupama Rajanbabu, Christi Titus, Ezhir Selvan Chidambarasamy, Devdas Madhavan, Anandan Murugesan, Kuppurajan Narayanasamy, P B Barani Kumar, Firoz Rajan, Anbukkani Subbian, Paari Vijayaragavan, Rahul Gupta, Arvind Kumar, Biplob Borthakur, Tapan Singh Chauhan, Akhil Govil, Shubhra Gupta, Vijay Mohan Hanjoora, Monish Raut, Ashish Sharma, Aseem Srivastava, Saurabh Tiwari, Vartika Vishwani, Nissi Evelyn, Karuna Sree Pendyala, Navakoti Prasad, Jyoti Bothra, Mainak Deb, Koushik Herle, Harish Jayaram, Lavanya Kannaiyan, Abirami Krithiga, W Mukta, Anil Matai, Pooja Nagpal, Prachi Pathak, Sumit Banerjee, Ramkaran Chaudhary, Gautam Ram Choudhary, Ankita Chugh, Pawan Dixit, Abhay Elhence, Nitesh Gahlot, Mayank Garg, Navdeep Kaur Ghuman, Deepak Jha, Prakash Kala, Amanjot Kaur, Vijay Madduri, Sanjeev Misra, Himanshu Pandey, Puneet Pareek, Manish Pathak, Kirtikumar J Rathod, Mahaveer Singh Rodha, Rahul Saxena, Naveen Sharma, Shashank Shekhar, Mahendra Singh, Pratibha Singh, Bhaskar Suryanarayanan, Jeewan Ram Vishnoi, Shivang Amin, Vishal Bhende, Rohit Kumar, Tanishq Sharma, Kesavan Murugesan, Sathish Muthu, Abhinav Balachandar Subbiah Ramasamy, Debarshi Chatterjee, C Gerber Gerber, Indranil Ghosh, Upasana Naskar, Gaurav Aggarwal, Sanjit Kumar Agrawal, Azaz Ahmed, Sujoy Gupta, Prateek Jain, Deepak Jain, Vishal Kewlani, Amrit Pipara, Noopur Priya, Roopak Raja, Sudip Shakya, Abhishek Sharma, Robin Thambudorai, Dimple Kharkongor, Anjoo Agarwal, Naseem Akhtar, Akshay Anand, Mona Asnani, Ankur Bajaj, Arun Chaturvedi, Akhilanand Chaurasia, Loreno E. Enny, Surabhi Garg, Sameer Gupta, Anoop Kumar Jaiswal, Somil Jaiswal, Yashpal Jaware, Navneet Kala, Ruchi Karnatak, Apjit Kaur, Vijay Kumar, Manoj Kumar, Ambrish Kumar, Upander Kumar, Amit Kumar Shrivastava, Seema Mehrotra, Brijesh Mishra, Anand Mishra, Namrata, Bal Krishna Ojha, Ahmad Ozair, Uma Shanker Pal, Amita Pandey, Nancy Raja, Shiv Rajan, Pooja Ramakant, Ashutosh Roy, Rekha Sachan, Satyanarayan Sankhwar, Pushp Sankhwar, Divya Sarin, Ayushi Shukla, Sushil Singh, Mohit Singh, Kul Ranjan Singh, Renu Singh, Uma Singh, Urmila Singh, Vandana Solanki, Abhinav Arun Sonkar, Chhitij Srivastava, Parijat Suryawanshi, Vivek Tewarson, Rajat Verma, Manju Lata Verma, Awdhesh Yadav, Gaurav Agarwal, Gyan Chand, K.M.M. Vishvak Chanthar, Dileep Hoysal, Anjali Mishra, Nitin Batra, Arun Bhatti, Rupali Chopra, Shakina David, Tapasya Dhar, Uma Kant Dutt, Rohini Dutta, Sumir Gandhi, Parvez David Haque, Ritu Jain, B Paul Sudhakar John, Sreejith Kannummal Veetil, Gurvinder Kaur, Navneet Kumar, Anil Luther, Anupam Mahajan, Amit Mahajan, Kavita Mandrelle, Shefin Mathews, Vishal Michael, Partho Mukherjee, Pinki Pargal, Rajesh Paul, Pranay Pawar, Anupam Phillip, Rachel Phillips, Abhishek Samuel, Noel Singh, Inderjot Singh, Abhijit Singh, Sarvpreet Singh Grewal, Anusha Singhania, Selven Thirumalai, Ashish Varghese, Joshua Wesley, Philip Alexander, Josy Thomas, Pradeep Zechariah, Mariam Anjum Ifthikar, Rohan Thomas Mathew, Rohan Shetty, M Vijayakumar Vijaykumar, BM Zeeshan Hameed, Sufyan Ibrahim, Gayathri Jyothish, Sunil Krishna, L Badareesh, Stanley Mathew, Arjun Suresh Kumar, Navneet Kumar Chaudhry, Narinder Singh, Piyush Kumar Sinha, Rachith Sridhar, Sunny Agarwal, Srikant Balasubramaniam, Lipika Baliarsing, Swati Chhatrapati, Charulata Deshpande, Satish Dharap, Ashni Dharia, Sarita Fernandes, Suraj Gandhi, Mangesh Gore, Abhilash Jayakumar, Rameshwar Mhamane, Anand Nirgude, Sandesh Parab, Manish Patil, Amit Peswani, Anjana Sahu, Sarika Samel, Fagun Shah, Dipti Haridas, Amruta Kulkarni, Vijay Shetty, Bejoy Abraham, Varun Agarwal, Quazi Ahmad, Ashishkumar Asari, Mohammad Ismail Attar, Nagaraja Sekhar Ayyalasomayajula, Sutej Bachawat, Shivani Bachhav, Vivek Badhe, Sanjiv Badhwar, Shubhabrata Banerjee, VIKAS Basa, Vipul Bothara, Somnath Chattopadhyay, Sohin Chaudhari, Rahul Chavan, Priyank Chawathe, Shailja Dadhich, Anuj Dalal, Avinash Date, Mandar Deshpande, Preetham Dev, Niren Dongre, Anirudha Doshi, Maya Gade, Shreyash Gajjar, Mohan Gawande, Amol Ghalme, Bhavisha Ghugare, Ishita Gupta, Manoj Jain, Divakar Jain, Saumya Sekhar Jenasamant, Vaishali Joshi, Vinay Joshi, Neha Kalwadia, Nandkishore Kapadia, Hari Bipin Radhakrishnan Kattana, Tirathram Kaushik, Akshat Kayal, Shama Kovale, Yogesh Kulkarni, Abhaya Kumar, Kranthi Kumar, Kashmira Kumawat, Vidyadhar Lad, Namrata Maskara, Rajesh Mistry, Smruti Ranjan Mohanty, Kanchan Motwani, Manoj Mulchandani, Mandar Nadkarni, Sanjay Pandey, Mrunal Parab, Dinshaw Pardiwala, Amrita Patkar, Neha Pawar, Abhijit Pawar, Abhinav Pednekar, Vishal Peshattiwar, Harshwardhan Pokharkar, Ojas Potdar, Amit Pothare, Faizan Rahmani, Sunil Rajput, Nalla Ramji Narendra, Anuradha Rao, Suresh Rao, Himanshu Rohela, Rajendra Sakhrekar, Dhanshree Salunkhe, Gursev Sandlas, Hrishikesh Sarkar, Afroz Satpathy, Raghuram Sekhar, Yashwant Shelke, Sanket Sadanand Shetty, Shweta Shetye, Anshumala Shukla Kulkarni, Umang Singal, Faisal Solanki, Rajendra Sonawane, Raghavendraswami Thete, Yuvaraja Thyavihally, Raj Vhatkar, Sameer Vora, Santosh Waigankar, Shruti Wasnik, Mona Yadav, Rammohan Yedave, Hriday Acharya, Anant Bangar, Manoj Bharucha, Parag Dhumane, Santosh Karmarkar, Rajesh Nathani, Archana Nehe, Abhay Nene, Webster Jerry Noronha, Naresh Palapalle, Priyank Patel, Dilroop Poyyil, Rajeev Redkar, Munjal Shah, Rahul Deo Sharma, Shruti Tewari, Ganesh Bakshi, Vikram Chaudhari, Anuja Deshmukh, Ashwin Desoouza, Stuti Gupta, Deepa Nair, Prakash Nayak, Shraddha Patkar, C S Pramesh, Ajay Puri, Sajid Qureshi, Prakash Shetty, Ts Shylasree, Purvi Thakkar, Shivakumar Thiagarajan, Virendra Kumar Tiwari, Saiesh Reddy Voppuru, Rajesh Soni, Anushri Soni, Gira Soni, Junaid Alam, Dinesh Bagaria, Minu Bajpai, Akshay Kumar Bisoi, Arvind Chaturvedi, Sandeep Chauhan, Narendra Choudhary, Rajendra Singh Chouhan, Sunil Chumber, Surya Kumar Dube, Kamran Farooque, Vasubabu Gudala, Amit Gupta, Mohit Joshi, Apoorva Kabra, Shashank Sharad Kale, Abhinav Kumar, Subodh Kumar, Dhruv Mahajan, Rajesh Malhotra, P Ramesh Menon, Biplap Misra, Samarth Mittal, Rajinder Parshad, Pratyusha Priyadarshini, Sushma Sagar, Pradeep Brijkishor Sharma, Shilpa Sharma, Vijay Sharma, Vivek Trikha, Mayank Tyagi, Kuldeep Bansal, Harvinder Singh, Kalyan Varma, Lovenish Bains, Anurag Mishra, Rajdeep Singh, Rohit Bhardwaj, Abhishek Mittal, Sabarirajan Ponnusamy, Gyan Ranjan Singh, Isha Tuli, Pramod Bhor, Sanjay Dhar, Rahul Ghodke, Sachin Kale, Manisha Aggarwal, Himani Gupta, Gurleen Kaur, Ashwani Kumar, Ajinkya Deshpande, Anup Gadekar, Tanmay Jaysingani, Taufiq Panjwani, Rakesh Patil, Vivek Sodhai, Narendra Vaidya, Sunil Kumar Vishwakarma, Utkarsha Wayal, Debnarayan Dutta, Suvendu Maji, Rajnish Arora, Somprakas Basu, Mohit Dhingra, Pankaj Kumar Garg, Amit Gupta, Farhanul Huda, Pankaj Kandwal, Ravi Kant, Rajkumar Kottayasamy Seenivasagam, Navin Kumar, Shashank Kumar, Lokavarapu Manoj Joshua, Radheyshyam Mittal, Dharma Ram Poonia, Deepak Rajput, Saravanan Sadhasivam, Sudhir Singh, Vivek Singh, Kshitija Chandanwale, Chandrashekhar Mahakalkar, Melissa Philip, Vaibhav Thorat, Yousuf Choudhury, Devishmita Das, Mautushi Das, Subhadra Goala, Ravi Kannan, Farhana Yasmin Laskar, Parbin Laskar, Kapil Malik, Poulome Mukherjee, Abhishek Sarkar, S Thoibisana Singha, M Nongalei Singha, Damayanti Singha, Gowtham Srungavarapu, Ritesh Tapkire, Siempui Tling, Syed Muzamil Ishaq Andrabi, Gowhar Aziz Bhat, Nisar Chowdri, Robindera Kour, Asif Mehraj, Fazl Parray, Raahil Shah, ZAMIR AHMAD Shah, Rauf Wani, Soujanya Adamala, Ravikanth Gowder, Siddhi Hegde, M S Mashitha, N Ranganath, Shreya Sreeram, Sushil Ankadavar, Darshan Bafna, Bharat Dhanani, Nikhil Ingle, Piyush Jadhao, Sinu Joseph, Swapnil Kapote, Shirin Karkada, Vijay Kumar, Parag Lad, Rajan Lohia, Suyog Madje, Venkateshwaran Narasiman, Ashish Phadnis, Meer Chisthi, Gejoe George, I Yadev, Harihara Jothi, Alfie Kavalakat, Dilber Pareed, Godwin David C. Mathew, Livingston Abel, Mansi Agrawal, B Rabindranath, Manish Baldia, Ravi Kishore Barla, Manisha Beck, Santosh Benjamin, Jeremy Bliss, Lisa Cherian, Sreekar Devarakonda, Geley Ete, Arun Jacob Philip George, Amish Gohil, Deeptiman James, Mark Ranjan Jesudason, Lallu Joseph, Treasa Joseph, Kathir Joyson, Gomathi Karnan, Albert Kota, Pushplata Kumari, Anitha Loganathan, Vasanth Mark Samuel, John Mathew, Rohin Mittal, Senthil K Nathan, Joby Elizabeth Ninan, Ajay Philip, R. Priyadarshini Priyadarshini, S Suganya, Habie Samuel, Gilbert Samuel, Daniel Selvaraj, Srujan Sharma, Suraj Surendran, T D Hariharan, Santhosh Kumar Thangaraj, Vinotha Thomas, Varghese Thomas, John K Thomas, Y S Harish, Manobhiram Boggavarapu, Karthik Chandra Vallam, Murali Krishna Voonna, Marilaeta Cindryani Lolobali, Christopher Ryalino, Tjokorda Gde Agung Senapathi, Mahadewa Tjokorda, I Made Gede Widnyana, Dita Aditianingsih, Aino Auerkari, Susilo Chandra, Fachreza Aryo Damara, Mohammad Adya Firmansha Dilmy, Achmad Kemal Harzif, Sidharta Kusuma Manggala, Dedy Pratama, Affan Priyambodo, Andi Ade Ramlan, Ratna Farida Soenarto, Adhrie Sugiarto, Ilham Utama Surya, Tamara Tango, Aida Rosita Tantri, Raihanita Zahra, Dedy Fachrian, Andi Hasyim, Ade Susanti, Teddy Saputra, Erwin Syarifuddin, Andi Asadul Islam, Gabriele Kembuan, Hendra Pajan, Ahmad Hannan Amrullah, Khoirul Anam, Thirza Hadipranata, Julius Albert Sugianto, Aidyl Fitrisyah, Mayang Indah Lestari, Nur Rachmat Lubis, Rizal Zainal, Zulkifli Zulkifli, Erick Gamaliel Amba, Warren Lie, Andika Adiputra Thehumury, Tedy Apriawan, Yunus Kuntawi Aji, Roidah Taqiyya Zahra Wathoni, Sumadi Lukman Anwar, Teguh Aryandono, Wirsma Arif Harahap, Juni Kurniawaty, Djayanti Sari, Artanto Wahyono, Yunita Widyastuti, Akhmad Yun Jufan, Parisa Arjmand, Mohammad Etezadpour, Babak Ganjeifar, Masoumeh Hosseinpoor, Hassan Mehrad‐Majd, Mohsen Rajati, Reza Assadi, Ehsan Noori, Parisa Rajaei, Samira Hajisadeghi, Mohammad Mehdizadeh, Mina Soltani, Narges Alizadeh, Gholamreza Azarnia Azar Nia, Hamid Heidari, Seyedmohamad Hosseini Zavareh Hosseini Zavareh, Ali Moazami Pour Moazami Pour, Farokh Savaddar, Mostafa Vahedian, Hoora Amouzegar, Gholamreza Azarnia Azar Nia, Mojdeh Bahadorzadeh, Seyedeh Homa Hemmasi, Ahmad Kachoie, Ali Karimi Karimi, Sepideh Miraj, Monireh Mirzaie, Hossein Mokarami Mokarami, Amrollah Salimi, Roghayyeh Ahangari, Ali Ahmadvand, Ali Bashiri, Morteza Borhani, Mohammad Haidari, Mohammad Taghi Imani Khosroshahi, Shahrokh Jahan Bini, Mohsen Koosha, Mohammad Kazem Moslemi, Ali Naghibi, Shahrzad Tehrani, Zahra Yazdi, Seyyed Hassan Adeli, Majid Alborzi, Hamed Bagheri, Mohsen Eshraghi, Hassan Fatemi Manesh, Mohammad Ghomeisi, Seyed Fakhreddin Hejazi, Ahmad Kachoie, Saeed Madani, Nima Najafian Motahaver, Samieh Norouzi, Mahdi Pezeshki Modarres, Ali Shafiee, Jamshid Vafaeimanesh, Hossein Yusefi, Ahmad Kachoie, Saeed Madani, Elahe Hosseini, Pourya Medhati, Hamed Nikoupour, Kayvan Aghazadeh, Shahin Bastaninejad, Payman Dabirmoghaddam, Reza Erfanian, Mohammadreza Firouzifar, Farrokh Heidari, Shirin Irani, Ebrahim Karimi, Ali Kouhi, Masoud Motasaddi Zarandy, Mahtab Rabbani Anari, Saleh Sandoughdaran, Saeed Sohrabpour, Ardavan Tajdini, Nasrin Yazdani, Hamed Akhavizadegan, Esmaeil Rezghi Maleki, Naser Yousefzadeh Kandevani, Nima Bagheri, Seyed Amir Javadi, Seyed Hadi Kalantar, Farzaneh Keneshlou, Zahid Hussain Khan, SM Javad Mortazavi, Sayedali Ahmadi, Jaber Hatam, Mohammad Hossein Khosravi, Mohammad Hossein Nabian, Leila Oryadi Zanjani, Fardis Vosoughi, Mohammadreza Golbakhsh, Seyyed Hossein Shafiei, Babak Siavashi, Ali Al‐Isawi, Mohammed Al‐Masood, Dania Al‐Najjar, Yarub Gahtan, Sara Nabil, Najat Abdul Hameed, Jumana Abdul Hameed, Abdullah Ahmed, Ahmed Hilmi, Ali Akadh, Rand Hussein, Abbas Aljebur, Sadik Hassan, Haithem Hussein Ali, Fahad Al‐Hasani, Mustafa Wameedh Ibrahim, Mubder Mohammed Saeed, Haidar Muhssein, Success Akindoyin, Hilary Ikele, Catherine McNestry, Stevie Barry, Nikhil Dewan, Bosom Ekwere, Murtaza Essajee, Ream Langhe, Calista Marshall, Darya Musa, Zeeshan Ahmad, Emmet Andrews, Bruno Chan Chin, Mark Corrigan, Amy Edwards Murphy, Christina Fleming, Niamh Foley, Padraig Gardiner, Daniel Hechtl, Michelle Hsiao, Mohd Yasser Kayyal, Shane Killeen, Maria Lyons, Stephen O’Brien, Cathy Burke, Matt Hewitt, Marwa Mohamed, Syeda Farah Nazir, Mei Yee Ng, Nor Azlia Abdul Wahab, Cathy Monteith, Oladayo Oduola, Lylas Aljohmani, Timothy Nugent, Sami Abd Elwahab, Paula Corr, Lauren Crone, Niall Davis, Johnathon Harris, Arnold Hill, Mohsen Javadpour, David Kearney, Robert Anthony Keenan, Deirdre Nolan, James Ryan, Mark Philip Hehir, Brendan McDonnell, Carmen Regan, Michael Geary, Fergal Malone, Claire McCarthy, Clare O Connor, Donal B O’Connor, Jarlath Bolger, Cillian Clancy, Shane Considine, Stefanie M Croghan, Noel Donlon, Emma Donohoe, Caroline Herron, John Larkin, Thomas Hugh Lynch, Barry Maguire, Rustom Manecksha, Andrea Mc Carthy, Katharina Nagassima, Erica O’Sullivan, Pat Rohan, Ryan Roopnarinesingh, Salloum Salloum, Thomas Aherne, Mary Barry, Gareth John Bowen, Ellen Boyle, Carolyn Cullinane, Joseph Dowdall, Ann Hanly, Ahmed Hassanin, Helen Heneghan, Conor Hurson, Orlaith Kelly, Rory Kennelly, Aoife Kiernan, Sean T Martin, Nawar Masarani, Damian McCartan, Enda W McDermott, Ben Murphy, Kin Cheung Ng, Nwabundo Njeze, Aine O’Neill, Ruth S Prichard, Ned Quirke, Ian Sean Reynolds, Des Winter, Rowan Casey, Ben Creavin, Mutaz Elamin, Amy Gillis, Dara Kavanagh, Michael Kelly, áine McNamee, Muheilan Muheilan, Paul Neary, Patrick Owens, Akshaya Ravi, Paul Ridgway, Paul Carroll, Chris Collins, Amenah Dhannoon, Helen Earley, Amy Fowler, Aisling Hogan, Aoife Lowery, Peter McAnena, Charlie Timon, Stewart Walsh, Wisam Al‐Ramli, Imran Azeem, Muhammad Usaama Bahadoor, Zsolt Bodnar, Hassan Elmusharaf, Faisal Saeed Hassan, Adam Hingum, Huilun Huan, Seamus Jennings, Syed Mohammad Umar Kabir, Mohamed Hamed Khalid, Mariya Kuteva, Syed Nadeem Mujtaba, Shanell Peeriyah, Hina Rehman, Muhammad Assam Sarwar, Tony Shaju, Ian Stephens, Michael Sugrue, Joseph Thomas, Manvydas Varzgalis, Saqib Zeeshan, Alisha Jaffer, Larne Jones‐Whiting, Colin Peirce, Sean Johnston, Seantee Lim, éanna Ryan, Alwaleed Abdelgadir, Sara Ahmed, Youssef Al‐Mukhaizeem, Tara Connelly, Fiachra Cooke, Clare Crowley, Ivor Cullen, Michael Flanagan, Amy Fogarty, Orna Glynn, Claudia Guerrero Martinez, Mohamed Alfatih Hamza, Mekki Hassan, Ibrahim Hegazy, Rhodri Hill, Amr Kazim, Azriny Shaziela Khalid, Muhammad Abdullah Khalid, Zubair Majeed, Aidan Manning, Peter McCullough, Seamus Murphy, Peter Neary, Anthony Noone, Gerrard O’Donoghue, John O’Kelly, Eddie Odonnell, Elaf Osman, Jessica Ryan, Rafeh Saeed, Ahmad Abo Arar, Arsan Abu Abed, Daniel Dykman, Ahmad Elnassasra, Inbar Gatot, Haim Gavriel, Ruthie Gold‐Deutch, Nadav Haim, Jonathan Hammerschlag, Yehuda Hershkovitz, Ahmad Jaber, Adi Kenoshi, Ron Lavy, Omar Majadla, Limor Muallem‐Kalmovich, Hilli Nativ, Igor Rabin, Yael Sandler, Oded Zmora, Osnat Zmora, Miklosh Bala, Yonatan Avraham Demma, Yuri Fishman, Gad Marom, Naor Avni, Ofra Carmel, Amicur Farkas, Michael Ron Freund, Lior Gonen, Yaacov Gozal, Dmitry Greenman, Stanislav Kocherov, Nevo Margalit, Orit Nahtomi Shick, Israel Alexander Ostrovsky, Rivka Pardes, Michal Perets, Tal Shahar, Henry Shapiro, James Tankel, David Teren, Reuven Yahud, Shlomo Yellinek, Paolo Balercia, Lisa Catarzi, Giuseppe Consorti, Pasquale Cianci, Domenico Gattulli, Marina Minafra, Enrico Restini, Enrico Andolfi, Filippo Annino, Frezza Barbara, Alessia Biancafarina, Edoardo Bussolin, Marco De Prizio, Ulpjana Gjondedaj, Marilena Gubbiotti, Gianni Mura, Giuseppe Antonino Pellicano, Giacomo Maria Pirola, Rezart Sulce, Gennaro Martines, Vincenzo Papagni, Arcangelo Picciariello, Stefano Magnone, Michele Pisano, Elia Poiasina, Laura Alberici, Filippo Antonacci, Alessandro Arena, Angela Belvedere, Fabio Bernagozzi, Paolo Bernante, Pietro Bertoglio, Lorenzo Bianchi, Maria Bisulli, Barbara Bonfanti, Safia Boussedra, Jury Brandolini, Crescenzo Cacciapuoti, Stefano Cardelli, Riccardo Casadei, Matteo Cescon, Alessandro Cipolli, Riccardo Cipriani, Luca Contu, Francesco Costa, Niccolo Daddi, Eugenia De Crescenzo, Pierandrea De Iaco, Alessandra De Palma, Massimo Del Gaudio, Anna Nunzia Della Gatta, Giampiero Dolci, Giulia Dondi, Matteo Droghetti, Sergio Nicola Forti Parri, Elena Garelli, Chiara Gelati, Giuliana Germinario, Federico A. Giorgini, Carlo Ingaldi, Elio Jovine, Kenji Kawamukai, Antonio Lanci Lanci, Raffaele Lombardi, Maria Elisa Lozano Miralles, Claudio Marchetti, Michele Masetti, Francesco Minni, Daniele Morezzi, Daniele Parlanti, Alice Pellegrini, Anna Myriam Perrone, Anna Paola Pezzuto, Marco Pignatti, Gianluigi Pilu, Valentina Pinto, Gilberto Poggioli, Silvana Bernadetta Puglisi, Diego Raimondo, Matteo Ravaioli, Claudio Ricci, Sara Ricciardi, Francesco Ricotta, Roberta Rizzo, Angela Romano, Matteo Rottoli, Riccardo Schiavina, Renato Seracchioli, Matteo Serenari, Margherita Serra, Piergiorgio Solli, Gioia Sorbi, Mario Taffurelli, Marta Tanzanu, Achille Tarsitano, Marco Tesei, Gabriele Vago, Tommaso Violante, Simone Zanotti, Raffaele Aspide, Giacomo Bertolini, Carlo Bortolotti, Alessandro Carretta, Ambra Caruso, Alfredo Conti, Carla De Vita, Filippo Friso, Emanuele La Corte, Diego Mazzatenta, Alessandro Pirina, Vittoria Rosetti, Carmelo Sturiale, Matteo Vincenzi, Matteo Zoli, Francesco Castagnini, Davide Maria Donati, Tommaso Frisoni, Stefano Lucchini, Emanuela Palmerini, Francesco Traina, Anna Maria Baietti, Bruno Berselli, Silvia Bolognesi, Erich Fabbri, Francesco Farnia, Pietro Maremonti, Concetta Marganella, Ernesto Pasquini, Vito Antonio Piserchia, Gian Marco Prucher, Alessandra Razzaboni, Silvia Ricci, Giacomo Sollini, Caterina Testoni, Mohammed Abu Hilal, Nine de Graaf, Roberta La Mendola, Giulia Arrigoni, Gian Luca Baiocchi, Elena Cagnazzi, Rossella D’Alessio, Francesco Doglietto, Federico Ferrari, Marco Fontanella, Batog Igor, Sarah Molfino, Franco Odicino, Pier Paolo Panciani, Giorgio Saraceno, Enrico Sartori, Luca Zanin, Giuseppe Esposito, Federica Frongia, Adolfo Pisanu, Mauro Podda, Nicola Cillara, Alessandro Cannavera Putzu, Raffaele Sechi, Emmanuele Abate, Massimiliano Casati, Letizia Laface, Marcello Schiavo, Fabio Marino, Fabrizio Perrone, Paolo Annicchiarico, Alessandro Cappellani, Matteo Cavallo, Alessia Giaquinta, Rossella Gioco, Massimiliano Veroux, Pierfrancesco Veroux, Antonino Zanghì, Antonio Cianci, Arturo Lo Giudice, Maria Grazia Matarazzo, Giorgio Ivan Russo, Giuseppe Sarpietro, Carmen Emanuela Scandura, Ida Barca, Adriano Carnevali, Antonio Carpino, Maria Giulia Cristofaro, Gilda De Paola, Giuseppe Giannaccare, Daniela Novembre, Giuseppe Sammarco, Vincenzo Scorcia, Angeli Christy Yu, Marcello D’Andrea, Lorenzo Mongardi, Luigino Tosatto, Mirko Barone, Felice Mucilli, Angelo Muraglia, Ottavia Caserini, Domenico Benvenuto Giuliani, Marco Monti, Alessia Morello, Edoardo Segalini, Felice Borghi, Desiree Cianflocca, Alberto Daniele, Danilo Donati, Enrico Gelarda, Giorgio Giraudo, Maria Carmela Giuffrida, Alessandra Marano, Elena Olearo, Vincenzo Pruiti Ciarello, Andrea Puppo, Valentina Testa, Marco Giacometti, Sandro Zonta, Eleonora Monti, Andrea Porta, Daniele Sambucci, Arianna Birindelli, Barbara Carrara, Bruno Compagnoni, Roberto Del Giudice, Sara Elisabetta Dester, Daniele Lomiento, Silvia Ruggiero, Lucio Taglietti, Fabio Viotti, Domenico Lacavalla, Savino Occhionorelli, Michele Rubbini, Massimiliano Bernabei, Nicolò Fabbri, Marta Fazzin, Carlo V. Feo, Marco Torchiaro, Renato Costi, Edoardo Virgilio, Lorenzo Arlia, Giuseppe Barbato, Ilenia Bartolini, Andrea Bottari, Chiara Bruno, Alessandro Bruscino, Carlotta Checcucci, Fabio Cianchi, Francesco Coratti, Rosita De Vincenti, Annamaria Di Bella, Massimiliano Fambrini, Laura Fortuna, Oreste Gallo, Giacomo Gigliucci, Luca Giovanni Locatello, Gherardo Maltinti, Jacopo Martellucci, Paolo Prosperi, Maria Novella Ringressi, Flavia Sorbi, Fabio Staderini, Antonio Taddei, Alessandro Anastasi, Giuseppe Canonico, Emiliano Chisci, Linda Gabellini, Fabrizio Masciello, Stefano Michelagnoli, Tommaso Nelli, Luca Tirloni, Giovanni Alemanno, Armando Arminio, Renata Beck, Carlo Bergamini, Antonella Cotoia, Vincenzo Lizzi, Giuseppe Maccagnano, Vito Pesce, Antonio Luciano Sarni, Nicola Tartaglia, Fernanda Vovola, Andrea Avanzolini, Antonio Bocchino, Raffaele Bova, Davide Cavaliere, Fabrizio D’acapito, Giorgio Ercolani, Francesca Fappiano, Carlo Alberto Pacilio, Leonardo Solaini, Giancarlo D’Andrea, Laura Lavalle, Veronica Picotti, Andrea Barberis, Marco Filauro, Matteo Santoliquido, Alessandra Aprile, Fabio Barra, Raffaele De Rosa, Raquel Diaz, Simone Ferrero, Piero Fregatti, Claudio Gustavino, Michele Iester, Chiara Kratochwila, Andrea Massobrio, Davide Pertile, Stefano Scabini, Umberto Scovazzi, Domenico Soriero, Marco Sparavigna, Carlo Traverso, Aldo Vagge, Denise Gambardella, Manfredo Tedesco, Stefano D’Ugo, Norma Depalma, Marcello Giuseppe Spampinato, Angelo Airoldi, Ariberto Brivio, Marco Chiarelli, Ludovica Gibelli, Bonfanti Giulia, Samuele Grandi, Giovanni Pesenti, Fulvio Tagliabue, Mauro Zago, Riccardo Lenzi, Jacopo Matteucci, Luca Muscatello, Gaia Colletti, Marco Guido Confalonieri, Andrea Costanzi, Colomba Frattaruolo, Andrea Locatelli, Michela Monteleone, Giorgio Badessi, Maria Caffo, Gerardo Caruso, Eugenio Cucinotta, Antonino Francesco Germano, Carmelo Mazzeo, Camillo Leonardo Bertoglio, Paolo De Martini, Giovanni Ferrari, Alessandro Giani, Pietro Maria Lombardi, Michele Mazzola, Ludovica Baldari, Daniele Bissacco, Luigi Boni, Elisa Cassinotti, Maurizio Domanin, Lorenzo Pignataro, Sara Torretta, Santi Trimarchi, Daniele Armellin, Silvia Basato, Laura Bernardi, Francesca Bunino, Giovanni Capretti, Francesco Maria Carrano, Carlo Castoro, Giovanni Colombo, Andrea Costantino, Francesca De Lucia, Armando De Virgilio, Matteo Di Bari, Fabio Ferreli, Francesca Gaino, Marco Gramellini, Carlotta La Raja, Luca Malvezzi, Salvatore Marano, Giuseppe Mercante, Flavio Milana, Gennaro Nappo, Georgios Peros, Francesca Pirola, Vanessa Rossi, Elena Russo, Giuseppe Spriano, Sara Tamburello, Alessandro Zerbi, Alberto Aiolfi, Davide Bona, Andrea Sozzi, Laura Adamoli, Mohssen Ansarin, Luca Bertolaccini, Sabine Cenciarelli, Francesco Chu, Rita De Berardinis, Uberto Fumagalli Romario, Giacomo Pietrobon, Giulia Sedda, Lorenzo Spaggiari, Marta Tagliabue, Antonella Ardito, Maria Caputo, Paola Cellerino, Valentina D’alessandro, Elisa Galfrascoli, Maria Paola Giusti, Marco Lotti, Roberto Santambrogio, Marco Antonio Zappa, Andrea Bondurri, Alessandro Michele Bonomi, Francesco Colombo, Michele Achille Crespi, Piergiorgio Danelli, Angelo Gabriele Epifani, Luca Ferrario, Alice Frontali, Claudio Guerci, Anna Maffioli, Francesco Ferrara, Luca Antonio Aldrighetti, Domenico Baccellieri, Luca Bertoglio, Giulia Bonavina, Massimo Candiani, Giorgio Candotti, Arianna Casiraghi, Laura Mariangela Castellano, Paolo Ivo Cavoretto, Roberto Chiesa, Federica Cipriani, Paola De Nardi, Guido Fiorentini, Filippo Gagliardi, Alessandro Galdini, Alessandro Grandi, Elena Marotta, Simonetta Massaron, Andrea Melloni, Pietro Mortini, Gianluca Nocera, Martina Piloni, Mirko Pozzoni, Francesca Ratti, Riccardo Rosati, Alessandro Ferdinando Ruffolo, Pierpaolo Sileri, Alfio Spina, Andrea Vignali, Cristina Barberio, Luigi Beretta, Francesca Cavenago, Nora Di Tomasso, Stefano Fresilli, Giovanni Landoni, Stefano Lazzari, Gaetano Lombardi, Marilena Marmiere, Fabrizio Monaco, Gabriele Todaro, Stefano Turi, Alberto Zangrillo, Francesca Bertolina, Giorgio Bogani, Stefano Bonomi, Pierfrancesco Cadenelli, Valentina Chiappa, Stefano Piero Bernardo Cioffi, Davide Citterio, Lara Valentina Comini, Umberto Cortinovis, Maurizio Cosimelli, Antonino Ditto, Marco Fiore, Massimiliano Gennaro, Lorenzo Giannini, Alessandro Gronchi, Marcello Guaglio, Marco Guzzo, Andrea Leva, Alberto Macchi, Elena Manzo, Fabio Martinelli, Ilaria Mattavelli, Vincenzo Maria Mazzaferro, Francesco Raspagliesi, Luigi Rolli, Laura Sala, Roberto Salvioni, Mario Santinami, Silvia Segattini, Luca Sorrentino, Carlotta Zaborra, Alexandre Anesi, Gianmaria Casoni Pattacini, Mattia Di Bartolomeo, Francesca Pecchini, Arrigo Pellacani, Micaela Piccoli, Federico Fusini, Andrea Gattolin, Marco Migliore, Roberto Rimonda, Diego Sasia, Elisabetta Travaglio, Federica Brunetti, Marco Cereda, Marco Ceresoli, Luca Cigagna, Cristina Dell’Oro, Alessandro Fogliati, Robert Fruscio, Tommaso Grassi, Maini Marzia Isabella, Luca Carlo Nespoli, Massimo Oldani, Sara Ornaghi, Nicolò Tamini, Carmine Antropoli, Antonio Castaldi, Alessio Palumbo, Gaia Altieri, Umberto Bracale, Francesco Corcione, Marcello De Luca, Giovanni Domenico De Palma, Maria Michela Di NUZZO, Ruggero Lionetti, Dalila Loredana Lo Bue, Gaetano Luglio, Gianluca Pagano, Roberto Peltrini, Nello Pirozzi, Francesca Paola Tropeano, Alessia Aversano, Andrea Belli, Maria D’amico, Paolo Delrio, Francesco Izzo, Renato Patrone, Daniela Rega, Pietro Maida, Ester Marra, Gianpaolo Marte, Andrea Tufo, Francesco Bianco, Antonio Cappiello, Simona Gili, Paola Incollingo, Alessandra Novi, Giulia Bagaglini, Claudio Iovino, Francesco Menegon Tasselli, Maria Paola Menna, Francesco Maria Romano, Settimio Rossi, Guido Sciaudone, Francesco Selvaggi, Lucio Selvaggi, Francesca Simonelli, Guido Coretti, Mario Pannullo, Adolfo Renzi, Francesca Ascari, Giuliano Barugola, Giacomo Ruffo, Paolo Baroffio, Paolo Bellora, Laura Enrica Benedetti, Cristina Cerri, Giordana D’Aloisio, Maurizio Ferrari, Elisa Francone, Sergio Gentilli, Herald Nikaj, Antonella Chessa, Alessandro Fiorini, Luca Campagnaro, Franco Chioffi, Pietro Ciccarino, Roberto Colasanti, Francesco de Falco, Fotios Kalfas, Gioacchino Mattisi, Giulia Nezi, Matteo Palma, Angelica Rizzoli, Domenico Rossi, Davide Russo, Renato Salvador, Francesco Volpin, Guido Bissolotti, Stefano Fusetti, Francesco Lemma, Vito Chiantera, Mariano Catello Di Donna, Giulio Sozzi, Emanuele Cammarata, Sofia Campanella, Daniela Canzonieri, Adriana Cordova, Federico De Michele, Ettore Dinoto, Mara Franza, Leo Licari, Daniele Matta, Domenico Mirabella, Felice Pecoraro, Roberto Pirrello, Pierfrancesco Pugliese, Fernando Rosatti, Giuseppe Salamone, Francesca Toia, Massimiliano Tripoli, Cosimo Callari, Dario Di Miceli, Leo Licari, Alfredo Annicchiarico, Luca Bellanti, Michela Bergonzani, Roberto Berretta, Elisa Cabrini, Vito Andrea Capozzi, Fausto Catena, Federico Cozzani, Paolo Del Rio, Marco Domenichini, Anna Fornasari, Antonio Freyrie, Tiziana Frusca, Mario Giuffrida, Gennaro Perrone, Giulia Rossi, Matteo Rossini, Andrea Varazzani, Vittorio Arici, Antonio Bozzani, Lorenzo Cobianchi, Matteo Filardo, Marika Sharmayne Milani, Franco Ragni, Andrea de Manzoni Garberini, Edoardo Baldini, Diana Carpaneto, Michele Cauteruccio, Corrado Ciatti, Luigi Conti, Serena Gattoni, Pietro Maniscalco, Gerardo Palmieri, Calogero Puma Pagliarello, Giuseppe Caristo, Raffaele Galleano, Michele Malerba, Marcello Calabrò, Francesca Farnesi, Elia Giuseppe Lunghi, Andrea Muratore, Nicoletta Sveva Pipitone Federico, Joel Reuben Abel, Lorenzo Andreani, Darienzo Antonio, Vittorio Aprile, Riccardo Balestri, Giacomo Benettini, Stefano Berrettini, Luca Bruschini, Massimo Chiarugi, Federico Coccolini, Simone Colangeli, Camilla Cremonini, Lodovica Cristofani Mencacci, Iacopo Dallan, Silvia De Santi, Gregorio Di Franco, Lorena Di Girolami, Giacomo Fiacchini, Niccolò Furbetta, Stylianos Korasidis, Marco Lucchi, Andrea Morandi, Luca Morelli, Serena Musetti, Carlo Maria Neri, Matteo Palmeri, Miriana Picariello, Francesco Porcelli, Marco Puccini, Nicolo Roffi, Erica Statuti, Dario Tartaglia, Alberto Tonelli, Matteo Vianini, Gianluca Baronio, Mauro Montuori, Enrico Pinotti, Stefano Maria Massimiliano Basso, Federica Maffeis, Paolo Ubiali, Lorenzo Aguzzoli, Saverio Coiro, Giuseppe Falco, Vincenzo Dario Mandato, Valentina Mastrofilippo, Simone Mele, Caterina Baldi, Carlo Corbellini, Gianluca Matteo Sampietro, Massimo Dugo, Mauro Garino, Chiara Marafante, Antonio Masciandaro, Elisabetta Moggia, Alessandra Murgese, Felice Eugenio Agro, Gabriella Teresa Capolupo, Filippo Carannante, Marco Caricato, Vincenzo Denaro, Erica Mazzotta, Rocco Papalia, Giuseppe Pascarella, Alessandro Strumia, Biagio Zampogna, Matteo Cinquepalmi, Marco Colasanti, Celeste Del Basso, Federica Falaschi, Nicola Guglielmo, Roberto Luca Meniconi, Alessandra Pecoraro, Sofia Usai, Giacomo Crescentini, Antonella Larcinese, Emanuele Picone, Giovanni Sinibaldi, Annamaria Agnes, Salvatore Agnes, Sergio Alfieri, Francesco Belia, Valentina Bianchi, Giuseppe Bianco, Alberto Biondi, Paola Campennì, Valerio Cozza, Sabatino D’Archi, Domenico D’Ugo, Veronica De Simone, Marta Di Grezia, Sofia Di Lorenzo, Federica Ferracci, Valeria Fico, Gianluca Franceschini, Pietro Fransvea, Giulio Gasparini, Luca Gordini, Antonio La Greca, Francesco Litta, Celestino Pio Lombardi, Angelo Alessandro Marra, Angelo Parello, Marco Maria Pascale, Romeo Patini, Gilda Pepe, Roberto Persiani, Caterina Puccioni, Carlo Ratto, Fausto Rosa, Gianmarco Saponaro, Lorenzo Scardina, Tedesco Silvia, Giuseppe Tropeano, Maria Benevolo, Daniele Bugada, Flaminia Campo, Maria Gabriella Dona, Valentina Manciocco, Paolo Marchesi, Riccardo Mastroianni, Francesco Mazzola, Silvia Moretto, Raul Pellini, Gerardo Petruzzi, Barbara Pichi, Giuseppe Simone, Gabriele Tuderti, Jacopo Zocchi, Luigi Marino Cosentino, Andrea Sagnotta, Roberta Angelico, Vittoria Bellato, Michela Campanelli, Marzia Franceschilli, Michele Grande, Giorgio Lisi, Tommaso Maria Manzia, Lorenzo Petagna, Bruno Sensi, Giuseppe Sica, Leandro Siragusa, Giuseppe Tisone, Marco Assenza, Barbara Binda, Massimo Biondi, Gioia Brachini, Placido Bruzzaniti, Mauro Casagrande, Flavia Ciccarone, Pierfranco Maria Cicerchia, Bruno Cirillo, Daniele Crocetti, Giancarlo D’ambrosio, Vito D’andrea, Francesca De Felice, Giorgio De Toma, Carlo Della Rocca, Giulia Duranti, Pietro Familiari, Enrico Fiori, Giovanni Battista Fonsi, Alessandro Frati, Stefania La Rocca, Filippo La Torre, Pierfrancesco Lapolla, Giovanni Marruzzo, Simona Meneghini, Andrea Mingoli, Francesco Pata, Andrea Picchetto, Antonella Polimeni, Diego Ribuffo, Maurizio Salvati, Antonio Santoro, Paolo Sapienza, Luigi Simonelli, Valentino Valentini, Martina Zambon, Giuseppa Zancana, Emma Zuppi, Simone D’Annunzio, Cosimo De Nunzio, Silvia Fiorelli, Mohsen Ibrahim, Chiara Loffredo, Domenico Massullo, Cecilia Menna, Rocco Monica, Massimiliano Pelli, Erino Angelo Rendina, Leonardo Teodonio, Andrea Tubaro, Giulio Argenio, Giulio Accarino, Accarino Giancarlo, Antonio Nicola Giordano, Luca Cardinali, Elisa Sebastiani, Grazia Travaglini, Erika Andreatta, Emanuele Luigi Giuseppe Asti, Daniele Bernardi, Luigi Bonavina, Caterina Froiio, Andrea Lovece, Chiara Copelli, Alfonso Manfuso, Pasquale Di Maio, Marco Giudice, Oreste Iocca, Rosario Cennamo, Tommaso Cornali, Francesco Di Marzo, Cristian Altana, Francesco Bussu, Giampiero Capobianco, Anna Giacomina Carta, Sandro Ciccarello, Maria Laura Cossu, Pietrina Cottu, Giacomo De Riu, Salvatore Dessole, Francesco Dessole, Salvatora Dettori, Carlo Doria, Alessandro Fancellu, Claudio F Feo, Giorgio Carlo Ginesu, Giuliana Giuliani, Marco Giuseppe Iannuccelli, Massimo Madonia, Roberto Mancino, Olindo Massarelli, Gianfranco Meloni, Fabio Milia, Andrea Mulliri, Teresa Perra, Marco Petrillo, Antonio Piras, Franco Piredda, Francesco Pisanu, Alberto Porcu, Davide Rizzo, Angelino Sanna, Antonio Mario Scanu, Fabrizio Scognamillo, Damiano Soma, Anna Rita Tanca, Alessandro Tedde, Matteo Tedde, Luigi Angelo Vaira, Andrea Bartalini Cinughi de Pazzi, Osvaldo Carpineto Samorani, Daniele Fusario, Luigi Marano, Fabio Marino, Gaia Oldrà, Anna Lisa Pesce, Stefania Angela Piccioni, Luca Resca, Vincenzo Ricchiuti, Franco Roviello, Vinno Savelli, Alberto Abrate, Pierpaolo Bordoni, Lorenzo Bosio, Guglielmo Clarizia, Francesco Fleres, Marco Franzini, Antonio Fratto, Pierluigi Giumelli, Alessandro Grechi, Alessandro Longhini, Fabrizio Lorusso, Elisa Scarnecchia, Federica Scolari, Alessandro Spolini, Vincenzo Maiuri, Matteo Papandrea, Arturo Roncone, Lorenzo Conti, Andrea Rizzi, Marco Rovagnati, Alberto Brolese, Tommaso Cai, Francesco Antonio Ciarleglio, Francesca Dalprà, Gianni Malossini, Liliana Mereu, Irene Tamanini, Saverio Tateo, Giovanni Viel, Enrico Battistella, Paolo Boscolo Rizzo, Cristoforo Fabbris, Marco Massani, Giacomo Spinato, Roberta Tutino, Ugo Grossi, Alessandro Iacomin o, Simone Novello, Maurizio Romano, Serena Rossi, Giulio Santoro, Giacomo Zanus, Sokol Trungu, Giada Aizza, Gabriele Bellio, Selene Bogoni, Marina Bortul, Biagio Casagranda, Sara Cortinovis, Nicolò de Manzini, Davide Drigo, Paola Germani, Manuela Mastronardi, Lucia Paiano, Silvia Palmisano, Pier Luigi Filosso, Francesco Guerrera, Matteo Marro, Mauro Rinaldi, Enrico Ruffini, Stefano Salizzoni, Laura Bardelli, Mattia Berselli, Giacomo Borroni, Eugenio Cocozza, Matteo Desio, Salomone Di Saverio, Bottazzoli Elisa, Giuseppe Ietto, Valentina Iori, Domenico Iovino, Lorenzo Livraghi, Valentina Marchionini, Stefano Megna, Emma Amal Nahal, Mara Palumbo, Valeria Quintodei, Alessandra Zullo, Lucrezia D’Alimonte, Giovanni Pirozzolo, Chiara Vignotto, Tommaso Campagnaro, Andrea Caravati, Simone Conci, Carlotta De Cristofaro, Gabriele Gecchele, Tommaso Giuliani, Jacopo Graziosi, Alfredo Guglielmi, Salvatore Paiella, Corrado Pedrazzani, Tommaso Pollini, Simone Rattizzato, Andrea Ruzzenente, Roberto Salvia, Giulia Turri, Matilde Bacchion, Giovanni Butturini, Andrea Casaril, Alessandro Giardino, Harmony Impellizzeri, Marco Inama, Frigerio Isabella, Gianluigi Moretto, Marco De Zuanni, Enrica Deiana, Mario Guglielmo, Alessandro Broglia, Claudia Casarini, Caterina Costanza Zingaretti, Marta Bonaldi, Giovanni Cesana, Francesco Mastriale, Stefano Olmi, Matteo Uccelli Yasuyuki Fukami, Takuya Saito, Tsuyoshi Sano, Naoki Hirai, Kazuyoshi Hirota, Tetsuya Kushikata, Tasuku Oyama, Junichi Saito, Katsuhiko Ishibashi, Mizue Kamiyama, Kyongsuk Son, Kentaroh Tarao, Takayuki Yamada, Toshiya Shiga, Chisaki Aze, Yoshihiko Deguchi, Hirotoshi Hasegawa, Tatsuki Hoshino, Yasushi Innami, Hiroyuki Inoue, Shingo Ito, Takeshi Nomura, Tomomi Ogihara, Reina Okada, Takashi Ouchi, Yuri Sekiya, Keikoku Tachibana, Emi Takano, Masae Yamamoto, Shuko Matsuda, Yuka Matsuki, Kenji Shigemi, Teruyuki Hiraki, Yui Inoue, Shosaburo Jotaki, Tatsuro Abe, Masatoshi Eto, Junichi Inokuchi, Eiji Kashiwagi, Fumio Kinoshita, Satoshi Kobayashi, Ken Lee, Takashi Matsumoto, Keisuke Monji, Hidekazu Naganuma, Masaki Shiota, Ario Takeuchi, Shinju Obara, Saori Takatsuki, Saori Tanaka, Koji Iida, Kota Kagawa, Shuichiro Neshige, Tomohiro Chaki, Naoyuki Hirata, Satoshi Kazuma, Motonobu Kimizuka, Sho Kumita, Noriaki Nishihara, Sato Satoshi, Atsushi Sawada, Shunsuke Tachibana, Michiaki Yamakage, Yuta Nakamura, Kozo Sato, Tomoya Irie, Tomoko Irisawa, Eiki Kanemaru, Yuko Koga, Yoshihiko Chiba, Jun Makino, Shinnosuke Ohama, Shinichiro Okada, Kano Teruaki, Tatsuya Kida, Tomohide Takei, Kazuhiro Hanazaki, Hiroyuki Kitagawa, Tsutomu Namikawa, Toshiyuki Mizota, Chikashi Takeda, Shintaro Yagi, Hiroshi Imai, Makoto Ishitobi, Manabu Kato, Kouhei Nishikawa, Takeshi Sasaki, Hiroshi Yonekura, Akihiro Kanaya, Daisuke Irimada, Haruka Ishikawa, Yu Kaiho, Mitsuru Ida, Masahiko Kawaguchi, Kenji Kawamura, Munehiro Ogawa, Hiroshi Okada, Shunji Endo, Yoshinori Fujiwara, Masaharu Higashida, Hisako Kubota, Toshimasa Okada, Hironori Tanaka, Tomio Ueno, Kazuhiko Yoshimatsu, Motohiro Kikukawa, Akira Kuriyama, Susumu Matsushime, Daisuke Hashimoto, Hishikawa Hidehiko, Haruaki Hino, Yoji Hisamatsu, Akio Kamiya, Hidefumi Kinoshita, Masato Kita, Toshinori Kobayashi, Taku Michiura, Hirokazu Miki, Tomohiro Murakawa, Hidetaka Okada, Tomohito Saito, Ryoichi Saito, Motohiko Sugi, Genichiro Sumi, So Yamaki, Tomohisa Yamamoto, Aya Yoshida, Tatsuya Kambara, Sayaka Kanematsu, Okazaki Satoshi, Takeshi Hijikawa, Hiroaki Kitade, Hidesuke Yanagida, Tomoyuki Fujita, Satsuki Fukushima, Naoki Tadokoro, Taku Furukawa, Yusuke Iizuka, Yuji Otsuka, Masamitsu Sanui, Ikumi Sawada, Hideki Iwahashi, Morikazu Miyamoto, Masashi Takano, Tsutomu Mieda, Chihiro Ando, Tetsuro Isada, Taku Ishizaki, Qaed Bani Amer, Yuki Fujimoto, Sachi Ishida, Yasuma Kobayashi, Norifumi Kuratani, Tomoe Sakurai, Misa Takada, Yutaka Iba, Junji Tsukagoshi, Akira Yamada, Yuki Amano, Kentaro Fumoto, Shusaku Noro, Ayataka Fujimoto, Naoto Kuroda, Kyoichi Tomoto, Yukiyasu Okamura, Teiichi Sugiura, Katshuhiko Uesaka, Natsuki Takemura, Kento Kuroda, Megumi Hayashi, Satoi Kaneko, Izumi Kawagoe, Tsukasa Kochiyama, Ai Yamaguchi, Nobutsugu Abe, Tadao Ando, Mieko Chinzei, Kouichi Hirano, Yoichi Kobayashi, Ryota Matsuki, Hironori Matsumoto, Akira Motoyasu, Harumasa Nakazawa, Hikari Noguchi, Kaio Okamura, Motoaki Ono, Hiroyuki Seki, Eiji Sunami, Atsushi Tajima, Shinji Tanigaki, Kohji Uzawa, Hidenobu Watanabe, Yukari Furuhata, Satoshi Toyama, Tokujiro Uchida, Yutaka Enomoto, Yuri Furukawa, Yoko Hasumi, Jinso Hirota, Katsuyuki Iida, Shingo Ikeda, Tatsuhiko Ikeda, Natsuko Kawamata, Yosuke Kawasaki, Minako Koizumi, Yoshiharu Kono, Shigeki Kuzuhara, Junichi Maeda, Mai Moriyama, Shoichi Nagamoto, Rinako Nakanishi, Michio Noda, Atsushi Seichi, Shintaro Takahashi, Kobayashi Takashi, Misuzu Takeda, Kenta Tanakura, Katsuyuki Terajima, Munechika Tsuji, Chiharu Ueshima, Naoya Yamamoto, Mari Yamamoto, Toshiya Yokota, Yuki Yoshioka, Sakoh Yuri, Shintaro Iwata, Akira Kawai, Shuhei Osaki, Masashi Ishikawa, Masae Iwasaki, Tomonori Morita, Reina Hirooka, Yoshinori Nakata, Shigehito Sawamura, Yusuke Ishida, Aya Kawachi, Takayuki Kobayashi, Fumiaki Nagashima, Naoki Suzuki, Kohei Ando, Noriko Miyazawa, Yukio Tanaka, Khatab Abuissa, Alaa Aljabali, Amal Almasri, Majdi Ali Alqudah, Mera Alrabadi, Rana Alshara, Yasmeen Alzghoul, Ahmed Alzughoul, Ali Freihat, Emran Jeitan, Qais Owais, Aseel Smadi, Mahmoud Abukhadra, Huthifa Abunawas, Abdallah Ahmed Mezel Al‐Azzam, Ammar Aladaileh, Amer Alajalen, Ismail Albadawi, Mohammad Alhawatmeh, Mohannad Yaser Alkhaza’leh, Roaa Salem Jwaid Alneimat, Saba Alwahedy, Abdel Rahman Mohannad Ahmad Alwardat, Slsabela Dhoon, Khaled El‐Qawaqzeh, Tarteel Hrerat, Enas Jaradat, Teeba Mubaydeen, Husam Sarayrah, Mohammad Abdel Elah A. Tarawneh, Mohammad Yousef Karasneh, Amany Zurgan, Mohammad Alassaf, Sayel H. Alzraikat, Justin Z. Amarin, Ahmed Hijjawi, Rand Y. Omari, Haya H. Suradi, Louay Y. Zaghlol, Ahmad Abdalah, Ghaida Abdallah, Ammar Abu Tarieh, Tareq Abu‐libdeh, Faisal Abualteen, Murad Al Abdallah, Zakaria Al Bdour, Seif Al Dahabrh, Farah Al‐kasaji, Zaid Al‐sheikh Ali, Mohammad Al‐thaher, Tareq Alnajjar, Mohannad Alqedrh, Mohammad Alsaleh, Mohammad AlZaatreh, Ghandi Amayreh, Faris Ayasra, Yazeed Ayasra, Ahmad Eid, Ibrahim Ghayada, Khaled Moh’d Ahmed Hasanein, Anas Hassouneh, Raid Hijazeen, Majedah Hmeidan, Ahmed Khaled, Barihan Khasawneh, Omar Kifayeh, Yasser Lafi, Abdulrahman Qasem, Jasem Seba, Qusai Semrin, Fda Sma, Mohammad Theab, Haitham Tumeh, Muna Aba Zaid, Alaa Abazeed, Bader Abbad, Osaid Abbadi, Nizar Abu‐Ishkerih, Malk Al‐Osta, Reham Al‐Zyadat, Yazan Alawneh, Bashar Omar Falah Alawneh, Majd Alhattab, Mohd Mujahed Alkurdieh, Ziad Awwad, Mohammad Hammouri, Hamzeh Hussein, Sereen Khader, Mariam Khalil, Omar Mansour, Ahmad Othman, Haitham Qandeel, Mohammad Saleem, Fouad Souri, Ahmad Tahboub, Raed Tayyem, Munir Abdallah, Ibrahim Abdel‐Hafez, Hamza Abu Obead, Hadeel Abudari, Ehab Abuhamour, Samer Abusadah, Amer Ahmad, Nimer Al‐azzeh, Sofian Al‐Adwan, Ali Al‐Darabah, Mohammad Al‐Qannas, Omar Aladawi, Basil Albaba, Qutaiba Alradawneh, Bourhan Alrayes, Mohammad Amaireh, Subhi Ashour, Reem Chabaan, Ammar Eskander, Ahmad Fawzi, Hanan Haidar, Manal Haij, Hisham Hamad, Haya Hamdan, Ahmed Hamss, Esraa Hassouneh, Shatha Husain, Ayah Hyasat, Zainab Ibrahim, Saja Jamaliah, Mohammed Khalil, Anas Massad, Ishak Mohamed, Mahmoud Qandeel, Dalia Qasrawi, Faisal Qassem, Mahmoud Raggad, Ebaa Rifai, Nedal Semreen, Reham Shehada, Marwan Shorman, Abdallah Al‐Shibi, Shatha AlSA’AFIN, Morad Bani‐hani, Bahaa Nimer, Sandra Nofal, Layth Qaraqe, Mohammed Salameh, Ma’moun Saleh, Ghaidaa Sanjuq, Alaa Sharabi, Adnan Sumadi, Alaa Sweiti, Jamal Yaghmour, Sabri Zaza, Rahaf Alhindi, Eman Baninasr, Nizar Abu‐Ishkerih, Amro Abuleil, Mohamad K. Abou Chaar, Mahmoud Al‐Masri, Hani Al‐Najjar, Fade Alawneh, Motasem Al‐latayfeh, Abdel‐Ellah Al‐Shudifat, Abd Almonem Shaikh Ahmad, Abdelkarim Abdeljalil, Haneen Abdelrahman, Abdulmalek Abdulmalek, Lina Abedalqader, Laith Abu Abed, Amro Abuleil, Omar Al Smadi, Salameh Alarood, Mohammad Allouzi, Maha Almansour, Sari Almi’ani, Abdulhakim Alsaiad, Khaled Alshaikh, Farah Androus, Mirna Awbakh, Belal Azzam, Hammam Bany Yasin, Maher Daoud, Lujain Dawod, Omar Dawod, Reem Elmusa, Saba Fadli, Farah Hammad, Majd Hunaiti, Elmi Ahmed Mohamed Jimaale, Mahmoud Muhtaseb, Rawan Owaimer, Zaki Qulaghassi, Saleh Romman, Rawand Saket, Said Sharawi, Alaa Tawalbeh, Mohammed Al‐howthi, Laila Ababneh, Roba Ababneh, Faris Jamal Abu Za’nouneh, Marah Abu‐Mehsen, Sajedah Al DHOUN, Ahmad Al Khassawneh, Ahmed Al Sharie, Ayah Ahmad Al Shraideh, Tawfik Al‐Dabaa, Salsabeel Al‐Jarrah, Hothaifa Al‐Jarrah, Hadeel Al‐Othman, Zuhair Alaradi, Qutaiba Alshannaq, Rima Alsulaiman, Motasem Alzaqh, Yazan Alzu’bi, Ziad Audat, Mohammad Bahhour, Saja Bdour, Roa Khatatbeh, Fares Marji, Tagleb Mazahreh, Dina Nail, Amjad Nuseir, Asmaa Rabab’ah, Taher Sawadi, Omar Wafi, Ghina Zidan, Dima Abu Muhfouz, Abdullah Nael, Ahmad Qasim, Hazim Ababneh, Amar Al‐Jarrah, Faisal Rawagah, Dima Y Abu Ismail, Luai Abu‐Ismail, Wedad Al‐dolat, Mai Alzoubi, Malak Alzoubi, Mu’taz Alzu’bi, Ruba Asha, Ali Guboug, Hasan Hussein, Almu’atasim Khamees, Shireen Rawashdeh, Mohammad Sanwar, Adnan Ababneh, Basil Abu‐Eisheh, Haneen Ahmad Alhami, Saif Aldeen Al Dwairi, Hana’ Al Shurman, Zakaria Al Yahya, Hussam Al‐atiyah, Hasan Al‐Balas, Anas Aljaiuossi, Ahmad Alkhatib, Shatha Alqawasmi, Sadeel Alqudah, Mahmoud Alshourman, Abdulsalam Bani Hamad, Anas Bany Issa, Raed Ennab, Sara Hamdoni, Zaid Hijazi, Ali Khafaja, Mohamed Mraiyan, Riyad Obeidat, Dina Olaywah, Mohamad Qudah, Mhamdd Talal, Askar Aidarov, Raikhan Bolatbekova, Aisulu Sarmenova, Almat Kodasbaev, Mukhtar Kulimbet, Orazbek Sakhov, Ildar Fakhradiyev, Talgat Nurgozhin, Timur Saliev, Shynar Tanabayeva, Baurzhan Zhussupov, Ydyrys Almabayev, Sultan Amrayev, Berik Dzhumabekov, Maulen Malgazhdarov, Kanat Tezekbaev, Dilyara Kaidarova, Yerlan Kukubassov, Alima Satanova, Michael Mwachiro, Robert Parker, Ian Simel, Shamshuddin Mohammedali, Claude Mwaria, Ralph Obure, Sayed Shah Nur Hussein Shah, Chetan Hirani, Muthoni Kibunyi, Claire Mailu, Victor Mwangi, Roy Ogenya, Lydia Osea, Davies Cheruiyot, Omar Hirsi, Carolyne Muiru, Samuel Wanjara, Chang Woo Kim, Suk Hwan Lee, Ahmad Almulla, Mustafa Dashti, Omar Elghany, Abdullah Sultan, Husain Al‐Mahmeed, Jasim Alabbad, Ibtisam Albader, Shuaib Aldalal, Mahdi Baba, Fareed Abdulsalam, Naser AlHajri, Athari Alwael, Brook Ayele, Hatem Marie, Ahmed Zaitoun, Ahmad M. AlAli, Ahmed ALKhamis, Ous Alozairi, Sulaiman Almazeedi, Salman AlSabah, Mostafa Abdelkarim, Asmaa Al Rashed, Saud Al Subaie, Tariq Al‐Shaiji, Abdullatif Al‐Terki, Omar Alhunaidi, Ali Aljewaied, Khaled AlZamel, Ahmed Farag, Hussein Hayati, Salah Termos, Sarah Wood, Maha Al‐Gilani, Mutlaq Al‐Sihan, Haifa Alotaibi, Dmitrijs Lobovs, Andrejs Pcolkins, Armands Sivins, Ingus Arnolds Apse, Annija Evelīna Berga, Anna Ivanova, Jevgenijs Demicevs, Igors Ivanovs, Aleksejs Kaminskis, Janis Opincans, Jānis Pāvulāns, Agris Rudzāts, Anna Udre, Samer Dbouk, Imadeddine Farfour, Mohamad Rakka, Ingrid Antonios, Joe El Hage, Stephanie Hage, Lea Haiby, Hiba Kahi, Anthony Lichaa, Rita Milan Moussa, Michel Salameh, Riad Sarkis, Obey Albaini, Tony Haykal, Bassem Safadi, Hussein Abou‐Abbass, Reem Al Makari, Zeina Al Zein, Mariam Baidoun, Rania Itani, Mustafa Owiedat, Ali H. Abdel Sater, Michel Akl, Nour Badran, Sara El Mustapha, Fathallah Fatouh, Youssef Ghoussoub, Nader Saad, Fatima Serhan, Bassem Souleiman, Walid Alame, Peter Ghiya, Firas Ibrahim, Rasha Abdallah, Mohamad B. Kassab, Lina Karout, Samar Karout, Aya Abdul Al, Hilal Chaaban, Ghinwa Fakih, Huzifa Haj‐Ibrahim, Rabih Awad, Hadi El Assaad, Khalil Tamoos, Ahmed Aqeelah, Ahmed Alfeqeeh, Alsnosy Abdullah Khalefa Mohammed, Hosam Muftah, Taha Abdulrahman, Naeimah Ahseen, Hayat Omar Abunaaja, Abdulmuez Abdulmalik, Abd El Jawad Al Gasi, Faraj Al Maadany, Ghadah Alkadeeki, Sara Alsaeiti, Fatimah Elkhafeefi, Nasre Elrefai, Rofida Ahmad, Wafa Aldressi, Tarek Alhouni, Safya Alkarky, Hana Alkeelani, Rauoof Alkuwafi, Rema Benhariz, Nagat Bettamer, Musa Busarira, Hassan Bushaala, Tariq Diryaq, Osama Elbargathe, Tarik Eldarat, Mustafa Elfadli, Jamael Elfitori, Asma Elmahgoub, Reem Elsahti, Milad Gahwagi, Yosef Hassan, Fathi Hussain, Taha Mirsal, Marwa Mohammed, Ebtesam Saad, Wafa Aldressi, Rauoof Alkuwafi, Tarik Eldarat, Mohamed Amnaina, Haifaa Il Hadad, Mostafa Mosabha, Arowa Hassan Abdulrahman Alansari, Awatif Alhaje, Aaya Haron, Akram Alkaseek, Suhir Alsuwiyah, Mona Hamed, Ghozlan Yagoub, Samer Khel, Sana Omar, Rowaida Yousef, Abubaker Abdelmalik, Yahya Abosnaina, Ma’aly Abuhlaiga, Ahmed Omar Abushahma, Alhosen Saleh M Aldelensi Alzubi, Asma Aljanfi, Mohamed Alnaser Alnehum, Ahmad Alzedam, Anass Ben Amer, Malik Delhen, Galia Maderi, Faisel Matoug, Burooj Mohammed, Marwa Salem, Mohamed Sawalem, Alhadi Jahan, Abdussalam Jahan, Basma Alazabi, BahaUldin Alezabi, Ayman Almugaddami, Mona Masaud Amro Alazabi, Amin Salem Ahmed Egdeer, Fatma Sefi, Dania Burgan, Elhusain Kamoka, Abdulrauf Salamah, Alya Abdalhadi, Saedah Abdeewi, Mohammed Abdelkabir, Marwah AlHADAD, Ahmed Awrayit, Mabroka Eshnaf, Mohammad Yahmad, Abdulhafid Abudher, Khayriyah Alshareef, Faisl Elamin, Farah Abojeila, Mohamed Aliwa, Rayet Al Islam Ben Jouira, Sana Shagour, Ahmed Altobal, Ayman Meelad, Omar Aljuroushi, Hadeel Almadani, Hajer Attia, Ahmad Bouhuwaish, Amal Sharif Eljali, Ayyah Emran, Mabruka Omar, Radhwan Salim, Ashraf Samer, Nayrouz Abdullah Abulshuwashi, Emadeddin T M Ben Khalifa, Hdaya Abdalla S Benabdalla, Hatem Elnageh, Ali Hammad, Duha Milad Abdullah, Marwa Abusalem, Esraa Ben Esmael, Fatma Benmasoud, Laila Debri, Sumayya Essayah Dwaga, Randa Elmokhtar, Manal Ezeddin Kamel Sheta, Houda Khalifa, Bushra Sayeh Mohamed Elhabashi, Balkees Younis, Bushray Almiqlash, Amna Elmabrouk, Ahmed Momen, Abdalrahem Essied Alzubi Alzoubi, Kusay Ayad, Ameerah Mahdi Abraheem Hasan, Fawzie Musrati, Taha Suliaman, Malek Abusannoga, Mohammed Alawami, Ans Malek, Hatem Ajaj, Fras Elhajdawe, Eman Abdulwahed, Hajir Aboazamazem, Mohammed Aboubeirah, Aya Alqurpaa, Entisar Alshareea, Sarra Aribi, Tomather Aribi, Marwa Biala, Reem Ghamgh, Mohannned Issa, Amani Hamid Lamari, Mohammed Mufth, Omar Sudig Abboud, Muad Fathi Khalleefah Abu Hallalah, Saleh Abumahara, Sarah Aburima, Mohammed Adrees, Abdurahman Ahtash, Asmaa Al Shukri, Hind Alameen, Rehab Alarabi, Alarabi Alarabi, Aisha Alelwany, Abdulkarim Alesmail, Noran Algadi, Khuloud Almaqrahi, Hadel Alosta, Mohamed Alsharedi, Rasha Bashir, Mahmoud Ben Ghrema, Sari Bin Nour, Sofian Elbarouni, Ahmed Elhadi, Abdulmohimen A Elkhadar, Esraa Elkouba, Ibrahim Ellojli, Nabiha Elmsherghi, Abdulmohimen Gammoudi, Abdulraouf Ghariba, Fatima Zayed Gjam, Arwa Haidar, Hala Helal, Mohammed Huwaysh, Ahmed Khalid Alhadheeri, Eslam Kriem, Ahmed Msherghi, Halim Osman, Mohamed Ali Ossman, Marwah Shilfeet, Laila Turshani, Nouran Albishty, Abdulmueti Alhadi, Salem Ali, Manal Ahmed Altoumi, Sumayyah Ghayth Bahroun, Eman Younes, Hana Atarabulsi, Mohamed Elshaibi, Abdullmujeeb Othman, Ismail Ali Saleh, Mohammed Alayan, Hayat Ben Hasan, Najat Shaban Ben Hasan, Rabab Shaban Ben Hasan, Vidmantas Barauskas, Edvinas Dainius, Zilvinas Dambrauskas, Albertas Dauksa, Inga Dekeryte, Rita Gudaityte, Kristijonas Jasaitis, Akvilė Koženiauskaitė, Virgilijus Krasauskas, Ausra Lukosiute‐Urboniene, Almantas Maleckas, Romualdas Riauka, Vygintas šlenfuktas, Saulius Svagzdys, Kestutis Urbonas, Tomas Vanagas, Linas Venclauskas, Donatas Venskutonis, Justas Zilinskas, Marijus Ambrazevicius, Vilius Syminas, Oleg Aliosin, Agne Cizauskaite, Vitalijus Eismontas, Jonas Jurgaitis, Vytenis Mikutaitis, Donatas Petrauskas, Narimantas Evaldas Samalavicius, Dainius Simcikas, Algirdas Slepavicius, Albinas Tamosiunas, Vaidotas Turskis, Bernardas Vasiliauskas, Laura Aniukstyte, Audrius Dulskas, Gediminas Januška, Justas Kuliavas, Margarita Montrimaite, Albertas Cekauskas, Aiste Gulla, Azuolas Algimantas Kaminskas, Sarunas Kozenevskis, Tomas Poskus, Kestutis Strupas, Arunas Zelvys, Georges Decker, Audrey Noël, Maheriandrianina Fanambinana Voahary Rajaonarivony, Randimbinirina Zakarimanana Lucas, Herimampionona E Andriantsoa, Mamisoa B Rasamoelina, Rakotonarivo Aina Andrianina Vatosoa, Liantsoa Andriamanana, Casimir FP Rahantasoa Finaritra, Aristide Romain Raherison, Tsirimalala Rajaobelison, Haritiana Rakotoarisoa, Maharo Ramifehiarivo, Solonirina Harinarindra Ranaivoson, Hajamihamina Marina Parfaite Randriantsoa, Fanjandrainy Rasoaherinomenjanahary, Narindra Njarasoa Mihaja Razafimanjato, Jeannie BA Razafindrahita, Luc Hervé Samison, Solofoarimanana Solofoarimanana, Guillaume Odilon Tsiambanizafy, Rakotonaivo Mamisoa Judicaël, Corinne Eulalie Solo, Tojomamy Herinjaka Ralaizafindraibe, Fanonjomahasoa Safiry Andofenohasina, Hanta Rasataharifetra, Vanona Barijaona Razafindraibe, Angelin Sablon Herinirina, Andre Das, Thanga Ganapathy, Yohesuwary Gunarasa, Kalyani Mariapan, Maathichsudhaar Muniandy, Mohd Firdauss Osman, Mohd Azem Fathi Mohammad Azmi, Jien Yen Soh, Wan Zainira Wan Zain, Andee Dzulkarnaen Zakaria, Zaidi Zakaria, Kheng Hooi Chan, Firdaus Hayati, Syamim Johan, Jin Jiun Mah, Ratha Krishnan Sriram, Sentilnathan Subramaniam, Nur Shahirah Binti Muhammad Shahimi, Khairul Hazim Hamdan, Mohd Razali Ibrahim, Guhan Muthkumaran, Ju Ann Tan, Mohana Raj Thanapal, Dayang Anita Abdul Aziz, Mae‐Lynn Bastion, Mohd Hairul Nizam Harun, Farrah‐Hani Imran, Norshamsiah Md Din, Ayesha Mohd Zain, Mushawiahti Mustapha, Syed Nabil, Ainal Adlin Naffi, Abd Jabar Nazimi, Roszalina Ramli, Bee Hong Soon, Pavin Bal, Joo Qing Cheng, Mohd Rusdi Draman, Ahmad Nazran Fadzli, Sakina Ghauth, WeiPin Hung, Suniza Jamaris, Peng Soon Koh, Sivakumar Krishnasamy, Yeong Sing Lee, Yew Toong Liew, Jeffery Zk Lim, Raymond Chung Siang Lim, Hiong Chin Lim, Siti Farhan Moh Pauzi, Shireen Anne Nah, Ashvin Krishna Nair, Doris Sin Wen Ng, Yuki Julius Ng We Yong, Kamarajan Ramayah, April Camilla Roslani, Rahmah Saaid, Anand Sanmugam, Mee Hoong See, Kanesh Kumaran Seevalingam, Neha Sethi, Srihari Singaravel, Syeda Nureena Syed Jafer Hussain Zaidi, Chu Yik Tang, Li Ying Teoh, Kian Boon Wong, Ruben Xavier, Ahmad Shuib Yahaya, Norhafiza Ab, Rahman, Islah Munjih Ab, Rashid, Mohd Fahmi Abd Aziz, Mohd Norhisham Azmi Abdul Rahman, Nasser Muhammad Amjad, Shahidah Che Alhadi, Faisal Elagili, Mohd Nazli Kamarulzaman, Azmi Md Nor, Ahmad Faidzal Othman, Mohd Yusof Sainal, Mat Salleh Sarif, Richelle Chua, Aini Fahriza Ibrahim, Abdul Rauf Bin Ahmad, Yii Ling Lau, Ka Yee Liew, Kandasami Palayan, Hui Yu Wong, Jasiah Zakaria, Nor Azimah Abd Aziz, Norazila Abdul Rahim, Noorneza Abdul Rahman, Bahiyah Abdullah, Mohamed Faizal Bin Sikkandar, Fatin Firman, Nik Mohd Hazleigh, Intan Kartika Kamarudin, Shahril Khalid, Mohd Fairudz Mohd Miswan, Akmal Zulayla Mohd Zahid, Norasyikin Mustafa, Kadhim Obaid, Mohd Yusoff Bin Yahaya Yahaya, Ahmad Ramzi Yusoff, Predrag Andrejevic, Jeremy Borg Myatt, Svetlana Brincat, Clifford Caruana, Kurt Lee Chircop, Francesca Chircop, Daniel Mario Chircop, Charles Cini, Viktoria Czok, Melanie Farrugia, Gabriella Grima, Fatimah MJ Hassan Almukhariq, Esther Muscat, Darryl Pisani, Mohsin Hassan Khan Roshan, Nicholas Schembri, Mohamed Shoukry, Neville Spiteri, Gilbert Tanti, Ziad Aboharp Hasan, Alberto Bazan Soto, Cedillo Rodrìguez Jonathan Rubén, Gustavo Esteban Lugo Zamudio, David Gerardo Miranda Gómez, José Alfredo Pérez Meave, Victor Pinto Angulo, Antonio Rojas Aguilar, Erik Efrain Sosa Duran, Alejandro Vàzquez Perez, Diego Arroyo, Jose Corona‐Cruz, Hector Martinez‐Said, Heriberto Medina‐Franco, Manuel Mendez, Teresa Moreno Y Suárez Ma, Dolores Orta Díaz, Martha Patricia Pérez de León Vázquez, Angélica Azucena Soto Carvajal, Fidel Soto Hernández, Juan Ignacio Stenner, Adriana Valenzuela López, Mauricio Rene Hernandez, Rey Jesus Romero, Aldo Bernal Hernandez, Ana Olivia Cortes‐Flores, Carlos Jose Zuloaga Fernandez del Valle, Carlos Andres Colunga Tinajero, Jaime Orozco‐Perez, Laura Gabriela Peña Balboa, Guillermo Yanowsky‐Reyes, Víctor Hugo Alcalá Torres, Francisco José Barbosa Camacho, Irma Valeria Brancaccio Perez, Miguel Angel Calderon‐Llamas, Aldo Camacho Gomez, Edgar‐Joaquin Cortes‐Torres, Esteban Cueva‐Martinez, Oscar Durán Anguiano, Isaac Esparza Estrada, Roberto Fierro‐Rizo, Paola Flores Becerril, Clotilde Fuentes Orozco, Miguel García Castillo, Alejandro Gonzalez Ojeda, Ana Sofia Gonzalez Rubio, Erick Alonso González‐García de Rojas, Luis Humberto Govea‐Camacho, Jose Aldo Guzman Barba, Juan Carlos Ibarrola Peña, César íñiguez Martínez, Francisco Javier Llamas‐Macias, Ruben Eduardo Morán Galaviz, Cesar Nuño‐Escobar, Oscar‐Everardo Olvera‐Flores, Jaqueline Osuna‐Rubio, Jose Victor Pérez‐Navarro, Rodrigo Prieto‐Aldape, Luis Ricardo Ramirez Gonzalez, Marco Vinicio Ramírez Sánchez, Emilio Reyes, Olaya Moramay Romero‐Limón, Carlos Beas Ruiz‐Velasco, José Antonio Sánchez Martínez, Guillermo Sanchez‐Villaseñor, Ismael Sedano‐Portillo, Gabriela Ambriz Gonzalez, Pedro Berrones Moreno Berrones Moreno, Ana Beatriz Calderon Alvarado, José de Jesús Cárdenas Barón, Araceli Hernández, Francisco Javier León Frutos, Edgard Efren Lozada Hernandez, Eduardo Morales Valencia, Karla Daniela Pérez, Francina Bolanos‐Morales, Julio Herrera‐Zamora, Patricio Santillan‐Doherty, David Omar Arriaga Zavala, Salvador Francisco Campos Campos, Lizette Carmona, Antonio Galindo Nava, Blanca Santos, Juan Roberto Torres Cisneros, Katya Bozada‐Gutiérrez, Jose Jesus Herrera, Mucio Moreno Portillo, Francisco Pérez López, Mario Trejo‐Avila, Carlos Valenzuela Salazar, Maria Regina Alvarez, Fernando Cordera, Marco Antonio de la Rosa Abaroa, Antonio Gómez‐Pedraza, Roberto Hernandez, Antonio Maffuz‐Aziz, José Antonio Posada, Carlos Robles Vidal, Jose‐Luis Beristain‐Hernandez, Blas Eduardo Quintero Sada, Nicolás Uxmal Soruco Heredia, Ernesto Anaya, Ricardo Arceo‐Olaiz, Bernardo Gutiérrez Sougarret, Sonia Lopez Flores, Hector Martinez‐Said, Francisco J. Ochoa Carrillo, Raymundo Alfredo Pérez Uribe, Ludwigvan Adriano Bustamante Silva, Arianne I Lupián‐Angulo, Christian Enrique Soulé Martínez, Francisco César Becerra García, Ernesto Javier Guerrero Casillas, Edgar Uribe, Nereida Esparza Arias, J. Mindy Hernández‐Nava, David Isla‐Ortiz, Javier Melchor‐Ruan, Esmeralda Romero Bañuelos, Rosa Salcedo‐Hernández, Diana Vilar‐Compte, Paola Zinser Peniche, Jorge L. Aguilar Frasco, Alejandro Alfaro‐Goldaracena, Gabriela Alejandra Buerba, Emma Castro, Carlos Chan, Ruben Cortes, Ismael Dominguez‐Rosado, Daniel Garay Lechuga, Miguel Herrera, Carlos Hinojosa, Gabriel Lopez‐Pena, Edgar Martos, Heriberto Medina‐Franco, Miguel ángel Mercado, Paulina Moctezuma Velázquez, Roberto Moguel, Mariano Oropeza, Emmanuel Peña Gómez Portugal, Oscar Emmanuel Posadas‐Trujillo, Noel Salgado‐Nesme, Oscar Santes Jasso, Catherine Sarre, Karla Yareli Hernandez Skewes, Cristian Jimenez, Roque Delfino Licona‐Meníndez, Guillermo Montiel, Alberto Navarrete‐Peón, Shannat Ortega, Fernanda Romero Lechuga, Oscar Roque, Fernando Sevilla, Rogelio González‐López, Roberto ángel Núñez‐González, José Antonio Ortega‐Jiménez, Antonio Alvarado, Alfonso Alvarez Manilla Orendain, José Alberto Atristain Pesquera, Diego Frutos, Felipe Garcia, Juan Antonio González León, Marlin Guzman, Dorihela Herappe, Jesús Giovanni Inzunza Miranda, Miguel Angel Jimenez Botello, Ricardo Lerma, Fabián Hilario Mendoza Pedraza, Monica Noguez Castillo, Jose Anatolio Resendiz, Oscar Alejandro Sánchez García, Laura Elena Fernandez Rios, Pamela Frigerio, Iset Garcia, Gloria Isela Mendoza Frías, Emmanuel Moreno, Raquel Quiroga, Alejandra Anzures Mendoza, Arantza Govela Hinojosa, Aldo Izaguirre, Luis Hdez Miguelena, Jose Manuel Luna Vazquez, Indalecio Barcelata Rodriguez, Raul Ramos Mange, Antonio Ramos‐De la Medina, Carlos Reyes Utrera, David Zarate Saenz, Miguel Angel Zavala Gonzalez, Francisco Andrade González, Ludivina Cortes, David Roman, Aaron Sanchez, Radu Gurghiș, Gheorghe Rojnoveanu, Marin Vozian, Petru Caraja, Gheorghe Croitor, Roman Mosneaga, Sarnai Erdene, Lundeg Ganbold, Sergelen Orgoi, Soyombo Orsoo, Altanchimeg Sainbayar, Erdene Sandag, Burmaa Sanjaa, Rita Ait Benhamou, Abdessamad El Azhari, Bennis Ghita, Sara Lhassani, Sidi Mamoun Louraoui, Mounir Rghioui, Sarra Saaf, Taha Gadouali, Iltimass Gouazar, Moniba Korch, Ahmed Letrache, Amina Louari, Anas Zamame, Samia Ousouss, Meryem Zekhnini, Hafni Abderrazaq, Wijden Abichou, Hamza Ahlaou, Amal Baicha, Salma Baroudi, Ghita Chaoui, Hind El Azzazi, Saja El Masaoudi, Chiraz Hassoun, Hajar Moujtahid, Anas Riyahi, Ouijdane Zaim, Narjiss Aji, Imane Ammouze, Yasser Arkha, Hajar Bechri, Meryem Benchekroun Belabbes, Zaineb Benslimane, Lina Boualila, Othmane Bouanani, Abdessamad El Ouahabi, Leila Essakalli Hossyni, Boulaadas Malik, Mohammed Yassaad Oudrhiri, Taha Ismail Sefrioui, Adam Tagmouti, Mustapha Bensghir, Abdelghafour Elkoundi, Othman Belarabi, Zakaria Houssaïn Belkhadir, Amine Benkabbou, Saber Boutayeb, Brahim El Ahmadi, Yassine El Bouazizi, Abdelilah Ghannam, Taha Kabbaj, Anass Mohammed Majbar, Raouf Mohsine, Amine Souadka, Badra Aliou Kone, Marcel Didier Ndayishyigikiye, Akutu Munyika, Fillipus Ndatewapo, Peter Ngungi Njuki, Philipp Plarre, Srihari Singaravel, Jenevive Abrahams, David W Borowski, Clara Jeketera, Kwasi Yeboah, John Tabiri Abebrese, Alfred Mureko, Pueya Rashid Nashidengo, Francis William Quayson, Justina Shikongo, Subarna Bhusal, Yam Dwa, Ashoo Joshi, Eva Berkeveld, Frank Bloemers, Alexander Borgstein, Suzanne Gisbertz, Sarah Mikdad, Mark van Berge Henegouwen, Roel Bakx, Marc Besselink, Daniel HL Lemmers, Anne‐Jasmin Roelofs, Paul van Amstel, Claire van Helsdingen, Ludi Smeele, Niels Harlaar, Frederik Jonker, Sjirk van der Burg, Justin Y. van Oostendorp, Saranda Ombashi, Tim van der Voort, Martijn van Geldorp, Lisanne Posma‐Bouman, Hans Donald de Boer, Annette Olieman, Henriette Smid‐Nanninga, Jean‐Paul P.M. De Vries, Rianne Hogenbirk, Schelto Kruijff, Milou Noltes, Pieter Steinkamp, Tyche Derksen, Josephine Franken, Steven Oosterling, Peter Nolte, Jelle van der List, Ian Alwayn, Okker Bijlstra, Andries Braat, Ruth Bulder, Michèle de Kok, Robin Faber, Ben Goudsmit, Jaap Hamming, Sven Mieog, Alexander Vahrmeijer, Fenna E.M. van de Leemkolk, Joost van der Vorst, Jan van Schaik, Merel Verhagen, Kim Albers, Larsa Gawria, Harry Van Goor, Michiel Warle, Tessa M. van Ginhoven, Charlotte Viëtor, Evert‐Jan Boerma, Lara Lallitsch, Donald Schweitzer, Wouter Leclercq, Julie Sijmons, Peter‐Jan Vancoillie, Joop Konsten, Maarten van Heinsbergen, Nicole Dekker, Frank den Boer, Julian Dimech, Kaveh Djamali, Carolyn Fowler, Naji Ghamri, Helen Houston, Reece Joseph, Astha Kantroo, Jin Kwun, Samuel Lie, Nicholas Lightfoot, Andrew MacCormick, Sue Olliff, Natasha Trilokekar, Daniel Wood, Aruna Ekanayaka, Chris Furkert, Asanga Nanayakkara, Divyansh Panesar, Rebecca Teague, Oliver Waddell, Lauren Bidois, Zoe Clifford, Brodie Elliott, Frank Frizelle, Tamara Glyn, Eric Lim, Oliver Lyons, Jared Mclauchlan, Ella Nicholas, Michael O’Grady, Justin Roake, Harsh Singh, Ramez Ailabouni, David Kieser, John Rietveld, Mohammed Alqassab, Mostafa Amer, Annie Chan, John Egbuji, Mairi Fullarton, James Haddow, Robyn Hawkins, Vui Teck Lu, Omar Mohyieldin, Thomas Oliver, Kai Sheng Saw, Ramanen Sugunesegran, James Wilkins, Esther Woodfield, Joseph Baker, Jitoko Cama, Xavier Field, Alamea Fulivai, Ruhella Hossain, Ben Kelly, Jasen Ly, James McKelvie, Vivek Meiyappan, Cindy Xin Yi Ou, Vidit Singh, Chris Varghese, William Xu, Alex Hedley, Nick McIntosh, Hinerangi Temara, Jake Aitken, Brytt Frunt, Cheyaanthan Haran, Max Hardie Boys, Prashant Lakshman, Esther Pinfold, Sarah Rennie, Sarah Cowan, Nigel Henderson, Jasmin King, Jessica Leary, Cameron Castle, Matt Gaston, Jeong Ha, Maira Haimona, Steffanie Jury, Zoe Lahood, Matthew Lim, Dillon MacIntyre, Josephine Mak, Annelise Neal, William Shelker, George Weeratunga, Liam Ferguson, Christopher Harmston, Chelsea L Heaven, Matthew James McGuinness, Imogen Watt, Henry Witcomb Cahill, Abdussemee Abdurrazzaaq, Hameedat Opeyemi Abdussalam, Ademola Adebanjo, Aminat Olayinka Ahmed, Opeyemi Busayo Borokinni, Olushola Kayode Fasiku, Usman Gwaram, Onyedika Okoye, Oluwole Olaomi, Ademola Adeyeye, Akinola Akinmade, Elizabeth Enoch, Samuel Fayose, Victor Kayode‐Nissi, Innih Kadiri, Stella Oduah, Julius Olaogun, Mohammed Abdull, Mohammed Kabir Abdullahi, Auwal Babayo Kwankiyel, Shahir Bello Umar, Kefas Bwala, Sulaiman Hassan, Abubakar Bappah Ja’afar, Umma Hani Ja’afaru, Saidu Kadas, Haruna Liman, Baje Makama, Aminu Mohammad Mohammad, Abdullahi Musa Kirfi, Funmilayo Oyediji, Husseini Yakubu Mohammed, Peter Agbonrofo, Oghenevwegba Akpoghor, Alexander Arekhandia, Oseyi Dawodu, Ekaniyere Edetanlen, Chizoba Efobi, Esezobor Egbor, Nosakhare Enaruna, Michael Ezeanochie, Ekene Ezenwa, Ferdinand Ijekeye, Joe Imumoren, Oseihie Iribhogbe, Omorodion Irowa, Moses Momoh, Nnamdi Nwashilli, Osarobo Obahiagbon, Ozoemene Obuekwe, Emeka Danielson Odai, Vincent Odigie, Oluwatomi Odutola, Amina Okhakhu, Stanley Okugbo, Samson Oni, Ngozi Onyeagwara, Eustace Oseghale, Osarenkhoe Osemwegie, Philip Osho, Veronica Ugwi, Charles Uwagboe, Sofiat Yusuf, Adekunle Abiodun, Olufemi Akinloa, Moses Shodipo, Alhassan Abdullahi, Akinwale Afolabi, Ishak Lawal, Chidiebere Peter Echieh, Chimaobi Nwagboso, Ubong Udoh, Bolaji Akanni, Solomon Anyimba, Isaac Chukwu, Sebastian Ekenze, Uchechukwu Ezomike, Ikponmwosa Gold, Ikenna Nnabugwu, Ikechukwu Nwafor, Louis Okolie, Kelechukwu Okoro, Blasius Okwara, Ndubuisi Onyemaechi, Khalifa Abdulsalam, Abayomi Arogundade, Mohammed Kura, David Nwosu, Habiba Abdullahi, Eyaofun Agida, Oseremen Aisuodionoe‐Shadrach, Hafees Ajibola, Godwin Akaba, Omolabake Ale, Terkaa Atim, Malachy Ayogu Emeka, Adeka Benard, Amina Buba, John Chinda, Stephen Edino, Sefiu Eniola, Stephen Garba, Aliyu Isah, Dennis Anthony Isah, Zaman Joshua, Ndubuisi Mbajiekwe, Philip Mshelbwala, Rafat Muhammad, Richard Offiong, Samson Olori, Anne Olute, Olabisi Osagie, Idoko Pius Ogolekwu, Bilal Sulaiman, Salisu Suleiman, Aminu Umar, Olukayode Abayomi, Rukiyat Abdus‐salam, Sikiru A. Adebayo, Amos Adeleye, Samuel Ademola, Oludolapo Afuwape, Akinlabi Ajao, Sanusi Akinsola, Timothy Aladelusi, Omobolaji Ayandipo, Lateef Ayodele Baiyewu, Mosimabale Balogun, Israel Daodu, Ifeanyichukwu Kelvin Egbuchulem, Peter Elemile, Constantine Ezeme, Ayotunde James Fasunla, Olalere Gbolahan, Ifeanyi Iwuagwu, Taiwo Akeem Lawal, Afieharo Michael, Jemiludeen Morhason‐Bello, Onyekwere Nwaorgu, Izegaegbe Obadan, Olubunmi Odeyemi, Omowonuola Ogundoyin, Olakayode Ogundoyin, Segun Ayodeji Ogunkeyede, Tolulope Ogunrewo, Oghenekevwe Okere, Titilola Oladejo, Ajibola Oladiran, Naomi Olagunju, Ifedolapo Olaoye, Olayinka Olawoye, Dare Olulana, Adeola Olusanya, Akinyinka Omigbodun, Paul Onakoya, Mathias Orji, Babatunde Osinaike, Oyeleye Oyelakin, Olugboyega Oyewole, Samuel Sule, Augustine O Takure, Ebere Ugwu, Adesoji Ademuyiwa, Bosede Afolabi, Peter Ajayi, Opeyemi Akinajo, Felix Alakaloko, Oluwole Atoyebi, Orimisan Belie, Chris Bode, Ihediwa Chibuike George, Olumide Elebute, Francis Ezenwankwo, Adedeji Fatuga, Oluwaseun Ladipo‐Ajayi, Ayomide Makanjuola, Christian Makwe, Bolaji Mofikoya, Ephraim Ohazurike, Rufus Wale Ojewola, Kehinde Okunade, Adeyemi Okunowo, Thomas Olagboyega Olajide, Oluwafemi Oni, Justina Seyi‐Olajide, Kehinde Tijani, Andrew Ugburo, Aloy Okechukwu Ugwu, Paul Abiola, Henry Abiyere, Idowu Adebara, Adebayo Adeniyi, Olabisi Adeyemo, Abimbola Ariyibi, Olakunle Babalola, Adewumi Bakare, Oluseyi Banjo, Peter Egharevba, John Obateru, Owolabi Ojo, Abiodun Okunlola, Adewale Olajide, Fatudimu Oluwafemi, Oluwole Oyeleye, Adedayo Salawu, Bakare Tajudeen Ishola Babatunde, Moruf Abdulsalam, Aderinsola Adelaja, Abimbola Adeniran, Olalekan Ajai, Kazeem Atobatele, Olabamidele Ayodele, Funmi Bello, Omolara Faboya, Olufemi Idowu, Jeuel Idowu, Muntaqa Mustapha, Olufunmilade Omisanjo, Roland Osuoji, Julius Vitowanu, Omolara Williams, Anthonia Abe, Adewale Aderounmu, AbdulHafiz Adesunkanmi, Adewale Adisa, Temitope Ajekwu, Samuel Ajekwu, Olalekan Ajiboye, Adeleke Akeem Aderogba, Olusegun Isaac Alatise, Mustapha Babatunde, Simon Balogun, Adebimpe Ijarotimi, Adedayo Lawal, Olugbenga Ojo, Olalekan Olasehinde, Adebayo Olugbami, Akaninyene Eseme Ubom, Funmilola Wuraola, Ademola Agbaje, Tajudeen Mohammed, Lukman Abdur‐Rahman, Abiodun Adeniran, Muideen Adesola, Ademola Adeyeye, Abdulrahman O. Afolabi, Isiaka Aremu, Iyehunwa Ayinmode, Jibril Bello, Grace Ezeoke, Sadiya Gwadabe, Gbadebo Ibraheem, Saidu Ibrahim, Munir’deen Ijaiya, Abdulrasheed Nasir, Peter Olalekan Odeyemi, Olukayode Ogunade, Afusat Olabinjo, Samuel Adegboyega Olatoke, Tope Olowogbayi, Stephen Onjefu, Ademola Popoola, Hadijat Olaide Raji, Nasiru Salawu, Hafeez Salawu, Tolulope Olayinka Sayomi, Segun Segun‐Busari, Kenechukwu Uche‐Okonkwo, Olajide Abiola, Olalekan Anipole, Olumuyiwa Ogunlaja, Oluwaseyi Adesina, Barnabas Alayande, John Ebenezer, Kenneth Enwerem, Christian Isichei, Mercy Isichei, Pwaluke Luku, Paul Omiragi, John Onyeji, Joel Adze, Ifeanyi Aghadi, Akawu Auta, Sharon Duniya, Joseph Gyuro, Stephen Kache, Jamila Lawal, Jerry Godfrey Makama, Garba Mohammed, Amina Mohammed‐Durosinlorun, Kabiru Salisu, Matthew Taingson, Nuhu Yusuf, Happy Amos, James Daniel, Danjuma Sale, Idris Usman Takai, Lawal Abdullahi, Sani Abdullahi, Mamuda Abdulrahman, Mohammed Kabir Abubakar, Valentine Adikaibe, Misbahu Ahmad, Jameel Ismail Ahmad, Sani Ali Aji, Mohammed Salele Aliyu, Lofty‐John Anyanwu, Maryam Babba Danagundi, Yusuf Halidu Bako, Nafisatu Bello‐Muhammad, Ibrahim Umar Garzali, Muhammad Ghazali Hasheem, Ismail Hassan, Sadiq Hassan, Mustapha Ibrahim Usman, Nasiru Ismail, Yasir Nuhu Jibril, Musbahu Kurawa, Mahmoud Kawu Magashi, Bardi Martins, Usman Mohammed Bello, Saminu Muhammad, Abubakar Bala Muhammad, Shamsuddeen Muhammad, Sulaiman Muhammad Daneji, Ibrahim Aliyu Mukhtar, Kabir Musa Adamu, Callistus Nwachukwu, Uchenna Kelvin Omeje, Ifeanyi James Orji, Abdurrahman Abba Sheshe, Ibrahim Suleiman, Sani Abdullahi Yunusa, A Maryam, Kabiru Bello Abubakar, Saudat Garba Habib, Farrukh Mahmood, Ibrahim Abolaji Alabi, Soliudeen Arojuraye, Mohammed Salihu, Samuel Alade, Felix Kumolalo, Chiedozie Mogbo, Olaolu Adebayo, Oluwasuyi Ige, Gbenga Jones, Tunde Odunafolabi, Chukwuma Okereke, Oluniyi Oyetunde Olubayo, Chris Akani, Datonye Alasia, Justina Omoikhefe Alegbeleye, Oti Nimi Aria, Abimbola Awopeju, Kemebradikumo Edonkumoh, Onyeanunam Ekeke, Sotonye Fyneface‐Ogan, Celestine John, Terhemen Kasso, Inye Korubo, Christie Mato, Rosemary Ogu, Charles Okpani, Anthony Okpani, Ijeoma Onwuagha, Ijeoma Oppah, Victory Oputa, Emmanuel Oranu, Ngozi Orazulike, Job Otokwala, Henry A.A Ugboma, Adekunle Ajayi, Ibukunolu Ogundele, Babatunde Salami, Okeoghene Ajagha, Yakubu Ali, Mudi Awaisu, Muhammad Daniyan, Lovely Fidelis, Stanley Emeka Nwabuoku, Oluseyi Ogunsua, Nasir Oyelowo, Tunde T. Sholadoye, Musliu Adetola Tolani, Alfa Yakubu, Venko Filipce, Vladimir Rendevski, Blagoj Shuntov, Natalija Cokleska Shuntov, Lazar Todorovic, Gordana Georgieva, Tomislav Jovanoski, Katerina Jovanovska, Bisera Nikolovska, Igor Peev, Sofija Pejkova, Blagoja Srbov, Andrijana Trajkova, Viktor Trenchev, Sasho Dohcev, Josif Janchulev, Skender Saidi, Oliver Stankov, Sotir Stavridis, Aleksandar Trifunovski, John George Massoud, Najib Abu Draz, Khalil Al Ajmi, Ghadeer Al Ajmi, Ayman Al Amri, Ghalib Al Badaai, Nawf Al Balushi, Zainab Al Balushi, Wameedh Al Bassam, Maather Al Farsi, Wadha Al Ghafri, Alaa Al Ghafry, Mohammed Al HOSNI, Moza Al Kalbani, Maha Al Kalbani, Lamya Al Kharusi, Safiya Al Kharusi, Mohammed Al Mutani, Hani Al Qadhi, Amani Al Raisi, Nihal Al Riyami, Hilal Al Sabti, Maha Al Shaibi, Rashid Al‐Abri, Abdullah Al‐Mujaini, Aya Al‐Rashdi, Suha Alajmi, Zamzam Albadi, Hanadi AlBusaidi, Ahmed Alhadeethi, Noor Aljabri, Muhannad Alkazrooni, Nadiya Alkharousi, Al‐Salt Alkharusi, Khadija ALKiyumi, Noof AlMaharbi, Sabaa Alsaadi, Marwa Alsharji, ALi Hamed ALSharqi, Fatma AlShehhi, Sareyah Alsibai, Thuwaiba Alsulaimani, Mustafa Alward, Mohammed Alwashahi, Hussain Alyafii, Adil Alzadjali, Tayseer Basi, Khadija Dalmar, Bashar Dawud, Mohamed Mourad Gargouri, Rajeev Kariyattil, Ahmed Omer Kenawy, Koshy Kochummen, Arif Kolethekkat, Mahesh Krishna Pillai, SureshKannan Ks, Farman Ali Laghari, Attibele Palaksha Manjunatha, Joseph Kunju Mathew, Rahil Muzaffar, Asha Nair, Hafsa Ismail Ibrahim Naiya, Zuhail Nazar, Silja Pillai, Ferdous Rahim, Khalid Rahman, Shahila Sheik, Mohammad Salman Siddiqi, Edwin Stephen, Shahzad Younas, Mishal Fatima, Ayesha Javed, Muhammad Ahsan Khan, Fatima Tuz Zahra Shakir, Riaz Hussain Siddiqui, Rizwan Sultan, Danish Syed, Ishtiaq Ur Rehman, Shahrukh Warraich, Humera Naz Altaf, Mohammed Amir, Saleha Yurf Asghar, Abu Bakar Hafeez Bhatti, Ali Haider Bangash, Sehrish Latif, Wajih Naqvi, Muhammad Tariq Abdullah, Saleha Abdullah, Arooj Ahmed, Muhammad Fahim Ahsan Ahsan, Fahad Akhtar, Sana Ali, Mehwish Ansar, Muhammad Aqib, Muhammad Arslan, Sumaira Ashraf, Azaz Ayubi, Tayyab Azam, Hamza Bhatti, Shahab Huda, Abeer Irshad, Nazia Ishaque, Tanwir Khaliq, Farheen Khan, Mumtaz Ahmad Khan Khan, Namrah Mahmood, Ali Raza Malik, Tahir Malik, Komail Malik, Manzer Mehmood, Fatima Mustafa, Fazal Noor, Sarwar Nubair, Isbah Rashid, Waqar Saeed, Hannan Sajid, Sajid Shah, Ali Shami, Minhaj Us Siraj Siraj, Mariam Tareen, M Burhan Ul Haq, Shahzad Hussain Waqar, Manzar Abbas, Syeda Maria Ahmad Zaidi, Iffat Ahmed, Shahnoor Ahmed, Kunwar Armash Ahsan, Munazza Akhtar, Ramsha Akhund, Adil Al‐Karim Manji, Hajra Arshad, Muhammad Ozair Awan, Aliya Aziz, Humaira Aziz, Shameen Bhutto, Faiqa Binte Aamir, Syed Raziuddin Biyabani, Uzma Chishti, Mohsin Chundrigar, Shayan Khalid Ghaloo, Waqqas Haroon, Shiraz Hashmi, Murtuza Hassan, Hina Inam, Nausheen Kassam, Abbas Kazmi, Majid Khan, Muhammad Osama Khan, Sameeta Kumari, Muhammad Jehangir Malik, Maheen Mansoor, Russell Seth Martins, Areeba Mubarik, Muhammad Musaab Munir, Muhammad Bazil Musharraf, Falak Naz, Shahryar Noordin, Arsalan Pervaiz, Javeria Bilal Qamar, M Fazlur Rahman, Pallavi Rani, Yasir Rasheed, Haleema Sadia, Marwah Saeed, Mohammad Safri, Shaharyar Salim, Abida K. Sattar, Shahzad Shamim, Maryam Sherwani, Muhammad Ahsan Iqbal Siddiqui, Astad Sidhwa, Javeria Tariq, Isbaah Tejani, Usama Waqar, Fahad Zahid, Abid Jamal, Asad Ali Kerawala, Muhammad Ali Ghufran, Amjad Siraj Memon, Lajpat Rai, Summaya Saeed, Khursheed Ahmed Samo, Mariyah Anwer, Manisha Aswani, Shahneela Manzoor, Shoaib Muhammad, Muhammad Arshad, Syeda Namayah Fatima Hussain, Inshrah Moin, Muhammad Kazim Rahim Najjad, Manal Nasir, Osama Bin Sohail, Ali Mangi, Naveed Markhand, Hassan Mushtaq, Aiman Tariq, Kashif Zia, Hassaan Ahmed, Nasir Ahmed, Talha Ahmed Qureshi, Shafqat Ali Shaikh, Rabia Anwer, Owais Arshad, Bushra Ayub, Sehrish Batool, Muhammad Chatni, Tariq Chundrigar, Samina Saleem Dojki, Minahil Elahi, Bakhtawar Farooqui, Amna Ghouri, Nabeel Hassan, Zainab Hussain, Hussaini, Fizza Iftikhar, Faizan Iqbal, Anum Javed, Dania Javed, Wajahat Kamal, Shifa Khan, Zeeshan Uddin Mughal, Ghulam Murtaza, Areeba Nadeem, Areeba Nasir, Fatima Nayyer, Kanwal Nisa, Yaseen Rauf, Umer Saeed Haroon, Shah Masabat Saleem, Raza Sayyed, Haseeb Seriwala, Aaqil Shah, Safia Siddiqui, Asher Tanweer Tanveer, Nida Tariq, Junaid Zaman, Mehroz Zamir, Sami Ullah Niazi, Muhammad Ayyub Anjum, Muhammad Yasir Naseem, Ameer Afzal, Ehsan Ahmed, Jawad Ahmed, Shujat Ahmed Riaz, Ali Akbar, Bilal Akbar, Maria Ali, Imran Ali Ali, Abrar Ashraf Ali, Ghazanfar Ali, Zahra Altaf, Ahmed Siddique Ammar, Hafiz Muhammad Arif Arif, Imran Aslam, Muhammad Mustehsan Bashir, Usman Farooq, Muhammad Farooq, Khalid Masood Gondal, Muhammad Javed Iqbal, Zafar Iqbal, Fahad Ismail, Muhammad Khurram Jameel, Muhammad Haris Janjua, Azwa Janjua, Prabesh Joshi, Mamoona Khadam, Romaisa Khan, Muhammad Asadullah Khawaja, Sobia Manzoor, Muhammad Mohsin, Khizra Mumtaz, Muhammad Musaab, Syed Asghar Naqi, Fatima Naumeri, Umer Nazir, Sadaf Noureen, Yaseen Rafi, Ali Raza, Sushil Rijal, Irfan Saleem, Muhammad Zeeshan Sarwar, Hafiz Syed Zaigham Ali Shah, Muhammad Sharif, Ayesha Shaukat, Usman Siddique, Hafiz Saqib Sikandar, Mohammad Sohail, Fatima Tu Zahara, Yasmeen Usman, Balakh Sher Zaman, Misbah Zeb, Muhammad Farooq Afzal, Fateen Ahmad, Waqas Ahmed, Shahzad Akram, Azhar Alam, Danish Ali, Ahsan Ali, Ammar Tariq Alvi, Suleman Asif Asif, Madiha Aslam, Muhammad Daood Daood, Muhammad Jawad, Usama Muhammad Kathia, Ismaeel Khalid, Anwar Khan, Muhammad Imran Khokhar, Zain Khurshid, Hamza Noman, Ahsan Raza, Hassan Taha, Muhammad Zubair, Noor Ul Huda Maria, DrAtif A Janjua, Qamar Ashfaq Ahmad, Sheraz Ahmad, Imran Ali, Sana Amin, Momina Anjum, Muhammad Asjad, Aatif Aslam, Mahmood Ayyaz, Luqman Ali Bajwa, Akkasha Bakhtiar, Alia Bashir, Fizza Batool, Samiullah Bhatti, Roshan Butt, Usman Butt, Shabbar Hussain Changazi, Omer Iqbal Cheema, Muhammad Haris Chishti, Bushra Haq, Ali Hashmi, Muhammad Hassan, Usman Hassan, Khizar Hayat, Javaid Iqbal, Ayesha Iqbal, Amna Ishaq, Sundas Javed, Hina Javed, Aymen Javed, Muhammad Kashif, Muhammad Saud Khalid, Wasim Hayat Khan, Rohma Khan, Fatima Khan, Usman Latif, Arif Mahmood, Faizan Majeed, Kiren Malik, Rumaisa Masood, Fasiha Munawwar, Asma Mushtaq, Muhammad Shoaib Nabi, Ahmad Nasir, Shazia Naureen, Zahida Noreen, Muhammad Ghulam Qadir Qadir, Ahmad Uzair Qureshi, Gul e Raana Raana, Madeeha Rashid, Abdul Rehman, Sidra Riaz, Arfa Saboor, Muhammad Hamza Sadiq, Komal Saeed, Humaira Saleem, Kiran Sarfraz, Zahid Shafiq, Aymen Shafqat, Hassan Shaukat, Fateh Sher, Rubina Sohail, Amina Suhail, Bilal Syed Muhammad, Usama Umair, Muhammad Umar, Muhammad Usama, Natasha Usman, Kalsoom Waheed, Muhammad Waris Farooka, Shafqat Wasim, Tayyiba Wasim, Fahad Yasin, Zain Zafar, Imdad Ahmad Zahid, Sabahat Zaka, Salma Zaman Zaman, Junaid Azad, Zareen Fatima, Muhammad Imran Anwar, Haroon Javaid Majid, Muhammad Ahmed Naseer, Muhammad Taimoor Shah, Ammar Ali, Muhammad Aslam, Muhammad Usama Aziz, Farhan Gohar, Waseem Humayoun, Hassan Khan, Shiza Naeem, Warda Tahir, Nabila Talat, Kashif Alvi, Muhammad Asif, Maria Mehmood, Muhammad Ali, Muhammad Hamid Chaudhary, Aniqa Shahzad, Muhammad Sher, Murtaza, Hijab Siddique, Waleed Anjum, Umer Farooq, Manzoor Ahmad, Siddique Ahmad, Muhammad Alam, Fouzia Faizi, Shehla Faridoon, Musarrat Hussain, Muhammad Iftikhar, Yousaf Jan, Muhammad Kalim, Tilal Ahmed Raza, Farrukh Ozair Shah, Muhammad Shah, Imran Tahir, Hamza Khan, Waleed Mabood, Muhammad Naeem, Shabir Ahmad, Haseeb Ahmed, Jawad Ahmed, Hazrat Akbar Akbar, Muhammad Shaheer Akhtar, Noman Ali, Mehboob Ali, Sadaf Ali, Irfan Ali, Hasbi Allah Amin, Shehzadi Amin, Muhammad Asif, Bakhtawar Ayub, Muhammad Basir, Syed Imran Bukhari, Ahmad Faraz, Muhammad Salman Farsi, Ambrin Gul, Hina Gul, Ayaz Gul, Sana Gul, Sajjad L Haider, Syed Ikramullah Ikramullah, Kashif Jan, Muhammad Khalid, Salman Khan, Muhammad Faisal Khan, Faraz Ahmad Khan, Iftikhar Mian, Aiman Moeen, Obaid Mohmand, Rehana Rahim, Naila Raziq, Riaz RehmAn, Azra Salim, Tanveer Shafqat, Faaiz Ali Shah, Suliman Shah, Muhammad Shakeel, Tahsin Ullah, Shams Ur Rehman, Sabaeena Wali, Farnaz Zahoor, Gulmeena Zeb, Laila Zeb, Haseeb, Waqas Raza, Muhammad Tariq, Zein el Amir, Daneyal Arshad, Mashal Daud, Umer Javed Chughtai, Mirza Hammad Rauf, Zaina Sajid, Rubaba Abid, Ayesha Azam, Tallat Farkhanda, Kanwal Firdos, Tabinda Khalid, Sobia Malik, Javeria Mashal, Ruqyyah Salim, Madiha Saqib, Aneela Shaheen, Madiha Shahid, Irfana Yasmeen, Faryal Ahmed, Aneeqa Ali, Zarafshan Amjid, Muhammad Idrees Anwar, Iqtaza Arif, Anam Ashraf, Adam Umair Ashraf Butt, Faryal Azhar, Muhammad Daniyal Daniyal, Ramlah Ghazanfor Ghazanfor, Daniyal Ishtiaq, Manahil Jamil, Saad Javed, Ziaullah Khalid, Jahangir Sarwar Khan Khan, Muhammad Sarfraz Khan, Usama Mahmood, Ashraf Mahmood, Abdur Rehman Malik, Ramisha Naseer Nagra, Fuad Ahmad Khan Niazi, Maleeha Saleem, Bushra Saleem, Tayyaba Saleem, Raja Khalid Shabbir, Imtiaz Ahmed Shakir, Eesha Yaqoob, Ashfaq Ahmad, Surgeon Hafiz Riaz Hussain Awan, Muhammad Usman Malik, Waqar Bhatti, Azka Shaheen, Hassan Nawaz Yaqoob, Hadeel Awad, Balqees Mohamad, Dalia Wafi, Mustafa Abu Jayyab, Hitham Toman, Hamdoon Abu‐Arish, Ibrahim Al‐Slaibi, Hussam I. A. Alzeerelhouseini, Fahmi Jobran, Raghad Lahlooh, Sadi A. Abukhalaf, Fatima Aburayyan, Khalil Abuzaina, Ayat A. Aljuba, Roaa Aljunaidi, Tareq Alzughayyar, Adham Amro, Sarah Amro, Mays Dweik, Safa Halman, Qutaiba Qafisha, Farah Shaheen, Balqis Shawer, Daleen Shehadeh, Jihad Zalloum, Khaled Demyati, Mohammed Hajhamad, Zain Saqfalhait, Emmy Arrue Del Cid, Moises Cukier, Homero Rodriguez‐Zentner, Francisco Abed, Rafael Osmar Adorno Garayo, Ruben Aguilar, Ana Aguilar, Domingo Javier Aguilera Maidana, Diego José Almada Casañas, Teresa Almiron, Analia Arrua, Pedro Avila, Brenda Balmaceda, Alexandra Barreto, Analy Barreto Galeano, Belinda Barrientos Nuñez, Magdalena Benegas, Martin Berden, Amalia Bogarin, Jorge Bonnin, Edulfo Britez Barrios, Julio Burgos, Carlos Fabián Cárdenas Melgarejo, José De Jesus Casco Samudio, Sandra Cataldi, Jorge Augusto Centurion, Luz Maria Cespedes Ramirex, Katerine Colina, Gabriel Augusto Cuevas Almando, Karina De Bleecker, Antonella Dragotto, Eiji Eiwa, Juan Carlos Enciso, Carla Espinola, Lujan Estigarribia, Celso Ariel Fernandez, Rocio Ferreira, Blas Figueredo, Daniel Fleitas, Claudia Fretes, Martin Fretes, Walter Rene Fretes Gonzalez, Humberto Nicolas Galleano Ruiz, Julio Gauto, Claudia Gauto, Viviana Gomez, Nadia Gómez, Osvaldo Insfran, Liz Karina, Christian Legal, Oscar Llanes, Walter Martínez, Walter Rodrigo Martínez Torres, Kiichiro Matsumura, Esteban Daniel Mendoza Galván, Leila Mereles Noguera, Sergio Mora, Daniel Navarro, Lidia Noguera Roman, Analia Núñez, Lorena Ocampos, Alma Maria Ojeda Rojas, Derlis Joel Ojeda Villasboa, Juan Palacios, Jessica Pamela Portillo Sosa, Rossana Ramírez, German Ramos, Diego Samudio, Jorge Eduardo Sisa Acosta, Rodney Tellez, Porfiria Urquhart, Beatriz Vera, Jorge David Vera Florentin, Daniel Vidallet, Gilce Teresita Villalba, Jacob Vömel, Laura Aquino, Marisa Natalia Martinez, Cesareo Saldivar Patiño, Omar Aguayo, Maria Esther Ferreira Aguilera, Federico García, Maria Giangreco, Derlis Osorio, Cynthia Marlene Rodríguez Sosa, Paulo Villanueva, Sandra Elizabeth Centurion Rolon, Rozana Ohara, Juan Marcelo Portillo, Ruben Milciades Varela Cano, Juan Manuel Baez Melgarejo, Francisco Vidal Candia, Carlos Chirico, Sofia Belen Diaz Pineda, Diego Alfonso Paiva Vera, Ayrton Facundo Valdovinos, Dahiana Fatima Velazquez, Diego Raul Abente Arriola, Analia Britos, Daniel Cantero, Jessica Centurión, Celso Ariel Fernandez, Ricardo Ignacio Olmedo Bareiro, Dayhana Duarte, Susana Paredes, Carlos Pfingst Rojas, Angel Luis Agüero Delgado, Juan Marcelo Delgado Godoy, Helmut Alfredo Segovia Lohse, Oscar Benitez, Victor Luraschi, Joel Molinas, Dulce Añasco, Alejandro Arévalo Barreto, Cristhian Chavez Rivaldi, Martin Chenu, Luz Garcia, Rosana Godoy, V Gustavo Miguel Machain, Maria Jose Martinez Velázquez, Brayan David Pedroso Alvarenga, Agustin Rodriguez Gonzalez, Rosa Sánchez, María Liz Sánchez, Herald Rene Segovia Lohse, Cesar Giuliano Sisa Segovia, Osmar Cuenca, Sara Melgarejo, Ever David Sosa Ferreira, Glenda Marina Falcon Pacheco, Estefany Angela Flores Anaya, Evy Yamil Huaman Huallanca, Karla Melissa Marchan Palma, Robinson Mas Melendez, Liliam Palomino, Arazzelly Del Pilar Paucar Urbina, Jorge Ordemar, Manuel Quiroz, Jose Rios Chiuyari, Victor Diego Caballero Sarabia, Carlos Eduardo Otiniano Alvarado, Lorena Fuentes Rivera Lau, Christian Lozano, David Torres, Henry Rafael Acosta Castro, Gabriel Bayona‐Alvarado, Giuliano Borda‐Luque, Joao Cruz, Grecia Lizzetti, Victor Vasquez Morales, Milagros Niquen‐Jimenez, Martin Ormeño, Liz Ortiz, Kewin Quispe de la Roca, Darwin Artidoro Quispe‐Cruz, Edgar Sanz, Sebastian Shu Yip, Katherine Tataje Poma, Ximena Paola Vasquez Ojeda, Gaby Susana Yamamoto Seto, Claudia Arias, Carlos Palacios, Juan Francisco Pintado Mejia, Jose Maria Silva Barandiaran, Eduardo Huaman, Guillermo Enrique Reyes Gamonal, Claudia Suazo Carmelo, Sergio Zegarra, Jenner Betalleluz Pallardel, Diana Lizet Cruz Condori, Jose Vasquez Valverde, Eduardo Huaman, Regina Amparo Ugarte Oscco, Cesar Alberto Vergel Cabrera, Consuelo Diaz, Gian Mendiola, Angie Paredes Caturiny, Adolfo Salazar, Joel Jesús Sánchez Estupiñan, Marco Sandoval Vaez, Roy Arangoytia, Francisco Berrospi, Jorge Luis Bustamante Polo, Jimmy Cárdenas Coaquira, Fernando Cárdenas Escalante, Darwin Oliver Desposorio Armestar, Sheyla Katherine Diaz Mora, Jheff Laura, Aldo Lopez Blanco, Karoll Tatiana Meza Garcia, Jaime Moreno, Eduardo Payet, Joan Perez Villena, Daniel Cardenas, Yahaira Tatiana Carpio Colmenares, Luis Alcides García Barrionuevo, Miguel Roberto Li Valencia, Percy Mansilla Doria, Fiorella Palomino Escalante, Mónica Falcón Coronado, Carlos Shiraishi Zapata, Pamela Valderrama Rocha, Ulbar Edinson, Maria Rosa Ortiz, Vladimir Laureano Velasquez Huarcaya, Andrei Cesar Abella, Ma, Carmela Andal, Willmar Anoso, Monica Maria Bejasa, Aldrin Cuasay, Jesus Fernandez, Eunice Mercado, Amabelle Moreno, Alejandro Palines, Trina Regalado, Cristeta Unira, Clarence Pio Rey Yacapin, Carlo Angelo Cajucom, Mary Anne Carol Cueto, Charmagne Loren Ramos, Avril David, John Isaac Merin, Alberto Roxas, Nipseey Pauloe Candelario, Marc Paul Lopez, Ramon Alberto Ramos, Khrissa Lea Elisa Violago, Yvann Kenneth Benosa, Louie Czelline De Leon, Marlow Esguerra, Rene Luis Filarca, Corina Gaddi, Patrick Jovan Gagno, Jeffy Guerra, Angelica Aubrey Morla, Rivero Opano, Edgardo Penserga, Mark Jheric Tesil, Hazel Turingan, Joel Aldana, Alyssa Carmina Almelor, Jaime JR Almora, Angeli Anne Ang, Maria Angelica Arada, Jeric Arbizo, Diana Barretto, Ronnie Baticulon, Kevin Ivan P. Chan, Elmer Jason Cruz, Pauleen de Grano, Juan Gabriel De Leon, Jojiemar De Pano, Maria Angela Dealino, Daryl Anne del Mundo, Hans Jesper Del Mundo, Czarlo Dela Victoria, Juan Miguel Roberto Delgado, Jan Erik Detran, Robert Zaid Diaz, Edward Arlu Dinoy, Efren Domingo, Heather Grace Dulnuan, Dumlao Patricio, Carlo Elises, Emmanuel Estrella, Joan Marie Flor, Grace Gana, Bynlee Stuart Go, Ana Melissa Hilvano‐Cabungcal, Jacinto Cesar, Jan Alexeis Lacuata, Marie Carmela Lapitan, Renee Leen Laudato, Gerardo Legaspi, Ryle Li, Liana Mae Lobo, Claudine Lukban, Christopher John Macapugay, Nikko Magsanoc, Enrique Manalang, Jon‐Alexis Montemayor, Jaysonnel Notario, Marie Dione Sacdalan, Jan Armelynn Santos, Abigail Sarmiento, Dennis Serrano, Samantha Siahetiong, Krisniel Florence Solis, Denorson Sotalbo, Erik Tongol, Germaine Ursabia, Patricia Ann Uy, Charmaine Grace Valeros, Karen Joyce Velasco, Vincent Venida, Hans Edison Yap, Mario Angelo Zamora, Anika Johanna Agoncillo, David Vincent Antonio, Nicole Robyn Bangayan, Marinelle Castro, Grace Lynn Estanislao, Anne Kathleen Ganal‐Antonio, Faiyazudin Amado Ibrahim, Peter Jarin, Laine Valerie Kongsun Ching, Josephine Alexandra Lim, Mariles Nazal, Amanda May Ong Vaño, Louie Racelis, Kirsten Ramos, Jeryl Anne Silvia Reyes, Ron Daniel Rivera, Jose Antonio Salud, Elva Marita Sarte, Dale Kingsley Sy, Eileen Grace Tancinco, Anna Mariel Torio, Avegail Niña Uy, Ryan Rainiel Abary, Dabalos Jesus, Vincent Siquian, Kenneth Yabut, Jose Carlos Barcelon, Ma, Inez Carballo, Jo Ann Chiu, Aeris Jane D. Nacion, Piotr Major, Michał Pędziwiatr, Justyna Rymarowicz, Marcin Bobiński, Jan Kotarski, Karolina Rasoul‐Pelińska, Konrad Futyma, Pawel Miotla, Ewa Rechberger, Bogdan Koczy, Karol Szyluk, Pedro Botelho, Mariana Branco Lopes, Brigitta Cismasiu, Paulo Matos Costa, Jean‐Michel Fallah, Gonçalo Teodoro Fernandes de Freitas, Filipa Ferreira, José Guilherme Gonçalves Nobre, Susana Henriques, Rui Lino, Alexandre Macedo, Cláudia Mendes, Ana Lúcia Preto Barreira, Nuno Ramos, Susana Candeias Rodrigues, Catarina Rolo Santos, Ricardo São Pedro, Melissa Silva, Carla Sousa, Rui Ribeiro, José Santos, Octavio Viveiros, Ricardo Alves, Pedro Serralheiro, Tobias Teles, Liliana B. Sousa, Dulce Helena Ferreira de Carvalho Carvalho, Rui Costa Soares, Cristina Costeira, João Graveto, Daniel Martins Jordão, Filipe Paiva‐Santos, Pedro Parreira, Anabela de Sousa Salgueiro Oliveira, Paulo Santos‐Costa, Constança Azevedo, Daniela Machado, Filipa Mendes, Miguel Semião, Joana Bolota, Ana Margarida Cinza, Manuel Damasio Cotovio, Sofia Leandro, Joana Oliveira, Rita Pedroso de Lima, Mário Pereira, Miguel Rocha Melo, Cristina Velez, Paulo Cardoso, Nicole Cardoso, Joana Cristina Domingues, Pedro Henriques, Maria Isabel Manso, Ruben Martins, Gildasio Martins dos Santos, Henrique Morais, Rute Pereira, Tatiana Revez, Vera Inês Ribeiro, Ricardo Ribeiro, Ana Soares, Sandra Sousa, João Teixeira, José Azevedo, Ana Cabral, Catarina S. Rodrigues, Paulo Alves, Gonçalo Ferreira, Rita Gonçalves Pereira, Miguel Neves, Nuno Rama, Inês Sousa, Mafalda Almeida, Ricardo Bettencourt Morais, Ana Campos, Cristina Caroça, Mariana Couto Bártolo, Luis Galindo, Ricardo Girao, José Pais, Nelson Silva, Carlos Vaz, Jose Henrique Albuquerque Messias, Francisca Brito da Silva, André Caiado, Filipa Fonseca, Sandra Maurício, Inês Peyroteo, Rodrigo Ramos, Inês Salgado, Patrícia Santos, Joana Patena Forte, Maria Luís Sacras, Ema Santos, Pedro Azevedo, Beatriz Costeira, Filipe Neves, Susana Ourô, Marisa Peralta Ferreira, Mafalda Sousa Fernandes, Joana Isabel Almeida, João Almeida Pinto, Rui Almeida‐Reis, Tiago Correia de Sá, Marcelo José Maia Azevedo Costa, André Dias, Vânia Fernandes, Inês Ferraz, Catarina Gil Gil, álvaro Gonçalves, Leticia Lima da Cruz, Catarina Lima da Silva, Luciana Lopes, Nuno Machado, Joana Marialva, Margarida Nunes Coelho, José Pedro, Cristiana Pereira, Ana Ribeiro, Rui Santos, Pedro Saraiva, Rita Silva, Francisca Tavares, Maria Teixeira, Nuno Teixeira, Bernardo Teixeira, Pedro Valente, Edgar Amorim, Odilia Conde, Miguel F. Cunha, Beatriz Dias, David Dias, Ana Fazenda, José Pedro Fernandes dos Santos, Filipe Isidro, João Pedro Melo Neves, Juan Rachadell, Inês Isabel Sampaio da Nóvoa Gomes Miguel, Liliana Sequeira, José Davide, Eurico Emanuel de Vale Gonçalves de Castro Alves, João Oliveira, Jorge Santos, Marisa Domingues Santos, Donzília Sousa Silva, Vítor Valente, Alexandre Almeida, Juliana Almeida Rego, Vítor Alvarenga, Duarte Nuno Amaro, Joao Barbosa‐Breda, Diogo Barreiro, Pedro Cabeça Santos, Carolina Carreiro, Sara Castanheira Rodrigues, Ana Salomé Cavaleiro Leitão de Carvalho, Bernardo Correia, Francisca Costa, Vítor Devezas, Nuno Dias, Carlos Silva Faria, Sara Fernandes, Joaquim Ferreira, Fabio Gomes, Cristina Granja, Filipa Jácome, João Lixa, Ana Magalhães, Mariana Magalhães Maia, Rita Martins, Mafalda Martins Sousa, Hugo Meleiro, Margarida Mendes, João Moreira, Joana Paiva, André Pereira, António Pereira‐Neves, Teresa Pina‐Vaz, Gabriela Pinheiro, Sílvia Pinho, Carina Ramos, Pedro Ramos, Patricia Ramos, Margari da Ribeiro, João Rocha‐Neves, Hugo Santos‐Sousa, Helena Silveira, Carolina Soares‐Aquino, Ana Rita Teles, Susan Vaz, Paula Vieira, Rodrigo Vilares Morgado, Luciana Cidade Costa, Marco Santos, Catarina Baía, Rita Canotilho, Ana Margarida Correia, Ana Paula Ferreira Pinto, Jose Flavio Videira, Alberto Abreu da Silva, Mariana Claro, Daniel Costa Santos, Ana Cláudia Deus, João Vaz Grilo, Carlos Boto, Rita Branquinho, Sónia Rodrigues, Fernanda Alves, Marlene Andrade, Carlos Baltazar Branco, António Castanheira, Cátia Ferreira, Gonçalo Guidi, André Guimarães, Clara Leal, Joana Lisboa, Luís Machado, Rita Marques, Prescillia Marques, Daniela Martins, Ana Melo, Mariana Morais, Cátia Moreira, Clara Mota, Mário Moura, Sara Nunes, Antonio Oliveira, João Pedro Reis, Inês Sá, Rita Sapage, Sílvia Silva, Patrícia Sousa, Rita Sousa, Diogo Sousa, Nádia Tenreiro, Ricardo Vaz Pereira, Bruno Vieira, Susana Lima Oliveira, José Pinto, André Tojal, Samir Abdalla, Soliman Alkassem, Mhamed Samer Alkhatib, Fahad Aurif, Fayez Bin Omran, Nasir Muzaffar, Sheraz Abayazeed Ahmed, Abdelrahman Abdelaal, Shereen Aboutaleb, Abdelrahman Abusabeib, Ahmad Abutaka, Khalid Ahmed, Ayman Ahmed, Mahmood AL Dhaheri, Mohammed Al Dosouky, Salim Al Lahham, Mohannad Al‐Tarakji, Saif Almudares, Artefaa Alshamari, Ibrahim Amer, Saif Badran, Ahmed Elaffandi, Mohamed Elakkad, Walid Elmoghazy, Mohamed Ghali, Sugad Mohamed, Muhammad Muslim, Munzir Obaid, Amjad Qabbani, Ahmed Faidh Ramzee, Mohammad Sameer, Amjad Shah, Ibnouf Sulieman, Ali Toffaha, Siddig Abdalla, Mohamed Abdelkareem, Malik Shakeel Ahmed, Omer Al‐Yahri, Mohammed Aljanabi, Hatem Kamkoum, Mohammad Burhan Khan, Mazin Khattabi, Ejaz Latif, Musab Murad, Shameel Musthafa, Iqbal Rasool Wani, Rania Berrami, Arnold Marchis, Mircea Hogea, Liviu Mugurel Bosinceanu, Victor Costache, Andreea Costache, Rad Costel Claudiu, Tudor Ștefan Dumitrescu, Silviu Tiberiu Makkai‐Popa, Bogdan Moldovan, George‐Ovidiu Muntean, Pisica Radu‐Mihai, Codin Saon, Vlad Silaghi, Aurel Mironescu, Adrian Papa, Lucian Vida, Cosmin Bezede, Andrei Chitul, Florin Grama, Valentin Calu, Octavian Enciu, Adrian Miron, Elena Adelina Toma, Sergiu Catalin Baraian, Cezar Ciubotaru, Bogdan Diaconescu, Florin Iordache, Alexandru Neagu, Ionut Negoi, Cristina Niculae, Bogdan Stoica, Cosmin Balan, Serban Ion Bubenek Turconi, Daniela Filipescu, Raluca Goicea, Mihai Stefan, Liana Valeanu, Nicolae Bacalbasa, Irinel Popescu, Dima Simona, Vlad Alexe, Olivia Batog, Mihnea Davidescu, Madalina Iliescu, Veronica Manolache, Natalia Motas, Sebastian Ionescu, Gabriela‐Mariana Militaru, Andreea‐Madalina Serban, Octav Ginghina, Razvan Valeriu Iosifescu, Mara Mardare, Radu Mihail Mirica, Andrada Spanu, Andrei Bogdan Văcărașu, Radu Costea, Lucian Negreanu, Nicoleta Aurelia Sanda, Narcis Octavian Zarnescu, Eugenia‐Claudia Zarnescu, Alexandra Beuca, Ciprian Cucoreanu, Andras David, Radu Drasovean, Ioan‐Alexandru Florian, Ioan Stefan Florian, Catalin Magan, Apostu Raluca‐Cristina, Radu Razvan Scurtu, Andrei Trif, Eduard Acatrinei, Patriciu Achimas‐Cadariu, Tareg Al Momani, Miruna Babut, Cristina Bujoreanu, Bordianu Christian, Alma‐Andreea Corpodean, Sergiu Crivat, Cătălin Dumitraș, Dan Tudor Eniu, Delia Florean, Ignat Florin, Vlad Alexandru Gata, Cojocaru Ion, Alexandru Irimie, Muresan Iulia Andrada, Patricia Jako, Katinka Kallos, Gabriel Lazar, Cosmin Lisencu, Puscas Marius‐Emil, Sergiu Matei, Mihnea Bogdan Borz, Ioan Milotoiu, Dragos Stefan Morariu, Maximilian Muntean, Narita Nadia Maria, Codrut Cosmin Nistor‐Ciurba, Alex Orădan, Alexandru Petrusan, Bogdan Petruț, Madalina‐Claudia Pocol, Irina‐Maria Puscas, Alin Rancea, Dragan Serghei, Elena‐Bianca Stănică, Cristina Suciu, Stefan Titu, Calin Tohatan, Evelyn Vanea, Schitcu Vlad, Ioan Catalin Vlad, Stelian Mogoanta, Carmen‐Aurelia Mogoanta, Stefan Paitici, Mihail‐Gabriel Dimofte, Sorinel Lunca, Ana‐Maria Musina, Cristian Ene Roata, Cristian Sandu, Natalia Velenciuc, Drochioi Ilie Cristian, Vlad Porumb, Mikhail Kirov, Vsevolod Kuzkov, Igor Zabaldin, Yegor Molitvin, Makhmud Ramazanov, Shamil Yuzbekov, Roman Bilenko, Veronika Budyakova, Anokhin Evgeny, George Gendrikson, Gunko Irina, Stoian Iudin, Anna Kapustina, Ilya Karpov, Mikhail Kurtenkov, Andrey Litvin, Oksana Matanova, Polina Melashenko, Artem Pobelenko, Arina Provozina, Vladimir Durleshter, Pavel Markov, Vadim Pykhteev, David Gorin, Ayrat Kaldarov, Andrey Kriger, Alexandr Burmistrov, Eduard Galliamov, Grigoriy Gololobov, Abdula Magomedaliev, Mirzemagomed Pirakhmedov, Erin Sergey, Nazir Yurkuliev, Григорий Карсотьян, Алексей Погонин, Магомедсалам Эльдерханов, Vladimir Balaban, Sergey Barkhatov, Yuliya Churina, Aleksandr Derinov, Sergey Efetov, Ksenia Feoktistova, Yury Kitsenko, Viktor Kochetkov, Grigory Korolev, Kirill Lazarev, Yuliya Medkova, Valery Nekoval, Pavel Pavlov, Sergey Rodimov, Irina Rudenko, Alexandra Shlomina, Darya Shlyk, Ludmila Sidorova, Petr Tsarkov, Petr Tsugulya, Inna Tulina, Victor Zhurkovskiy, Albina Zubayraeva, Ярослав Краснов, Mikhail Agapov, Eduard Galliamov, Tatyana Garmanova, Victor Kakotkin, Ekaterina Kazachenko, Valery Kubyshkin, Alexandr Lukianov, Daniil Markaryan, Ekaterina Semina, Khasan Dzhumabaev, Sergey Gordeyev, Zaman Mamedli, Evgeniya Aleksandrova, Egine Balayan, Zinaida Galchikova, Aleksandr Gusev, Victoria E. Khoronenko, Denis Kotov, Maria Shemetova, Emin Sugaipov, Pavel Suvorin, Kirill Usachev, Vladimir Varvarin, Анна Маланова, Arkady Bedzhanyan, Mikhail Bredikhin, Eduard Charchyan, Yulia Frolova, Konstantin Petrenko, Evgenya Tyurina, Татьяна Македюнская, Alexander Abelevich, Andrey Bazaev, Alexey Yanishev, Kiselev Nikolai, Gaik Torgomyan, Vladimir Zagainov, Vitaly Dorofeev, Igor Panov, Oleg Perepelitsa, Kseniya Arutyunyan, Nikita Burlov, Mariia Karpenko, Gleb Khrykov, Alexey Shilyaev, Kirill Shostka, Evgeny Zagaynov, никюлай Манкевич, иван Сердюкюв, Anastasia Novikova, Oleg Ten, Aleksandr Zakharenko, Anton Akulaev, Dmitrii Buzanakov, Roman Chernikov, Maxim Chernykh, Timur Dzhumatov, Zhanna Glushchenko, Gleb Kim, Nikita Kubin, Ivan Labetov, Andrej Povalij, Dmitry Shkarupa, Dmitry Shmatov, Andrei Shulgin, Nepomnyaschaya Swetlana, Alexey Trofimow, Olga Volkova, Denis Zinkevich, Anna Zolotoukho, дмитрий Байбуз, Элла Григюрян, Golomidov Alexander, Aleksandr Butyrskii, Evgeniy Drozdov, Andrey Koshel, Nikolay Shefer, Petr Chavkin, Oleg Midlenko, Saydash Shamsutdinov, Evgeny Toneev, Novgorodskii Vladimir, Abakar Abdullaev, Mahir Gachabayov, Сергей Егюрюв, Юрий кудрявцев, анна кудрявцева, вячеслав Тен Oscar Rwego, Isaie Sibomana, Oda Ituze, Christophe Mpirimbanyi, Sandrine Umutesi, Fidele Byiringiro, Ainhoa Costas‐Chavarri, Muneza Eugene, Ngoga Eugene, Sofía Garcés Palacios, George Gasana, Afrika Guido, Charlène Habarugira Inyange, Lule Joseph, Claudia Kabanyana, Patrick Kwizera, Nzabamwita Liliane, Yves Nezerwa, Muyenzi Leon Ngeruka, Alexandre Nyirimodoka, Jean Paul Rugambwa, Daniel MUHIRE Runanira, Musore Sam, Jean Paul Shumbusho, Ian Shyaka, Alphonse Marie Sibomana, Belise Stella Uwurukundo, Jc Allen Ingabire, Dusabimana Anitha, Mediatrice Batangana, Achille Bizimana, Nyinawabagesera Blandine, Gisele Bunogerane Juru, Muhawenimana Claudine, Nyirasebura Dancilla, Vincent Dusingizimana, Lambert Dusingizimana Rutayisire, Muhawenimana Emmanuel, Basonga Emmy, Ntirenganya Faustin, Gasengayire Gilbert, Sylvie Inyange, Magnifique Irakoze, Amol Kulkarni, Sonia Mukase, Severien Muneza, Emmanuel Munyaneza, Emmanuel Mutabazi, Isaie Ncogoza, J M Solange Nkubito, Steven Nshuti, Edmond Ntaganda, Domitille Nyirahabakurama, Jeannette Nyirahabimana, Barangwa Theophile, Jean Christian Urimubabo, Mukanyange Violette, Rene Mukezamfura, Ronald Tubasiime, Immaculee Mutimamwiza, Irénée Niyongombwa, Japhet Ntezamizero, Mushabab Alshahrani, Nasir Magboul, Ahmed Mangahy, Abdulaziz Alghamdi, Abdulrahman Almutawa, Fahad Alqahtani, AbdulAziz Al Hindi, Abdullah Albarrak, Abdullah Almunifi, Saad M. Alqahtani, Afnan Alsultan, Ahmed Alzahrani, Nasser Alzerwi, AlaELDEIN Elnaema, Musaed Rayzah, Reda Abd ElGhany, Ahmed Al Ameer, Dauda Bawa, Mohammed Elzain, Fadi Wakill, Abdulrahman Almuawi, Omar Mohamed Alsamahy, Mir Fahiem‐ul‐Hassan, Mohammed Al Duhileb, Hussain Al Jawad, Emad Al‐Absi, Huda Alawami, Ghaleb Alazzeh, Amirah Aldhurais, Fozan Aldulaijan, Susan Alghamdi, Thabet Alghazal, Zahrah Alhajji, Abdulrahman Alkhatib, Nora Almana, Ashraf Almatar, Bikheet Almatar, Manal Alnaimi, Hadeel AlOmran, Sarah Alrubaish, Turki Alshammari, Munir Alsuwaimel, Abdullah Alwabari, Ali Alzahir, Abdulaziz Babaier, Ameera Balhareth, Sara Kanfar, Tariq Madkhali, Mumtaz Sarang, Tawfeeq Tawfeeq, Bader Alburayh, Ahmed Alkhalifah, Abddulrahman Almulhim, Malak Abutaleb, Mohammed Ageeli, Ehab Alameer, Nuraddin Alhakami, Barrag Alhazmi, Saud Alkhayrat, Saeed Almalki, Norah Durayb, Salman Ghazwani, Riyadh Hakami, Mohammed Miftah, Sameer Otayfah, AbdulRahman Sari, Momen Shawk, Abdu Ayoub, Awsam Hakami, Hesham Hamaly, Awaji Al Nami, Hussain Alqaser, Mohammed Alsadiq, Orkia Belhadri, Mohammed Fouad, Othman Iskander, Hazem Khatab, Mohammed Othman, Mohammed Rida, Samer Al Athath, Mohammed Alharthi, Ameen Alherabi, Basmah Altuwayjiri, Khalid Bakier Mohammed, Muhannad Bayazid, Walid Bukhari, Mohammed Ghunaim, Mohammad Monir Abbas, Abdullah Abdullah, Bassam Addas, Nouf Akeel, Osman Al‐Radi, Sonds Al‐Shammakh, Ibtihal Alghamdi, Amani Alhaddad, Mohammed Alharthi, Murad Aljiffry, Alia Aljifri, Adil Aljohari, Maram Alkhatieb, Soha Alomar, Samir Alsulaimani, Abdulmalik Altaf, Mohammed Alyousef, Fatima Arab, Kholoud Awaji, Saleh Baeesa, Khalid Bajunaid, Mohammed Bangash, Mohammed Basendowah, Nasir Bustangi, Sara Farsi, Deema Farsi, Nada Farsi, Ali Farsi, Mohammed Ghunaim, Alaa Habeebullah, Ahmad Khoja, Ashraf Maghrabi, Nadim Malibary, Ziad Malibary, Mohammed Nassif, Anfal Nawawi, Abdulrahman J Sabbagh, Salma Sait, Abdulaziz Saleem, Ali Samkari, Hanaa Tashkandi, Nora Trabulsi, Ahmed Alawi, Abdulaziz Alghamdi, Bassam Altalhi, Turki M Alzaidi, Heba Jaloun, Samiullah Mughal, Ahmed Organjee, Mohammed Rashwan, Abdulmajeed Abdulaziz Saeedi, Mustafa Samadony, Mohamed Shalaby, Mazen Zidan, Mohammed Algarni, Majed Almuraee, Mubarak Alshahrani, Awwadh Althobaiti, Azah Althumairi, Ali Alyami, Alwaleed Alyami, Mohammed A Alzahrani, Hadi Hakami, Ahmed Abd Elwahab, Abouelnour Abdelbaset, Nabil Abdulqawi, Abdullah Abdulrahem, Abdullah Abubakar, Mohammed Ahmed, Hessa Al Habes, Mohammed Al Jamahir, Rashed Al Qudhaya, Hajr Al Wadei, Ali Al‐Qannass, Qusay Al‐Qurashi, Mohammed Al‐Urfan, Samer Alammari, Abdulbari Alawadhi, Adel Albaiti, Ibrahim Albakry, Maher Alkahal, Samer Alkarak, Omar Alkhanbashi, Mashhour Alqannas, Abdullah Alqattan, Murtagi Alraboui, Yasir Alsagoor, Mazen Alsakkaf, Nasser Alsharif, Mohammad Alyami, Hamad Alyami, Mohammed Alzamanan, Mahdi Alzamanan, Delia Cortés‐Guiral, John Spencer Daniels, Amar Elawad, Said Elsagheer, Mohammed Fagihi, Mana Hajlan, Emad Hamdy, Mohammad Ibrahim, Safa Mahmoud, Taher Mahnashi, Atef Mansour, Haider Mohamed, Rawabi Mohammed, Mayad Moktash, Mohammad Mousa, Hussain Munyif, Hamoud Obied, Mohammed Samara, Tariq Azam Siddiqi, Youssef Taha, Abdulrahman Al Amri, Ghaleb Aboalsamh, Ohood AlAamer, Abeer Alaglan, Salman Alahmed, Khalid Alaqeely, Ali Albargawi, Abdullah A. Alghamdi, Sultan Alhabdan, Fahad Alhelal, Hani Alkattan, Mohammed Alnamshan, Mohammed Alnasser, Lolwah Alriyees, Meaad Alromaihi, Norah Alsabty, Maryam Alsafi, Jawaher Alsahabi, Nahar Alselaim, Azah Althumairi, Hassan Arishi, Manerh Bin Mosa, Khalid Bin Saad, Ahmed Binjaloud, Sarah Breakeit, Hattan Dagestani, Abdullah Eissa, Basmah Ghallab, Azza Madkhali, Arwa Mahfouz, Muath Abaalkhail, Muhammad Abukhater, Mehjabeen Adenwalla, Ghiath Al Saied, Yousof Alabdulkarim, Ahmed Alamri, Mohammed Alateeq, Majed Albarrak, Naif Aldhaam, Abdullah AlFakhri, Mohammad Alfarah, Ahmad Alghamdi, Bandar Alharthi, Mofarej Alhogbani, Aisha Mansoor Ali, Mohammed Alkahlan, Raed Almannie, Mohammed Almatrafi, Khalid Almohaimeed, Eman Almotairi, Fatema Almushawah, Hisham Almutawa, Suliman Alobaid, Loai Alqahtani, Saad Alqasem, Shahad Alqreen, Mohammed AlRayih, Mohammed Alreshidan, Khaled Alsaleh, Alanoud Alsamari, Nouf Alshammari, Abdulaziz Alshammari, Zeyad AlSolami, Fahd AlSubaie, Youssuf AlSuhaibani, Yazeed Alsuliman, Saleh Alsuwaydani, Abdulrahman Alturki, Saud Alzahrani, Gmaan Alzhrani, Mohammad Asiri, Tehmina Aziz, Mohammed Bafaquh, Maher Moazin, Marah Nadreen, Ahmed Abu‐Zaid, Mohammad Aburahmah, Lama Al Humaid, Aminah Al Nafesa, Saleh Al Nassar, Faisal Al Otaibi, Ismail A. Al‐Badawi, Mohammad Al‐Qattan, Amira Alabbasi, Hani Alahdal, Ibrahim Alahmadi, Ahmed Alahmari, Sara Alaqel, Ali Alassiri, Nof Albawardy, Khalid Aldaghiri, Homoud AlDahash, Maryam ALeissa, Saad Algarni, Ammar Alhaidari, Othman Alhammad, Naif Alhathal, Alaa Alhazmi, Norah Alhazzaa, Amal Alhefdhi, Boshra Alhelal, Meshari Alhuthayl, Abdullah Alkassim, Wafa Alkhayal, Ali Almalaq, Osama Almalik, Felwa AlMarshad, Meshal Almeshal, Razan Almesned, Hadi Almohsen, Hanan Almutairi, Alanoud Alomair, Osama Alomar, Faris Alomran, Naif H Alotaibi, Ahmed AlOtaibi, Moraya Alqahtani, Abdulmajeed AlQahtani, Saad Alqarni, Reem AlRakaf, Fay Alresaini, Omar Alrifai, Musab Alsakka, Reem Alsalamah, Yazeed Alsebayel, Muhannad Alsemari, Jaffar Alshahri, Saud Alshanafey, Khalid Alshehri, Yasir AlShehri, Saif Alsobhi, Thuraya AlSumai, Assma Altaher, Ibrahim Althubaiti, Abdullah Alzahrani, Muhammad Ansary, Ibrahim Asiri, Hizabr Elsheikh, Faisal Farrash, Zakaria Habib, Fuad Hashem, Maher Hassounah, Attiya Ijaz, Abdulaziz Jarman, Dana Kalagi, Wafa Khudier, Samer Koussayer, Nehal Mahabbat, Sabreen Maqbol, Mohamed Amir Mrad, Wessal Otaif, Eyas Othman, Rajeev Pant, Atif Rafique, Syed Raza, Bashayer Saeed, Hani Salem, Qutaiba Shah Mardan, Peter Spangenberg, Maha Tulbah, Nadia Ajomah, Abdullah Albdah, Rami Alghamdi, Abdullah Almufarrih, AlHanouf Alsaloom, Norah Alsubaie, Sharfuddin Chowdhury, Kareem ElSanhoury, Mohammed Koumu, Abu Talha Siddiqui, Amal Abdulkareem, Kaleem Ahmed, Abdulrazag Ajlan, Khalid Akkour, Sultan A Al Amri, Rehab Al Moagal, Amro Al‐Habib, Hazem Al‐Mandeel, Ghadeer Al‐Shaikh, Adel Alahaidib, Mohammed Alali, Ossama Alamri, Abdullah Alatar, Khalil Alawi, Nada Alayed, Ahmed Alburakan, Lateefa Aldakhyel, Abdulrahman Aldakkan, M Zahir Aldalati, Wassim Aldebeyan, Abdallah Alferdaus, Doaa Alfraidy, Lolowah Alghuson, Hani Alhalal, Basmah Alhassan, Fawzi Aljassir, Abdullah Aljunaydil, Abdulaziz Aljurayyan, AbdulAziz AlKanhal, Waleed H. Alkhamis, Abdulaziz Almaawi, Ahmad Almalki, Raed Almannie, Abdullah Almousa, Abdulaziz Alomar, Jalal Alowais, Awadh Alqahtani, Abdulaziz Alsaif, Nuha Alsaleh, Hisham Alsanawi, Sulaiman Alshammari, Ibrahim Alshaygy, Abdulmajeed Altoijry, Talal Altuwaijri, Ikhlass Altwejri, Nasser Alwehaibi, Reem Alyahya, Meshari Alzahrani, Abduljabbar Alzuhair, Khalid Arab, Zeneb Babay, Ali Bassi, Ahmad Bin Nasser, Areej Bokhari, Sherif Elwatidy, Hadeel Helmi, Abdullah Kattan, Saad Khashogji, Nayef Mansour Alshammari, Rafif Mattar, Hatan Mortada, Bushr Mrad, Thamer Nouh, Yasser Sabr, Mishary Shalhoub, Turki Alajmi, Deem Alakeel, Rana Alhossaini, Ali Aljuzair, Muath Alrashed, Tareq Alsabahi, Ameen Alshehri, Talal Altahan, Mohammad Altuwaijri, Muath Alwabel, Mohamed Aly, Mosa Alzahrani, Adel Assiri, Ahmed Bakhsh, Mohammed Sbaih, Jubran Al Faifi, Shehanah Al Omair, Saad Alobaysi, Saad Alobaysi, Khalid Beidas, Nawaf Modahi, Norah Musallam, Khaled El Gazzar, Khaled Ghabban, Ahmed Abdulmohsen, Hussam Adi, Amgad Afifi, Khaled AI Nwijy, Turki AI Zahrani, Jehad Akiely, Abdulaziz Al Harthi, Attiya Al Zahrani, Feras Alahmad, Yousef Alalawi, Ahmad Alayed, Abdallah Alghazo, Nawaf Alharthi, Sultan Alharthi, Mohammad Alnoaiji, Bandar Alqahtani, Abdulellah Alqarni, Noorsabah Alrubays, Mohammed Alshehri, Abdulmajeed AlShehri, Mamdouh Alzaibak, Majed Attoun Attoun, Ahmed Awaji, Yaquob Daghriri, Reda Daoud, Aymad Eltawab, Sherif Emara, Ayman Farouk, Anas Fatani, Mojahid Ghazi, Talal Khewater, Elsayed Monier, Nizar Musawa, Magdy S. El‐Bahnasawy, Mahmoud Zakaria, Saleh ALghamdi, Waleed Althobaiti, Selmy Awad, Ibrahima Konate, Abdourahmane Ndong, Jacques Tendeng, Ivan Paunovic, Nikola Slijepcevic, Nouf Alajaji, Lidija Aleksić, Goran Barisic, Aleksandar Bogdanovic, Miljan Ceranic, Ivan Dimitrijevic, Keramatollah Ebrahimi, Daniel Galun, željko GrubaČ, Nikola Grubor, Nenad Ivanović, Jelenko Jelenkovic, Stefan Kmezić, Djordje Knezevic, Zoran Krivokapic, Stojan LatinČić, Velimir Markovic, Slavko Matić, Marko Miladinov, Aleksandar Ninic, Maja Pavlov, Ivana Pavlovic, Ilija Pejovic, Dejan Radenkovic, Zeljko Radojkovic, Slobodan Rašić, Milorad Reljic, Predrag Sabljak, Aleksandar Sekulić, Vladimir šljukić, Boris Tadic, Jovica Vasljević, Dejan VeliČković, Marko Zivanovic, Pavle Gregoric, Zlatibor Loncar, Dusan Micic, Drago Jelovac, Srbislav Pajić, Milan Petrovic, Sanela Sumrak, Vladimir Bascarevic, Ivan Bogdanovic, Danica GrujiČić, Rosanda Ilic, Miloš Joković, Mihailo Milićević, Filip Milisavljević, Aleksandar Miljković, Aleksandra Paunovic, Vuk šćepanović, Aleksandar Stanimirovic, Marko Todorovic, Miljan Folic, Ana Jotic, Sanja Krejovic Trivic, Jovica Milovanovic, Aleksandar Trivic, Lazar Davidovic, Stefan Ducic, Igor Koncar, Uros Bumbasirevic, Zoran Dzamic, Boris Kajmaković, Bogomir Milojevic, Nebojša Prijović, Marko Zivkovic, Aleksandar Lazic, Nebojsa Mitrovic, Dejan Stevanovic, Marko Buta, Ana Cvetkovic, Igor Djurisic, Merima Goran, Zorka Inic, Andjela Ivezić, Nikola Jeftic, Marko Jevric, Vladimir Jokic, Milan Kocic, Zoran Kozomara, Ivan Markovic, Srdjan Nikolic, Nada Santrac, Nevena Savković, Igor Spurnic, Dobrica Stevic, Dejan Stojiljkovic, Jovana Tripkovic, Nikola Vucic, Milan Zegarac, Tatjana Dejanovic, Vladimir Djukic, Srdjan Marinković, Miljan Milanovic, Nemanja Pijanovic, Milena Radosavljevic, Predrag Savic, Dejan Stojakov, Rastko živić, Sinisa Ducic, Mikan Lazovic, Jovana BojiČić, Vladica Cuk, Jovan Juloski, Aleksandar Karamarkovic, Marko Kenic, Bojan Kovacevic, Igor Krdzic, Vladan Milutinović, Vladimir Djan, Velicko Trajkovic, Dragana Zivkovic, Aleksandar Đermanović, Danica Golijanin, Dejan Lukic, Zoran Radovanovic, Dragana Radovanovic, Sanja Zahorjanski, Adrian Chiow, Jun Xian Hing, Lip Seng Lee, Sey Kiat Terence Lim, Chi Wei Mok, James Ngu, Su‐Ming Tan, Hiang Jin Tan, Nan Zun Teo, Hana Arbab, Candy Choo, Doris Mae Dimatatac, Yee Low, Min Hoe Chew, Hui Wen Chua, Tousif Kabir, Frederick Koh, Sabrina Ngaserin, Lester Ong, Kiat Tee Benita Tan, Alvin Tan, Wei‐Wen Ang, Yew‐Lam Chong, Jeffrey J Leow, Biquan Liu, Ming Ngan Aloysius Tan, Ern Yu Tan, Kar Yong Wong, Sharon Yeo, Ladislav Czako, Branislav Gális, Arpád Panyko, Kristián šimko, Radoslav MorochoviČ, Martin Paulo, Marián Sedlák, Jan Grosek, Jurij Aleš Košir, Ales Tomazic, Leyla Al Mahdawi, Nuhi Arslani, Andrej Avsenak, Uros Bele, Andrej Bergauer, Krešimir Blažević, Dejan Bratus, Dalibor Cankoski, Andrej Cokan, Bojana Crnobrnja, Andraz Dovnik, Vojko Flis, Samo Fokter, Urska Gajsek, Minja GregoriČ, Marko Hazabent, Tomaz Jagric, Niko KavČiČ, Jure Knez, Nina Kobilica, Rok KovaČiČ, Lara Lukman, Urska Marolt, Jan Mlakar, Tamara Mohorko, Andrej MoliČnik, Ivan Perić, Stojan PotrČ, Milena Senica Verbic, Mateja Sirše, Jerneja Vidmar, Adnan Sayid Abdo, Abdifatah Dahir Ali, Sabra Aqil, Hassan Daoud, Abdilaahi Ahmed Hayir, Sadiq Ibrahim, Afnan Nuh, Kadra Omar, Ridwan Saeed, Nikita Blake, Cassandra Ferreira, Corne Nel, Colin Noel, Nikita Blake, Marcel de Kock, Cassandra Ferreira, Nathan Kaplan, Emile Kayombo, Paballo Clinton Khaeane, Asha Malan, Wikus Wessel Mulder, Corne Nel, Colin Noel, Daylen Rutledge, Cornel Steyn, Gert Steyn, Naser Almgla, Mohamed Daoub, Hammaad Gamieldien, Thomas Hilton, Nazmie Kariem, Maahir Kariem, Christo Kloppers, Maritz Laubscher, Wanga Mtimkulu, Walid Mugla, Daniel Nel, Ferhana Gool, Timothy Hardcastle, Wasim Mahomed, Nivashen Pillay, Kayla Rossini, Stephanie Van Straten, Annamart Laubscher, Adelaide Lee, Colin Noel, Mohammed Yusuf Parker, Sinead Quirke, Therese Du Preez, Adeline Jacobs, Reinier Swart, Modise Zacharia Koto, Neha Kumar, Imraan Sardiwalla, Mapuor Areu, Cristina Barrena López, Juan Campos Garcia, Paula Ferrara, Christoph José Klein Zampaña, Raquel Lopez, Hernan Sandoval, Guillermo Carreño, German Mínguez Ruiz, Antonio Rodriguez Infante, Conrado Andrés Ros, Raquel Angulo Artal, Luis Barbier, Patricia Caja Vivancos, Guillermo García‐Operé, Marta Jiménez‐Jiménez, Héctor Marín, Mikel Oñate, Luis Antonio Pascua‐Gómez, María Alexandra Pesántez Peralta, Mikel Prieto, Ibabe Villalabeitia Ateca, Igor Alberdi San Roman, Ladislao Cayetano Paniagua, Laura Gomez Fernandez, Joan Camí, Pablo Collera, Martínez David, Carlos Javier Gómez Díaz, Josep Maria Muñoz Vives, Jordi Querolt Coll, M. Dolors Rosines Cubells, Cristina Soto Montesinos, Guillermo Triana, Marina Xicola, Alfonso Alias, Albert Baduell, Mariano Balaguer‐Castro, María Pilar Camacho Carrasco, Marina Cámara Vallejo, Borja Campuzano Bitterling, Anna Carreras‐Castañer, Guillem Claret, Francisco J. Cuesta‐González, Alberto Di Somma, Joaquim Enseñat Nora, Neus Fabregas, Ada Ferrer Fuertes, Abel Ferrés, Cesar Ginesta, Jose Juan Gonzalez Sanchez, Isabel Gracia, Jhon Alexander Hoyos Castro, Montsant Jornet‐Gibert, Xavier Morales, Alejandra Mosteiro‐Cadaval, Leire Pedrosa, José Poblete Carrizo, Marina Renau‐Cerrillo, Luis Alberto Reyes Figueroa, Pedro Roldan Ramos, Jordi Rumià Arboix, Marta Sabater‐Martos, Josep M. Segur, Ramon Sieira‐Gil, Thomaz E. Topczewski, Jorge Torales, Ramon Torné, Pere Torner, Victor Turrado‐Rodriguez, Ricard Valero, Marian Vives‐Barquiel, Eulalia Ballester, Alberto Basterra Rincon, Vitiello Giulia, Belén Martin Arnau, Anna Sánchez López, Santiago Sánchez‐Cabús, Marta Gil, Silvia Martin, Monica Serrano‐Navidad, Mauricio Agüero Mariño, Núria Casanova Torrequebrada, Berta Fabregó Capdevila, Marta Jimenez Toscano, Adrián López Campillo, Gemma Mancebo, Ester Miralpeix, Blanca Montcusí, Marina Munarriz, Ariadna Salvadó, Josep M Sole‐Sedeno, Clara Téllez Marquès, Ana Centeno álvarez, Ruth Lopez Gonzalez, Luisana Riba Combatti, Andrea Sanz Llorente, Miguel Bejarano Serrano, MªPilar Canals Sin, Blanca Capdevila Vilaró, Maria Coronas Soucheiron, Mario Cuesta Argos, Irene de Haro Jorge, Yaiza Galvañ Félix, Luis García‐Aparicio, Saura García Laura, Rebeca Leal, Albert Malet Contreras, Oriol Martin Sole, Irene Martínez‐Padilla, Clara Massaguer, Ines Moraleda Gudayol, María Elena Muñoz Fernández, Pedro Palazon Bellver, Paco Parri, Cristina Pérez Costoya, Sonia Pérez‐Bertólez, Jordi Prat‐Ortells, Mireia Riba Martínez, Javier Rojas‐Ticona, Josep Rubio‐Palau, Leopoldo Tapia Moral, Xavier Tarrado, Francisco Vicario, Francesc Angles Crespo, David Bosch Garcia, Berta Escudero, Carlos Garcia Cardona, Jorge Nuñez, Sergi Bellmunt‐Montoya, Javier de la Torre, úrsula Acosta, Veronica Alonso Mendoza, Iago Alvarez Saez, Eva Andreu Riobello, Fernando Ascanio Gosling, Jose Bagan, Coro Bescós, Ruth Blanco‐Colino, Irene Brana, Bartomeu Caimari, Marta Cicuendez López Ocaña, Anna Conesa, Alba de Pablo García‐Cuenca, Natalija Dediulia, Francesc Duran‐Valles, Eloy Espin‐Basany, Martin Espinosa‐Bravo, Daniel Gil‐Sala, Jordi Giralt López de Sagredo, Maria Jose Gomez‐Jurado, Susana González‐Suárez, Ernesto Guerra‐Farfan, Juan Sebastian Guillén, María Guisasola Rabés, Viviana Andrea Hernández Angel, Maria Jurado Ruiz, Yuri Loaiza, Manuel Lopez, Susana Manrique, Maria Dolores Mateo Arzo, Joan Minguell‐Monyart, Enric Miret Alomar, Sonia Monreal Clua, Nuria Montferrer Estruch, Víctor Morales Ariza, Clara Morales Comas, Judith Murillo, Jose Maria Nieto Rodriguez, Jorge Nuñez, Elena Vilardell Ortiz, Jorge Pamias, Montserrat Pascual Pascual Arellano, Remei Perera Sarri, Aleix Pons Bartroli, Lucia Porteiro Mariño, Nil Prat, Alfredo Pueyo Ferrer, Rosa Pujol Pina, Jordi Raurich‐Leandro, Maria José Reche Padilla, Joaquín Rivero Déniz, Natalia Rodriguez, Ana Rodríguez‐Tesouro, Fabian Rojas Portilla, Manuel Saez Barba, Vanessa Sanchez Torrents, Berta Serrano, María Sol Siliato Robles, Christian Siso Raber, Iñigo Soler, Maria Carmen Suescun López, Jordi Teixidor Serra, Cristina Tello‐Díaz, M Pilar Tormos Pérez, Marta Utrilla Pérez, Ramon Vilallonga, Inmaculada Vives, Irene Vives Roselló, Vázquez García César, Mireia Gili‐Bueno, Carlos Gomez Roig, Gloria González, Aamer Malik, Maria del Mar Martí‐Ejarque, Mario Rafael Medina Hernández, Carolina Pozo, Laura Ruiz‐Villa, Nuria Sierra, Antonio Valle, Susana Velasquez, Mirentxu Arrieta, Daniel Escobar, Unai Garcia De Cortazar, Arkaitz Lara, Alicia Rojo, Kiara Tudela, Idoia Villamor, Javier Aitor Zabala Lopez‐Maturana, Begoña Estraviz, Laura Fernández Gómez Cruzado, Melania González de Miguel, Aitor Landaluce‐Olavarria, David Lecumberri, Victor Luis Gomez Corujo, Silvia Bleda, Joaquin De Haro, Mercedes Estaire Gómez, Carlos Martínez‐Pinedo, David Padilla‐Valverde, Rafael Picón Rodríguez, Francisco Javier Redondo Calvo, Daniel Sanchez‐Pelaez, Alfonso Espinosa Ruiz, José Manuel Morales‐Puebla, Eduardo Rodriguez, Ignacio Alcalá Rueda, Gonzalo Díaz Tapia, Alvaro Sanchez Barrueco, Maria Dieguez López, Isabel López García, Pilar López‐Toribio López, Marina Bosch, Carolina Curtis Martínez, Alba Fernández Candela, Sandra Lario Pérez, Cristina Lillo García, Clara López de Lerma Martínez de Carneros, Francisco López Rodríguez‐Arias, Inmaculada Oller, Saray Quinto, Luis Sánchez‐Guillén, Antonio Sanchís López, álvaro Soler‐Silva, Daniel Triguero Cánovas, Victoria Garcia Peces, Estefania Hernández‐García, Guillermo Plaza, Rafael Abad Alonso, Francisca Aranda Lozano, Marta Calvo Fernández, Eneko Del Pozo Andres, Josune Etxabe Gurrutxaga, Francisco Javier Fernández Pablos, Francisco Javier Ibáñez‐Aguirre, Begoña Ochoa Villalabeitia, Natalia Ortega, Amaia Sanz Larrainzar, Bakarne Ugarte‐Sierra, Irune Vicente Rodriguez, Virginia Jimenez, Javier Jimenez Miramón, Jose M Jover, Tamara Llamero, Jose Luis Ramos Rodriguez, Ainhoa Valle Rubio, Maria Teresa Albiol, Codina Antonio, Marta Bertrand, Clara Codony, Olga Delisau‐Puig, Ramon Farres Coll, Cristina Farrés Pla, Jorge Garcia‐Adamez, Nuria Gomez Romeu, David Julià Bergkvist, Santiago Lopez‐Ben, Eloy Maldonado‐Marcos, Marcel Pujadas, Berta Tió, Francesc Tuca, Elizabeth Bárcena, Esther Brea Gómez, Antonio Francisco Guisado Calderón, Elisabeth Fernandez Castro, Montserrat Mairal Fraile, Oscar Pastor, Lucia Diego García, Cristina García Amador, Raquel Latorre Fragua, Marta Allue, Pablo Colsa, Teresa Gimenez Maurel, Raúl Latorre Tomey, Luis Francisco Martín Anoro, Luca Ponchietti, Marta Roldón Golet, Alejandra Utrilla Fornals, Carlo Brugiotti, Carlos Jiménez Viñas, Fátima Sena‐Ruiz, Julio Plata‐Bello, Octavio Arencibia, Daniel Gonzalez Garcia‐Cano, Maria Laseca, Antonio Navarro‐Sánchez, Iván Soto‐Darias, David Ortiz López, Maria Pelloni, Aida Cristina Rahy‐Martín, Sara Berrocal, Elima Pilar Cagigal Ortega, Jorge Calvera, Iria Cervera, Patricia Díaz Peña, Silvia Verónica Domínguez Ovejas, Francisco Donis, Diego Enjuto, Patricia Fernández Bernabé, Gema Fraga, Raúl Garcés García, Antonio García, Elena Garcia De Castro, Antonio García Domínguez, Miguel Angel García García, Judit Gonzalez, Carles Heredia Llinàs, Inés Hernández, Norberto Herrera Merino, Carmen Lucero León Gámez, Ana Luis Siles, Maria Marqueta De Salas, Paula Martinez Pascual, Almudena Ortiz Simón, Laura Osuna, Marta Perez Gonzalez, Antonio Ramos Bonilla, Adolfo Ramos‐Luengo, Lorena Rodríguez Gómez, Jose Luis Uquillas, Miguel Angel Alonso Prieto, Marta Ballesteros‐Pomar, Sara Busto Suarez, Mario Castaño, Laura Castillo Pardo, Mario De Arriba Alonso, Fernando Diez Burón, Anna Farrés Rabanal, Diego Fernández‐Samos Fernández, David Garcia, Cristina García Pérez, Miguel García Sanz, Cesar Gracia, Javier Gualis, Gonzalo Gutiérrez Carrillo, Mateo Hevia, Eva Higuera Miguélez, Gregorio Laguna, Cristina Lombardia Gonzalez de Lera, Pablo López Martínez, Pasquale Maiorano, Sergio Marcos Contreras, Elio Martín Gutiérrez, Marta Molina, Irene Perez, Teresa Renedo Villar, Luis Angel Suarez Gonzalez, Eva Alvarez Torres, Luz Bueno Rey, Zulay Adriana Calderon Barajas, Ana M Castaño‐Leon, Juan Delgado Fernandez, Aida Fernández, Pablo Garcia Pimentel, Luis Jimenez‐Roldan, Meta Levstek, Carolina Lugo Duarte, Rosalia Navarro Casado, Claudia Olea Vielba, Ana Maria Parada Rodriguez, Igor Paredes, Patricia Pascual‐Cambero, Miguel Saiz Sánchez‐Buitrago, Claudia Sarrais Polo, Victor Zarza Fernandez de Alegria, Mireya Bonet, Alberto Encinas Vicente, Jose Miguel Villacampa Auba, Maria Dolores Burgueño, Hanna Perez‐Chrzanowska, Patricia Serrano Méndez, Thomas W. Jorgensen, Lucila Marquez, Mercedes Martin, Diana Crego Vita, Mónica Huecas, Felipe Velasco, Verónica Alen Villamayor, Santiago Alonso Bartolomé, Miguel Alonso Juarranz, Luis Felipe ávila‐Ramírez, Javier Buendia Pérez, Barbara Burgos‐Blasco, Enrique Camarero Rodríguez, Paula Campelos Fernández, Claudia Celotti, Marco Ciappara Paniagua, Ignacio Cristobal, Jana Dziakova, Alejandro Encinas Bascones, Elena Fernandez‐Martin, Inés Gil Prados, Jesus Gimeno Hernandez, Maria Alejandra Giraldo, Manuel Gomez Cervantes, Noemi Guemes Villahoz, Araceli Hernández Ramos, Maria Cruz Iglesias Moreno, Bibiana Lasses Martínez, Francisco Leyva Rodríguez, Leyre López Antoñanzas, Javier Martín Monterrubio, Jose Maria Martinez‐de‐la‐Casa, Lourdes Montero Cruces, Jesús Moreno Sierra, Jose Maria Muguerza, Alexia Victoria Paluso Montero, Natalia Pérez Romero, Adriana Poch, Jaime Rodríguez de Alarcón, Adriana Ruano Campos, Rebeca Ruiz Roman, Patricia Saez Carlin, Cristina Sánchez del Pueblo, Lidia Sotillo Valenzuela, Armando Galvan, Miguel ángel García Ureña, Enrique González, Manuel Medina Pedrique, Ana Maria Minaya Bravo, Alvaro Robin Valle de Lersundi, Carlos San Miguel, Juan Aragón‐Chamizo, Monica Ballon, Jose M Barrio, Jorge Caño Velasco, Beatriz Castro Catalan, Laura Cebolla Rojas, Renan Carlo Colombari, Miguel Cuende Diez, Paula Dujovne Lindenbaum, Oscar Gil‐de‐Sagredo, Silvia Kayser Mata, Pablo Lozano Lominchar, Olga Mateo‐Sierra, Yeniffer Tatiana Moreno Salazar, Angela Moreno‐Gutierrez, Melanie Morote, Uxue Murgoitio, Nikolas Palma Caucig, Lucía Polanco Pujol, Alejandro Prosperi, Begoña Quintana‐Villamandos, María Belén Ramírez Senent, Cristina Rey Valcarcel, Javier Rio, Diego Fernando Ruiz Chiriboga, Mercedes Sanz, Maria Tudela, María Adrien Lara, Raquel Alarza, Isabel Alonso Sebastian, Mariano Artés Caselles, Laura Calles‐Sastre, Marcos Casas Sánchez, Aritz Equisoain Azcona, Lucía Fuentes, Lucia Gil Cidoncha, Sofía Herrero Gámiz, Eva Iglesias Garcia, Jose Luis Lucena De La Poza, Mª Eugenia Marín Martínez, Pilar Martín Rodrigo, Joaquin Manuel Muñoz Rodríguez, Verónica Polaino, Alberto Pueyo Rabanal, Xabier Remirez Arriaga, Xiana Rial, Laura Román García de León, Miguel Sánchez Suárez, Maria Eugenia Torguet Muñoz, Borja Bazán Inostroza, Lara Blanco Terés, Alba Correa Bonito, Angela de la Hoz, Marcello Di Martino, Gustavo Eisenberg, Javier Garcia Septiem, Jose Maria Lopesino, Rocio Maqueda, Elena Martin‐Perez, Jose Luis Muñoz de Nova, Jorge Prada, Julia Revuelta Ramírez, Ana Rodríguez Sánchez, álvaro Valdés de Anca, Laura Colao García, David Díaz Pérez, Enrique Esteban Agustí, Pablo Galindo Jara, Ana Belén Gallardo, Cesar Garcia, Maria Gutierrez Samaniego, Miguel Angel Hernandez Bartolome, Lorenzo Rabadan, Barriga Sánchez Raquel, Javier Serrano González, Beatriz Diéguez, Miguel Hernández‐García, Manuel Losada, Carolina Alfonso Carrillo, María Belén Alonso Bartolomé, Estibaliz Alvarez, Mario Alvarez‐Gallego, Paula Aragón‐Ramos, Pablo Cesar Arteaga Asensio, Luis Asensio Gomez, Diego Carrion, Nuria Chavarrias, Alvaro de Arriba, Alexander Forero‐Torres, Jose Antonio Gazo Martínez Gazo, Alberto Gegúndez Simón, Juan Gómez Rivas, Natalia Gonzalez Alcolea, Carolina Gonzalez‐Gomez, Sara Gortázar de Las Casas, María Alexandra Heras Garceau, Alicia Hernández Gutierrez, Luis Lassaletta, Fuad Samir Lopez Fernández, Marta Mancheño, Blanca Mateos‐Serrano, José Manuel Morales‐Puebla, Isabel Pascual Miguelañez, Maria Fernanda Pedrero Escalas, Julio Peñarrocha, Yolanda Perez, Maria Isabel Prieto‐Nieto, Teresa Rivera Schmitz, Laura Rodrigáñez, Ines Rubio‐Perez, Isabel Sanchez Cuadrado, José Ignacio Sánchez Méndez, Jorge Fernando Tone, Carlos Toribio Vazquez, Aitor Urbieta, Santiago Valderrabano Gonzalez, Alvaro Yebes, Ignacio Zapardiel, Diego Córdova García, Manuel Diez Alonso, Francisca Garcia‐Moreno Nisa, Laura Jiménez, Inmaculada Lasa, Belen Matías‐García, Fernando Mendoza‐Moreno, Nelson Morales Palacios, Marina Pérez González, Ana Sánchez Gollarte, Alejandro Sanchez Pellejero, Cristina Vera Mansilla, Rafael Barberá, Lourdes Montes‐Jovellar, Fátima Sánchez Fernández, Alberto Cabañero Sánchez, Sara Fra Fernández, Nicolas Moreno Mata, Alfredo Abad Gurumeta, Ane Abad‐Motos, Barbara Algar‐Yañez, Gutiérrez Bérénice, Fernando Corella, Olga De La Varga‐Martínez, Paula Fernandez‐Valdes‐Bango, Sandra Maria Gadin‐Lopez, José María Martínez‐Gómiz, Elena Nieto‐Moreno, Ana Nieto‐Moreno, Montserrat Ocampos Hernandez, Maria Laura Pelegrina‐Lopez, Javier Ripollés‐Melchor, Alicia Ruiz Escobar, Elena Sáez‐Ruiz, Rosa Sanz‐Gonzalez, Beatriz Vazquez‐Rivero, Marta Alcaraz Fuentes, Antonio Lara, Belén Vielva del Campo, Fernando Alcaide Matas, Simbad Costas‐Ochoa, José María García Pérez, Elizabeth Marmol, Paula Troncoso Pereira, Maria Del Pilar Concejo Cutoli, Maria Teresa Fernández Martín, Juan Ramón Gómez López, Juan Carlos Martín del Olmo, Pablo Calvo Espino, Paloma Guillamot Ruano, Javier Páramo Zunzunegui, Antonio Albarracín Marín Blázquez, Alfonso Aliaga‐Sanchez, Marta María Arroyo Domingo, Miriam Artés Artés, Ana Isabel Avellaneda Camarena, Aida Blaya, Emny Rochell Bobadilla Romero, Milagros Carrasco Prats, Pedro Vicente Fernández‐Fernández, Antonio‐José Fernández‐López, Lorena Galindo Iñiguez, Damián García Escudero, Víctor Javier García Porcel, Vanesa Garcia Soria, Clara Giménez Francés, Antonio Javier Gomez Poveda, Rebeca Gonzalez Celdran, Francisco Miguel González Valverde, Laura Guillamon Vivancos, Elena Gurrea‐Almela, Alejandra Jara Maquilón, Jose David Jimenez Parra, Abd Al Aziz Lanagrán Torres, Raquel Lax Perez, Carmen Maria Lopez Lopez, Francisco Machado, Alfonso Marco Garrido, Florencio Manuel Marin Martinez, Jesús Aarón Martínez Alonso, Victoria Martínez Muñoz, Esther Medina, Olimpia Molina, José Manuel Muñoz Camarena, Julian Onate, Pedro Antonio Parra Baños, Emilio Peña Ros, Enrique Rubio, Miguel Ruiz‐Marín, Marina Sánchez Robles, Carlos Sanchez Rodriguez, María Valero Soriano, María Teresa Yepes García, Felipe Alconchel, Andrés Balaguer Román, Valentín Cayuela, Ana Conesa, Ana Delegido García, Pedro José Gil Vázquez, Beatriz Gómez Pérez, Paula Gómez Valles, Francisco Gómez‐Bosch, Vicente J. León‐Muñoz, Francisco Martínez, Joaquin Moya‐Angeler, Alvaro Navarro‐Barrios, Tatiana Nicolás‐López, Pablo Ramirez Romero, Emiliano Cano‐Trigueros, María Concepcion Alonso González, Leticia Gómez Viana, Ana Pastor Zapata, Elisa Contreras Saiz, Tamara Díaz Vico, Daniel Fernández Martínez, Maria Fernandez‐Hevia, Luis Joaquín García Flórez, Raquel Rodríguez‐Uria, Sandra Sanz, Lorena Solar‐Garcia, Aida Suárez Sánchez, Jaume Gelonch, Heura Llaquet Bayo, Dietmar Reinaldo, Miriam Abdulkarim Polo, Asier Aguirre, Marta Alomar Bofill, Santiago Baena, Ashish Bartakke, Carmen Cabeza Oliver, Marta Castro Suárez, José Andrés Cifuentes Rodenas, Olga Claramonte Bellmunt, Enrique Colás‐Ruiz, Ant Onia Crespí Mir, Anabel De La Llave Serralvo, Mar Escales, Isabel Rosa Fernández Burgos, Pablo Gandia Gonzalez, Juan Lliteras Jorge, María Cristina Martínez Canto, Javier Mata, Andreu Morell, Naila Pagès, Sara Pérez Palao, Cristian Plaza Valiente, Catalina Ramon Barcelo, Carmen Reyero Fernández, Albert Reyes Claret, Leticia Rodriguez Vaquero, Maialen Saez de Vicuña Salinas, Pilar Santos Cidon, Concepcion Segura, Aina Serra, Maria Serra Guivernau, Maria Soler Pedrola, Marta Tasende, Marta Torrent Lluch, María Inmaculada Valldeperas Hernández, Rosa‐Maria Yañez‐Lpez, Andrea Craus‐Miguel, Laura Fernández Vega, Natalia Pujol Cano, Juan José Segura‐Sampedro, Susana Bella Romera, Laura Colet Oliver, Ivan Dot Pascuet, Mireia Duart, Enrique Jose Ruiz Velasquez, Isabel Tello Galindo, Eduardo Arrea Salto, Jaume Cámara Cabrera, Marta Camats Terré, Juan Gabriel Castro Ríos, Marta de la Rosa‐Estadella, Anna Fernandez‐Colorado, Anna Gasulla‐Rodriguez, Antonio Gimenez‐Gaibar, Elena Gonzalez, Yeray Maldonado Sotoca, Matilde Molina‐Corbacho, Montserrat Monfort Mira, Bernardo Núñez, Josep Rodoreda, Marta Santos Espí, Arnau Verdaguer Figuerola, Elisa Angela Diego‐Alonso, Jaime López‐Sánchez, Luis Muñoz‐Bellvis, Jacobo Trebol, Borja Aguinagalde, Cristina Gabriela Alzate Arsuaga, Ainhoa Andres Imaz, Iñigo Arana, Coro Aranzabal Urrutia, Mikel Armendariz, Lorena Arrabal, Iñigo Augusto, Adolfo Beguiristain, Laura Busto, Ainhoa Cuevas, Itziar De Ariño, Alejandro Elúa, Leticia Fernandez, Arantza Fernandez‐Monge, Lander Gallego, Alba Garcia, Amaia Garcia Dominguez, Gregorio Garmendia, Carmen Grañén, Pelayo Hevia Rodríguez, Maria Iraola, José Miguel Izquierdo, Raul Jimenez, Aintzane Lizarazu, Jon Ander Lizarbe, Claudia Cristina Lopes Moreira, Iker López, Ruth Marquina González, Tania Pastor, Adrián Recio Ayesa, Ana Paula Riverola Aso, Carballo Rodríguez, Araceli Rodríguez, Maria Inmaculada Ruiz Montesinos, Patricia Torres, Jose Undabeitia, Ainara Villafruela, Jon Zabaleta, Maria Barrionuevo Ramos, David Blanco, Marta Calderón, Eva Corral Rubio, Angel Cuadrado‐García, Daniela Cubek, Ignacio Fariña, Maria Fernandez LLorente, Natalia Flores Amador, Mariana García Virosta, Luis Garcia‐Sancho Tellez, Adelina Gonzalez Martinez, Maria Jose González‐Gimeno, María Hernandez, Rubén Herreros Ruiz‐Valdepeñas, Carmen Jimenez Sanchez, Eduardo Llamazares Cobo, María López, Javier López‐Martin, Teresa Martínez Marivela, Luis Miguel Martinez Parra, Paloma Maté Mate, Paloma Medrano, Manuel Moriche Carretero, Sara Núñez O’Sullivan, Manuel Ortega Oria de Rueda, Irene Ortega Vázquez, María Begoña Pastor Nieto, Clara Pérez, Antonio Perez Ferrer, Antonio L. Picardo, Remedios Revilla Amores, Carmen Rodríguez Haro, Jose Alberto Rojo López, Fatima Sanchez Cabezudo Noguera, Cristina Sanz, Daniel Serralta de Colsa, Cristina Valor Garcia, Marta Velasco Martinez, Iván Andújar Lara, Aranzazu Calero‐Lillo, Enrique López‐Ruiz, Alberto Díaz García, Alejandro Menéndez Moreno, Luis Eduardo Pérez‐Sánchez, ángela Pascual, Miguel Adeba García, Marta Alonso Fernández, Javier Alvarez Gama, Roberto Ballestero, Loreto Berjon De La Vega, Paola Calleja Hermosa, Mariana Carrillo‐Rivas, Alfonso Casado, Andrea Cerveró, Bonifacio Cimadevilla Calvo, Patricia Corriols Noval, Paloma de la Dehesa Cueto‐Felgueroso, Miren Jasone Diez Zapirain, José Estevez Tesouro, José Antonio Fernández‐Dívar Sánchez, Lucia Garcia Alcalde, Juan García Cardo, Tito García Moreno, Daniel García‐López, Belen Garcia‐Montesinos Perea, Miren Gonzalez Benito, Rocio Gonzalez‐Aguado, Miguel ángel Gordo Vega Gordo‐Vega, Jose Gutierrez‐Banos, Jaime Jimeno Fraile, Yolanda Jubete Castañeda, Patricia Lopez Gomez, Antonia Jesús López López, Rubén Martín‐Láez, David Mato Mañas, Carmelo Morales‐Angulo, Dieter Morales‐Garcia, Marcelo Moreno Suarez, Sara Naranjo, Jose Luis Rabago, Jose Manuel Rabanal, Rocio Revuelta Zorrilla, Adriana Reyes Echeverría, Juan Carlos Rodríguez‐Sanjuán, Juan Ramón Sanz, Isabel Simal Badiola, Guillermo Tejón, Enrique Toledo Martínez, Raquel Varea Malo, Miguel Angel Freiria Eiras, Begoña Fadrique, Tania Funes, Elena González Revilla, Isaias Alarcón, Daniel Aparicio Sánchez, Pablo Beltran Miranda, Carmen Cepeda‐Franco, María Josefa Cuevas López, Sandra Dios‐Barbeito, Virginia María Durán Muñoz‐Cruzado, Carlos Gonzalez De Pedro, Rosa M Jimenez‐Rodriguez, Francisco Manresa‐Manresa, Gabriel Marin, Cristobalina Martin‐Garcia, Sara Martínez‐Núñez, Juan Manuel Martos Martinez, Lucas Mengíbar, Felipe Pareja Ciuro, Eduardo Perea del Pozo, Veronica Pino Diaz, José Pintor‐Tortolero, Claudia Quintero‐Pérez, Irene Ramallo‐Solís, Maria Luisa Reyes Diaz, Mercedes Rubio Manzanares Dorado, Alejandro Sanchez Arteaga, Ana Senent‐Boza, Luis Tallon‐Aguilar, Jose Tinoco‐Gonzalez, Luis‐Cristobal Capitan‐Morales, Juan Cintas Catena, Juan‐Carlos Gomez‐Rosado, Javier Valdes‐Hernandez, María Del Campo Lavilla, Alina López De Fernández, Jose Ramon Oliver Guillen, Miriam Abellan Lucas, Mar Achalandabaso Boira, Alba Diaz Padillo, Maria Gómez Romero, Linda Grace Puerto Tamayo, El Mostafa El Yaqine Er Raoudi, Melody García Domínguez, María ángeles Gascón Domínguez, Marta González Pérez, Alba Hernáez Arzoz, Joana San Anton, Cristian Ruminot, Hander Guillermo Acosta Diaz, Daniella Laguado, Kristina Aghababyan, Jr Alba, Enrique Artigues, Jose Bagan, Rocío Belda, Miguel Beltran, Jose Ignacio Blanes, Elena Campos Carot, Miriam Cantos, Laura Costa, Carolina Ferrer Gomez, José María Gallego Sánchez, Oscar Gil‐Albarova, Juan Gilabert‐Estellés, Emilio Lopez Alcina, Isabel López Sánchez, Severiano Marin Bertolin, Carolina Martinez‐Perez, Antonio Melero Abellán, Claudia Mulas Fernández, Judith Murillo, Beatriz Novoa, Ruth Nuñez, Pablo Renovell Ferrer, Guijarro‐Jorge Ricardo, Carolina Soledad Romero Garcia, Miguel Sanfeliu Giner, Silvia M. West, Enrique Zapater, Julio Domenech, Ignacio Miranda, Ana Monís, Alejandro Roselló Añón, María José Sangüesa, Jorge Sanz Romera, Marcos Adrianzén, Rafael Badenes, Maria Barrios Carvajal, Mireia Bauzá, Ana Benítez Riesco, Jose Antonio Carbonell Lopez, María‐Carmen Fernández‐Moreno, Ricardo Gadea Mateo, Francisco Garcia, Maris Luisa García‐Pérez, Luisa Paola Garzon, Ana Izquierdo, Maria Lapeña Rodríguez, Carlos León‐Espinoza, Fernando Lopez, Rosa Martí Fernández, Sara Martínez Castro, Berta Monleón López, Isabel Mora Oliver, David Moro‐Valdezate, Ernesto Muñoz Sornosa, Sara Palomares Casasús, Leticia Pérez Santiago, Mara Albert Fort, Reyes Balanzá, Lorena Bermell Marco, Juan Carlos Bernal‐Sprekelsen, Carla Carratalá Pérez, Claudia Pilar Clemente Tomas, Marta Córcoles, Jose ángel Diez Ares, Carlos Domingo Del Pozo, Nuria Estellés Vidagany, Gonzalo Garrigos, Mar Gimeno Gimeno Vicente, Blanca Alastrue Giner, Segundo Gomez‐Abril, Paloma Gonzalez, Paula Gonzálvez Guardiola, Guillermo Sebastian Martínez Fernández, Charo Martínez García, Lucía Martínez‐Costa, Luz María Moratalla Charcos, Sergio Navarro Martínez, Carmen Payá‐Llorente, álvaro Pérez Rubio, Nuria Peris, Eduardo Picazo Pineda, Jordi Planelles Gómez, Francisco Ripoll Vidal, Andrea Roca, Enrique Salmerón‐González, Raquel Segura Roselló, Ramon Trullenque Juan, Cristina Verdejo Gimeno, Jose Miguel Zaragozá García, Gema Zomeño Bravo, Marcos Bruna Esteban, Hanna Cholewa, Milton de Jesus, Matteo Frasson, Luis Oliver García, Jorge Sancho‐Muriel, Héctor J Aguado, Antonio Jose Alonso Villalba, Francisco Ardura, Carmen Elena Badillo Bercebal, María Bedate Núnez, Alvarez‐ramos Begoña Aranzazu, Juan Beltrán de Heredia, Hugo Bermejo, Juan Berrocal Cuadrado, Maria Bragado González, Juan Bustamante‐Munguira, Raul Calvo Gonzalez, Yolanda Carrascal, Ana Belén Casas Marcos, María Carmen Cervera, Sergio Chávez Valladares, ángel Cilleruelo Ramos, Susana Cortes, Jesús Crespo‐Sanjuán, María Cuaresma, Luis Antonio Cuellar Martin, Beatriz De Andrés‐Asenjo, Carlos Ferreras García, Alvaro Fuentes‐Martín, Abel Ganso, Elena García García, Luis Garcia‐Florez, Virginia Garcia‐Virto, Juan Gatón, Julio Alberto Gobernado Tejedor, Tania Gómez Sanz, Jose Ignacio González Martín, María Begoña Gregorio Crespo, Ana Herranz Arriero, Jose Herreros, Marta Ibáñez Nieto, César Infante, Carlos Jezieniecki, Moises Juarez, Gregorio de Jesus Labrador Hernandez, Ana Patricia Legido Morán, Ricardo León Fernández, Alejandro Leon‐Andrino, Almudena Llorente, Maria Lopez Pais, Mauricio Loucel Bellino, Sergio Martin, Miguel Angel Martin‐Ferrero, Gonzalo Martinez Municio, Elvira Mateos Alvarez, José María Matilla, Luis María Merino Peñacoba, Mario Montes‐Manrique, Fernando Natal álvarez, David C Noriega, Henar Nuñez Del Barrio, Francisco Javier Ortiz de Solórzano Aurusa, Lucía Pañeda, Victoria Pascual Escudero, Miguel Pascual Samaniego, Adela Pereda, Marta Pérez Febles, Laura Pesquera, Maria Plata, Emma Puertas Ruiz, Mario Rodriguez‐Lopez, Alejandro Romero de Diego, Ana Ruano, María Ruiz Soriano, Cristina Sánchez Torralvo, Silvia Santiago Maniega, Bárbara Segura‐Méndez, Clarisa Simon Perez, José Soro‐García, Paula Suárez Mansilla Suárez‐Mansilla, Jeancarlos Trujillo Díaz, Mª Esther Valsero Herguedas, Andrea Vazquez Fernandez, Eduardo Velasco García, Ana Zabalza, álvaro Zamora Horcajada, Fernando Acebes García, Enrique Asensio Díaz, Martín Bailón, Alejandro David Bueno Cañones, Alvaro Centeno Velasco, Ekta Choolani Bhojwani, Fernando Labarga Rodríguez, Sara Mambrilla, Pablo Marcos‐Santos, Toledano Miguel, David Pacheco Sánchez, Baltasar Pérez‐Saborido, Pilar Pinto, Katherine Plua, Javier Sanchez Gonzalez, Vicente Simo, Francisco J Tejero‐Pintor, Rosalía Velasco López, Sandra Veleda Belanche, Fabiola Eugenia Michel Campos, Iciar Muñoz Lindez, Marc Vallve‐Bernal, Maria Balluerca, Maite Camuera, David Garcia, Pablo Helguera, Imanol Herrero, Patricia Paunero Vazquez, María Sánchez‐Rubio, Isami Soeda, Alba Vazquez Melero, María Dolores Arribas Del Amo, Vicente Manuel Borrego Estella, Estefania Casas, Néstor Castán Villanueva, Daniel Delfau Lafuente, Elena Delgado Blanco, María Domingo, Ismael Gil Romea, Azucena Gonzalo, Emilio Lagunas Lostao, Noelia Lete Aguirre, Yaiza Martinez Lahoz, Jesús Víctor Pérez‐Tierra Ruiz, Maria del Mar Soriano, Isabel Valero Lázaro, Esmeralda Carnicer Escusol, Guillermo Pola Bandres, Laura Sánchez Blasco, Juan Luis Blas Laina, Beatriz Cros, Jorge Escartin, Patricio Andrés Freile Pazmiño, David Garcia‐Aguilera, Ana Mejía Casado, Ana Nogués, Issa Talal El‐Abur, Carlos Yánez, Victoria Duque Mallén, Isabel Gascon Ferrer, Carlos Gracia‐Roche, Ursula María Jariod Ferrer, María Victoria Simón Sanz, Tomas Uson, Waseem Ahamed, Sumudu Jayasinghe, Eranda Karunadasa, Prathibha Nelihela, Selvaratnam Srishankar, Sujeewa Priyantha Bandara Thalgaspitiya, Dickson Wickramarathna, Nagenthiram Harivallavan, Umesh Jayarajah, Pirashanthan Nagarasa, Eranga Perera, Kanapathipillai Piratheep, Vithranage Srimantha Dewsiri Rodrigo, Charitha Sooriyabandara, Meera Thayalan, Rajendram Thayaparren, Arulprashanth Arulanantham, Oshan Basnayake, Kanishka De Silva, Gagana Ganga, Anuja Nadeeshan Kumarasinghe, Mahanada Udukala, Koculen Vimaleswaran, Prashanthan Yogarajah, Ravindri Jayasinghe, Probhodana Ranaweera, Mohamed Rishard, Dharmabandhu N Samarasekera, Sanjeewa Seneviratne, Sivasuriya Sivaganesh, Dakshitha Wickramasinghe, Chamal Fernando, Nishantha Mendis, Prabuth Dulanjan Weeraddana, Balasingam Balagobi, Balasupramaniam Sathesan, Thanusan Vimalakanthan, Lanka Dasanayake, Malitha Patabendige, Dj Wickramasooriya, Kugarajh Ganeshapillai, Shanthamoorthy Gishanthan, Sathyamoorthy Prasanna, Malitha Patabendige, Pramodh Chandrasinghe, Dileepa Ediriweera, Sumudu Kumarage, Asanka Jayawardane, Athula Kaluarachchi, Malitha Patabendige, Pituwala Liyanage Adithya Sirisena, Abubakr Abubakr, Hozifa Mohamed, Ismail Mustafa, Mogahid Sharfeldein, Samir Ismail, Reem Musa, Monira Sarih, Mussab Abaker, Eman Adam Abdalla, Mohamad Abdelbagi, Alsafe Adlan, Khalid Ahmed, Ahmed Alnaeem, Safa Atyah, Yasir Awad, Rsheeda Dawelbait, Mohamed EL Hag, Ibrahim Elbashir, Sami Eldirdiri, Samwal Eldirdiri, Mohammed Eltahier Abdalla Omer, Lana Abdalgadir Ahmed Mohamed, Hiba Hassan Rehmtallah Ahmed, Mohammed Balla Yousif Balla, Abdulmoiz Aljafari, Abdulmalek Aljafari, Elsagad Mohamed, Abdulkader Mohammad, Abdulkader Shaar, Mohamed Nafea Shaar, Maab Hussein Yousif Elhafiz, Mohammed Husien Yosif Elhafiz, Abubakr Ali Mohammed Alhassan Humidan, Almigdad Ali, Hisham Eljack, Mohamedyasin Elrashid, Manhal Ahmed, Albrra Alhag, Douaa Ali, Mohammed Ahmed Babikir, Imad Bakheit, Hytham K. S. Hamid, Sami Mohamed, Walid Shaban, Nidhal Siddig, Mohamed Suliman, Rihab Yasir Abdelmagid, Abubaker Yassin, Omer Abbas, ElTahir Abdelrahim, Muhammed Ahmed, Mohamed Elobaid, Adil Hashim, Ali Mohamed, Omer Mohamed, Khalid Osman, Abdallah Yousif, Safaa Abdulaal, Marwa Elsadig, Mohammed Hussain, Malaz Amir Ataalmanan, Doha Amir AtaAlmanan, Tebyan Amir AtaAlmanan, Alaa Elgaili, Maria Mohamed, Usra Omara, Alkhansa Osman, Amna Osman, Safaa Saad, Mohammed Elmujtba Adam Essa Adam, Abdelkareem Ahmed, Ali Adil Ali Karar, Aya ALhassan, Mohanad Alsidig, Mohamed Eljack, Mohammed Ibrahim Mohammed Ali, Awad Kamal Awad Osman, Liena Mohamed, Samah Ibrahim Omer Mohamed Osman Mohamed Osman, Altayeb Mohammed, Ayman Mohammed, Khadija Muhmmed, Eltahir Musa, Egbal Sahal Abdelmajed, Khalid Z.M.Y Hamid, Omnia M. M Yousif, Mahmoud Saleh, Osman Bashir, Yassein Elhussein, Omar Madani, Eva Angenete, Jennifer Park, Lars Gillberg, Monir Jawad, Linas Pieteris, Yousif Saeed, Sergej Safonov, Martin Spångfors, Henrik Andersson, Hans Bahlmann, Karin Björnström Karlsson, Michelle Chew, Lara Daham, Lina De Geer, Helen Didriksson, Gunilla Gagnö, Carina Jonsson, Anna Oscarsson, Pamela Buchwald, Mathilde Delorme, Marie‐Louise Lydrup, Shahin Mohseni, Arvid Pourlotfi, Souheil Reda, Kajsa Anderin, Nina Blomme, Josefin Segelman, Malin Simlund, Anders Thorell, Fredrik Wogensen, Daniel Danielsson, Peter Elbe, Rebecka Hultgren, Boel Hynning, Fredrik Klevebro, Karin Lind, Ebba K Lindqvist, Carl Montan, Ernesto Sparrelid, Clara Svenberg Lind, Jeremy Wales, Andreas älgå, Martin Nordberg, Emil Pieniowski, Ioannis Gkekas, Niklas Löfgren, Karolina Niska, Martin Rutegård, Malin Sund, Robert Zürner, Silvio Däster, Otto Kollmar, Savas Soysal, Stephanie Taha‐Mehlitz, Athanasios Tampakis, Dominik Valentin Flury, Marie Heyne‐Pietschmann, Gregor Jan Kocher, Ana M Calinescu, Barbara Wildhaber, Markus Gass, Jürg Metzger, Andreas Scheiwiller, Lukas Briner, Oliver Dwidar, Eleftherios Gialamas, Steven Grandjean, Pietro Ricciardi, Marc‐Olivier Sauvain, Alexandra Calmels, Christodoulou Michel, Morena Antonilli, Dimitrios Christoforidis, Alessandra Cristaudi, Jacopo Galafassi, Maria Luisa Gasparri, Francesco Mongelli, Martino Munini, Andrea Papadia, Sotirios Georgios Popeskou, Michel Adamina, Alex Alfieri, Thomas Bächler, Matteo Giardini, Christian Gingert, Laura Guglielmetti, Inna Meyer, Tobias Müller, Daniel Schöni, Folco Solimene, Frank Weidner, Marco Bueter, Daniel Gero, Karoline Horisberger, Amelie Müller, Carmen Portenkirchner, Andreas Thalheimer, Jeannette Widmer, Ahmad Hmaideh, Alaa Kour, Saleem Swaes, Omar Alannaz, Ramez Aljasem, Ezeddin Dabbagh, M. Wafa Hamoud Alhussein, Amr Hamza, Mazen Mohammad, Abd El‐Fattah Mouhandes, Mohamad Shareeda, Mohammad Nour Shashaa, Mousa Sifat, Sheraa Tahhan, Rahaf Toutounji, Mohamed Abdulrahman, Albaraa Abdulsalam, Ziad Al Jarad, Hala Alayyoubi, Dina Alfarra, Ahmad Alhamid, Mahmoud Alnasser, Mulham Gharib, Ruwaid Gharib, Obaida Kabel, Osama Kherallah, Abdulqader Klaho, Mohamed Shukri Najjar, Ahmad Razouk, Joud Shiekhoni, Wael Alkhaleel, Amira Almosa, Mohammad Aloulou, Maen Alzaeem, Khaled Arnaout, Muhamad Zakaria Brimo Alsaman, Mohamad Estanbouli, Hanadi Hawa, Ahmad Nbrass Kabawi, Ghina Maarawi, Yehia Mashhadany, Sana Shaikh Torab, Waleed Abd, Tayma Abd Alghafour, Hani Abdalnour, Nazeeh Al Aktaa, Marwan Al Aliwy, M Maher Al Arje, Tasneem Al Najjar, Ahmad Al‐Mouakeh, Ahmad Aldakhil, Aos Alhamid, Hasan Alhasan, Mohamad Shadi Alkarrash, Jumaa Alkhamis, Sara Alshekh, Ahmad Yamen Arnaout, Oula Azizeh, Mohammed Dalaleh, Aghyad Danial, Zain Douba, Raghad Ghadri, Mohammad Maen Ghannam, Anas Ghawi, Ahmad Ghazal, Luma Haj Kassem, M. Yasser Halwani, Shahed Hamadieh, Mohammad Hanino, Ala Aldeen Hasan, Haya Jawish, Nasr Ullah Kabbani, Saadullah Kabbany, Mohamad Kadi, Lama Kadoura, Ahmad Amir Kayali, Ammar Kayyali, Aya Kelzia, Amana Kezze, Abdullah Khalaf, Maymona Khayata, Mohammad Nour Kitaz, Mohammad Kour, Raghad Makki, Ruqaya Masri, Joseph Masri, Noor Masri, Muhammad Mazketly, Shiar Mustafa, Ezzeldin Nashed, Yaman Qoudra Danial, Hasan Raslan, Rima Salem, Mohammed Asaad Salem, Thabet Sayed Bakir, Manaf Sharabaji, Majd Sheikh Alganameh, Duaa Sheikh Kadro, Joudy Shurbatji, Mohammad Noor Sultan, Dana Sultan, Hala Swied, Bakri Tarras Jarkas, Tayf Toutounji, Fadi Ward, Rama Zazo, Aya Zazo, Zainab Zeino, Mohamed Alaktaa, Wael Alsado, Hala Fares, Nouran Hawa, Hamza Ibrahim, Abdulmonem Kawas, Mourad Niazi, Bayan Toutounji, Mohammad Abdow, Mohamad Ahmad, Tareq Ahmad, Rawan Al Azhar, Hiba Al Hage Diab, Rahaf Al Mulke, Mohammad Al‐Jadaan, Ahmad Alhussein, Zahra Alnajem, Omar Alneser, Bayan Alsaid, Elias Azar, Lubna Bakr, Ammar Hamza, Yara Houdifa, Sami Jomaa, Samer Kefo, Hiba Mardini, Alaa Mohammad, Fadi Rayya, Fatima Razzouk, Mohannad Saleh, Anwar Shamandi, Ammar Youzkatli Alkhatib, Omar Abdul Salam, Dany Ajami, Yusra Al‐Sabbagh, Mohammad Alhamid, Ahmad Alkhaledi, Danny Hadidi, Massa Jabra, Mamdouh Kallas, Antoine Laktine, Joelle Naem, Naya Naoum, Ghena Mohammed Fawaz Ashour, Ahmad Alhouri, Deema Fallouh, Ahmad N Mohammad, Ahmad Rmman, Ali Slitin, Christine Tanos, Ibrahim Wakim, Hanaa Zahrawi, Majd Abouassi, Layth Abouassi, Ayham Aboutrab, Basel Ahmad, Ahmad Mustafa Ahmad, Aya Ahmad, Afaf Ahmad, Mohamad Alabras, Mohammad Albasheer, Ola Alkasser, Mhd Khaled Alkasser, Ali Alkhdor, Marah Azkoul, Mohammad Karam Chaaban, Haya Deeb, Basel Jazieh, Basel Kouz, Youssef Kozah, Raied Mohamad, Ammar Mohammad, Antoine Naem, Mhd Zied Nasri, Mohammed Mouaz Shebani, Mohammed Shwin, Alaa Sommaq, Amal Youssef, Tala Al Asadi, Suhad Alkhateb, Rasha Khalil Alsayyad, Roula Altom, Mhd Amin Alzabibi, Batoul Bakkar, Hlma Ismail, Salma Khadem Alsrouji, Sara Melhem, Mohamad Sankari, Mosa Shibani, Noura Shujaa, Ibrahim Adham, Salma Al‐Houssami, Maram Balouli, Ghassan Jisry, Yazan Rahmeh, Simon Yanni, Sally Yousef, Aline Ahmad, Marah Ali, Abdulrahman Almjersah, Aya Almoustafa, Hiba Dahhan, Bashar Haj Hassan, Alaa Hamdan, Ali Hammed, Salah Hammed, Rawan Harfoush, Rama Hasan, Natalie Jarkas, Moufid Mahfoud, Ahmed Moussa, Alaa Sulaiman, Siba Suliman, Yu‐Ning Hu, Jun‐Neng Roan, Meng‐Ta Tsai, Yi‐Chen Wang, Varut Lohsiriwat, Thammawat Parakonthun, Nicha Srisuworanan, Voraboot Taweerutchana, Somcharoen Saeteng, Sophon Siwachat, Apichat Tantraworasin, Shane Charles, Danielle Dimsoy, Shane Khan, Akash Ramsaroop, Shivanand Ramsubhagh, Karim Ayed, Oussama Baraket, Mohamed Hedi Ghalloussi, Abbassi Imed, Nahla Kechiche, Waad Farhat, Ammar Houssem, Erdal Birol Bostanci, Volkan Oter, Erol Pişkin, Cihangir Akyol, Sibel Demirel, Mehmet Ali Koç, Can Konca, özge Yanık, Basak Bolukbasi, Huseyin Gobut, Emre Gülçek, Kerim Karabulut, Ramazan Kozan, Sezai Leventoğlu, Farshad Noori, Elif özeller, Can şahin, Ali Yalcinkaya, Mesut Yavaş, Aydın Yavuz, Utku Akgor, Omer Cennet, Hilmi Anil Dincer, Murat Gultekin, Nazlı Orhan, Mustafa Oruç, Serdar Culcu, Lütfi Doğan, Cemil Yüksel, Alp Yildiz, Aybala Yildiz, Perihan Ekmekçi, Baturay Kansu Kazbek, ülkü Ceren Köksoy, Deniz Serim Korkmaz, Zeynep şahan çetinkaya, Elif Aybeniz Yildirim, Hakan Yilmaz, Metin Yığman, Nasuh Utku Dogan, Selen Doğan, Mehmet Sakinci, Ozgen Isik, Murat şen, Tuncay Yılmazlar, Utku özgen, Ugur Sungurtekin, Arda Isik, Yener Aydin, Ilker Ince, Ali Bilal Ulas, Hüseyin Cahit Yalçın, Mehmet Kaplan, Tuğba Kaplan, Elif Tugce Kaplan, Afag Aghayeva, Bilgi Baca, Inci Sahin, Zumrud Aliyeva, Akif Enes Arikan, Erman Aytac, Onur Dülgeroğlu, Volkan Ozben, Cihan Uras, Emre Sivrikoz, Akif Enes Arikan, Ismail Ahmet Bilgin, Bahadır Bozkırlı, Güralp Onur Ceyhan, Ismail Hamzaoglu, Halil Kara, Tayfun Karahasanoğlu, Olus Api, Mehmet Ceyhan, Aylin Pelin Cil, Telce Aysen Gurbuz, Cumhur B Lent Urman, Ahmet Akbas, Yunus Emre Aktimur, Yuksel Altinel, Fikret Calikoglu, Serdar Demirgan, Ahmet Guray Durmaz, Gulcin Ercan, Candas Ercetin, Hasim Furkan Gullu, Nadir Adnan Hacım, Serhat Meriç, Kamil özdoğan, Merve Tokocin, Mehmet Alim Turgut, Talar Vartanoglu, Hakan Yigitbas, Hüsnü Aydın, Ece Bahçeci, Mestan Bilmez, Elif Binboğa, Sinan Binboga, Gokhan Demirayak, Bengi Demirayak, Keziban Doğan, Ahmet Cem Dural, Murat Ekin, Sina Ferahman, Fatih Guven, Mehmet Karabulut, Sema Karakaş, Selçuk Köse, Ayşe Büşra önder, Ismail Umut Onur, Nuri Alper Sahbaz, Levent Yaşar, Ulviye Yigit, Güneş özlem Yıldız, ümmühan Zeynep Bilgili, Bayram Doğan, Cevper Ersoz, Abdullah Ilktac, Senad Kalkan, Eyyüb Selim ünlü, Halil Alis, Süleyman Büyükaşık, Burak Kankaya, Oguzkagan Batikan, Ensar çakır, Musa Murat Caliskan, Mahmut Emin çiçek, Anil Demir, Selim Doğan, Emre Erdoğan, Mert Güler, Muhammed Gürlük, Ufuk Oguz Idiz, Rozan Kaya, Cebrail Oğuz, ömer Akay ömer, Serkan Süleyman özpak, Hüsnü şevik, Mert Mahsuni Sevinc, Cihad Tatar, Oğuzhan Tekin, Furkan Türkoğlu, İshak Yıldız, Gokhan Atis, Mehmet çağlar çakıcı, Ayberk İplikçi, Ozgur Kazan, Asif Yildirim, Mustafa Yücel Boz, Rahim Horuz, Mustafa Soytas, Müserref Beril Dincer, Ali Fuat Kaan Gok, Irem Karatas, Metin Keskin, Kemalettin Koltka, Mukadder Orhan Sungur, Ilker Ozgur, Tuba Banaz, Tugan Bese, Sukru Cebi, Fuat Demirkıran, Ergin Erginöz, Sefa Ergün, Betul Ibis, M. Bugrahan Karaca, Mehmet Faik OZcelık, Ahmet Necati Sanli, Akif Turna, Server Sezgin Uludağ, Mehmet Velidedeoglu, Beyza Irem Yabaci, Abdullah Kağan Zengin, Cem Emir Guldogan, Emre Gundogdu, Mehmet Mahir Ozmen, Mehmet Abdussamet Bozkurt, Yasin Kara, Ali Kocataş, Adem özcan, Oktay Akça, Elif Akova Deniz, Berk Cimenoglu, Recep Demirhan, Elif Cansu Gundogdu, Alper Kafkasli, Ahmet Kale, Kemal Saracoglu, Ramazan Sarı, Muhammed Fatih Simsekoglu, İbrahim Fethi Azamat, Emre Balik, Dursun Buğra, Burak Giray, Cemil Burak Kulle, Cagatay Taskiran, Dogan Vatansever, Aykhan Abbasov, Hakan Yanar, Ahmet Akmercan, Gül çakmak, Tuncay Kötan, Tunc Lacin, Ayten Saracoglu, M. Umit Ugurlu, Tumay Umuroglu, Tevfik Kıvılcım Uprak, Esra Yamansavci şirzai, Bedrettin Yıldızeli, Elif Baran, Emre Bozkurt, Sinan ömeroğlu, Mert Tanal, Aydın Eray Tufan, Mehmet Uludag, Gürkan Yetkin, Murat Kalın, Omer Faruk Ozkan, Hanife şeyda ülgür, Tayfun Bisgin, Muhammet Berkay Sakaoglu, Selman Sökmen, Deniz Caglar, Candan Cicek, Demre Delipinar, Emre Divarci, Egemen Ozdemir, Hilmican Ulman, Seda Dilek Yetut, Feyme Zafer, Ismail Sert, Burçin Abud, Murat Akalin, Cengiz Aydin, Bulent Calik, Emine Burcu Cigsar, Semra Demirli Atıcı, Ayberk Dursun, Mustafa Emiroglu, Hüseyin Erdinç, Tayfun Kaya, Gizem Kilinc, Yasemin Kirmizi, Merve Dilara öney, Mustafa Onur Oztan, Semra Salimoğlu, Cem Tugmen, İbrahim Uyar, Sertaç Ata Güler, Alican Güreşin, Ozan Can Tatar, Nihat Zafer Utkan, Kemal Arslan, Mustafa Bilal Hamarat, Ismail Hasirci, Mehmet Serkan Ozkent, Mehmet Eşref Ulutaş, Burak Yilmaz, Mertcan Akçay, Yesim Akdeniz, Emrah Akin, Fatih Altintoprak, Enes Baş, Zulfu Bayhan, Guner Cakmak, Recayi çapoğlu, Fehmi çelebi, Hakan Demir, Enis Dikicier, Ugur Can Dulger, Necattin Firat, Emre Gönüllü, Ahmet Tarik Harmantepe, Muhammet Burak Kamburoğlu, Belma Kocer, Ibrahim Furkan Küçük, Baris Mantoglu, Ali Muhtaroğlu, Kayhan Ozdemir, Merve Yigit, Zehra Alan Köylü, Engin Aybar, Mert Candan, Ahmet Burak Ciftci, Elif çolak, Huseyin Eraslan, Gultekin Ozan Kucuk, Süleyman Polat, Ahmet Can Sarı, Mustafa Safa Uyanik, Kürşat Yemez, çağrı Büyükkasap, Kayahan Eyuboglu, Ali Guner, Murat Emre Reis, Nurullah Damburacı, Omer Karahan, Barış Sevinç, Fatma Ayca Gultekin, Okedi Francis Xaviour, Odongo Samuel, Mohammed Hirsi, Gaston Turinawe, Isakwa Ibrahim, Isabirye Isa, Isaac Mubezi, Daniel Asiimwe, Mumbere Bienfait, Isaac Edyedu, Franck K. Sikakulya, Andrew Kakeeto, Esther Namutosi, Mutekanga Nicholus, Ssekitooleko Badru, Treasure Ibingira, Ehanga Idi Marcel, Racheal Kirabo, Hervé Monka Lekuya, Ronald Mbiine, Cephas Nakanwagi, Christine Namugenyi, Joseph Ntege, Arnold Ntege, Henry Othieno Misanga, Nantambi Rose, John Baptist Ssenyondwa, Nimanya Stella Alice, Lois Asiimwe, Clara Atieno Odhiambo, Francis Basimbe, Brian Bbosa, Walter Dreak Erabu, Bili Hussein, Joel Kiryabwire, Castro Kisuule, Ronald Kiweewa, Moses Magezi, Lynette Mpagi Katassi, Dionizi Muganga, Didace Mugisa, Othiniel Musana, Fredderick Mutyaba, Twaha Muwanga, Peter Muwanguzi, Assumpta Nabawanuka, Teddy Nakirijja, Goretti Nassali, Rosemary Nassanga, Moses Nassimu, Peter Ssekweyama, Peter Ssenyonjo, Kagga Ssenyonjo, Eden Micheal Ssettabi, Linda Turyabahika, Justine Walusimbi, Daniel Zaake, Herman Lule, Benson Oguttu, Paul Matovu, Denis Oluka, Mujuni Samson, Otolia Isaac, Okello Simon Peter, Vladyslav Hordoskyi, Andrii Kurmanskyi, Sofiia Zhurba, Maksym Boruta, Andrey Kebkalo, Volodimir Tyselskiy, Andriy Beznosenko, Olha Bubliieva, Viktor Cherniienko, Slava Kopetskyi, Yevhenii Kostiuchenko, Bogdan Maksymenko, Alina Minich, Sikachov Sergei, Ihor Yovenko, Khaled ElSisy, Ahmed Ibrahim, Mohamed Mashhour, Amin El Helw, Ravi Trehan, Sattar Alshryda, Vikram Somashekhar Basappanavar, Hisham M. Elsayed, Rajnish Garg, Suresh Kailasam Sivamurthy, Sabina Khan, Safeena Kherani, Rubina Lone, Ibrar Majid, Awadelkarim Mohamed, Abid Qazi, Mohamed Serour, Mohamed Mashhour, Nayzak Raoof, Gavin Spence, Ferial Mohamed Ali Abbas, Maryam Babar, Sheela Anne George Varayannoor, Diary Mohammed, Muna AbdulRazzaq Tahlak, Khalid Alawadi, Ehab Aldlyami, Amar Alomar, Taiceer Abdulwahab, Murad Abdunabi, Mohamed Mashhour, Rakesh Kundra, Mohamed Mashhour, Amr El Yamany, Mohamed Mashhour, Hossam Al Mahdy, Antony Louis Rex Michael, Maitha Alqemzi, Hayder Alsaadi, Bilal Elyafawi, Hamda Khansaheb, Omar Abdulateef, Hayder Al‐Masari, Amna Salam Al‐Wandi, Sameh S. Alsafty, Shaimaa Ibrahim, Kareem S. Khalil, Nour Abdel‐Fattah, Rachel Dbeis, Ruari Jardine, Priyanka Kunte, Gianluca Maresca, Stuart McIntosh, Lucy McLeod, Ahmed Nassar, Ashrafun Nessa, Laura Osborne, Peter Osei‐Bonsu, William Paine, Jelizaveta Pereca, Shafaque Shaikh, Rachel Vaughan, Kanastana Yasotharan, Jenny Ferry, Megan Kershaw, Angharad King, Joanna Krawczyk, Cheri Price, Anushka Sieunarine, Andrew Temperton, Vimla Victor, Sophie Walker, Matthew Williams, Thomas Woodhouse, Andras Farkas, Maria Hobrok, Rhona Kilpatrick, Matthew Newman, Aalap Asurlekar, Stephen Dalgleish, Peter Davies, Joseph Farrimond, Elizabeth Lindsay, Joe Littlechild, Mariam Malik, Suad Nimale, Vaila Robertson, Nicole Robin, Wan Nee Shue, Robert Sinnerton, Giles Faria, Adam Fell, Ifeanyi Kem Onubogu, Jai Relwani, Oluwaseun Adeboyejo, Mandeep Singh Bindra, Kim Borsky, Muhammad Umar Fawad, Shoaib Fahad Hussain, Fadi Issa, Aiman Jamal, Catriona Luney, Neale Marlow, Aman Sethi, Haleema Siddique, Abin Varghese, James Vaz, Demin Li, Caitlin Rigler, Rajen Tailor, Matthew Bye, Elorm Daketsey, Jane Elford, Rahela Islam, Rosalind Jones, Tharangani Kathiravan, Sujatha Kumari, Kavitha Mattam, Richard Morris, Daniel Murray, Amy Nixon, Carwyn Roberts, Zoe Seymour, Arshad Siddiqui, Chris Smith, Lianne Stevenson, Odai Yaghi, Asem Almaghrebi, Josephine Kahiu, Nikhita Patel, Anish Koneru, Emma Long, Ayesha Rahman, Selina Ravenscroft, Tobias Stedman, Gianina Tutoveanu, Alex Ward, Lucy Wibmer, Katie Cross, Mohammed Fakhrul‐Aldeen, Kayleigh Spellar, Shahrukh Ahmad, Joshua Boyes, John Ferns, Matthew Freudmann, Saqib Ghumman, Siddhartha Handa, Abdul Jalil, Manish Kaushal, Panna Patel, Peter Sodde, Sachith Sreenivasan, Sook Han Yee, David Ensor, Mark Gotecha, Shaikh Sanjid Seraj, Premalatha Sharavanan, Anuhya Vusirikala, Rishi Das, Nikhil Ponugoti, Rosalind Grace Beckett, Soraya Conroy, Joel Corkill, Angharad Davies, Katie Gilmore, Lysander Gourbault, Thomas Parsons, Tobias Roberts, Beibit Bashabayev, Cristina Croitoru, Emmet Dorrian, Michael McGreevy, Niamh Murphy, Firas Aljanadi, Joseph Doyle, Hashim Elshibly, Reubendra Jeganathan, Mark Jones, Jamal Khan, Maryam Khan, Alexandra Lee, Nyasha Mutsonziwa, Rebecca Reid, James Archer, Christopher Bache, Andre Cardoso Almeida, Abdulrahman Odeh, Max Pachl, Rebecca Schembri, Anna Gateley, Victoria Hodgetts Morton, Helen van Vliet, Audrey Kwong, Elaine Leung, Sudha Sundar, Megan Williamson, Irshad Ahmed, Abdulrahman Alsaggaf, Amarbaj Chandock, Nnaemeka Chidumije, Sharad Karandikar, Tabassum Khan, Arab Rawashdeh, Ali Sallam, Rishi Singhal, Syed Osama Zohaib Ullah, Ruhina Alam, Yvonne Asiedu, Aneel Bhangu, Alina‐Maria Budacan, Shafiq Ahmad Chughtai, Anant Desai, Anvay Deshpande, Mohammed Farid, Samuel Ford, Wiliam Foy, Adam Gittins, Ewen Griffiths, Jonathan Herron, Michael Howells, Rizwana Imran, Tobias James, Sivesh K Kamarajah, Rugved Kulkarni, Tsun Yu Kwan, Catherine Leng, Andraay Leung, Ansar Mahmood, Thomas McLelland, Paul Nankivell, Alessandro Parente, Ishan Radotra, Keith Roberts, Bhaskar Satsangi, Neil Sharma, Manjunath Siddaiah‐Subramanya, Muhammad Harris Siddique, Thomas Smith, Wai Cheong Soon, Veena Surendrakumar, Fabio Tirotta, Salem Jamhour, Dhanyata Narendra, Suresh Panchakshariah, Narendra Babu Siddaiah, Mohamed Albendary, Edward Balai, Alvaro Bedoya‐Ronga, Felicia Elena Buruiana, Ali Yasen Y. Mohamedahmed, Lopa Patel, Rajeev Peravali, Shahin Qadri, Miski Scerif, Abdul Sillah, Jenny Wright, Laith AlSaket, Jayant Cherukat, Mohamed Saleem Noor Mohamed, Arab Rawashdeh, Rishi Singhal, Nauman Ahmed, Clarissa Ern Hui Fang, Abin Holla, Zayne Mahmud‐Ahmad, Mohammad Masaarane, Surya Narayan, Pornjittra Rattanasirivilai, Irena Shiderova, Asma Sultana, Silvian Tan, Hasan Usmani, Osborne Peter Vaz, Jonathan Barker, Mohammed Elniel, Lucy Fell, Fatma Khan, Geerthan Nagachandra, Lara Rimmer, Utkarsha Basu, Tsitsi Chituku, Kingsley Lasing, Devaraj M Navaratnam, Milind Rao, Aliaa Shamardal, Athula Tennakoon, Nisar Haider Zaidi, Sadaf Zehra, Thomas Baker, Genevieve Brixton, Rotimi David, Tobias Klatte, Agata Majkowska, Joanna Manson, Ryan Potter, James Yea, Muntadhir Al‐uzri, Omar AlShakhshir, Jordan Bickerdyke, Peter Bobak, Victoria Brown, Alexios Dosis, Mohamed Hashem, Max Hayden, Harry Hodgson, Irfan Jumabhoy, Fiona Langlands, Kenneth Linton, Mariana Matias, Amelia Milton, Hannah Louise Morley, Frances Mosley, Christopher Raine, Matthew Rose, Amy Round, David Selwyn, Isabel Slark, Alexander Sumpner, Jonathan Wareing, Sam Wareing, Jessica Watson, Claire Winton, Ashish Gupta, Rahi Karmarkar, Lavinia Margarit, Rucira Ooi, Amr Shalaby, Shahrima Sheefat, Aurelia Vas, Islam Zarad, Mazin Mohamed, Chloé Monnier, Mohamed Radhi, Mai Baquedano, Catherine Bradshaw, Massimo Caputo, Lakith Kapuge, Benjamin Martin, Filippo Rapetto, Mohamed Shalaby, Ella Teasdale, Sarah Biggs, Natalie Blencowe, Abigail Campbell, Florence Caslake Holding, Ffion Dewi, Ettorino Di Tommaso, Lauren Dixon, Ysabelle Embury‐Young, Gustavo Guida, Kathryn Hogan, Lucy Huppler, Caitlin Jordan, Thomas Andrew Maccabe, Catherine Macleod‐Hall, Tina Anto Menachery, Samir Pathak, Ryan Preece, Reesha Ranat, Jonathan Randall, Sophie Rozwadowski, Douglas West, Jack Bellerby, Matthew Chan, James Cullen, Richard Donovan, Tomos Edwards, Rhiannon Frostick, Kate Gregory, George Higginbotham, Kalina Hristova, Kueni Igbagiri, Tarik Jichi, Rhys Luckwell, Sophie McDonald, Joshua Moreau, Dimitri Pournaras, Sarah Shiels, Thomas Tilston, Matt Towner, Agnes Hamilton‐Baillie, Neil Ryan, Adewale Ayeni, Najam Husain, Keren Pathmanathan, Olatoyosi Williams, Aneish Mangarai, Ashwini Virgincar, Amina Akhtar, Rohan Ardley, Evripidis Tokidis, John Hardie, Sarmad Kazzaz, Stratton King, Jayson Roberts, Karim Abdelraouf Moawad, Amit Agrawal, Abdulrahman Al‐Mohammad, James Ashcroft, Susannah Ashfield, Alan Askari, Anita Balakrishnan, Deepika Bhojwani, Garance Biosse‐Duplan, Patrick Coughlin, Nick Dai, Richard Justin Davies, Henry Dunne, Amer Durrani, Michael Feretis, Daniel I. Fernando, Alice Fae Ferreira, Brian Fish, Fanourios Georgiades, Marios Ghobrial, Andr eas V Hadjinicolaou, Rob Hammond, Sarah Hardwick, Yaldasadat Hashemipour, Thusitha Hettiarachchi, Victoria Hudson, Amy Huseyin, Peter Hutchinson, Ekpemi Irune, Claire Jackson, Asif Jah, Niel Kang, Wasim Khan, Maaz Khan, Angelos Kolias, Harry Kyriacou, Siong‐Seng Liau, Andre Chu Qiao Lo, Samuel Long, Liam Masterson, Claudia Gabriela Mitrofan, Ahmed M. H. A. M. Mostafa, Suzanne Murphy, Robert O’Neill, Reece Patel, Mohamed Rabie, Will Raby‐Smith, Siobhan Mairead Rooney, Sabrina Helena Rossi, Neil Russell, Eniola Salau, KT Matthew Seah, Nicholas Segaren, Farakh Shahzad, Arnaldo Neves Santos Silva, Aminder Singh, Jeremy Solly, Harry Spiers, Grant D. Stewart, TO Kendrick, Adam Townson, Rebekka Troller, Sara Venturini, Catriona Walker, James Wheeler, Christopher Y.K. Williams, Kai Yuen Wong, Athanasios Xanthis, Navid Ahmadi, Olivia Baker, Raisa Bushra, Aman Coonar, Fabio Falconieri, John Hogan, Asanish Kalyanasundaram, Anum Mir, Eyal Ran Nachum, Maria Nizami, Mohamed Osman, Dulan Samaraweera, Daniel Sitaranjan, Christopher Smith, Ismail Vokshi, Sze Hui Wong, Emma Rose Michelle Woolcock, Roa Hassan, Nikhil Math, Mark Yao, Saad Azher, Tom Combellack, Natasha Harley, Ghis Tahhan, Idrees Ahmed, Christopher Bowler, Sarah Choi, Elinor Davies, Victoria Evans, Rhodri Evans, Gemma Harrell, Andrew Hughes, Robert Mcleod, David Owens, Michael Shinkwin, Samuel Stevens, Tosin Akinyemi, Funbi Ayeni, Khalid Bhatti, Ruben Canelo, Basem Hamzah, Syed Mannan, Charlotte Bee, Pawan Kumar Dhruva Rao, Ishtiak Mahamud, Riyam Mistry, Arjun Paramasivan, Gabriela Bocsa, Andrew Brown, Conor Carroll, Bhargavi Chandrasekar, Arthur Day, Sophie Dodd, Nicola Eardley, Emma Ewins, Lucy Fuller, Sukhpreet Gahunia, Parmilan Gill, Joseph Hanna, Tristan Heath, Christopher Khoory, Emily Krishnan, Weisang Luo, Nichola Manu, Leo Maric, Emmeline Martin, Alex Moore, Sunanda Roy Mahapatra, Olivia Lumiap Serevina, Dale Vimalachandran, Rosanne Ching, Zainab Dhorat, Hal Munton, Shiva S Tripathi, Reem AlAbdulwahed, Amanda Czyz, Adeoye Oluwakanyinsola Debo‐Aina, Aniket Deshpande, Hadyn K.N. Kankam, Dana Low, Benjamin Paolini, Aswathy Pavithran, Sunita Saha, Sreelakshmi Suresh, Anjelli Wignakumar, William Campbell, Martin King, Sarah Small, Haneen Abed, Mariam Baig, Phoebe Brobbey, William Carlos, Danny Chandla, Timothy Davis, Fatema Dhaif, Kathryn Dickson, Peter Goodwin, Sarah Henning, David Izadi, David Jeevan, Valdone Kolaityte, Maria Kolokotroni, Nicola Mackay, Adam Pilarski, Jenardan Sellathurai, Smruta Shanbhag, Alastair Stephens, Arthika Surendran, Thomas Ward, Doaa Zeidan, Mohammed Balbola, Chi Hoi Lee, Jimmy Mena, Emila Paul, Antony Bateman, Ash Bhalla, Hasan Daoud, James Davies, Gareth Davies‐Jones, Abdulla Ebrahim, Lucy Gossling, Amit Goyal, Ahmed Hamad, Hannah Javanmard‐Emamghissi, Anuja Joshi, Joanne McKay, Emma Ogden, Ziyan Sheng, Lavanya Umashankar, Lotte Weenink, Emily Weisfeld, Emmanuel Adebajo, Sorour Borayek, Mark Cribb, Nabil Elmaleh, Emily Heywood, Omar Mulla, Omar Ahmed, Tasleem Akhtar, Kerrie Aldridge, Vishal Amin, Jonathon Dawson, Rachael Dolan, Zi Hao Reuel Heng, Alexander Hollis, Akash Jangan, Jo Lau, Aamer Mughal, Jeremy Newman, Rachel Olive, Shayan Shahidi, Atif Sharif, Julian Sonksen, Arooj Syed, Isaac Wahnon, Michael Wall, Stephanie Weedon, Jeyakumar R Apollos, Megan Kerr, Muneeb Zafar, Sheng Wei Chiam, Stephen Davison, Laura Doherty, Christopher Donoghue, Alastair Faulkner, Yong Kiat Goh, Katie Hoban, Jeremy Lee Jun Shern, Alasdair MacInnes, Jaiganesh Manickavasagam, Rebecca McNicol, Sudhara Niriella, Eimear O’Connell, Ramaa Parulekar, Kalpana Ragupathy, Mohammad Rahman, Arthur Chen Wun Tan, Kimberly Tiang, Lynn Darragh, Jennifer Foreman, Ian McAllister, Cherith Semple, Sathyaseelan Arumugam, Tom Collicott, Gargeshwari Krishnamurthy Guru Raghavendra, Khizar Khan, Cho Ee Ng, Irvita Sharma, Shreyas Supparamaniam, Sabina Wallace‐King, Paul Cullis, Beniamino Forte, Phillip Holt, Merrill McHoney, Abigail Semple, Caitlin Brennan, Paul Brennan, William Cambridge, Thomas Drake, Andrew Durden, Mark Galea, David Henshall, Gustav Linder, Richard McGregor, Joshua McIntyre, Gabriel Metcalf‐Cuenca, Samuel Molyneux, Joel Norton, Jay Jaemin Park, Alexandra Rice, Lydia Robb, Lauren Ross, Cameron Simpson, Richard JE Skipworth, Gordon Snowden, Andrew Tambyraja, Daniel Thompson, Gareth Turnbull, Marianne Watters, Tim White, Connor Boyle, Filip Brzeszczyński, Katherine Hodge, Alexander Laird, Yasuko Maeda, Hugh Paterson, Scott Smith, Peter G Vaughan‐Shaw, Mariam Ahmed, Asuvathan Aravinthan, Markus Baker, Zachary Baxter, Miles Benjamin, Anupama Jeyakumar, William Lloyd, Mana Rahimzadeh, Nithin Thoppuram, Katerina Anesti, Morgan Bayley, Eleanor Burden, Joanne Cozens, Urszula Donigiewicz, Douglas Ferguson, Alex Goubran, Thomas Hubbard, Wei Jia, Matthew Jukes, Edward David Lumley, Lisa Massey, Frank McDermott, John McGrath, Aye Myintmo, Michael Ng, Katie‐Louise Parker, Jonathan Phillips, Heather Pringle, Niroshini Rajaretnam, Amin Siddig, Oliver Small, Guang Yim, Sadia Afzal, Kais Al Suyyagh, Gemma Faulkner, Michael Greenhalgh, Ghazal Hodhody, Joann Lum, Olivia McCabe‐Robinson, Joseph McKay, William Ryan, Bethan Salmon, Chloe Smart, Henry St Aubyn Bilton, Matthew Walmsley, Patrick Watmough, Leo Watton, Barry Dent, Hannah Emerson, Porfyrios Korompelis, Wendy McCormick, Stuart Rundle, Kiran Singisetti, Karen Vejsbjerg, Cara Vincenti, Thomas Watkinson, Benjamin Wormald, Daniel Carson, David Holroyd, Nigel Jamieson, Marina Nagiub, Jonathan Pugh, Rachel Thomas, Rachael Boardley, Elsie Bridgman, Mustafa El Sheikh, Simon Lammy, Mark Danton, Ciaran Doherty, William Flynn, George Gradinariu, Ed Peng, Joseph Atley, Molly Bradbury, Simon Dwerryhouse, Vipul Garg, Rajesh Gopireddy, Lydia Newton, Tsi Njim, Muni Pinjala, Manuk Wijeyaratne, Alaa Awad Hussein Ameri, Bence Atkari, Gakul Bhatta, Monica Bogdan, Christopher Chien Liang Liao, Joe Hwong Pang, Laura Parry, Vamsi Velchuru, Mei Ying Chin, Abi Hayward, Salasiah Othman, Anna Sayers, Soorya Siva, Ahmed Abubakar, Claudia Dyball, Manal Javid, Taran Khurana, Kar Yeung Kenneth Ko, Stephen McAleer, May Ting Tan, Jonathan Horsnell, Thumuluru Kavitha Madhuri, Emily Moore, Isabella Murray, Jamie Patel, Alex Tan, Farah Akthar, Emily Crane, Rishi Dhir, Praveen Gopinath, Sofia Green, Mohamed Habad, Salah Hammouche, Dalia Hammouche, Manal Jmaileh, Tjun Wei Leow, Htoo Lu, Jehangir Mahaluxmivala, Qasim Sohail, Catrin Sohrabi, Simon Wimsey, Oliver Cohen, Christopher I’Anson, Matthew Wright, Hani B Abdul‐Jabar, Sibusiso Ndlovu, Matthew Reid, Louie Lynn Sajorda, Muhammad Harris Siddique, Abhinav Singh, Cosimo Alex Leo, Carolynne Vaizey, Janindra Warusavitarne, Lucy Maling, Sheena Seewoonarain, TO Christopher, Abdelmonem Hassan Eid Abdelmonem, Mustafa Al‐Yaseen, George W V Cross, Krisztian Deierl, Vivek Dubey, Ahmad Ja’far, Riana Patel, Sybghat Rahim, Parisah Seyed‐Safi, Yat Wing Smart, Emad Soliman, Jija Thomas, Phillipa Beesley, Michael Biggs, Peter Davies, Laura Deacon, Laura Garden, Sean Howells, Megan Oldbury, Faazil Ahmed, Shereen Ajab, Vasileios Arzoglou, Alice Ball, Alison Cairns, Megan Chrysikopoulou, Amit Deshmukh, Ghazi Elshafie, Momin Eltayeb, Alasdair Findlay, Francesca Gatta, Mithila Govind, Jessica Harvey, Benjamin Huggon, Shah Jehan, Navid Kabuli, Eiman Khalifa, Sameera Khaliq, Mahmoud Loubani, Matthew Robert Marples, Rhona Martin, Adam McClean, Gautam Modak, Shahani Nazir, Sandhya Od, Andrew Parrish, Abirami Perumal Kanniappan, Andrew Quin, Piravin Kumar Ramakrishnan, Ritika Rampal, Brianda Ripoll, Razan Rislan, Robert Taylor, Dwarakesh Thalamati, Joshua Totty, Ben Ward, Arun Watts, Alex Wilkins, Jih Dar Yau, Shobhit Arya, Adeela Ashraf, Aman Bhargava, Shamira Hassan, Elizabeth Kmiotek, Suzette Samlalsingh, Georgios Karagiannidis, Evangelos Mallidis, Fahed Youssef, Tariq Alhammali, Pamela Dawson, Waleed Fahmy, Benjamin Gowers, Naren Kumar, Hannah Lennox‐Warburton, Michelle Lynch, Adam Mohammad, Salam Musa, Arnav Sahu, Sharan Sambhwani, Nomaan Sheikh, Ormond Mark Taylor, Michelle Wright, Marwa Al‐Azzawi, Nicola Hall, Michael Helley, Iain Hood, Emma Howie, Shannon Jordan, William Norton, Charlie Saunders, Duncan Stickle, Euan Toshney, David Watt, Andrew Whitfield, Ahmad Al‐Shaye, Ahmad Al‐Sukaini, Ahmad Attia, John Britton, Theo Clarke, Simona Ippoliti, Ignatius Liew, Gabriella Weisz, Ampere Yiu, Hesham Youssef, Areej Abdel‐Fattah, Muhammad Adeel Akhtar, Hwan Heo, Mhairi Clark, Gareth Clarke, Benjamin Parkin, Fraser Rae, Calum Sreenan, Roxane Stienstra, Lindsay Wallace, Michael Wilson, Salman Arif, Anupama Barua, Michael Blackwell, Grainne Bourke, Conor Bowe, Mahbub Chowdhury, Elliott Cochrane, Raquel Constantino‐duarte, Sophie Earl, Ikechukwu Ejiofor, Walid Elmahdy, Betsy Evans, Antonella Ferrara, Alice Fort‐Schaale, Claire Hardie, Michael Ho, Azar Hussain, Neeraj Kalra, Anastasios Kanatas, Chung Yan Vernon Lee, Stefan Louette, Ryan Mathew, Stephanie Milne, Emily Salt, Priya Shankar, Alexander Spyridoulias, Peter Szedlak, Josh Twigg, Ryckie Wade, Christopher West, James Wright, Antonios Athanasiou, Adam Barlow, Elaine Borg, Luke Bradshaw, Alessandro Brunelli, Abbigayle Buckton‐Perkins, Gemma Burdge, Eleanor Clarke, Peter Coe, Camilla Davies, Elizabeth Doxford‐Hook, Sheila Fraser, Joanna Gibson, Andrea Giorga, Konstantinos Gioutsos, Sunjay Jain, Angelika Kaufmann, Alexandros Laios, Susanna Lam, Terence Lo, Marie Lucas, Emma MacInnes, Sushil Maslekar, Catherine Moriarty, Christopher Nahm, Mohamed Otify, Adam Peckham‐Cooper, Cecilia Pompili, Charmian Reynoldson, Phoebe Robertson, Loren Rushton, Lucy Scott, Nanaki Singh, Helen Skinner, Giles Toogood, Danielle Trigg, Louise White, Jessica Whitney, Robert Wickstead, Alastair Young, Shahbaz Abdullah, Metesh Acharya, Nina Al‐Saadi, Keng‐Leong Ang, Rebecca Boyles, Edward J Caruana, Karishma Chandarana, Ravi Chotalia, Mohammed Fiyaz Chowdhry, Rachel Chubsey, Chiraag Thakrar Karia, Emmanuel Katsogridakis, Marinos Koulouroudias, Kudzayi Kutywayo, Kaylan Lad, Dominic Lam, Giovanni Mariscalco, Rayhaan Mohammed, Apostolos Nakas, Anishka Pabari, Shriyam Patel, Sridhar Rathinam, Athanasios Saratzis, Matthew Stephens, Evangelia Ioanna Tsiourva, Mustafa Zakkar, Alison Armstrong, Robert U Ashford, Wen Jie Chin, Srujan Rajesh, Oliver Lauridsen Siaw, Baljit Singh, Alex Boddy, Vijay Chavda, Neil Flint, Irina Georgieva, Mark Higgins, Jitendra Mangwani, Prashant Naik, Ninad Nigalye, Jvalant Nayan Parekh, Kathleen Wolff, Cameron Kuronen‐Stewart, Beatrix Weber, Zubair Ahmed, Joseph Attard, Amirah Azhar, Nikhil Kulkarni, Lydia Prusty, Dinesh Thekkinkattil, George Garas, Peter Gaskell, Asif Hasan, Terence Jones, Arjun Kattakayam, Christopher Loh, Raimundas Lunevicius, Natalie Maple, Stylianos Papalexandris, Shirley Pringle, Alasdair Santini, Andrew Schache, Richard Shaw, Ajay Sud, Harriet Corbett, Samuel Ebbs, Sian Falder, Margaret Hanley, Rachel Harwood, Rong Khaw, Anna McNamara, Ijeoma Okonkwo, Arvind Rammohun, Muhammad AbdulHakeem, Syed Al Nahian, Mark Field, Amer Harky, Ronan Kelly, Bilal Kirmani, Abinash Panda, Florentina Luiza Popescu, Mostafa Snosi, Ahmed Torky, Mauin Uddin, Zamesa Paulino Nathaniel, Shakil Ahmed, Kiran Altaf, Ryan Baron, Hannah Barrow, Philip Cornford, Declan Dunne, Hannah Earnshaw, Raja Eid, Jeremy Guilford, Lydia Hawker, Stephen Kaye, Rebecca Lenihan, Rachel McKinney, Luca Pagano, Rohith Rao, Tom Rayner, Vito Romano, Susannah Shore, Dana Sochorova, Robert Sutton, Francesco Torella, Thomas Ward, Chloe Ward, Simon Clark, Tarek Elmoslemany, Michael Jenkinson, Kevin Kelly, Vijay Kumar Kumar, Chris Lemos, Talanayar Mahalingam, Christopher Paul Millward, Vijayam Priya Nair, Vinita Sangai, Sarbpreet Sarao, Rajesha Srinivasaiah, Sudhir Venkataramaiah, Dominique Hughes, Luca Lancerotto, Lucy Li, Madlen Dewi, Eshan Mazumdar, Dema Motter, Jennifer Reid, Tony Chen, Isobel Hatrick, Holly Lyle, Freyia Mahon‐Daly, Niarah Ahmad, Guy Benshetrit, Zoe Burdon, Rupen Dattani, Nina Jyne Minette Dela Cruz, Jordan Faulkner, Madeleine Garner, Catrin Morgan, Rebecca Morgan, Toby Noton, Tanzeela Gala, Tom Georgi, Christopher Mifsud, Sujan Patel, Rose Stahl, Stella Vig, Muhammad Usman Ali, Sarah Kelly, Lokesh Lokesh, Michaela Paul, Daniel Shaerf, Hemant Sheth, Fiammetta Soggiu, Charlotte Holbrook, Ijeoma Nwachukwu, Katie Adams, Maame Aduse‐Poku, Tamara Alexander, Gill Arbane, James Arlidge, Craig Bailey, Melissa Baldwin, Heena Bidd, Aina Brunet‐Garcia, Gary Colville, Alexa Curtis, Christopher Delaney, Yasser Diab, Toby Dixson, Mothana Gawad, Siew‐Ling Harrison, Christopher Holt, Craig Johnstone, Findlay MacAskill, Sarah McCrindle, Kate Millar, Osama Naji, Rafal Niziol, Leyla Osman, Arun Sahai, Abegail Salvana, Ahmad Sayasneh, Michael Shaw, Sarah Tian, Annabelle White, Danny Jon Nian Wong, Valerie Wong, Christina Fotopoulou, Jennifer Ploski, Srdjan Saso, Katina Theodoropoulou, Alexandra Valetopoulou, Keyoumars Ashkan, Kathleen FM Fan, Angela Hancock, Josephine Jung, Maria Alexandra Velicu, Zoe Williams, Elizabeth Yeung, John Dabis, Ryan Geleit, Ian Gill, Thomas Holme, Nardeen Kader, Irfan Merchant, Akarshan Naraen, David Rawaf, Nashat Siddiqui, Qurrat Al Ain Atif, Rory Cuthbert, Cameron Dott, David Ferguson, Lana Huang, Priyanka Iyer, So Hee Kim, Srinath Ranjit, Varun Sarodaya, Lucy Tomasetti, Tayyub Yasin, Syed Zain Bukhari, Mamun David Dornseifer, Manojkumar S Nair, Mohit Bhatia, Basel Haddadin, Rehana Hafeez, Ahmed Mansy, Ahmed Mattar, Frank Smedley, Shihab Chowdhury, Brian Davidson, Nikolaos Dimitrokallis, Giuseppe Fusai, Jack Gilliland, Camila Hidalgo Salinas, Ioannis Kostakis, Reza Mirnezami, Reza Motallebzadeh, Krishnakumure Patel, Helen Quah, Massimo Varcada, Anam Anzak, Abhirup Banerjee, Louis Boyce, Rhiannon Brignall, Laura Clementoni, Evangelos Efthimiou, Sarah Epton, Martina Faimali, Despoina Iakovou, Jayan Dewantha Jayasinghe, Inês Leal Silva, Kaifeng Liang, Cortland Linder, Annamaria Minicozzi, Lalin Navaratne, Zoi Nikoloudaki, Henry Nnajiuba, Mohamed Hazem Okail, Elliot Onochie, Sankhya Prakash Vel, Rajesh Sivaprakasam, Catrin Sohrabi, Mohamed Adhnan Thaha, Bhaskar Thakur, Kalpesh Vaghela, Rosie Yeoward, Vincent Yip, Sibtain Anwar, Teofila Bueser, Aung Oo, Julie Sanders, Ashley Thomas, Marta Wachtl, Mateusz Zawadka, Mohamed Abouelazayem, Raluca Belchita, Caroline Hing, Nicholas Judkins, Antonio Leyte Golpe, Preemal Patel, Gowthanan Santhirakumaran, Jeremy Smelt, Carol Tan, Dimitrios Tsironis, Anita Eseenam Agbeko, Joachim Amoako, Chang Park, Khaled Sarraf, Joseph Shalhoub, Kapil Sugand, Jochem Caris, Hemant Kocher, David Choi, Ciaran Hill, Chan Hee Koh, Hugo Layard Horsfall, Hani Marcus, William Muirhead, Poppy Alport, Peter Barry, Charlotte Benson, John Butler, Hannah Dawson, Paula Fagan, Jonathan Hannay, Andrew Hayes, Anna Heeney, Robin Jones, Cyrus Kerawala, Erik Mayer, Anissa McClelland, Rachel O’connell, Edward Phillips, Gausihi Sivarajah, Myles Smith, Alannah Smrke, Dirk Strauss, Calisha Allen, Ivy Lim, Max Little, Chetan Parmar, Alexandra Sharpe, Benjamin Zakaria, Nicholas Kalavrezos, Natalie Marzouqa, Deepti Sinha, Tom Abbott, Stefano Andreani, Michael Bath, Mona Behravesh, Brendan Berry, Emma Collins, Isabella Drummond, Isabelle Farrow, Ora Jesner, Stephanie Kwok, Valerie Lan‐Pak‐Kee, Sriveena Naganathar, Patrick O’Hagan, Amar Odedra, Funlayo Odejinmi, Christine Ozone, Laura Roberts, Lesley Sheach, Matthew Short, Shawn Jia Hwang Tan, Zoe Thursz, Charlotte Trainer, Zaker Ullah, Md Tanveer Adil, Safia Ahmed, Chelise Currow, Jonathan Dennis, Alistair Hardy, Haidee Harrison, Anna Kaleva, Aneesah Khan, Shantata Kudchadkar, Minil Patel, Meysoon Qurashi, Amit Raithatha, Jayesh Sagar, Rishi Talwar, Ali Tarfiee, Ziad Al‐Khaddar, Farhat Naaz Amir, Mumtaz Bughio, Fares Ftaieh, Wut Hmone, Andrew Kennedy‐Dalby, Soumitra Mukherjee, Sara Nausheen, Christopher Smart, Dinesh Balasubramaniam, Balaji Jayasankar, Eoin O’Farrell, Mahmud Riad, Louai Abdeh, Saravpreet Ahluwalia, Kailash Bhatia, Natalie Cheyne, Haris Naseem, Gopalkrishna G Verma, Eilidh Waddell, Moez Zeiton, Numera Arif, Nabeel Cheema, Usman Halim, Charlie Moret, Paul Peters, John Michael Ranson, Jigar Shah, Andrew Wheelton, Ethan Clough, Henry de Berker, Sara Faily, Paul Farrelly, Helen Figgins, Ana Jeelani, Matthew Murphy, Jaya Nichani, Lucinda Pigott, Mamta Shah, Gabrielle Thompson, Jonathan Yates, Jade Chen Zhao, Sujesh Bansal, Kailash Bhatia, Swarnendu Dey, Ellie Humphry, Rahul Norawat, Noha Tageldin, Mohammed Abdelaziz, Moslem Abdelghafar, Ikenna Anderson Aneke, Thomas Bradley, Benjamin Brown, Katie Campbell, Charles Carey, Daniel Coakley, Natasha Elson, Verity Haffenden, Sam McNally, Shahd Mobarak, Emma Murray, Bradley Pittam, Michael Preston, Carlo Ross, Safdar Sarwar, Md Abu Sayed, Nazanin Shahrokhi, Catriona Shenton, Sahiba Singh, Anuradha Venugopal, Ashwin Bhadresha, Chu‐Hao Chiang, Sean Garcia, Rakesh Koshy, Mubasher A Qamar, John Adam, James Hadfield, Igor Maleyko, Amar Rangan, Andrea Sangheli, Srivishnu Thulasiraman, Anil Varma, Yks Viswanath, Alexander Martin, Sotiris Mastoridis, Chin Hang Sophia Sin, Anjana Singh, Rachel Soulsby, Amanda Taylor, Wai Huang Teng, Ruben Thumbadoo, Junaid Aamir, Mustafa El‐lami, Mandeep Koundu, Louise Le Blevec, Ebrahim Mahomed, Connor McGladdery, Samuel Newman, Lysia Richmond, Francesco Abbadessa, Corey Chan, Nicholas Dawe, Chathuranka De Silva, Mariantonia Ferrara, John Hammond, Roxane Hillier, Mustafa R Kadhim, John Moir, Stephanie Myszkowski, Thomas Salisbury, Peter Sudworth, Samuel Tingle, Timothy Williams, Lauren Best, David Bosanquet, Amisha Cameron, Ryan Chiang, Natalie Duric, Arthur Ee, Sarah Elgarf, Hawys Freeman, Tracy James, Dominic Knight, Owen Lewis, Steffan Morgan, Ellie Morgan, Alexandra Munteanu, Setthasorn Zhi Yang Ooi, Sajitha Parveen, Filip Sianos, Ishani Sinha, Sumi Surendran, Tamas Szakmany, Harriet Whewell, Dimitrios Angelou, Rachael Crompton, Maebh Doohan, Amy Doran, Jonathan Hagan, Katie McCaughey, Mark McKeever, Maria Pantelidou, Reem Salman, Samar Shalaby, Richard Thompson, Avinash Aujayeb, Rebecca Critchley, Benjamin Emmerson, John Guirguis, Karl Jackson, Nicole McLaughlin, Anna Porter, Ebru Freed, Marcu Vasilica, Dinnish Baskaran, Oliver Claydon, Rachael Collins, Raashad Hasan, Zahra Hussain, Ahmar Iftikhar Talib, Jian Shen Kiam, Vasileios Kouritas, George Lafford, Sam Norton, Joshua Ozua, Aravindh Ramalingam, Haisam Saad, Sari Samman, Bhavesh Tailor, Teri Toi, Daniel Watts, Sara Alsaad, Pritpal Aujla, Nathan Burnside, Sarah Cadwell‐Sneath, Zoe Chia, Michelle L Collins, Simon Craxford, Nivedita Das, Brett Doleman, Ahmed M El‐Sharkawy, Nathalie Fennell, Ketankumar Gajjar, Catherine Grundy, Karen Hassell, Mohammad Hawari, Adeel Ikram, Trisha Kanani, Harmanpreet Kaur, Zubair Khanzada, Maisie Lau, Ben Marson, Alastair Morton, Francis Githae Muriithi, Benjamin Ollivere, Simon Parsons, Oliver Pumphrey, Rebecca Rollett, Laura Sandland‐Taylor, Kathryn Steele, Muhammad Sarmad Tamimy, Elena Theophilidou, Nathan Tyson, Ravinder Vohra, Rini Vyas, Sven Watmore, Helen Weaver, Lisa Whisker, Fady Yanni, Asad Abbas, Alfred Adiamah, James Bailey, Ayan Banerjea, Balamurali Bharathan, James Blackwell, Catherine Boereboom, Hilary Brewer, Iain Cameron, Abeed Chowdhury, Christopher Deacon, Charis Demetriou, Edward Dickson, Andrew Edwards‐Bailey, Jonathan Evans, Sarah Fendius, Laurence Glancz, Dhanwant Gomez, Gordon Gregory, David Humes, Adeel Ikram, Jamaall Jackman, Aseel Khanfer, Amanda Koh, Christopher Lewis‐Lloyd, Ben Marson, Neil Mathias, Eyas Mohamed, Daniel Morris, Yulanda Myint, Gael R Nana, Yasar Nassif, Alex Navarro, Jimmy Ng, James Chean Khun Ng, Alan Norrish, Benjamin Ollivere, Olamide Oyende, Mohammed Patel, Melroy Rasquinha, John‐Joe Reilly, Sudip Sanyal, Jaspreet Kaur Seehra, Anneka Shah, Paul Thomas, Kathryn Thomas, Amari Thompson, Benjamin Varghese, Dawit Worku, Fadzlien Zahari, Akshath Adapa, George A. Antoniou, Mohammed Ashrafi, Bhalchandra Bhalerao, Daniel Cohen, Daniel Doherty, Matthew Gray, Tom Havenhand, Ioanna Kyrou, Siddharth Lokanathan, Ashwani Nugur, Thomas Banks, Manikandar Srinivas Cheruvu, Paul Cool, Debashis Dass, Salam Ismael, Helen Beech, Matthew Byrne, Pengchi Chen, Neel Doshi, Stefanos Gorgoraptis, Ali Asgar Hatim Ali, Lamiese Ismail, Jerocin Vishani Loyala, Sotiris Mastoridis, Naomi Neal, Benjamin Ng, Orna Ni Bhroin, Muna Patell, Omar Ben Forge Risk, Hooman Soleymani Majd, Stuart C Winter, Ho Ying Flora Wong, Roshneen Ali, Abdur‐rafee Ameen, Elizabeth Belcher, Christopher Bretherton, Hamez Gacaferi, Mario Ganau, Matej Goricar, Anne Hughes, Deva Jeyaretna, Gs Jutley, Nikhil Lal, Balint Viktor Lovasz, Chris McKinnon, John McNamara, Benjamin Ng, Claudia Paul, Puneet Plaha, Ganeshan Ramsamy, Karishma Shah, Sanskrithi Sravanam, Vikas Sud, Max van Essen, Kate Wallwork, Mohamed Hassan Fathy Hassan Abdallah, Sher Aslam, Khurram Ayub, Bhuvanshyam Bhaktavatsalam, Joanna Carvalho, Benjamin Dean, Stevan Jordan, Richard Myatt, Jonathan Norris, Muhammad Ather Siddiqi, Fraser Thomson, Ala Alasadi, Ellen Groundwater, Carina Banziger, Jamie Deyell, Lauren Donnelly, Thomas Samuel William Greensmith, Thomas Harding, Laura Inglis, Dominic Lee, Esther Oyewusi, Rachel Pennington, Christie Hok Yung Shum, Zack Slevin, Ryan Tan, Shivanie Acharya, Pantelis Amditis, Sanjay Asopa, Supriya Balasubramanya, Aladdin Bashir, Maham Khan, Sathya Lakpriya, Joshua Lau, Rachel Marshall‐Roberts, Douglas Miller, Sarah Miller, Nader Moawad, Hossam Nawara, Hope Nwinee, Luke Rogers, Sera Sarsam, Simran Singh, Sebastian Smolarek, Zaheer Tahir, Andrei Tanase, Aye Chan Thu, Zuzanna Wieczorek, Lu Yao, Ioannis Biliatis, Lara Armstrong, Jessica Lockhart, Kevin McElvanna, James Ashbridge, Fanny Belais, Janet Berry, Nadine Di Donato, Matthew Dipper, Jim Khan, Charlotte Parfitt, Samuel Stefan, Rajeev Advani, Stephen Canty, Madhu Chaudhury, Mariya Dunbobbin, Leanne Dupley, Rukmini Ghosh, Isabel Hughes, Priyatma Khincha, Ana Pardilho, Edward Parkin, Pradnya Patkar, Adrian Pearce, Isaac Phang, Kazim Riaz, Ioannis Sarantitis, S Ali Raza Shehrazi, Paul Turner, Fitzgerald Anazor, Sarah Brown, Radhika Chadha, Emily Crawley, Sofia Farina, Joseph Fennelly, Jiali Gao, Avadhut Kulkarni, Louise Kuo, Mariam Lami, Francesca Lewis, Mark Maher, Gianfranco Messina, Dilip Nair, Gregory Neal‐Smith, Bryony Peiris, Hannah Sellars, Harriet Shearman, Sophie Bird, Umer Chaudhry, Najeed Khan, Harshadkumar Dhirajlal Rajgor, Ajay Belgaumkar, Zain Elahi, Anum Ghani, Prabhat Prakash Narayan, Anu Sandhya, Mostafa Abdelkarim, Faizah Ali, Tanushree Dewan, Nicola Gallagher, Ahmed Hassan, Karishma Khan, Maisoon Matareed, Sreedutt Murali, Bhaven Murji, Ketevan Papidze, Gowtham Sundaram Venkatesan, Lola Tillson‐Hawke, Gireesha Tudawe, Bashar Abdeen, Syed Nayyar Afaque, Saad Bilal Ahmad, Aishah Ahmed, Aoun Ali, Mohamed Amin, Laura Cervini, Saeef Haque, Ken Weixing Ho, Samrat Mukherjee, Shivakumar Shankar, Lilanthi Wickramarachchi, Yu Hsuen Yang, Hafsa Younus, Matthew Hampton, Kiran Madhvani, Majed Al Najjar, Dinesh Alexander, Fayez Almari, Samer Bitar, Ruth Chelva, Daniel Fountain, Abdullah Gabr, Mohamed Galhoum, Abdullah Hanoun, Kenneth Koo, Christina Lipede, Abdul Madni, Musheer Hussain Mohamed, Zaf Naqui, Omar Pathmanaban, Ajay Radhakrishnan, Ashwanth Ramesh, Kohila Sigamoney, Tricia Tay, Richard Unsworth, Graham Branagan, Victoria Morrison‐Jones, Navamayooran Thavanesan, Muzaffar Ahmad Ahmad, Nur Amalina Che Din, Sabeen Majid, Ugam Shah, Yahia Al‐Tamimi, Andrew Bacon, James Catto, Yasin El‐Wajeh, Mairéad Kelly, Ricardo Mohammed‐Ali, Ola Rominiyi, Sanad Saad, Saurabh Sinha, Oliver Tame, Dylan Chew, Martha Fatima Irene De La Cruz Monroy, Anna‐Victoria Giblin, Richard Jackson, Rathan Jeyapalan, John Kiely, Cieran McGrory, James Murray, Jaydip Ray, Daniel Tadross, James Tomlinson, Alex Ward, Yen Nee Jenny Bo, Mohammad Iqbal, Aarti Lakhiani, Guleed Mohamed, William Parry‐Smith, Banchhita Sahu, Aya M Abbas, Naffis Anjarwalla, Jihène El Kafsi, Alasdair Gordon, Samer Jallad, Ehab Kahka, Snigdha Komatineni, Mehran Lashari, Julia Lord, Diana Marujo, Rajesh Midha, Mark Medhat Mikhail, Mafdi Mossaad, Arjun Patel, Tanvir Rafe, Anthony Rayner, Sawsan Saeid, Nilofar Samadi, Hao Meng Yip, Ahmed Abdalla, Raiyyan Aftab, Nathan Curtis, Thomas Dudding, Shoura Karar, Rebekah McCullough, Aaron Julius Punnen, Atiqur Rahman, Simon Williams, Dmitri Artioukh, Aloka Suwanna Danwaththa Liyanage, Krishnan Gokul, Karthikeyan Iyengar, Muyed Mohamed, Khushroo Suraliwala, Bianca Wadham, Chamindri Weerasinghe, Muhammad Aleem, Christopher Brown, Russell Cathcart, Olivia Cohen, Sarfraz Jamali, Miklos Kassai, Thomas Walker, Gbolahan Williams, Rachel Baumber, Rebecca Brinkler, Zara Hayat, Adam Hunt, James Parry, Samuel Adegbola, Govind Dhillon, Barnaby Farquharson, Shareef Mahdi, Tien Yeoh, Clarence Yeoh, Stephen Bromage, Somashree Chatterji, Melissa Condon, Suku George, Smitha George, Claire Hall, Janette Hunt, David Johnson, Vivek Kaushik, Magda Kujawa, Anil Kumar, Muhammad Isfandyar Khan Malik, Fatema Munshi, Sreedevi Nair, Eloka Okoye, Jen Hong Ong, Joanna Rooney, Milan Rudic, Inderpaul Samra, Jamel TaharAissa, Catherine Toksoy, Nimisha Vallabh, Imad Wafaie, Imad Zakieh, Yuhao Zhang, Maimoona Afzal, Erminia Albanese, Daisy Evans, Gomathy Gopal, Anthony Jaipersad, Ben‐Lawrence Kemah, Isaac Kobe, Ayesha Mahmud, Gourab Misra, Bobby Sachdev, Uzma Sadia, Jessica Spalding, Elizabeth Jones, Amy Lovett, Christin Henein, Jia Y Ng, Dimiter Shinkov, Chris Hartley, Amarkumar D Rajgor, Holt Walters, Alasdair Ball, Holly Digne‐Malcolm, Simon Fallis, Mohammed Hamid, Hannah Boyd‐Carson, Marta D’Auria, Manas Dube, Elizabeth Gemmill, Elisa Lenzi, Dimple Sapre, Arjun Shajpal, Wajiha Tehniyat, Nicholas Watson, Ghazia Ahmed, Sankar Ananth, Daniel Ashworth, Peter Cripps, Anna Davies, Dharminder Dhillon, Jennifer Edmondson, Richard Egan, Rhiannon Harries, Alastair Henry, Madhav Kittur, Godfrey Lau, Zoe Li, Miriam Nyeko‐Lacek, Harvey Rich, Ketan Shah, Lydia Tang, Rute Castelhano, Sherwin Ng, Vinay Shah, Paul Stanier, Olivia Wharf, Khine Myat Win, Thomas Badenoch, India Cox, Shanice Cox, Catriona Daly, Kaveh Davoudi, Paul Foster, Christian Grieco, Marianne Hollyman, Louise Hunt, Bhavani Kamalakannan, Andrew Kelly, Hamad Khan, Neil Lenus, Hamish Macdonald, Jo Morrison, Ellen Nelissen, Laura Newitt, Hammad Parwaiz, Emma‐Tina Segall, George Slade, Andrew Stevenson, Charles Geoffrey Dermot Stewart, Samuel Wenham, Amy Whitworth, Rakan Kabariti, Tahir Khaleeq, Pradyumna Naredla, Varkha Rattu, Jae Rhee, Asohanpal Sohanpal, Rosemary Sykes, Cait Bleakley, Joel Bowen, Aubrey Dickason, Ekemini Ekpo, Matthew Sargent, Rupert Scott, Jonathon Sheen, Ellen Thomas, Amrita Virdi, Katherine de Rome, Victoria Russell, Kapil Sahnan, Ahmed Ammar, Ahmed Elsayed, Janahan Sarveswaran, Nimai Desai, Srinivasan Gangadharan, Fahad Hossain, Ghiath Ismayl, Nikhil Khadabadi, Thomas Moores, Mohamed Saleem Noor Mohamed, Shweta Pawar, Aasim Saleemi, Mark Simmons, Sita Techaboonanake, Muhammad Jawad Zafar, Thomas Alexander, Hannah Emerson, Alice Llambias‐Maw, Khaled Abdelgalel, Verda Amin, Ruvinder Athwal, Anuj Bhatnagar, Vasileios Charalampakis, Leslie Chi Yan Cheung, Muha Hassan, Jonathan Lee, Praveena Mahalingam, Sean Ramcharan, Tin Sein, Shahbaz Zafar, Ngozi Anyaugo, Nitya Chandratreya, Jazal El‐Qudah, Venkat Mukund Reddy Galiveeti, Alyazeed Haddadin, Sattam Halaseh, Erfan Massri, Olumide Oyinloye, Ken Philip, Obafemi Wuraola, Aenone Harper Machin, Molly Jakeman, Li Yenn Yong, Timothy Board, Ayman Gabr, Abdulhakim Hassan, Asim Rajpura, Ketan Agarwal, Sanabel Al‐Maghrabi, Jennifer Allan, Joanna Craven, Bryony David, Bala Krishnan, Oliver Lane, Morag McLellan, Simon Powell, Luke Render, Sarah Shamim, Adam Truss, Raman Vinayagam, Ahmed Dhaif, Fraser Maxwell, Fiona Ross, James Blair, Sabri Bleibleh, Govind Singh Chauhan, Varun Dewan, Richard Dias, William Drew, Awais Habeebullah, Lydia Hiddema, Zainab Iftikhar, Jasprit Kaur, Sean Milner, Chinedu Ndegbu, Kishan Rajdev, Jeremy Reid, Nirbhaibir Singh, Osmond Thomas, Raghavan Vidya, Nuha Yassin, Mas’ud Adewusi, Islam Ahmed, Roudi Bachar, Sin Ting Natalie Cheng, Abigail Duckett, Sara El Falaha, Karim Hassan, Tajnin Mitu, Chun Ooi, Elsie Tan, Megan Walker, Sophie Watson, Mohamed Ashiq Mohamed Salim, Malaz Abbakar, Vanessa Badas, Radu Chirvasuta, Joe Drybrough, Alexandra Hamshere, Annabelle Harrison, Rebecca Ireson, Nelson Kamali, Abigail Lau, Sumant Luhana, Luisa MacDonald, Barbara Ribeiro, Alistair Savage, Tanisha Sharma, Paul Swift, Rachel Taute, Alex Wilkins, Stephanie Alford, Chase Elswick, Rakesh Patel, J. Quentin Clemens, John Michael DiBianco, Anna Faris, Mark Hemmila, Giulia I. Lane, Lena Napolitano, Jane J Wong, Shelly Abramowicz, Dina Amin, Steven Roser, Daniel Beswick, Elisa Birnbaum, Khaled Campa, Heather Carmichael, Carolyn Chabuz, Brandon Chapman, Nicole Christian, Marco Del Chiaro, Adam Dyas, Anna Gergen, Anne Getz, Ana Gleisner, Ryan Griffin, Laura Harmon, Scott Hirsch, Victoria Huynh, Juan Pablo Idrovo, Laura Leonard, Helen Madsen, Robert McIntyre, Jr, Robert Meguid, Kenny Rodriguez, Richard Schulick, Christopher Scott, Lauren Steward, Camille Stewart, Malcolm Su, Sarah Tevis, Daniel Thieu, Shane Urban, Catherine Velopulos, Jon Vogel, Thomas Vogler, Kristen Vossler, Michael Weyant, Franklin Wright, Aurora, Erika Bengtson, Mary Bokenkamp, Hannah Chacon, W. Drew Fielder, Alex Haynes, Charlotte Heron, Charles Hill, Cole Holan, Hannah Kay, Brian Kelley, Sabino Lara, Kathryn McElhinney, Kristofor A. Olson, E. Charles Osterberg, Sharmila Paul, Christopher Riley, Ayane Rossano, Romil Shah, Ifeoluwa Shoyombo, Pooja Srikanth, Pedro Teixeira, Marc Trust, Marian Williams‐Brown, Benjamin Bigelow, Eric Etchill, Ijezie Ikwuezunma, Amit Jain, Harsha Malapati, Ivy Mannoh, Adam Margalit, Sarah Rapaport, Srikrishna Vangipuram Suresh, Dominique Vervoort, Lekha Yesantharao, Bo Zhang, Hossam Abdou, Megan Anders, Jose Diaz, Michael Mazzeffi, Peter Rock, Salim Afshar, Andrea Balthazar, Laura Carlson, Karen Carver, Joseph Incorvia, Michael Kurtz, Craig D. McClain, John G. Meara, Meena Nathan, Caleb Nelson, Nathalie Roy, Rachel Saunders, Amir Taghinia, Vivian Williams, Allison Brown, Zara Cooper, Sameer Hirji, Joshua Jolissaint, David Mahvi, Barbara Okafor, Chandrajit Raut, Vanessa Roxo, Ciersten Burks, Matthew Naunheim, Allen Zhou, Frederick Drake, Djanira Fernandes, Maha Haqqani, Nicolette Jabbour, David Novikov, Joseph Sabra, Paul Tornetta, Lauren Tracy, Keianna Vogel, Joanna Wang, Shawn Wang, Shaun Wason, Emily Berner, Kerry Breen, Ander Dorken Gallastegi, Mohamad El Moheb, Anthony Gebran, Marilyn Heng, Haytham Kaafarani, Santiago Lozano Calderon, Michael McTague, Christopher Melnic, Andy Park, Anna Goldenberg‐Sandau, Rebecca Platoff, Lisa Shea, Debra Manning, Romeeka Perkins, Caroline Reinke, Samuel Ross, Kyle Thompson, Grace Chang, Dustin Dehart, Jared Ourieff, Anna Alecci, Rosalinda Alvarado, Anuradha Bhama, Daniel Deziel, Amir Dorafshar, Henry Govekar, Brian Gulack, Dana Hayden, George Kokosis, Andrea Madrigrano, Keith Millikan, Jonathan Myers, Cristina O’Donoghue, Philip Omotosho, Sam Pappas, Srikumar Pillai, Thea Price, Scott Schimpke, Ami Shah, Deana Shenaq, Nicole Siparsky, Alfonso Torquati, Peter Tsambarlis, Srinivas Vourganti, Al‐Faraaz Kassam, Ralph Quillin, Dennis Vaysburg, Mauro Bravo, Elyad Ekrami, Li Kai, Alparslan Turan, Cassie Decker, Elizabeth A Hennessy, Jennifer Rodriquez, Thomas Schroeppel, Zachery Stillman, Gary Schwartz, Emily Shih, Salah Aoun, Vin Shen Ban, James Caruso, Clay Burlew, Julia Coleman, Julie Clark, Kendyll Gartrelle, David Kwon, Kristin Colling, Andrew Barbas, Raquel Bartz, Sarah K. Dotters‐Katz, Tressa Ellett, Etienne Flamant, Hesham Gabr, Dilraj Grewal, George Kasotakis, David Kerr, Vijay Krishnamoorthy, Alexander Lazarides, Justin Ma, Sachin Mehta, Lucy Meyer, Courtney Mitchell, Demetrios Moris, Henry E. Rice, Diego Schaps, Zohal Ghulam‐Jelani, Motasem Refaat, Torre Soderlund, Amna Ali, Airi Katoh, Krista Kaups, Leigh Ann O’Banion, Yazen Qumsiyeh, Jessica Simpkins‐Fresno, Murad Almasri, Gauri Kulkarni, Hisham Marwan, Mohammad Mehdi, Rasaq Olaosebikan, Camilo Velasquez, Rodrigo Arrangoiz, Jeronimo Garcialopez De Llano, Adrian Legaspi, Maria Fernanda Mijares Olivo, Amit Sastry, Vanitha Vasudevan, Hector Garcia‐Chavez, Atif Iqbal, Yesenia Rojas‐Khalil, Malke Asaad, Charles Butler, Cameron Gaskill, Patrick Hensley, Jose Karam, Matthew Katz, Huan Nguyen, Alberto Pieretti, Christina L Roland, Perel Baral, D. David Beahm, German Berbel, Andres Bur, Shea Carver, Austin Findley, Christopher A. Guidry, Megan Hedlund, Corey Hounschell, Sean Kumer, Kelsey E Larson, Derek Marlor, Madhuri Reddy, Scott Turner, Alissa Urich, Alexander Cavalea, Brian Daley, Bailey Humphreys, Rachel Savoy, Mary Leech, Andrew Loehrer, Kari Rosenkranz, Avi Bhavaraju, Nolan Bruce, Lyle Burdine, Emmanouil Giorgakis, Kyle Kalkwarf, Mary Kimbrough, Garrett Klutts, Joseph Margolick, Tamara Osborn, Anna Privratsky, Matthew Roberts, Jennifer Baker, Peyman Benharash, Ronald W. Busuttil, Robert B. Cameron, Maggie DiNome, Joseph DiNorcia, Joseph Hadaya, Farnaz Haji, Lien Hua‐Feng, Catherine Juillard, Minna Lee, Garrett Salzman, Gregory Senofsky, Richard J. Shemin, Jeannie Shen, Carlie Thompson, Areti Tillou, Neal Bhutiani, Elizabeth Bruenderman, Samuel Carson, Clayton Ellis, Farid Kehdy, Robert C.G. Martin, Tyler Mouw, Amy Wise, Linda Britton‐Zier, Julie Dunn, Benjamin Massey, Brittany Bankhead‐Kendall, Chathurika Samudani Dhanasekara, Thomas Wyatt, Daniel Abbott, Taylor Aiken, Thomas Diehl, Eugene Foley, Stephanie Savage, Patrick Schwartz, Syed Nabeel Zafar, Lauren Camp, Ankush Gosain, Ruth A. Lewit, Alessia Cioci, Amber Collier, Gareth Gilna, Neha Goel, Joshua Kronenfeld, Rishi Rattan, Rebecca Saberi, Eva Urrechaga, Juan Figueroa, Basil Karam, Rachel Morris, Christopher J. LaRocca, Archana Ramaswamy, Steven Waisbren, Jacob Ankeny, Rahel Ghebre, Jane Yuet Ching Hui, Eric Jensen, Jordan Mattson, Rachel Vogel, Emily Colonna, Frederick Endorf, Rachel M. Nygaard, Alva Bethurum, Rohan Bhalla, David Bichell, Christopher Bonfield, Silky Chotai, Michael Dewan, Muhammad Owais Abdul Ghani, Alexander Hawkins, Aimal Khan, J. Matthew Kynes, Alexandra Mata, Christopher Wallis, Camila Walters, Alice Wang, Irving Zamora, Lindsey Zamora, Marah Maayah, Nensi Melissa Ruzgar, Melanie Sion, Sarah Ullrich, Dalia Alqunaibit, Kiah Andrews, Danielle Friedman, Anna Liveris, John McNelis, Yasmin Abedin, Brian Batko, Anthony Colon, Sofia Gabrilovich, Nina Glass, Carma Goldstein, Christina Gory, Janmejay Hingu, Brian Leoce, Anh Nguyen, Guergana Panayotova, Ruchi Ram, Deviney Rattigan, Jerette Schultz, Sara Solar, John Stein, Grace Tsui, Elizabeth Warnack, Osaid Alser, Apostolos Gaitanidis, Leon Naar, Charu Paranjape, Robert Sinyard, Sophia Angelides, Theresa Chin, Laura Fitzmaurice, Alexander Himstead, Mehraneh Jafari, Zeljka Jutric, Kari Kansal, Lydia Kirillova, Isabella Kuo, Kapila Patel, Erika Linmey Tay Lasso, Maki Yamamoto, Maleeha Ahmad, Steven Chang, Antonio Meola, Gary Alan Bass, Benjamin Braslow, Daniel Dempsey, Caoimhe Duffy, Daniel Holena, Jonathan Imran, John Keogh, Cara Lorenzi, Niels Martin, Mark Seamon, Lauren Huckaby, Katrina Morgan, Kristina Nicholson, Caroline Rieser, Lauren Rosenblum, Cindy Teng, J. Michael DiMaio, Mohanad Hamandi, Mary Hoffman, Allison T. Lanfear, John Squiers, Michael Connolly, Benjamin Hall, Tareq Kheirbek, Stephanie Lueckel, Elizabeth Renaud, Kevin Behm, Judy Boughey, Kristin Cardiel Nunez, Sean Cleary, Mihai Dumbrava, Travis Grotz, James Jakub, Rahul Kanade, David Larson, Janani Reisenauer, Sherief Shawki, Dennis Wigle, Rachael Callcut, Haradeen Dhillon, Avni Suri, Leonardo Weber Graeff, Ahmed Elshabrawy, Ahmed Mansour, Grace Ihsiu Todd, Mark Davies, Qi Yan, Walter Biffl, Kathryn Schaffer, Sarah Averbach, Allison Berndtson, Terry Curry, Alexandra Schwartz, Mary Kathryn Abel, Veronica Andaya, Kristina Benirschke, Katherine Bigay, Marissa Boeck, Lee‐lynn Chen, Lee‐may Chen, Huey‐Lan Chern, Amanda Compadre, Patricia Conroy, Alexa Glencer, Patrick Ha, Barbara CS Hamilton, Lucy Kornblith, Johannes R Kratz, Rondall Lane, Rex H Lee, Brenda Nunez‐Garcia, Richard O’Donnell, Doruk Ozgediz, Paul Park, Ankit Sarin, Ipsit Shah, Bonnie Sheu, Madhulika Varma, Katherine Wai, Rosanna Wustrack, Mary Jue Xu, Melissa Zimel, Pedro Escobar, Meredid Maldonado Santiago, Mohammad Azam, Asad Choudhry, Eric Hammond, William Marx, Jihad Abbas, Somya Al‐Embideen, Carol Angel, Bailea Bobich, Raul Bosio, Gregory Georgiadis, Tahir Jamil, Maria Kenner, Meghan Lark, Tamara Maghathe, Sonia Masih, Gabriel Naimy, Kristin O’Mara‐Gardner, Emily Rady, Roberta Redfern, Jack Sample, Sara Seegert, Joseph Sferra, Kaitlyn Sharp, Jessica Shoemaker, Stephen Stanek, John Stengle, Hibba Sumra, Jeffrey M. Sutton, Faris Azar, Anne Fischer, Jaymie Henry, Lawrence Lottenberg, Mario Rueda, Sasha Thiel, Adel Bozorgzadeh, John Kelly, Paulo Martins, Armando Salim Munoz Abraham, Erin Scott, Fernando Bonilla Cal, Camila Haro, Andrés Pouy, Gastón Acuña, Sofia álvarez, Ana Carmen Carbajal, Fabiola Castedo, Diego PereiraNuñez, Helena Sobrero, Josefina Tarigo, Rafat Al‐saban, Fatima Al‐Eryani, Siham Al Maqtari, Sahar Al‐maqtari, Mahmmoud Abdualqader, Majed Aeed, Ghadeer Al Sanany, Anwaar Al Dhafif, Buthina Al Jarmozi, Saba Al‐ameri, Awsan AL‐Dhaheri, Hamza Al‐Naggar, Mohammed Al‐Shehari, Dhyia Alhuq Al‐surimi, Amira Alakhdury, Sabreen ALashmali, Zainab Alattas, Manar Alduba’ai, Hamdan Aldumaini, Wafa Alhaddad, Abdelrahman AlHarazi, Nawal Alhemyari, Adnan ALhumaida, Maniee Aljabri, Hassan Almarashi, Zuhoor Almohanady, Marwa ALodine, Nooraldin Alqasemi, Basma Alqudaimi, Ansam Mohammed Ghaleb Alrobaiee, Mohammed Alsabri, Radfan Alsalal, Ramzi Alsayadi, Musaed Alsayadi, Reem Alshaipani, Ebrahim Alsharabi, Nora Atiah, Hadeel Bajjah, Muhib Esmael, Asma Ghallab, Rana Ghannam, Adwa’a Hamid, Lina Hashem, Sara Saif, Shaima Sayad, Nawras Shaban, Amatallah Hadi Shamsan, Sarah Shream, Amal Shukri, Shahd Zeid, Nabila Abdullah, Abdulrahman Faisal Al‐Garadi, Ahmed M. Ali Al‐Radhi, Norhan Naji, Shaima’a Nasser, Areej Taha Al Anaib, Ebrahim Abdurab, Fadhl Al Muhtadi, Amer Al‐hebbah, Ahmed Al‐madhrahi, Shehab Al‐Mahdi, Sakhr Abdulhakeem Al‐maswari, Ayman Al‐oqabi, Ibrahim Al‐Raimi, Zakarya Abdullah Al‐Zaazaai, Karim Al‐Zazay, Ali Alafif, Asem Albzzaz, Lina AlQalisi, Mohammed Alrezami, Haneen Alshargabi, Mokhtar Alshargabi, Hossam Ghanem, Kareem Saeed, Rasheed Shalabi, Fareed Showqi, Miriam Maimbo, Catherine Mkandawire, Mulaya Mubambe, Theresa Nkole, Vanessa Savopoulos, Patricia Shinondo, Chabwera Shumba, Penias Tembo, Shelton Chivanga, Ngqabutho Dube, Handsome Dube, Daniel Dzinotyiwei, Ronald Makanda, Moses Mangena, Partson Maphosa, Derek Matsika, Proud Mawere, Nkosikhona Moyo, Assel Moyo, Arnold Muguwu, Simbarashe Mungazi, Garikai Mwale, Gamuchirai Ndabvonga, Allan Ngulube, Mambote Crispin Olivier Ntoto, Hardlife Ranganai, Ernesto Sánchez Castillo, Oltah Tshuma, Grace Gwini, Vimbai Moyana Muguto, Gilbert Moyo, Trust Mushawarima, Tinashe Shoko, Maphios Siamuchembu, Rorisang Jamela, Marvellous Machiri, Willard Mushiwokufa, Eric Shumba, Antony Chengahomwe, Busisiwe Mlambo, Yemurai Bikwa, Maxwell Chimhina, Simbarashe Chinyowa, Jeremy Marume, Kudzayi Munanzvi, Precious G T Mutambanengwe, GlobalSurg Collaborative

**Affiliations:** ^1^ NIHR Global Health Research Unit on Global Surgery Birmingham UK

**Keywords:** COVID‐19, deep vein thrombosis, pulmonary embolism, SARS‐CoV‐2, venous thromboembolism

## Abstract

SARS‐CoV‐2 has been associated with an increased rate of venous thromboembolism in critically ill patients. Since surgical patients are already at higher risk of venous thromboembolism than general populations, this study aimed to determine if patients with peri‐operative or prior SARS‐CoV‐2 were at further increased risk of venous thromboembolism. We conducted a planned sub‐study and analysis from an international, multicentre, prospective cohort study of elective and emergency patients undergoing surgery during October 2020. Patients from all surgical specialties were included. The primary outcome measure was venous thromboembolism (pulmonary embolism or deep vein thrombosis) within 30 days of surgery. SARS‐CoV‐2 diagnosis was defined as peri‐operative (7 days before to 30 days after surgery); recent (1–6 weeks before surgery); previous (≥7 weeks before surgery); or none. Information on prophylaxis regimens or pre‐operative anti‐coagulation for baseline comorbidities was not available. Postoperative venous thromboembolism rate was 0.5% (666/123,591) in patients without SARS‐CoV‐2; 2.2% (50/2317) in patients with peri‐operative SARS‐CoV‐2; 1.6% (15/953) in patients with recent SARS‐CoV‐2; and 1.0% (11/1148) in patients with previous SARS‐CoV‐2. After adjustment for confounding factors, patients with peri‐operative (adjusted odds ratio 1.5 (95%CI 1.1–2.0)) and recent SARS‐CoV‐2 (1.9 (95%CI 1.2–3.3)) remained at higher risk of venous thromboembolism, with a borderline finding in previous SARS‐CoV‐2 (1.7 (95%CI 0.9–3.0)). Overall, venous thromboembolism was independently associated with 30‐day mortality (5.4 (95%CI 4.3–6.7)). In patients with SARS‐CoV‐2, mortality without venous thromboembolism was 7.4% (319/4342) and with venous thromboembolism was 40.8% (31/76). Patients undergoing surgery with peri‐operative or recent SARS‐CoV‐2 appear to be at increased risk of postoperative venous thromboembolism compared with patients with no history of SARS‐CoV‐2 infection. Optimal venous thromboembolism prophylaxis and treatment are unknown in this cohort of patients, and these data should be interpreted accordingly.

## Introduction

Patients hospitalised with COVID‐19 have a high risk of venous thromboembolism (VTE), with an estimated incidence between 9% and 26% [1, 2, 3, 4, 5] despite pharmacological prophylaxis, and as high as 21–31% in patients within critical care settings [1, 2, 4, 6]. As a result, preliminary mixed guidance has been issued, with some suggesting no change in practice, while others suggesting that increased doses and duration of pharmacological prophylaxis may be beneficial [7, 8]. However, such regimens are associated with serious bleeding risks [9]. Determining the optimal VTE prophylactic regimen for patients with moderate and severe COVID‐19 is an active area of research (e.g. REMAP‐CAP, ACTIV‐4a, ATTACC Investigators, pre‐print, https://doi.org/10.1101/2021.03.10.21252749) [10].

The incidence of VTE in surgical patients infected with SARS‐CoV‐2 is not well known. Most patients undergoing surgery already have risk‐factors for VTE, including immobility, surgical wounds and systematic inflammation. The addition of SARS‐CoV‐2 infection may further increase this risk, but the extent and impact are unknown, and large scale, prospective patient‐level data are lacking. Surgical patients may also carry asymptomatic SARS‐CoV‐2 infections, and whether this contributes to excess risk is also unknown.

Robust evidence is needed to enable clinicians and policymakers to minimise VTE risk in patients with SARS‐CoV‐2 infection. Ideally, such evidence would stratify the risk of VTE against both the duration of time between infection and surgery and presence or absence of symptoms. This study aimed to determine the VTE rate in patients with SARS‐CoV‐2 infection, stratified by current or prior infection.

## Methods

This study was conducted according to guidelines set by the strengthening the reporting of observational studies in epidemiology (STROBE) statement for observational studies [11]. This was a planned sub‐study and analysis from a prospective, international, multicentre cohort study of patients undergoing surgery during October 2020. Data were collected as part of this larger study in the same time frame. This prior study focused on overall 30‐day mortality with specific reporting on pulmonary complications. The methods and findings of this study were published previously [12].

Hospitals providing surgery from any surgical specialty were eligible for participation. Study approvals for participating hospitals were secured in line with local and national regulations before entry into the study. Local investigators were required to confirm that all mandatory approvals were in place before data collection could begin. The study protocol was either registered as a clinical audit with institutional review or a research study obtaining ethical committee approval depending on local and national requirements. Informed patient consent was obtained if this was necessary to comply with local or national regulations. In the UK, this study was registered as a clinical audit in the central co‐ordinating site and registered as either an audit or service evaluation at other recruiting institutions. Therefore, consent was not mandated from individual patients. Data were collected online and stored on a secure server running the Research Electronic Data Capture (REDCap, Vanderbilt University, Nashville, TN, USA) web application [13], based at the University of Birmingham, UK. Hospitals registered their interest to participate based on one or more surgical specialties. Participating specialties then collected data on consecutive patients who underwent surgery within their department during one or more pre‐selected weeks between 5 October and 1 November 2020, with a 30‐day postoperative follow‐up period. No changes were made to local patient care protocols during the course of this study. Only anonymised, routine clinical data were collected.

Adult patients, aged 18 y and over, undergoing elective or emergency surgery for any indication, from any specialty, were eligible. As VTE events are very rare in patients aged <18 y, these patients were not included from this current analysis. Surgery was defined as any procedure routinely performed in an operating theatre by a surgeon. A list of excluded procedures can be found in online Supporting Information Table S1.

Baseline patient characteristics included age, ASA physical status and smoking status. Age was collected as a categorical variable in deciles of years and categorised into three groups for analysis: 18–49 y; 50–69 y; and ≥70 y. The ASA physical status was dichotomised to 1–2 or 3–5. Patients were identified as smokers if they were current smokers or had smoked in the six weeks before surgery. Data collected on pre‐existing medical conditions included respiratory comorbidities (asthma; chronic obstructive pulmonary disease); congestive cardiac failure; cerebrovascular disease; chronic kidney disease; and ischaemic heart disease. Indications for surgery were classified as: benign disease; cancer; obstetrics; or trauma. Operative variables included urgency of surgery (elective or emergency); type of anaesthesia (local/regional or general); and grade of surgery (minor or major). National income was based on the World Bank's classification for each participating country [14].

A positive SARS‐CoV‐2 diagnosis was based on a patient having one or more of the following: a positive reverse transcriptase‐polymerase chain reaction (RT‐PCR) nasopharyngeal swab; a positive rapid antigen test; chest computed tomography (CT) scan showing changes in keeping with locally implemented protocols that indicate SARS‐CoV‐2 infection; positive immunoglobulin G or immunoglobulin M antibody test; or clinical diagnosis of symptoms in keeping with COVID‐19 in patients where no swab test or CT scan were available. Timing of diagnosis of SARS‐CoV‐2 in relation to the day of surgery was collected as a categorical variable and further collapsed into one of the following groups for analysis: no SARS‐CoV‐2; peri‐operative SARS‐CoV‐2 (diagnosed 7 days before to 30 days after surgery); recent SARS‐CoV‐2 (diagnosed 1–6 weeks before surgery); or previous SARS‐CoV‐2 (diagnosed ≥7 weeks before surgery). Data were also collected on the presence or absence of respiratory or non‐respiratory SARS‐CoV‐2 symptoms if patients had a pre‐operative SARS‐CoV‐2 diagnosis. These were analysed as a combined group of patients with asymptomatic infection or those with previous symptoms now resolved, or patients with ongoing symptoms. Symptoms in patients with a postoperative SARS‐CoV‐2 diagnosis were not analysed as it was not possible to separate these from standard postoperative symptoms.

The primary outcome measure was VTE within 30 days following surgery. Venous thromboembolism was defined as either deep vein thrombosis (DVT) or pulmonary embolism (PE). Deep vein thrombosis was defined as lower limb DVT with or without symptoms that was proven radiologically; and PE was defined as symptomatic PE, radiologically proven or fatal PE discovered at post‐mortem or as judged by the clinical team. Secondary outcome measures were postoperative pneumonia and mortality within 30 days of surgery. Full study definitions can be found in online Supporting Information Appendix S2.

Patients with data missing on VTE or SARS‐CoV‐2 status and patients aged <18 y were excluded in the analysis. The Chi‐square test of independence was used to compare groups in terms of categorical data. For the primary outcome of VTE within 30 days of surgery, multivariable logistic regression analysis was used to evaluate the association of SARS‐CoV‐2 infection and VTE after surgery, which was summarised using OR (95%CI). The model included clinically relevant patient and operative factors in order to adjust for covariates and reduce the risk of confounding (baseline patient characteristics; pre‐existing comorbidities; operative factors). Multivariable adjusted sub‐group analyses were performed based on the main analysis to define the patients in which SARS‐CoV‐2 infection was associated with additional risk of postoperative VTE above the expected baseline risk of that sub‐group. This was done in the following four sub‐groups: major surgery; minor surgery; elective surgery; and emergency surgery. A multivariable adjusted analysis was also fitted for 30‐day mortality as the outcome with VTE as the main explanatory variable, using clinically relevant patient and operative factors for adjustment. Analyses were performed with Stata SE version 16.1, (StataCorp, TX, USA).

## Results

**Figure 1:**
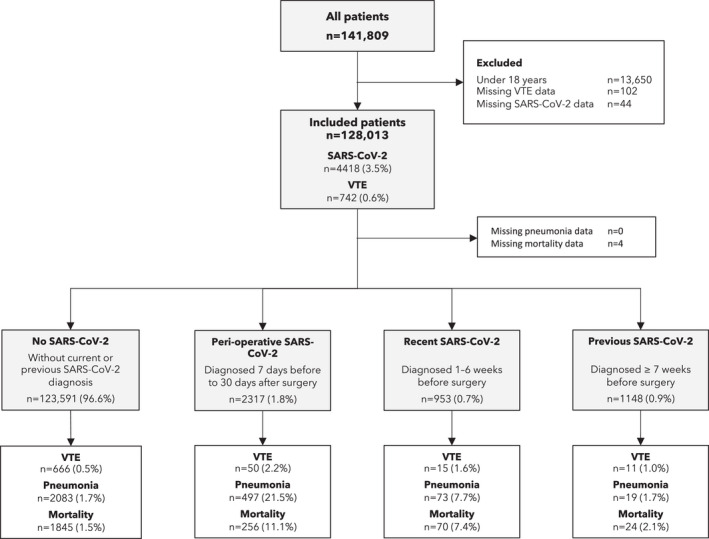
Flow diagram of patients showing venous thromboembolism (VTE), pneumonia, mortality and SARS‐CoV‐2 infection.

This analysis included 128,013 patients, from 1630 hospitals across 115 countries. Baseline patient and operative characteristics are shown in Table 1. Of these patients, 59,182 (46.2%) were men; 94,022 (73.5%) were ASA physical status 1–2 and 20,433 (16.0%) were smokers. The total number of patients who had a diagnosis of SARS‐CoV‐2 infection was 4418 (3.5%). The timing of SARS‐CoV‐2 diagnosis in relation to the day of surgery was peri‐operative (7 days before to 30 days after surgery) for 2317 patients (1.8%); recent (1–6 weeks before surgery) for 953 patients (0.7%); and previous (≥7 weeks before surgery) for 1148 patients (0.9%; Fig. 1). Postoperative pneumonia was chosen as the best fitting variable to represent more severe SARS‐CoV‐2 infection. Proportionally, pneumonia occurred most frequently in patients with peri‐operative SARS‐CoV‐2 (497 patients, 21.5%), followed by recent SARS‐CoV‐2 patients (73, 7.7%). Patients with previous SARS‐CoV‐2 (19, 1.7%) and no SARS‐CoV‐2 diagnosis (2083, 1.7%) had the same risk for postoperative pneumonia (Fig. 1). Compared with patients who did not have SARS‐CoV‐2 infection, patients with peri‐operative SARS‐CoV‐2 were older (28.2% vs. 21.3%, p < 0.001), ASA physical status 3–5 (39.6% vs. 26.2%, p < 0.001), underwent emergency surgery more often (58.4% vs. 29.5%, p < 0.001) and had a greater comorbid burden (Table 1). This trend was also present when comparing patients without SARS‐CoV‐2 and those with recent and previous infection (Table 1).

**Table 1:** Baseline patient, disease and operative characteristics stratified by SARS‐CoV‐2 status. Values are number (proportion).

	No SARS‐CoV‐2	Peri‐operative SARS‐CoV‐2	Recent SARS‐CoV‐2	Previous SARS‐CoV‐2	p value
	n = 123,595	n = 2317	n = 953	n = 1148	
Age; y					
18–49	55,651 (45.0%)	968 (41.8%)	461 (48.4%)	480 (41.8%)	<0.001
50–69	41,633 (33.7%)	696 (30.0%)	332 (34.8%)	470 (40.9%)	
≥70	26,309 (21.3%)	653 (28.2%)	160 (16.8%)	198 (17.3%)	
Missing	2	0	0	0	
Sex					
Female	66,495 (53.8%)	1228 (53.0%)	493 (51.7%)	611 (53.2%)	0.506
Male	57,096 (46.2%)	1089 (47.0%)	460 (48.3%)	537 (46.8%)	
Missing	4	0	0	0	
ASA physical status					
1–2	91,229 (73.8%)	1399 (60.4%)	635 (66.6%)	759 (66.1%)	<0.001
3–5	32,323 (26.2%)	918 (39.6%)	318 (33.4%)	389 (33.9%)	
Missing	43	0	0	0	
Smoking					
No	103,387 (83.9%)	1949 (84.3%)	845 (88.9%)	1017 (88.8%)	<0.001
Yes	19,835 (16.1%)	364 (15.7%)	106 (11.1%)	128 (11.2%)	
Missing	373	4	2	3	
Respiratory comorbidity					
No	111,785 (90.5%)	2026 (87.5%)	854 (89.6%)	1027 (89.5%)	<0.001
Yes	11,713 (9.5%)	290 (12.5%)	99 (10.4%)	121 (10.5%)	
Missing	97	1	0	0	
Congestive heart failure					
No	118,829 (96.2%)	2151 (92.8%)	907 (95.2%)	1079 (94.0%)	<0.001
Yes	4738 (3.8%)	166 (7.2%)	46 (4.8%)	69 (6.0%)	
Missing	28	0	0	0	
Cerebral vascular disease					
No	119,253 (96.5%)	2190 (94.5%)	922 (96.7%)	1103 (96.1%)	<0.001
Yes	4314 (3.5%)	127 (5.5%)	31 (3.3%)	45 (3.9%)	
Missing	28	0	0	0	
Chronic kidney disease					
No	120,475 (97.5%)	2179 (94.0%)	900 (94.4%)	1094 (95.3%)	<0.001
Yes	3092 (2.5%)	138 (6.0%)	53 (5.6%)	54 (4.7%)	
Missing	28	0	0	0	
Ischaemic heart disease					
No	112,894 (91.4%)	1995 (86.1%)	856 (89.8%)	1037 (90.3%)	<0.001
Yes	10,673 (8.6%)	322 (13.9%)	97 (10.2%)	111 (9.7%)	
Missing	28	0	0	0	
Indication					
Benign disease	76,169 (61.6%)	1215 (52.4%)	561 (58.9%)	777 (67.7%)	<0.001
Malignancy	23,251 (18.8%)	421 (18.2%)	211 (22.1%)	231 (20.1%)	
Trauma	14,595 (11.8%)	436 (18.8%)	114 (12.0%)	91 (7.9%)	
Obstetric	9577 (7.8%)	245 (10.6%)	67 (7.0%)	49 (4.3%)	
Missing	3	0	0	0	
Grade of surgery					
Minor	47,178 (38.2%)	695 (30.0%)	285 (29.9%)	439 (38.2%)	<0.001
Major	76,392 (61.8%)	1622 (70.0%)	668 (70.1%)	709 (61.8%)	
Missing	25	0	0	0	
Urgency of surgery					
Elective	87,117 (70.5%)	965 (41.6%)	604 (63.4%)	857 (74.7%)	<0.001
Emergency	36,471 (29.5%)	1352 (58.4%)	349 (36.6%)	291 (25.3%)	
Missing	7	0	0	0	
Anaesthesia					
Local/regional	34,508 (27.9%)	707 (30.5%)	222 (23.3%)	285 (24.8%)	<0.001
General	89,035 (72.1%)	1609 (69.5%)	731 (76.7%)	863 (75.2%)	
Missing	52	1	0	0	
Country income					
High	20,624 (66.9%)	399 (55.3%)	242 (38.4%)	121 (58.8%)	<0.001
Upper middle	20,238 (16.4%)	636 (27.4%)	345 (36.2%)	352 (30.7%)	
Low middle/low	82,733 (16.7%)	1282 (17.2%)	366 (25.4%)	675 (10.5%)	
Missing	0	0	0	0	

**Figure 2:**
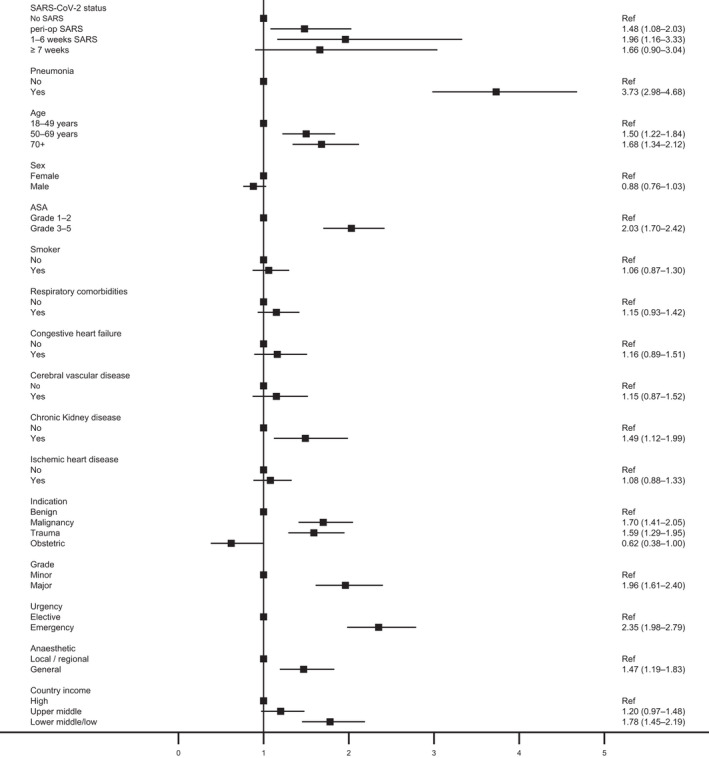
Forest plot of adjusted regression model for factors associated with venous thromboembolism. Figures show the reference value (Ref) and OR (95%CI) for the levels of each variable.

Overall, the rate of postoperative VTE was 0.6% (742/128,013). Of these 742 patients, 44.3% (329) had a PE only, 47.5% (352) had a DVT only and 8.2% (61) had both. A full description of the VTE event rates in relation to patient and operative characteristics are reported in Table 2 and details of PE and DVT by specialty can be found in online Supporting Information Table S2. In adjusted analyses, significant predictors of postoperative VTE were peri‐operative and recent SARS‐CoV‐2 infection; pneumonia; age >50 y; ASA physical status 3–5; chronic kidney disease; surgery for malignancy or trauma; major surgery; emergency surgery; having a general anaesthetic; and surgery performed in a country of upper‐middle, low‐middle or low income (Fig. 2, detailed in online Supporting Information Table S3). Pneumonia was strongly associated with postoperative VTE, and obstetric procedures had a lower rate of VTE when compared with benign (non‐obstetric, non‐cancer) surgery (Fig. 2).

**Table 2:** Unadjusted venous thromboembolism (VTE) rates by patient, disease and operative factors. Values are number (proportion).

	No VTE n = 127,270	VTE n = 742	p value
Age; y			
18–49	57,368 (45.1%)	192 (25.9%)	<0.001
50–69	42,852 (33.7%)	279 (37.6%)	
≥70	27,049 (21.3%)	271 (36.5%)	
Missing	2	0	
Sex			
Female	68,447 (53.8%)	380 (51.2%)	0.161
Male	58,820 (46.2%)	362 (48.8%)	
Missing	4	0	
ASA physical status			
1–2	93,699 (73.7%)	323 (43.5%)	<0.001
3–5	33,529 (26.3%)	419 (56.5%)	
Missing	43	0	
Smoking			
No	106,582 (84.0%)	616 (83.2%)	0.578
Yes	20,309 (16.0%)	124 (16.8%)	
Missing	380	2	
Respiratory comorbidity			
No	115,067 (90.5%)	625 (84.2%)	<0.001
Yes	12,106 (9.5%)	117 (15.8%)	
Missing	98		
Congestive heart failure			
No	122,296 (96.1%)	670 (90.3%)	<0.001
Yes	4947 (3.9%)	72 (9.7%)	
Missing	28	0	
Cerebral vascular disease			
No	122,786 (96.5%)	682 (91.9%)	<0.001
Yes	4457 (3.5%)	60 (8.1%)	
Missing	28	0	
Chronic kidney disease			
No	123,962 (97.4%)	686 (92.5%)	<0.001
Yes	3281 (2.6%)	56 (7.5%)	
Missing	28	0	
Ischaemic heart disease			
No	116,174 (91.3%)	608 (81.9%)	< 0.001
Yes	11069 (8.7%)	134 (18.1%)	
Missing	28		
Indication			
Benign disease	78,373 (61.6%)	349 (47.0%)	<0.001
Malignancy	23,915 (18.8%)	199 (26.8%)	
Trauma	15,066 (11.8%)	170 (22.9%)	
Obstetric	9914 (7.8%)	24 (3.2%)	
Missing	3		
Grade of surgery			
Minor	48,465 (38.1%)	132 (17.8%)	<0.001
Major	78,781 (61.9%)	610 (82.2%)	
Missing	25		
Urgency of surgery			
Elective	89,192 (70.1%)	351 (47.3%)	<0.001
Emergency	38072 (29.9%)	391 (52.7%)	
Missing	7		
Anaesthesia			
Regional/local	35,597 (28.0%)	125 (16.8%)	<0.001
General	91,650 (72.0%)	617 (83.2%)	
Missing	53		
Country income			
High	84,572 (66.4%)	484 (65.2%)	0.268
Upper middle	21,453 (16.9%)	118 (15.9%)	
Low middle/low	21,246 (16.7%)	140 (18.9%)	
Missing	0		

**Figure 3:**
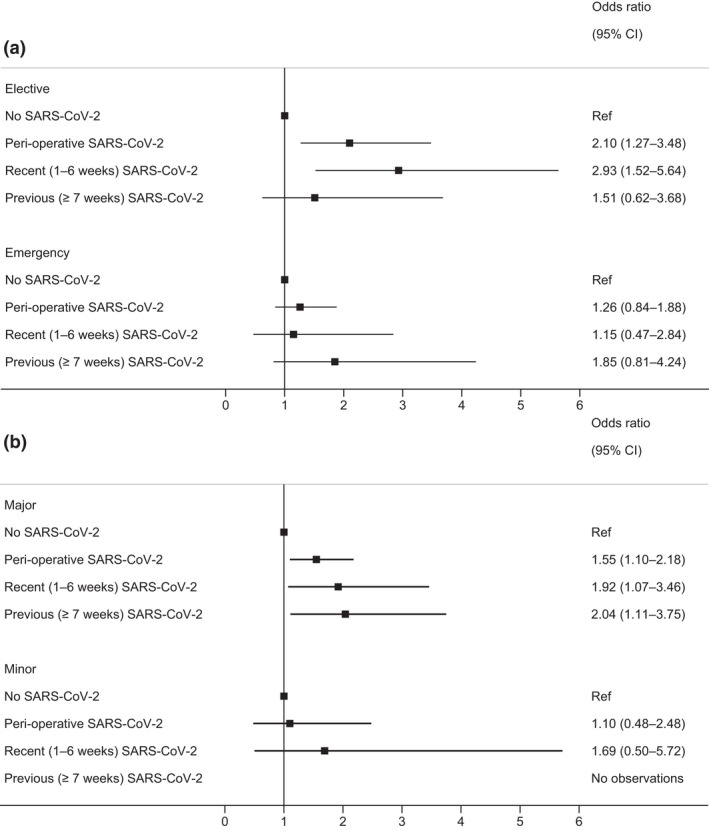
Adjusted sub‐group analysis for venous thromboembolism in (A) elective and emergency patients and (B) major and minor surgery patients. Sub‐group analyses are adjusted for the following variables: pneumonia; age; sex; ASA physical status; smoker; respiratory comorbidities; congestive heart failure; cerebral vascular disease; chronic kidney disease; ischaemic heart disease; indication; grade; urgency; anaesthesia; and country income. Full details can be found in online Supporting Information Tables S4 to S7. Figures show the reference value (Ref) and OR (95%CI) for the levels of each variable.

When compared against the main adjusted analysis of all patients (Fig. 2), sub‐group analysis of elective surgery patients only demonstrated a stronger association between peri‐operative and recent SARS‐CoV‐2 infection and VTE; this effect was diminished in patients having emergency surgery (Fig. 3). Sub‐group analysis in major surgery demonstrated similar risk of postoperative VTE to the main analysis in patients with peri‐operative, recent or previous SARS‐CoV‐2 infection, although there was no significant effect in patients who had minor surgery (Fig. 3).

**Figure 4:**
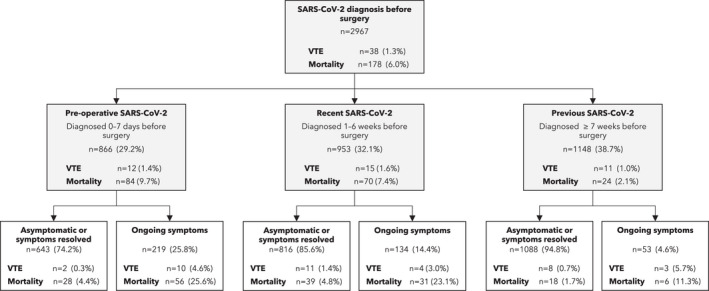
Venous thromboembolism (VTE) and 30‐day mortality in patients with pre‐operative SARS‐CoV‐2 by presence or absence of COVID‐19 symptoms.

In patients with pre‐operative SARS‐CoV‐2 infection, the presence of ongoing SARS‐CoV‐2 symptoms was associated with increased incidence of VTE when compared with patients without ongoing symptoms (Fig. 4). Ongoing symptoms were associated with an overall 4.6% (17/406) rate of postoperative VTE vs. 0.8% (21/2547) in patients who were asymptomatic or whose symptoms had resolved. This effect persisted even after stratifying patients by timing of SARS‐CoV‐2 diagnosis (Fig. 4) and was observed even in symptomatic patients with a SARS‐CoV‐2 diagnosis ≥7 weeks before surgery (5.7% in symptomatic patients vs. 0.7% in asymptomatic or resolved patients).

Overall, the rate of 30‐day postoperative mortality was 1.7% (2195/128,009). When this was stratified by SARS‐CoV‐2 infection and postoperative VTE, unadjusted analyses demonstrated an incremental increase in mortality rates with SARS‐CoV‐2 infection and VTE (Table 3). In adjusted analyses, VTE was independently and strongly associated (OR 5.4 (95%CI 4.4–6.8)) with 30‐day mortality (Table 4).

**Table 3:** Rate of 30‐day mortality stratified by SARS‐CoV‐2 and venous thromboembolism (VTE) status. Values are fraction (proportion)

	No VTE		VTE		p value
All patients	2047/127,271	1.6%	148/742	20.0%	< 0.001
No SARS‐CoV‐2 infection	1728/122,929	1.4%	117/666	17.6%	< 0.001
Any SARS‐CoV‐2 infection	319/4342	7.4%	31/76	40.8%	< 0.001
Peri‐operative SARS‐CoV‐2	236/2267	10.4%	20/50	40.0%	< 0.001
Recent SARS‐CoV‐2	60/938	6.4%	10/15	66.7%	< 0.001
Previous SARS‐CoV‐2	23/1137	2.0%	1/11	9.1%	0.103

**Table 4:** Adjusted regression model for predictors for 30‐day mortality. Values are fraction (proportion) or OR (95%CI)

	Mortality	OR (95%CI)	p value
VTE status			
No VTE	2047/127,267 (1.6%)		
VTE	594/742 (20.0%)	5.42 (4.36–6.75)	<0.001
SARS‐CoV‐2 status			
No SARS‐CoV‐2	1845/123,591 (1.5%)		
Peri‐operative SARS‐CoV‐2	256/2317 (11.1%)	2.38 (2.00–2.82)	<0.001
Recent SARS‐CoV‐2	70/953 (7.4%)	2.78 (2.09–3.71)	<0.001
Previous SARS‐CoV‐2	24/1148 (2.1%)	1.13 (0.72–1.76)	0.597
Pneumonia			
No	1666/125,337 (1.3%)		
Yes	529/2672 (19.8%)	5.28 (4.65–6.00)	<0.001
Age; y			
18–49	429/57,557 (0.8%)		
50–69	757/43,130 (1.8%)	1.62 (1.41–1.86)	<0.001
≥70	1009/27,320 (3.7%)	2.67 (2.31–3.10)	<0.001
Sex			
Female	956/68,825 (1.4%)		
Male	1239/59,180 (2.1%)	1.06 (0.96–1.16)	0.269
ASA physical status			
1–2	543/94,020 (0.6%)		
3–5	1651/33,946 (4.9%)	4.32 (3.84–4.86)	<0.001
Smoking			
No	1796/105,399 (1.7%)		
Yes	387/20,045 (1.9%)	1.16 (1.02–1.32)	0.021
Respiratory comorbidities			
No	1843/115,688 (1.6%)		
Yes	349/12,223 (2.9%)	0.95 (0.83–1.08)	0.410
Congestive heart failure			
No	1859/122,962 (1.5%)		
Yes	336/5019 (6.7%)	1.54 (1.34–1.78)	< 0.001
Cerebral vascular disease			
No	1940/123,465 (1.6%)		
Yes	255/4516 (5.7%)	1.37 (1.18–1.60)	< 0.001
Chronic kidney disease			
No	1884/124,644 (1.5%)		
Yes	311/3337 (9.3%)	2.32 (2.00–2.69)	<0.001
Ischaemic heart disease			
No	1677/116,778 (1.4%)		
Yes	518/11,203 (4.6%)	1.01 (0.90–1.14)	0.842
Indication			
Benign	1174/78,720 (1.5%)		
Malignancy	569/24,112 (2.4%)	1.90 (1.68–2.14)	<0.001
Trauma	413/15,236 (2.7%)	0.91 (0.80–1.04)	0.160
Obstetric	39/9938 (0.4%)	0.39 (0.27–0.55)	<0.001
Grade of surgery			
Minor	388/48,596 (0.8%)		
Major	1807/79,388 (2.3%)	1.80 (1.59–2.03)	<0.001
Urgency of surgery			
Elective	666/89,540 (0.7%)		
Emergency	1535/38,462 (4.0%)	5.62 (5.03–6.27)	<0.001
Anaesthetic			
Local/regional	306/35,721 (0.9%)		
General	1888/92,235 (2.1%)	1.90 (1.65–2.18)	<0.001
Country income			
High	1196/85,055 (1.4%)		
Upper middle	463/21,569 (2.2%)	2.43 (2.15–2.75)	<0.001
Lower middle/low	536/21,385 (2.5%)	4.73 (4.17–5.37)	<0.001

VTE, venous thromboembolism.

Peri‐operative SARS‐CoV‐2, 7 days before to 30 days after surgery; recent SARS‐CoV‐2, 1–6 weeks before surgery; previous SARS‐CoV‐2, ≥ 7 weeks before surgery.

## Discussion

This planned sub‐study found that SARS‐CoV‐2 infection was independently associated with an increased incidence of postoperative VTE in patients with peri‐operative and recent SARS‐CoV‐2 infection. In patients with pre‐operative SARS‐CoV‐2, ongoing symptoms were associated with an increased rate of postoperative VTE, irrespective of how long before surgery the diagnosis was made. Pneumonia was strongly associated with postoperative VTE, possibly due to a combination of SARS‐CoV‐2‐induced pneumonitis and a more difficult postoperative period involving infection, increased disease burden and greater immobility. Mortality was highest in patients with SARS‐CoV‐2 infection and VTE, and in adjusted analyses, SARS‐CoV‐2 and VTE were both independently associated with 30‐day mortality. However, these results were limited by a lack of information on pre‐operative anticoagulant use and postoperative VTE prophylactic regimens.

Overall, emergency surgery patients have a higher rate of postoperative VTE. However, sub‐group analysis demonstrated that in elective patients, there was a greater additional VTE risk in patients with SARS‐CoV‐2 when compared with patients with no SARS‐CoV‐2. This additional risk was not as pronounced in emergency surgery patients. Without data on any differences in the VTE prophylaxis and anticoagulation regimes between the elective and emergency surgery patients, no firm conclusions can be drawn. However, it is possible that this could be a consequence of the greater number of VTE risk‐factors associated with emergency surgery, resulting in any additional risk from SARS‐CoV‐2 infection having less of an impact on the overall risk of postoperative VTE. This translates into a disproportionately increased risk in patients undergoing elective surgery. Sub‐group analysis also demonstrated greater additional risk in patients undergoing major surgery. This is most likely due to the smaller proportion of patients with peri‐operative SARS‐CoV‐2 infection having minor elective surgery during the ongoing pandemic. Overall, minor surgery patients were exposed to fewer of the risk‐factors for VTE, and baseline VTE rate was low. Concurrent SARS‐CoV‐2 infection was not associated with a significant increase in additional risk.

There have been numerous studies describing the elevated rates of VTE in medical patients who are hospitalised with COVID‐19 on the ward or in the ICU, and results are pending for a number of ongoing randomised controlled trials investigating VTE prophylaxis and therapeutic protocols. To date, some studies describe increased bleeding risk with therapeutic (high) dosing of pharmacological VTE prophylaxis [10], and interim analyses of several randomised controlled trials report unfavourable outcomes with therapeutic pharmacological prophylaxis in patients with severe COVID‐19 (i.e. admitted to ICU). However, improved outcomes and a reduced requirement for organ support has been seen in patients with moderate severity of COVID‐19 (i.e. hospitalised) who receive therapeutic anticoagulation [15]. This highlights the challenge of anticoagulating patients with COVID‐19, which is likely to be even more complex in patients having surgery, although our results suggest that anticoagulation in patients with previous, recent or peri‐operative SARS‐CoV‐2 infection may be an important consideration.

Surgical patients represent a uniquely different cohort. Unlike medical patients, the primary reason for hospital admission for surgical patients is rarely due to COVID‐19, and other co‐existing primary pathology requiring surgical intervention is also present. Surgical patients undergo an operative procedure which artificially produces a wound that increases the risk of intra‐operative and postoperative bleeding and sets in motion a cascade of inflammatory responses known to alter haemodynamics and coagulation. Paired with this, surgical patients often experience a period of reduced mobility immediately before, during and after their operation, even for the young and normally fit and healthy. Furthermore, elective surgical patients are a group that can have a planned hospital admission, often following a period of self‐isolation with reduced mobility, and many of these patients will have peri‐operative mechanical ventilation. These differences in patient physiology and exposure signify a need to define VTE risk specifically in surgical patients, not only to provide a baseline understanding of peri‐operative risk in the setting of COVID‐19, but also to work towards constructing future VTE regimens specifically suited to surgical patients with active or prior SARS‐CoV‐2 infection.

This study has several limitations. First, information on postoperative VTE prophylaxis regimens (mechanical and pharmacological) and pre‐existing anticoagulation for specific patient comorbidities associated with prophylaxis (e.g. atrial fibrillation) were not collected as part of this study. During the period of study (October 2020), patients with known SARS‐CoV‐2 infection might have already empirically received enhanced VTE prophylaxis based on earlier reports associating COVID‐19 and an increased risk of VTE. The present study data report outcomes from VTE care and prophylaxis which were deemed acceptable and appropriate for each individual patient in participating departments from each country at the time of the study. Any additional risk could be interpreted as risk above prevailing VTE protocols and practice. Second, this study did not include patients who had an asymptomatic VTE diagnosed as a result of screening. Though there are reports of VTE screening being carried out in high‐risk patient groups, the clinical relevance of asymptomatic, distal DVT is uncertain and could lead to overdiagnosis and skewed results [1]. Venous thromboembolism diagnoses made in this study were likely due to symptomatic presentation or a high index of clinical suspicion leading to radiological confirmation, and we believe that the incidence of VTE in this study is representative of the true, clinically relevant figure. Third, the rate of SARS‐CoV‐2 infection in October of 2020 and the overall incidence of VTE in surgical patients were both relatively low. Despite the large number of patients in this study, some sub‐group analyses have resulted in small patient samples, and these should be interpreted with caution. Finally, there exists the possibility that some patients who had SARS‐CoV‐2 infection never attained a formal diagnosis and were therefore classified as no SARS‐CoV‐2. This most likely occurred in patients with asymptomatic infection. This study reported a high proportion of patients with asymptomatic infection which provides some reassurance that these cases were appropriately counted. While asymptomatic patients could have been misclassified as no SARS‐CoV‐2, this misclassification would have resulted in an underestimation of the overall difference in VTE between groups, and our estimate could be considered conservative.

Despite this study’s limitations, recent and peri‐operative SARS‐CoV‐2 infection may be an independent risk‐factor for postoperative VTE, and increased awareness and surveillance should be considered. At a minimum, we suggest close adherence to routine standard VTE prophylaxis for surgical patients, including the use of pharmacological agents when bleeding risk is minimal, and increased vigilance with a heightened index of suspicion and a lower threshold for definitive diagnostic testing in patients presenting with signs of VTE. Routine postoperative care of surgical patients should include interventions to reduce VTE risk in general, and further research is needed to define the optimal protocols for VTE prophylaxis and treatment for surgical patients in the setting of SARS‐CoV‐2 infection.

## Supporting information


**Appendix S1.** COVIDSurg Collaborative authors (all PubMed indexed co‐authors).

**Appendix S2.** Definitions of terms used in this study.

**Tables S1.** List of excluded procedures.
**Tables S2.** Ranked VTE rates alongside PE and DVT rates by specialty in all patients of any SARS‐CoV‐2 status
**Tables S3.** Adjusted regression model for factors associated with venous thromboembolism (shown in Figure 3).
**Tables S4.** Adjusted sub‐group analysis for VTE in elective patients only.
**Tables S5.** Adjusted sub‐group analysis for VTE in emergency patients only.
**Tables S6.** Adjusted sub‐group analysis for VTE in major surgery patients only.
**Tables S7.** Adjusted sub‐group analysis for VTE in minor surgery patients only.
