# Supplementary material for: SARS‐CoV‐2 infection and venous thromboembolism after surgery: an international prospective cohort study
Source: Anaesthesia. 2021 Aug 24;77(1):28–39. doi: 10.1111/anae.15563 (PMC8652887; doi:10.1111/anae.15563)
Supplement: Supplementary file 2 — Appendix S2. Definitions of terms used in this study. [file ANAE-77-28-s002.docx]

**Appendix S2.** Definitions of terms used in this study.

***Grade of surgery***

Grade of surgery was categorised on the basis of the Bupa schedule of procedures as either minor (minor or intermediate according to the Bupa schedule) or major (major or complex major according to the Bupa schedule).

***Postoperative pneumonia***

The US Centers for Disease Control (CDC) definition of pneumonia will be used, modified to

accommodate limited availability of radiological facilities at some participating centres:

At least one of the following:

• Fever (>38°C) with no other recognised cause.

• Leucopaenia (white cell count <4x109) or leucocytosis (white cell count >12x109).

• For adults >70 years old, altered mental status with no other recognised cause.

AND at least two of the following:

• New onset of purulent sputum or change in character of sputum, or increased respiratory secretions, or increased suctioning requirements.

• New onset or worsening cough, or dyspnoea, or tachypnoea.

• Rales, crackles or bronchial breath sounds.

• Worsening gas exchange (hypoxaemia, increased oxygen requirement).

Wherever possible, the diagnosis should be confirmed with a chest radiograph. The following findings confirm pneumonia:

• New or progressive and persistent infiltrates.

• Consolidation.

• Cavitation.

***Deep vein thrombosis***

Deep vein thrombosis (DVT) is defined as lower limb deep vein thrombosis with or without symptoms, proven by:

• Lower extremity ultrasonography revealing non-compressibility at the trifurcation of the popliteal vein or above, or

• Computed tomography (CT) venography demonstrating a constant intraluminal filling defect above the trifurcation of the popliteal vein.

***Pulmonary embolism (PE)***

Pulmonary embolism (PE)3-4 is defined as:

• Symptomatic PE confirmed by imaging (computed tomography pulmonary angiogram (CTPA) demonstrating new intraluminal filling defect in a subsegmental or greater sized pulmonary artery; or

• Ventilation/perfusion scanning with a high probability of PE; or pulmonary angiograph

demonstrating PE), or

• Fatal PE discovered at autopsy or as judged by the clinical team.
