# Supplementary material for: SARS‐CoV‐2 infection and venous thromboembolism after surgery: an international prospective cohort study
Source: Anaesthesia. 2021 Aug 24;77(1):28–39. doi: 10.1111/anae.15563 (PMC8652887; doi:10.1111/anae.15563)
Supplement: Supplementary file 3 — Tables S1. List of excluded procedures. Tables S2. Ranked VTE rates alongside PE and DVT rates by specialty in all patients of any SARS‐CoV‐2 status Tables S3. Adjusted regression model for factors associated with venous thromboembolism (shown in Figure 3). Tables S4. Adjusted sub‐group analysis for VTE in elective patients only. Tables S5. Adjusted sub‐group analysis for VTE in emergency patients only. Tables S6. Adjusted sub‐group analysis for VTE in major surgery patients only. Tables S7. Adjusted sub‐group analysis for VTE in minor surgery patients only. [file ANAE-77-28-s003.docx]

**Table S1.** List of excluded procedures.

| **Specialty** | **Excluded procedures** |
| --- | --- |
| Abdominal surgery | Ascitic drain (drainage of peritoneal cavity) |
|  | Endoscopic ultrasound |
|  | Laparoscopic ultrasound |
| Breast surgery | Breast biopsy |
| Cardiac surgery | Insertion of cardiac pacemaker |
|  | Percutaneous coronary intervention |
|  | Transluminal balloon angioplasty of coronary artery |
| Colorectal surgery | Colonoscopy (diagnostic or therapeutic) |
|  | Flexible sigmoidoscopy (diagnostic or therapeutic) |
|  | Proctoscopy (diagnostic or therapeutic) |
| Dental procedures | Implantation of tooth |
|  | Insertion of dental prosthesis |
|  | Orthodontic operations |
|  | Restoration of tooth |
|  | Extraction of tooth |
| Gynaecology | Cervical biopsy |
|  | Colposcopy (diagnostic or therapeutic) |
| Obstetrics | Any vaginal delivery (normal delivery, breech delivery, forceps delivery, vacuum delivery) |
|  | Surgical termination of pregnancy |
| Ophthalmology | Removal of foreign body from cornea |
| Orthopaedics | Bone biopsy |
|  | Injection in to joint |
|  | Muscle biopsy |
| Otolaryngology | Laryngoscopy (diagnostic or therapeutic) |
|  | Nasendoscopy (diagnostic) |
|  | Packing of cavity of nose |
| Thoracic surgery | Bronchoscopy (diagnostic) |
|  | Insertion of chest drain |
| Upper gastrointestinal surgery | Endoscopic retrograde cholangiopancreatography (diagnostic or therapeutic) |
|  | Liver biopsy |
|  | Oesophago-gastro-duodenoscopy (diagnostic or therapeutic) |
| Urology* | Bladder biopsy |
|  | Extracorporeal shock-wave lithotripsy |
|  | Flexible cystoscopy (diagnostic) |
|  | Percutaneous nephrostomy |
| Vascular surgery | Endovenous laser treatment for varicose veins |
|  | Insertion or removal of dialysis catheter |
|  | injection into varicose vein of leg |
|  | Transluminal (endovascular) procedures on arteries (diagnostic or therapeutic), including with open cut down to the artery |
|  | Transluminal (endovascular) procedures on veins (diagnostic or therapeutic) |
|  | Insertion or removal of Hickmann line |
| Other | Insertion of central venous catheter/ line |
|  | Insertion of chest drain |
|  | Lumbar (spinal) puncture |
|  | Percutaneous tracheostomy |
|  | Skin biopsy (including shave biopsy of skin) |
|  | Therapeutic epidural injection |
|  | Vacuum dressing |

*Transurethral resection of the prostate, transurethral resection of bladder tumour, rigid cystoscopy under general anaesthesia, and insertion of ureteric stent should be included.

**Table S2.** Ranked VTE rates alongside PE and DVT rates by specialty in all patients of any SARS-CoV-2 status

|  | VTE | | PE | | DVT | |
| --- | --- | --- | --- | --- | --- | --- |
| Neurosurgery | 37/2848 | 1.3% | 15/2848 | 0.5% | 27/2848 | 0.9% |
| Thoracic surgery | 21/2087 | 1.0% | 13/2087 | 0.6% | 8/2087 | 0.4% |
| Surgical oncology | 25/2699 | 0.9% | 11/2699 | 0.4% | 17/2699 | 0.6% |
| Colorectal surgery | 118/13,012 | 0.9% | 71/13,012 | 0.5% | 56/13,012 | 0.4% |
| General surgery | 99/10,925 | 0.9% | 54/10,925 | 0.5% | 55/10,925 | 0.5% |
| Orthopaedics | 175/20,815 | 0.8% | 86/20,815 | 0.4% | 99/20,815 | 0.5% |
| Spinal surgery | 24/2940 | 0.8% | 11/2940 | 0.4% | 15/2940 | 0.5% |
| Cardiac surgery | 20/2482 | 0.8% | 9/2482 | 0.4% | 12/2482 | 0.5% |
| Oesophagogastric | 25/3327 | 0.8% | 17/3327 | 0.5% | 13/3327 | 0.4% |
| Other surgery | 6/828 | 0.7% | 1/828 | 0.1% | 5/828 | 0.6% |
| Hepatobiliary surgery | 56/10,736 | 0.5% | 36/10,736 | 0.3% | 23/10,736 | 0.2% |
| Head & Neck surgery | 23/6515 | 0.4% | 10/6515 | 0.2% | 13/6515 | 0.2% |
| Gynaecology | 24/7629 | 0.3% | 12/7629 | 0.2% | 15/7629 | 0.2% |
| Vascular surgery | 25/8569 | 0.3% | 12/8569 | 0.1% | 16/8569 | 0.2% |
| Hernia surgery | 22/7812 | 0.3% | 17/7812 | 0.2% | 8/7812 | 0.1% |
| Obstetrics | 22/8967 | 0.2% | 4/8967 | 0.0% | 19/8967 | 0.2% |
| Plastic surgery | 6/2971 | 0.2% | 2/2971 | 0.1% | 5/2971 | 0.2% |
| Breast surgery | 8/5428 | 0.1% | 5/5428 | 0.1% | 5/5428 | 0.1% |
| Endocrine surgery | 4/2800 | 0.1% | 3/2800 | 0.1% | 1/2800 | 0.0% |
| Ophthalmology | 2/4315 | 0.0% | 1/4315 | 0.0% | 1/4315 | 0.0% |
| Dentistry | 0/281 | 0.0% | 0/281 | 0.0% | 0/281 | 0.0% |
| Missing | 27 |  |  |  |  |  |

**Table S3.** Adjusted regression model for factors associated with venous thromboembolism (shown in Figure 3).

|  | Odds Ratio | [95% Conf. | Interval] | p value |
| --- | --- | --- | --- | --- |
| SARS-CoV-2 status |  |  |  |  |
| No SARS-CoV-2 |  |  |  |  |
| Peri-operative SARS-CoV-2 | **1.48** | 1.08 | 2.03 | **0.016** |
| Recent SARS-CoV-2 | **1.96** | 1.16 | 3.33 | **0.012** |
| Previous SARS-CoV-2 | 1.66 | 0.90 | 3.04 | 0.103 |
| Pneumonia |  |  |  |  |
| No |  |  |  |  |
| Yes | **3.73** | 2.98 | 4.68 | **< 0.001** |
| Age; years |  |  |  |  |
| 18–49 |  |  |  |  |
| 50–69 years | **1.50** | 1.22 | 1.84 | **< 0.001** |
| ≥ 70 | **1.68** | 1.34 | 2.12 | **< 0.001** |
| Sex |  |  |  |  |
| Female |  |  |  |  |
| Male | 0.88 | 0.76 | 1.03 | 0.113 |
| ASA physical status |  |  |  |  |
| 1–2 |  |  |  |  |
| 3–5 | **2.03** | 1.70 | 2.42 | **< 0.001** |
| Smoker |  |  |  |  |
| No |  |  |  |  |
| Yes | 1.06 | 0.87 | 1.30 | 0.548 |
| Respiratory comorbidities |  |  |  |  |
| No |  |  |  |  |
| Yes | 1.15 | 0.93 | 1.42 | 0.197 |
| Congestive heart failure |  |  |  |  |
| No |  |  |  |  |
| Yes | 1.16 | 0.89 | 1.51 | 0.274 |
| Cerebral vascular disease |  |  |  |  |
| No |  |  |  |  |
| Yes | 1.15 | 0.87 | 1.52 | 0.320 |
| Chronic kidney disease |  |  |  |  |
| No |  |  |  |  |
| Yes | **1.49** | 1.12 | 1.99 | **0.007** |
| Ischaemic heart disease |  |  |  |  |
| No |  |  |  |  |
| Yes | 1.08 | 0.88 | 1.33 | 0.456 |
| Indication |  |  |  |  |
| Benign |  |  |  |  |
| Malignancy | **1.70** | 1.41 | 2.05 | **< 0.001** |
| Trauma | **1.59** | 1.29 | 1.95 | **< 0.001** |
| Obstetric | **0.62** | 0.38 | 1.00 | **0.050** |
| Grade of surgery |  |  |  |  |
| Minor |  |  |  |  |
| Major | **1.96** | 1.61 | 2.40 | **< 0.001** |
| Urgency |  |  |  |  |
| Elective |  |  |  |  |
| Emergency | **2.35** | 1.98 | 2.79 | **< 0.001** |
| Anaesthetic |  |  |  |  |
| Local / regional |  |  |  |  |
| General | **1.47** | 1.19 | 1.83 | **< 0.001** |
| Country income |  |  |  |  |
| High |  |  |  |  |
| Upper middle | 1.20 | 0.97 | 1.48 | 0.095 |
| Lower middle/low | **1.78** | 1.45 | 2.19 | **< 0.001** |

Peri-operative SARS-CoV-2, 7 days before to 30 days after surgery; recent SARS-CoV-2, 1–6 weeks before surgery; previous SARS-CoV-2, ≥ 7 weeks before surgery.

**Table S4.** Adjusted sub-group analysis for VTE in elective patients only.

|  | VTE | % | Odds Ratio | [95% Conf. | Interval] | p value |
| --- | --- | --- | --- | --- | --- | --- |
| SARS-CoV-2 status |  |  |  |  |  |  |
| No SARS-CoV-2 | 316 / 87,117 | 0.4% |  |  |  |  |
| Peri-operative SARS-CoV-2 | 20 / 965 | 2.1% | **2.10** | 1.27 | 3.48 | **0.004** |
| Recent SARS-CoV-2 | 10 / 604 | 1.7% | **2.93** | 1.52 | 5.64 | **0.001** |
| Previous SARS-CoV-2 | 5 / 857 | 0.6% | 1.51 | 0.62 | 3.68 | 0.366 |
| Pneumonia |  |  |  |  |  |  |
| No | 302 / 88,297 | 0.3% |  |  |  |  |
| Yes | 49 / 1246 | 3.9% | **5.54** | 3.92 | 7.83 | **< 0.001** |
| Age; years |  |  |  |  |  |  |
| 18–49 | 75 / 35,989 | 0.2% |  |  |  |  |
| 50–69 years | 152 / 33,642 | 0.5% | **1.71** | 1.27 | 2.31 | **< 0.001** |
| ≥ 70 | 124 / 19,911 | 0.6% | **2.18** | 1.56 | 3.06 | **< 0.001** |
| Sex |  |  |  |  |  |  |
| Female | 181 / 48,889 | 0.4% |  |  |  |  |
| Male | 170 / 40,651 | 0.4% | 0.95 | 0.76 | 1.18 | 0.616 |
| ASA physical status |  |  |  |  |  |  |
| 1–2 | 185 / 67,143 | 0.3% |  |  |  |  |
| 3–5 | 166 / 22,365 | 0.7% | **1.60** | 1.25 | 2.06 | **< 0.001** |
| Smoker |  |  |  |  |  |  |
| No | 292 / 75,485 | 0.4% |  |  |  |  |
| Yes | 58 / 13,811 | 0.4% | 1.11 | 0.82 | 1.48 | 0.504 |
| Respiratory comorbidities |  |  |  |  |  |  |
| No | 296 / 80,616 | 0.4% |  |  |  |  |
| Yes | 55 / 8860 | 0.6% | 1.16 | 0.85 | 1.57 | 0.351 |
| Congestive heart failure |  |  |  |  |  |  |
| No | 320 / 86,134 | 0.4% |  |  |  |  |
| Yes | 31 / 3385 | 0.9% | 1.28 | 0.86 | 1.90 | 0.225 |
| Cerebral vascular disease |  |  |  |  |  |  |
| No | 325 / 86,581 | 0.4% |  |  |  |  |
| Yes | 26 / 2938 | 0.9% | 1.33 | 0.88 | 2.03 | 0.177 |
| Chronic kidney disease |  |  |  |  |  |  |
| No | 333 / 87,616 | 0.4% |  |  |  |  |
| Yes | 18 / 1903 | 1.0% | 1.60 | 0.97 | 2.62 | 0.064 |
| Ischaemic heart disease |  |  |  |  |  |  |
| No | 289 / 81,605 | 0.4% |  |  |  |  |
| Yes | 62 / 7914 | 0.8% | 1.09 | 0.80 | 1.48 | 0.593 |
| Indication |  |  |  |  |  |  |
| Benign | 166 / 58,777 | 0.3% |  |  |  |  |
| Malignancy | 153 / 22,262 | 0.7% | **1.63** | 1.29 | 2.05 | **< 0.001** |
| Trauma | 27 / 4646 | 0.6% | **2.22** | 1.45 | 3.40 | **< 0.001** |
| Obstetric | 5 / 3855 | 0.1% | 0.75 | 0.29 | 1.94 | 0.555 |
| Grade |  |  |  |  |  |  |
| Minor | 58 / 34,452 | 0.2% |  |  |  |  |
| Major | 293 / 55,074 | 0.5% | **2.25** | 1.67 | 3.03 | **< 0.001** |
| Anaesthetic |  |  |  |  |  |  |
| Local / regional | 47 / 23,720 | 0.2% |  |  |  |  |
| General | 304 / 65,791 | 0.5% | **1.53** | 1.09 | 2.16 | **0.015** |
| Country income |  |  |  |  |  |  |
| High | 218 / 61,230 | 0.4% |  |  |  |  |
| Upper middle | 71 / 15,356 | 0.5% | **1.41** | 1.06 | 1.86 | **0.017** |
| Lower middle/low | 62 / 12,957 | 0.5% | **1.88** | 1.39 | 2.55 | **< 0.001** |

Peri-operative SARS-CoV-2, 7 days before to 30 days after surgery; recent SARS-CoV-2, 1–6 weeks before surgery; previous SARS-CoV-2, ≥ 7 weeks before surgery.

**Table S5.** Adjusted sub-group analysis for VTE in emergency patients only.

|  | VTE | % | Odds Ratio | [95% Conf. | Interval] | p value |
| --- | --- | --- | --- | --- | --- | --- |
| SARS-CoV-2 status |  |  |  |  |  |  |
| No SARS-CoV-2 | 350 / 36,471 | 1.0% |  |  |  |  |
| Peri-operative SARS-CoV-2 | 30 / 1352 | 2.2% | 1.26 | 0.84 | 1.88 | 0.267 |
| Recent SARS-CoV-2 | 5 / 349 | 1.4% | 1.15 | 0.47 | 2.84 | 0.761 |
| Previous SARS-CoV-2 | 6 / 291 | 2.1% | 1.85 | 0.81 | 4.24 | 0.146 |
| Pneumonia |  |  |  |  |  |  |
| No | 323 / 37,037 | 0.9% |  |  |  |  |
| Yes | 68 / 1426 | 4.8% | **2.94** | 2.20 | 3.94 | **< 0.001** |
| Age; years |  |  |  |  |  |  |
| 18–49 | 117 / 21,568 | 0.5% |  |  |  |  |
| 50–69 years | 127 / 9488 | 1.3% | **1.34** | 1.01 | 1.79 | **0.044** |
| ≥ 70 | 147 / 7406 | 1.0% | 1.37 | 0.99 | 1.88 | 0.054 |
| Sex |  |  |  |  |  |  |
| Female | 199 / 19,935 | 1.0% |  |  |  |  |
| Male | 192 / 18,527 | 1.0% | 0.81 | 0.65 | 1.00 | 0.051 |
| ASA physical status |  |  |  |  |  |  |
| 1–2 | 138 / 26,874 | 0.5% |  |  |  |  |
| 3–5 | 253 / 11,581 | 2.2% | **2.56** | 1.99 | 3.29 | **< 0.001** |
| Smoker |  |  |  |  |  |  |
| No | 324 / 31,707 | 1.0% |  |  |  |  |
| Yes | 66 / 6621 | 1.0% | 1.01 | 0.76 | 1.33 | 0.961 |
| Respiratory comorbidities |  |  |  |  |  |  |
| No | 329 / 35,070 | 0.9% |  |  |  |  |
| Yes | 62 / 3362 | 1.8% | 1.13 | 0.84 | 1.51 | 0.411 |
| Congestive heart failure |  |  |  |  |  |  |
| No | 350 / 36,825 | 1.0% |  |  |  |  |
| Yes | 41 / 1634 | 2.5% | 1.09 | 0.77 | 1.55 | 0.632 |
| Cerebral vascular disease |  |  |  |  |  |  |
| No | 357 / 36,880 | 1.0% |  |  |  |  |
| Yes | 34 / 1579 | 2.2% | 1.02 | 0.70 | 1.48 | 0.918 |
| Chronic kidney disease |  |  |  |  |  |  |
| No | 353 / 37,025 | 1.0% |  |  |  |  |
| Yes | 38 / 1434 | 2.7% | **1.46** | 1.02 | 2.08 | **0.038** |
| Ischaemic heart disease |  |  |  |  |  |  |
| No | 319 / 35,171 | 0.9% |  |  |  |  |
| Yes | 72 / 3288 | 2.2% | 1.07 | 0.80 | 1.42 | 0.644 |
| Indication |  |  |  |  |  |  |
| Benign | 183 / 19,940 | 0.9% |  |  |  |  |
| Malignancy | 46 / 1851 | 2.5% | **1.75** | 1.25 | 2.46 | **0.001** |
| Trauma | 143 / 10,590 | 1.4% | **1.44** | 1.14 | 1.83 | **0.003** |
| Obstetric | 19 / 6082 | 0.3% | **0.52** | 0.30 | 0.93 | **0.026** |
| Grade |  |  |  |  |  |  |
| Minor | 74 / 14,142 | 0.5% |  |  |  |  |
| Major | 317 / 24,313 | 1.3% | **1.78** | 1.36 | 2.34 | **< 0.001** |
| Anaesthetic |  |  |  |  |  |  |
| Local / regional | 78 / 11,997 | 0.7% |  |  |  |  |
| General | 313 / 26,445 | 1.2% | **1.37** | 1.03 | 1.82 | **0.029** |
| Country income |  |  |  |  |  |  |
| High | 266 / 23,821 | 1.1% |  |  |  |  |
| Upper middle | 47 / 6214 | 0.8% | 0.94 | 0.68 | 1.29 | 0.690 |
| Lower middle/low | 78 / 8428 | 0.9% | **1.59** | 1.20 | 2.11 | **0.001** |

Peri-operative SARS-CoV-2, 7 days before to 30 days after surgery; recent SARS-CoV-2, 1–6 weeks before surgery; previous SARS-CoV-2, ≥ 7 weeks before surgery.

**Table S6.** Adjusted sub-group analysis for VTE in major surgery patients only.

|  | VTE | % | Odds Ratio | [95% Conf. | Interval] | p value |
| --- | --- | --- | --- | --- | --- | --- |
| SARS-CoV-2 status |  |  |  |  |  |  |
| No SARS-CoV-2 | 545 / 76,391 | 0.7% |  |  |  |  |
| Peri-operative SARS-CoV-2 | 42 / 1622 | 2.6% | **1.55** | 1.10 | 2.18 | **0.013** |
| Recent SARS-CoV-2 | 12 / 668 | 1.8% | **1.92** | 1.07 | 3.46 | **0.030** |
| Previous SARS-CoV-2 | 11 / 709 | 1.6% | **2.04** | 1.11 | 3.75 | **0.022** |
| Pneumonia |  |  |  |  |  |  |
| No | 515 / 77,192 | 0.7% |  |  |  |  |
| Yes | 95 / 2199 | 4.3% | **3.33** | 2.60 | 4.27 | **< 0.001** |
| Age; years |  |  |  |  |  |  |
| 18–49 | 149 / 34,667 | 0.4% |  |  |  |  |
| 50–69 years | 233 / 27,029 | 0.9% | **1.54** | 1.22 | 1.93 | **< 0.001** |
| ≥ 70 | 228 / 17,694 | 1.3% | **1.74** | 1.34 | 2.24 | **< 0.001** |
| Sex |  |  |  |  |  |  |
| Female | 317 / 47,115 | 0.7% |  |  |  |  |
| Male | 293 / 32,273 | 0.9% | 0.94 | 0.79 | 1.11 | 0.452 |
| ASA physical status |  |  |  |  |  |  |
| 1–2 | 265 / 54,948 | 0.5% |  |  |  |  |
| 3–5 | 345 / 24,424 | 1.4% | **1.84** | 1.51 | 2.22 | **< 0.001** |
| Smoker |  |  |  |  |  |  |
| No | 512 / 67,483 | 0.8% |  |  |  |  |
| Yes | 96 / 11,649 | 0.8% | 1.01 | 0.80 | 1.27 | 0.933 |
| Respiratory comorbidities |  |  |  |  |  |  |
| No | 513 / 71,485 | 0.7% |  |  |  |  |
| Yes | 97 / 7848 | 1.2% | 1.19 | 0.95 | 1.50 | 0.137 |
| Congestive heart failure |  |  |  |  |  |  |
| No | 559 / 75,824 | 0.7% |  |  |  |  |
| Yes | 51 / 3556 | 1.4% | 0.96 | 0.71 | 1.31 | 0.807 |
| Cerebral vascular disease |  |  |  |  |  |  |
| No | 555 / 76,244 | 0.7% |  |  |  |  |
| Yes | 55 / 3136 | 1.8% | 1.31 | 0.98 | 1.75 | 0.073 |
| Chronic kidney disease |  |  |  |  |  |  |
| No | 570 / 77,388 | 0.7% |  |  |  |  |
| Yes | 40 / 1992 | 2.0% | 1.34 | 0.96 | 1.89 | 0.090 |
| Ischaemic heart disease |  |  |  |  |  |  |
| No | 503 / 71,676 | 0.7% |  |  |  |  |
| Yes | 107 / 7704 | 1.4% | 1.03 | 0.82 | 1.30 | 0.784 |
| Indication |  |  |  |  |  |  |
| Benign | 264 / 41,865 | 0.6% |  |  |  |  |
| Malignancy | 183 / 17,730 | 1.0% | **1.77** | 1.45 | 2.17 | **< 0.001** |
| Trauma | 140 / 10,733 | 1.3% | **1.39** | 1.10 | 1.76 | **0.005** |
| Obstetric | 23 / 9061 | 0.3% | **0.53** | 0.32 | 0.89 | **0.016** |
| Urgency |  |  |  |  |  |  |
| Elective | 293 / 55,074 | 0.5% |  |  |  |  |
| Emergency | 317 / 24,313 | 1.3% | **2.43** | 2.00 | 2.94 | **< 0.001** |
| Anaesthetic |  |  |  |  |  |  |
| Local / regional | 94 / 17,605 | 0.5% |  |  |  |  |
| General | 516 / 61,747 | 0.8% | 1.29 | 1.00 | 1.66 | 0.053 |
| Country income |  |  |  |  |  |  |
| High | 393 / 51,205 | 0.8% |  |  |  |  |
| Upper middle | 99 / 13,758 | 0.7% | 1.20 | 0.95 | 1.51 | 0.125 |
| Lower middle/low | 118 / 14,428 | 0.8% | **1.76** | 1.40 | 2.20 | **< 0.001** |

Peri-operative SARS-CoV-2, 7 days before to 30 days after surgery; recent SARS-CoV-2, 1–6 weeks before surgery; previous SARS-CoV-2, ≥ 7 weeks before surgery.

**Table S7**. Adjusted sub-group analysis for VTE in minor surgery patients only.

|  | VTE | % | Odds Ratio | [95% Conf. | Interval] | p value |
| --- | --- | --- | --- | --- | --- | --- |
| SARS-CoV-2 status |  |  |  |  |  |  |
| No SARS-CoV-2 | 121 / 47,178 | 0.3% |  |  |  |  |
| Peri-operative SARS-CoV-2 | 8 / 695 | 1.2% | 1.10 | 0.48 | 2.48 | 0.828 |
| Recent SARS-CoV-2 | 3 / 285 | 1.1% | 1.69 | 0.50 | 5.72 | 0.398 |
| Previous SARS-CoV-2 | 0 / 439 | 0 | No events |  |  |  |
| Pneumonia |  |  |  |  |  |  |
| No | 110 / 48,125 | 0.2% |  |  |  |  |
| Yes | 22 / 472 | 4.7% | **7.56** | 4.36 | 13.10 | **< 0.001** |
| Age; years |  |  |  |  |  |  |
| 18–49 | 43 / 22,877 | 0.2% |  |  |  |  |
| 50–69 years | 46 / 16,095 | 0.3% | 1.28 | 0.81 | 2.02 | 0.296 |
| ≥ 70 | 43 / 9624 | 0.5% | 1.48 | 0.87 | 2.52 | 0.148 |
| Sex |  |  |  |  |  |  |
| Female | 63 / 21,697 | 0.3% |  |  |  |  |
| Male | 69 / 26,899 | 0.3% | 0.71 | 0.50 | 1.02 | 0.062 |
| ASA physical status |  |  |  |  |  |  |
| 1–2 | 58 / 39,055 | 0.2% |  |  |  |  |
| 3–5 | 74 / 9518 | 0.8% | **3.16** | 2.05 | 4.86 | **< 0.001** |
| Smoker |  |  |  |  |  |  |
| No | 104 / 39,697 | 0.3% |  |  |  |  |
| Yes | 28 / 8777 | 0.3% | 1.35 | 0.87 | 2.09 | 0.181 |
| Respiratory comorbidities |  |  |  |  |  |  |
| No | 112 / 44,183 | 0.3% |  |  |  |  |
| Yes | 20 / 4374 | 0.5% | 0.98 | 0.59 | 1.62 | 0.925 |
| Congestive heart failure |  |  |  |  |  |  |
| No | 111 / 47,118 | 0.2% |  |  |  |  |
| Yes | 21 / 1462 | 1.4% | **2.18** | 1.28 | 3.73 | **0.004** |
| Cerebral vascular disease |  |  |  |  |  |  |
| No | 127 / 47,200 | 0.3% |  |  |  |  |
| Yes | 5 / 1380 | 0.4% | 0.47 | 0.18 | 1.19 | 0.110 |
| Chronic kidney disease |  |  |  |  |  |  |
| No | 116 / 47,235 | 0.3% |  |  |  |  |
| Yes | 16 / 1345 | 1.2% | **1.91** | 1.08 | 3.39 | **0.026** |
| Ischaemic heart disease |  |  |  |  |  |  |
| No | 105 / 45,083 | 0.2% |  |  |  |  |
| Yes | 27 / 3497 | 0.8% | 1.34 | 0.83 | 2.18 | 0.232 |
| Indication |  |  |  |  |  |  |
| Benign | 85 / 36,839 | 0.2% |  |  |  |  |
| Malignancy | 16 / 6382 | 0.3% | 1.08 | 0.61 | 1.90 | 0.801 |
| Trauma | 30 / 4501 | 0.7% | **2.50** | 1.61 | 3.89 | **< 0.001** |
| Obstetric | 1 / 874 | 0.1% | 0.69 | 0.09 | 5.10 | 0.718 |
| Urgency |  |  |  |  |  |  |
| Elective | 58 / 34,452 | 0.2% |  |  |  |  |
| Emergency | 74 / 14,142 | 0.5% | **2.06** | 1.40 | 3.04 | **< 0.001** |
| Anaesthetic |  |  |  |  |  |  |
| Local / regional | 31 / 18,111 | 0.2% |  |  |  |  |
| General | 101 / 30,472 | 0.3% | **1.82** | 1.19 | 2.77 | **0.005** |
| Country income |  |  |  |  |  |  |
| High | 91 / 33,843 | 0.3% |  |  |  |  |
| Upper middle | 19 / 7798 | 0.3% | 1.16 | 0.69 | 1.95 | 0.572 |
| Lower middle/low | 22 / 6956 | 0.3% | **1.85** | 1.12 | 3.05 | **0.016** |

Peri-operative SARS-CoV-2, 7 days before to 30 days after surgery; recent SARS-CoV-2, 1–6 weeks before surgery; previous SARS-CoV-2, ≥ 7 weeks before surgery.
